# Logic‐Gated Release of a Dual Payload from a β‐Lactam Mechanophore with a Rotaxane Actuator

**DOI:** 10.1002/anie.202512698

**Published:** 2025-07-14

**Authors:** Lei Chen, Guillaume De Bo

**Affiliations:** ^1^ Department of Chemistry University of Manchester Oxford Road Manchester M13 9PL United Kingdom

**Keywords:** Controlled release, Drug delivery, Logic gate, Mechanophore, Rotaxane

## Abstract

The force‐controlled release of functional molecules has attracted interest due to its potential use in drug delivery and self‐healing applications. Clever mechanochemical devices have been developed to that effect, but most are limited by the payload (type, number) they can deliver and their responsiveness to a single stimulus (force). However, the applications envisaged above require the precise delivery of a complex payload. In this context, a device able to release a diverse set of cargo molecules in response to a varied number of stimuli is desired. Here, we present a rotaxane‐based device that can release a dual payload in response to two different stimuli: force and water. The first payload is released mechanically, while the release of the second cargo requires both force and water in a way reminiscent of a logic AND gate. Our device is built around a β‐lactam mechanophore that is activated by a rotaxane actuator to release a ketene and reveal an imine that anchors the second cargo to the axle of the rotaxane; hydrolysis of the imine releases the second cargo molecule. The concept was demonstrated with the efficient release of two drugs, hymecromone and gemcitabine, both in solution and in bulk. We anticipate that our device should find applications in a variety of contexts where precise release is required.

## Graphical abstract

A rotaxane device operates as a logic AND gate to release a single or dual cargo payload upon application of force in the absence or the presence of water, respectively. This concept was demonstrated with the release of a ketene and varied drugs, paving the way for precision release applications.

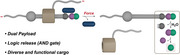

The force‐controlled release of small molecules^[^ ^1^, ^2^ ^]^ offers great promises for drug delivery, damage sensing, or self‐healing.^[^ ^3^ ^]^ Clever mechanoresponsive systems based on covalent mechanophores,^[^ ^4^, ^5^, ^6^, ^7^, ^8^, ^9^, ^10^, ^11^ ^]^ cages,^[^ ^12^ ^]^ or rotaxanes^[^ ^3^, ^13^, ^14^, ^15^, ^16^ ^]^ have been described. However, most of these systems can only release one type of cargo at a time upon application of a single stimulus (force). Hence, the use of more sophisticated release systems for the precise targeting of released molecules is desired. Dual cargo release has been shown on disulfide^[^ ^17^ ^]^ and furan‐based mechanophores,^[^ ^18^ ^]^ and the release of cargo molecules has been achieved upon application of both force and UV light stimuli,^[^ ^19^ ^]^ a strategy also used in mechanochromic applications.^[^ ^20^ ^]^ However, a system combining both characteristics does not exist. The multistimulus responsiveness can be achieved by mechanical gating,^[^ ^21^, ^22^, ^23^ ^]^ whereby a mechanosensitive group needs to be activated to reveal/expose a second functional group sensitive to a second stimulus. This strategy has been recently used to imbue recycling properties to polymers by exposing hydrolysable functionality upon mechanical activation.^[^ ^24^, ^25^, ^26^, ^27^, ^28^, ^29^ ^]^

We have recently shown how the pushing activation^[^ ^14^ ^]^ mode of a rotaxane actuator can selectively activate orthogonal bonds in 4‐membered ring mechanophores, either parallel or perpendicular to the main axis.^[^ ^15^ ^]^ In a β‐lactam mechanophore, for example, it can lead to the release of alkene or ketene derivatives, leaving isocyanate or imine functionalities at the end of the axle respectively. The hydrolysable nature of the imine moiety offers the possibility of releasing a second cargo molecule, an amine, in the presence of water. Here, we present a molecular device built as a logic AND gate, able to release a dual molecular payload consisting of a ketene, eventually hydrolyzed into a carboxylic acid, *and* an amine *if* water is also present (Figure 1 and Supplementary Video 1). To expand the diversity of the second cargo load, we have connected the cargo molecule to the β‐lactam core via a self‐immolative *para*‐aminobenzyloxycarbonyl linker which, upon imine hydrolysis, collapses to release amino‐alcohol **4**, CO_2_, and the desired amine or alcohol cargo molecule. We have demonstrated this concept with the release of hymecromone, an antispasmodic used to treat digestive disorders and a fluorophore, and gemcitabine, a wide‐spectrum anticancer drug. The logic‐gated release of the dual cargo payload was demonstrated in solution and bulk, with high efficiency. We anticipate that this new release mechanism should prove useful in a variety of controlled release applications in materials, for example, with the release of healing agents on the newly formed surface of a crack exposed to the atmosphere, and in medicine, with the precise delivery of drugs.

**Figure 1:**
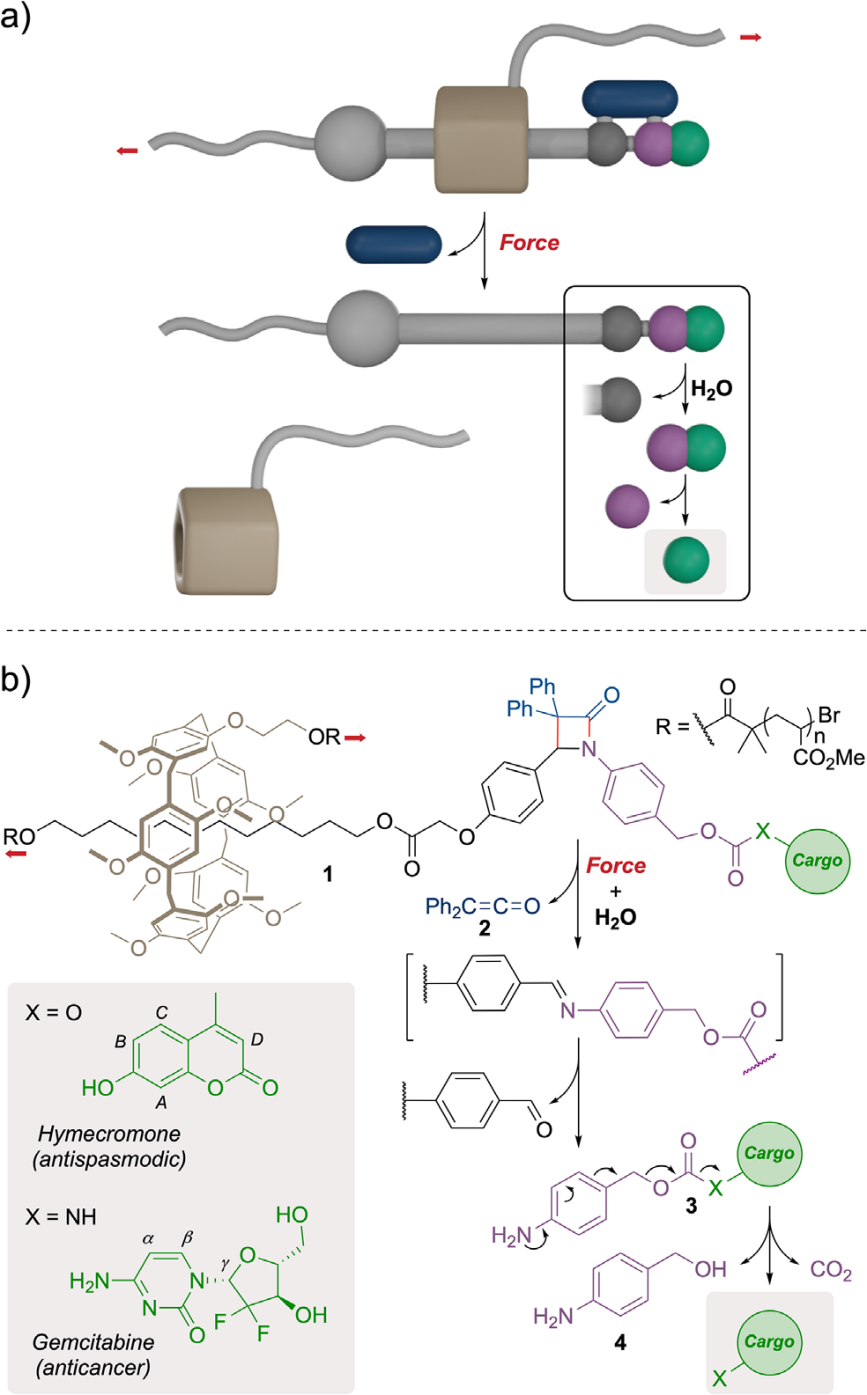
Logic‐gated release of a dual payload from a β‐lactam mechanophore with a rotaxane actuator. a) Cartoons illustrating the conceptual dual payload (blue rod and green ball) being released upon application of force and water stimuli. b) Rotaxane actuation of a β‐lactam mechanophore triggers a retro‐[2 + 2] cycloaddition that releases ketene **2** and reveals an imine functionality that is hydrolyzed in the presence of water to liberate self‐immolative amine **3**, which ultimately releases the desired cargo molecules. Red arrows indicate the direction of the force.

As a proof of concept, we initially investigated the release of a simple amine, anisidine **11** in a logic AND gate (Figure 2), using mechanical force and water as sequential stimuli, both in solution, by sonication, and in bulk, by compression and ball‐mill grinding (BMG).

**Figure 2:**
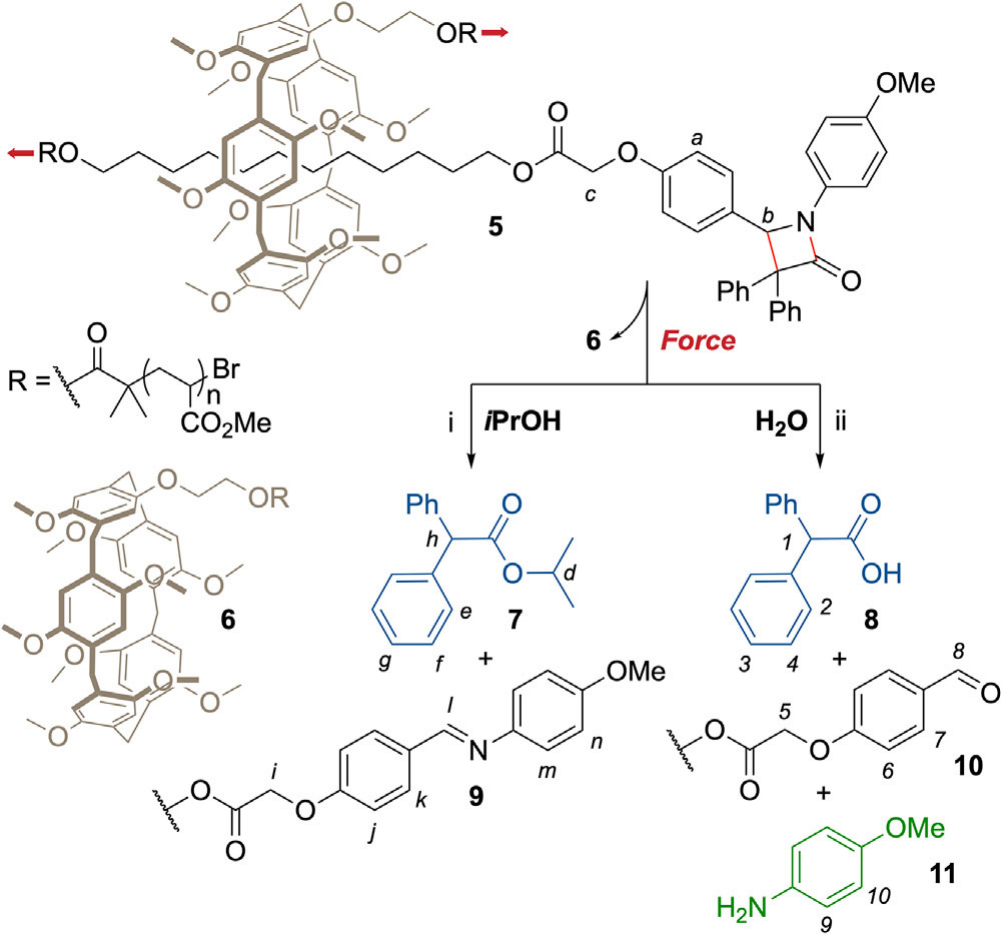
A rotaxane device activating a β‐lactam mechanophore acts as an AND gate for the release of anisidine in the presence of force and water. Activation of **5** in the presence of *i*PrOH affords ester **7** and imine **9**. Activation in the presence of H_2_O affords acid **8** and liberates anisidine **11** after imine hydrolysis. Conditions: US (20 kHz, 13.0 W cm^−2^, 1s ON/1s OFF), CD_3_CN/ *i*PrOH: 9/1 (i) or CD_3_CN/H_2_O: 9/1 (ii), 5–10 °C, 105 min.

Our model cargo‐releasing device **5** is based on a rotaxane architecture built from a pillar[5]arene (P5) macrocycle and a C12 axle stoppered with a β‐lactam mechanophore on one side and a poly(methyl acrylate) polymer (PMA) on the other.^[^ ^15^ ^]^ Conveniently, the cargo is loaded onto the rotaxane prior to the polymerization step, which reinforces the modularity of this design (see Supporting Information section 3.2). The second actuating PMA chain is anchored to the macrocycle which, upon mechanical activation, enables the pulling of the macrocycle toward the mechanophore. The macrocycle pushing against the *gem*‐diphenyl unit of the β‐lactam ring provokes the scission of the 4‐membered ring's bonds perpendicular to the axle in a formal retro‐[2 + 2] cycloaddition.^[^ ^15^ ^]^ Ketene **2** is released upon mechanical activation, while the amine can only be released from the axle upon hydrolysis of the imine in the presence of H_2_O (Figure 2).

The activation of chain‐centered macromolecular rotaxane **5** was initially performed in solution using high‐intensity ultrasound in the presence of *i*PrOH or H_2_O (Figure 2). We used CD_3_CN as the sonication solvent to allow for the ^1^H NMR analysis of the crude mixture. After sonication, the solvent was removed and the solid residue washed with MeOH to remove any nonpolymeric material. ^1^H NMR analysis of the polymer fraction shows that the imine moiety is left intact upon activation in CD_3_CN/*i*PrOH (*i‐n*, Figure 3a_ii,iii_), indicating the stability of this moiety in the absence of water, while activation in CD_3_CN/H_2_O leads to the complete hydrolysis of the imine group and the release of anisidine **11**, as evidenced by the emergence of peaks pertaining to aldehyde **10** in the post‐sonication spectrum (*5‐8*, Figure 3a_iv,v_). The release of the ketene and anisidine molecules was detected in the crude post‐sonication mixture. The ketene, which is quickly trapped by *i*PrOH or H_2_O upon release, is identified as the corresponding ester (*d‐h*, Figure 3b_i,ii_) or carboxylic acid (*1‐4*, Figure 3c_i,ii_). Anisidine (**11**) was found in the reaction performed in CD_3_CN/H_2_O (*9‐10*, Figure 3c_i–iii_), confirming its effective release when both stimuli (force and H_2_O) are applied, as expected from a logic AND gate.

**Figure 3:**
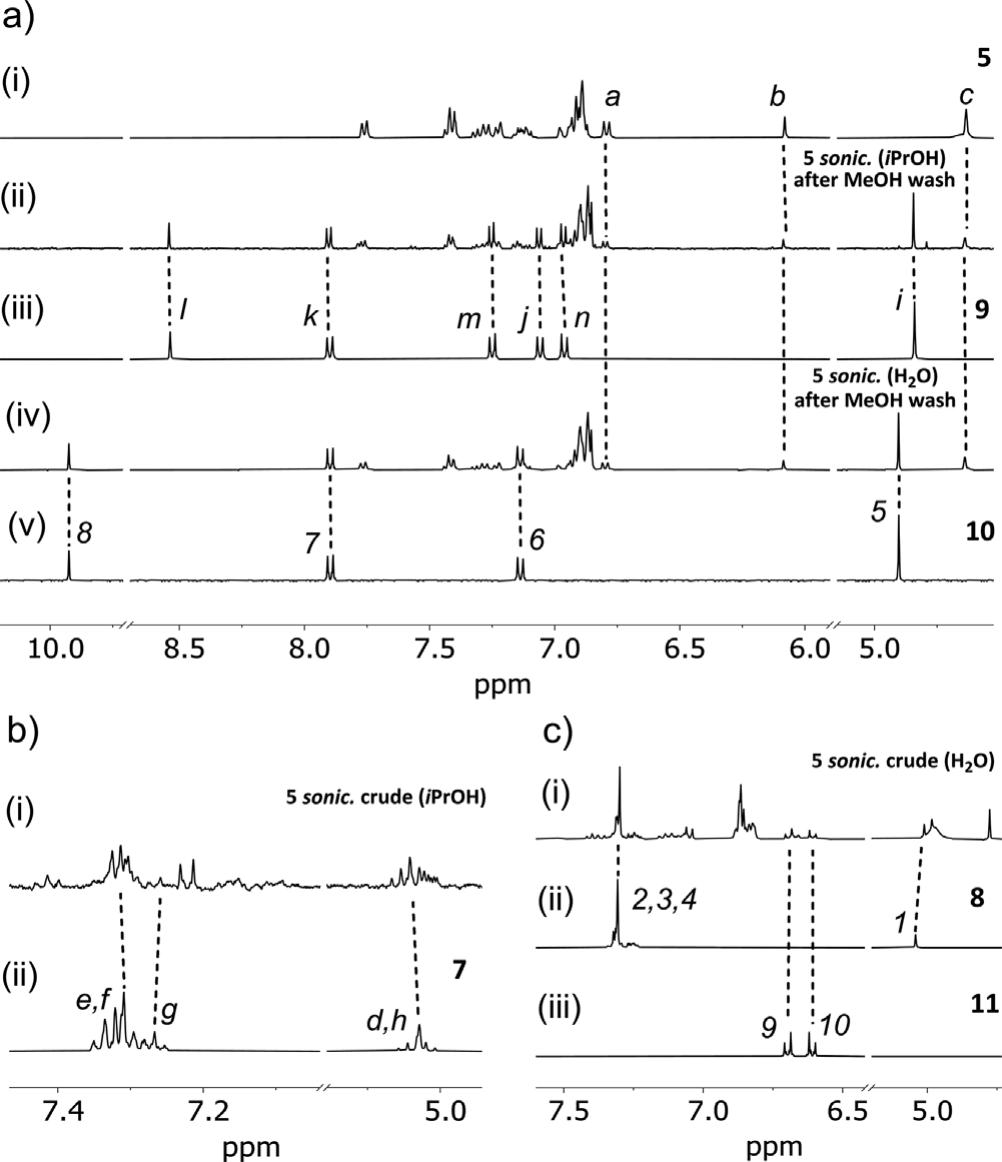
Partial ^1^H NMR (400 MHz, acetone‐*d_6_* or CD_3_CN/ROH, 298 K) spectra. a) Rotaxane **5** before (i) and after sonication in CD_3_CN/*i*PrOH (ii) or CD_3_CN/H_2_O (iv) along with reference compounds **9** (iii) and **10** (v). b) Crude mixture of sonication of **5** in CD_3_CN/*i*PrOH (i), along with reference compound **7** (ii). c) Crude mixture of sonication of **5** in CD_3_CN/H_2_O (i), along with reference compound **8** (ii) and **11** (iii). The assignments correspond to the lettering shown in Figure 2.

We then tested the logic release of amine **11** in bulk by BMG (30 Hz, liquid nitrogen, 60 min) and compression (manual press, 0.74 GPa, ≥ 60 min/cycle, 10 cycles). The BMG activation was performed on a dry (vacuum, 45°C, 48h) or wet (**5**/H_2_O: 1/1 w/w) sample (see Supporting Information section 7), and provided a similar level of activation to the sonication activation (Table 1). Remarkably, only a small amount of imine hydrolysis is observed in the dry sample (presumably coming from adventitious water in the experimental setup), confirming that the logic release of anisidine can be efficiently achieved in the solid state. Despite the lower rate and intensity of impact imparted by compression, this activation method led to the release of anisidine in 5% of the rotaxane (Table 1). In this case, no care was taken to dry the sample as this activation requires long reaction times and frequent sample manipulations (see Supporting Information); as a result, some of the activated mechanophore experienced imine hydrolysis. Noninterlocked control polymers confirmed the necessary presence of the rotaxane actuator and both stimuli to elicit the release of anisidine, both in solution and bulk (see Tables S2–S4).

**Table 1:** Activation parameters for polymer **5**.

Activation	Pre‐sonic. *Mn* *(kDa)/ Đ*	Post‐sonic. *Mn* *(kDa)/ Đ* ^b)^	Species after activation (%)^b)^, ^c)^	
			Imine	Aldehyde
Sonication (*i*PrOH)	155/1.13	41/1.26	64 ± 1	0
Sonication (H_2_O)	138/1.18	34/1.29	0	63 ± 1
BMG (Dry)^a)^	155/1.13	6/1.51	60 ± 3	5 ± 0
BMG (H_2_O)^a)^	155/1.13	9/1.36	0	68 ± 2
Compression	138/1.18	35/2.41	15 ± 11	5 ± 2

^a)^ Sample was dried or mixed with water before use. See Supporting Information section 7 for details

^b)^ Average value from two experiments. See Supporting Information table S2 for details

^c)^ See Supporting Information section 8 for calculation details.

To expand the scope of cargo released from this logic device, we introduced a self‐immolative *para*‐aminobenzyloxycarbonyl spacer to connect alcohol or amine cargo molecules to the force‐responsive β‐lactam core via a carbonate or carbamate linkage, respectively (Figure 1). To illustrate this concept, we elected to release hymecromone (HYM), a fluorophore and an antispasmodic drug, connected from its alcohol functionality, and gemcitabine (GEM), a wide‐spectrum chemotherapy medication, connected through its cytosine's amine (Figure 1). The release of both drugs was confirmed upon application of both required stimuli by sonication of **1*_HYM_*** and **1*_GEM_*** in a CD_3_CN/H_2_O mixture (Figure 1 and Table 2), as both hymecromone (*A‐D*, Figure 4a) and gemcitabine (*α‐γ*, Figure 4b) were identified by ^1^H NMR analysis of the MeOH extract. Incidentally, **1*_HYM_*** is also mechanochromic as the fluorescence of hymecromone is revealed upon release (see Figure S9). The release of both drugs was similarly confirmed in the solid state upon BMG activation of wet samples of **1*_HYM_*** and **1*_GEM_*** (Table 2). The lower gemcitabine content in the latter is probably resulting from the drug's degradation in milling conditions. Overall, these results demonstrate the efficacy of the logic approach for the release of functional molecules.

**Table 2:** Activation parameters for mechanophore **1**.

Activation		Pre‐sonic. *Mn* *(kDa)/ Đ*	Post‐sonic. *Mn* *(kDa)/ Đ* ^a)^	Species after activation (%)^a)^, ^b)^	
				Aldehyde	Drug
**1*_HYM_***	Sonication (H_2_O)	159/1.32	32/1.33	61 ± 1	61 ± 0
	BMG (H_2_O)		10/1.59	51 ± 1	51 ± 1
**1*_GEM_***	Sonication (H_2_O)	147/1.30	34/1.34	53 ± 0	54 ± 1
	BMG (H_2_O)		11/1.44	41 ± 0	19 ± 4

^a)^ Average value from two parallel experiments. See Supporting Information tables S2 and S4 for details

^b)^ See Supporting Information section 8 for calculation details.

**Figure 4:**
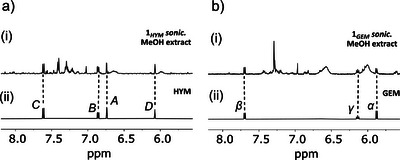
Partial ^1^H NMR (400 MHz, acetone‐*d_6_* or indicated, 298 K) spectra of mechanophore **1*_HYM_*** a): post‐sonication MeOH extract (i), along with reference HYM (ii), and mechanophore **1*_GEM_*** b): post‐sonication MeOH extract (i), along with reference GEM (ii) in CD_3_CN/H_2_O. The assignments correspond to the lettering shown in Figure 1.

In conclusion, we have described a new rotaxane device for the controlled release of a dual payload upon application of two different stimuli: force and water. This system is built around a β‐lactam ring, which acts as a mechanical gate for a hydrolysable imine junction. Mechanical activation of the β‐lactam by the rotaxane actuator provokes scission of the ring and the release of diphenylketene, the first payload. The newly revealed imine can then be hydrolyzed in the presence of water to release an amine, the second payload. We have demonstrated that this device acts as a logic AND gate for the release of the amine cargo. The structural diversity of the second payload was expanded with the introduction of a self‐immolative spacer connecting either alcohol or amine cargo molecules to the hydrolysable imine, as shown with the release of two drugs. The released was achieved in both solution and bulk with up to 68% efficiency. The dual payload offers the possibility of enhancing (e.g. the released acid could accelerate the imine hydrolysis) or supplementing (e.g. tagging) the action of the main cargo, while the necessity of applying two different stimuli increases the accuracy of timing/position of release (e.g. release of healing agents on the newly formed surface of a crack exposed to the atmosphere). Finally, this device has the potential to be extended into a multimechanophore system as the rotaxane architecture allows for the sequential activation of multiple cargo units.^[^ ^3^ ^]^ We anticipate that our device should find applications in a variety of contexts where precise release is required.

## Conflict of Interests

The authors declare no conflict of interest.

## Supporting information



Supporting Information

Supporting Information

## Data Availability

The data that support the findings of this study are available in the Supporting Information of this article.
