# Supplementary material for: Logic‐Gated Release of a Dual Payload from a β‐Lactam Mechanophore with a Rotaxane Actuator
Source: Angew Chem Int Ed Engl. 2025 Jul 14;64(37):e202512698. doi: 10.1002/anie.202512698 (PMC12416451; doi:10.1002/anie.202512698)
Supplement: Supplementary file 2 — Supporting Information [file ANIE-64-e202512698-s002.pdf]

Supporting Information

Logic-Gated Release of a Dual Payload from a β-Lactam Mechanophore with a Rotaxane Actuator

*Lei Chen and Guillaume De Bo**

Department of Chemistry, University of Manchester, Oxford Road, Manchester, M13 9PL, UK

*E-mail: guillaume.debo@manchester.ac.uk

1 Table of Contents

1 Table of Contents ... 2
2 General Experimental Details ... 5
3 Synthesis of Mechanophore, Rotaxane, Control and Reference Compounds ... 7
3.1 Synthesis of Mechanophore Compounds ... 7
3.1.1 Synthetic Routes to S6 ... 7
3.1.2 Synthesis of S2 ... 7
3.1.3 Synthesis of S5 ... 8
3.1.4 Synthesis of S6 ... 8
3.2 Synthesis of Rotaxane and Control Compounds ... 9
3.2.1 Synthetic Routes to S11 and S14 ... 9
3.2.2 Synthesis of S10 ... 9
3.2.3 Synthesis of S11 ... 10
3.2.4 Synthesis of S12 ... 11
3.2.5 Synthesis of S14 ... 12
3.2.6 Synthetic Routes to S17 ... 13
3.2.7 Synthesis of S16 ... 13
3.2.8 Synthesis of S17 ... 14
3.3 Synthesis of Reference Compounds ... 15
3.3.1 Synthesis of 7 ... 15
4 Synthesis of Polymers ... 16
4.1 Representative Procedure for SET-LRP of Methyl Acrylate Using Mechanophore Initiators ... 16
4.2 Synthesis of Mechanophore and Control Polymers ... 16
4.2.1 Synthesis of Polymer 5_{-138} and 5_{-155} ... 16
4.2.2 Synthesis of Polymer 1_{HYM} ... 17
4.2.3 Synthesis of Polymer 1_{GEM} ... 17
4.2.4 Synthesis of Polymer S20 ... 18
4.2.5 Synthesis of Polymer S21 ... 18
4.2.6 Synthesis of Polymer 9_{-112} and 9_{-114} ... 19
4.3 Synthesis of Reference Polymers ... 19
4.3.1 Synthesis of Polymer 10 ... 19
4.4 SEC Data for Synthesised Polymers ... 20
4.5 SEC Traces for Mechanophore and Control Polymers ... 21
4.6 SEC Traces for Reference Polymers ... 22
5 Mechanophore Activation via Ultrasound ... 23
5.1 General Procedure for Sonication Experiments ... 23
5.2 Sonication of Mechanophore Polymer 5_{-138} ... 23
5.3 Sonication of Mechanophore Polymer 5_{-155} at the Absence of Water ... 26
5.4 Sonication of Mechanophore Polymer 1_{HYM} ... 29
5.5 Sonication of Mechanophore Polymer 1_{GEM} ... 32
5.6 Sonication of Control Polymers S20 and S21 ... 35
5.7 Sonication of Control Polymer 9_{-112} ... 38
5.8 Analysis of Impurities in MeOH Extract from Polymer after Activation ... 40
6 Activation in Bulk by Compression ... 41
6.1 General Procedure for Compression Experiments ... 41
6.2 Compression of Mechanophore Polymer 5_{-138} ... 41
6.3 Compression of Control Polymer S20 ... 44
6.4 Compression of Control Polymer 9_{-112} ... 45
7 Activation in Bulk by BMG ... 46
7.1 General Procedure for BMG Experiments ... 46
7.2 BMG of Mechanophore Polymer 5_{-155} in Dry Condition ... 46
7.3 BMG of Mechanophore Polymer 5_{-155} in Wet Condition ... 49

7.4 BMG of Mechanophore Polymer 1_{HYM} in Wet Condition 52
7.5 BMG of Mechanophore Polymer 1_{GEM} in Wet Condition 55
7.6 BMG of Mechanophore Polymer S20 in Dry Condition 58
7.7 BMG of Mechanophore Polymer S20 in Wet Condition 60
7.8 BMG of Mechanophore Polymer S21 under Wet Conditions 62
7.9 BMG of Mechanophore Polymer 9_{-114} under Dry Conditions 64
7.10 BMG of Mechanophore Polymer 9_{-114} in Wet Condition 65
7.11 Stability of compounds 4 and 11 in BMG in Wet Condition 66
8 Calculation of Extent of Mechanophore Activation 67
8.1 Calculations for Polymer 5_{-138} in Sonication Experiments 67
8.2 Calculations for Polymer 5_{-155} in Sonication Experiments in the Absence of Water 68
8.3 Calculations for Polymer 5_{-138} in Compression Experiments, Polymer 5_{-155} in BMG Experiments, and Polymers S20, 9_{-112}, 9_{-114} in all types of activation 69
8.4 Calculations for Polymers 1_{HYM} and S21 70
8.5 Calculations for Polymer 1_{GEM} in the Sonication 71
8.6 Calculations for Polymer 1_{GEM} in the BMG 72
8.7 Summary of Mechanophores Activated by Sonication 74
8.8 Summary of Mechanophores Activated by Compression 75
8.9 Summary of Mechanophores Activated by BMG 76
9 NMR Spectra 77
9.1 Small Molecule NMR Spectra 77
9.1.1 Spectra of S2 77
9.1.2 Spectra of S5 78
9.1.3 Spectra of S6 79
9.1.4 Spectra of S10 80
9.1.5 Spectra of S11 81
9.1.6 Spectra of S12 82
9.1.7 Spectra of S14 83
9.1.8 Spectra of S16 85
9.1.9 Spectra of S17 86
9.1.10 Spectra of 7 87
9.2 Polymer NMR Spectra 88
9.2.1 Spectra of polymer 5_{-138} 88
9.2.2 Spectra of polymer 5_{-155} 89
9.2.3 Spectra of polymer 1_{HYM} 90
9.2.4 Spectra of polymer 1_{GEM} 91
9.2.5 Spectra of polymer S20 91
9.2.6 Spectra of polymer S21 92
9.2.7 Spectra of polymer 9_{-112} 93
9.2.8 Spectra of polymer 9_{-114} 94
9.2.9 Spectra of polymer 10 95
9.3 Post-Sonication NMR Spectra 98
9.3.1 Post-Sonication ^{1}H NMR Spectra of Polymer 5_{-138} (Run 1) 98
9.3.2 Post-Sonication ^{1}H NMR Spectra of Polymer 5_{-138} (Run 2) 99
9.3.3 Post-Sonication ^{1}H NMR Spectra of Polymer 5_{-155} (Run 1) 101
9.3.4 Post-Sonication ^{1}H NMR Spectra of Polymer 5_{-155} (Run 2) 102
9.3.5 Post-Sonication ^{1}H NMR Spectra of Polymer 1_{HYM} (Run 1) 104
9.3.6 Post-Sonication ^{1}H NMR Spectra of Polymer 1_{HYM} (Run 2) 105
9.3.7 Post-Sonication ^{1}H NMR Spectra of Polymer 1_{GEM} (Run 1) 107
9.3.8 Post-Sonication ^{1}H NMR Spectra of Polymer 1_{GEM} (Run 2) 109
9.3.9 Post-Sonication ^{1}H NMR Spectra of Polymer S20 111
9.3.10 Post-Sonication ^{1}H NMR Spectra of Polymer S21 112
9.3.11 Post-Sonication ^{1}H NMR Spectra of Polymer 9_{-112} 114
9.4 Post-Compression NMR Spectra 116

9.4.1 Post-Compression ^{1}H NMR Spectra of Polymer 5_{-138} (Run 1) 116
9.4.2 Post-Compression ^{1}H NMR Spectra of Polymer 5_{-138} (Run 2) 117
9.4.3 Post-Compression ^{1}H NMR Spectra of Polymer S20 119
9.4.4 Post-Compression ^{1}H NMR Spectra of Polymer 9_{-112} 120
9.5 Post-BMG NMR Spectra....................................122
9.5.1 Post-BMG (Dry) ^{1}H NMR Spectra of Polymer 5_{-155} (Run 1) 122
9.5.2 Post-BMG (Dry) ^{1}H NMR Spectra of Polymer 5_{-155} (Run 2) 123
9.5.3 Post-BMG (Wet) ^{1}H NMR Spectra of Polymer 5_{-155} (Run 1) 125
9.5.4 Post-BMG (Wet) ^{1}H NMR Spectra of Polymer 5_{-155} (Run 2) 126
9.5.5 Post-BMG (Wet) ^{1}H NMR Spectra of Polymer 1_{HYM} (Run 1)....................................128
9.5.6 Post-BMG (Wet) ^{1}H NMR Spectra of Polymer 1_{HYM} (Run 2)....................................129
9.5.7 Post-BMG (Wet) ^{1}H NMR Spectra of Polymer 1_{GEM} (Run 1)....................................131
9.5.8 Post-BMG (Wet) ^{1}H NMR Spectra of Polymer 1_{GEM} (Run 2)....................................132
9.5.9 Post-BMG (Dry) ^{1}H NMR Spectra of Polymer S20 134
9.5.10 Post-BMG (Wet) ^{1}H NMR Spectra of Polymer S20 135
9.5.11 Post-BMG (Wet) ^{1}H NMR Spectra of Polymer S21 137
9.5.12 Post-BMG (Dry) ^{1}H NMR Spectra of Polymer 9_{-114} 138
9.5.13 Post-BMG (Wet) ^{1}H NMR Spectra of Polymer 9_{-114} 140
10 Mass Spectrometry Isotopic Patterns 142
10.1 Isotopic distribution of S10 142
10.2 Isotopic distribution of S11 142
10.3 Isotopic distribution of S12 143
10.4 Isotopic distribution of S14 143
10.5 Isotopic distribution of S16 144
10.6 Isotopic distribution of S17 144
11 References 145

2 General Experimental Details

Unless otherwise stated, all reagents and solvents were purchased from commercial suppliers and used without further purification. Dry solvents were obtained by passing through an activated alumina column on a Phoenix SDS solvent drying system (JC Meyer Solvent Systems, CA, USA). Compounds **S1**,[1] **S9**, **S15**,[2] **S7**[3] and **S8**[4] were prepared according to literature procedure. Compounds **S18**, **S19**, **S22**, **S23** and polymers **5**, **S20**, **9**, **10** were described in a previous paper.[5] Polymers **5**$_{\text{-138}}$, **S20**, **9**$_{\text{-114}}$ and **10** were reused in this study.

Size exclusion chromatography (SEC) analyses were performed in THF solution (1.0 mg mL^{-1}) at 40 °C using a GPC/SEC Agilent 1260 Infinity II with 2 × PL gel 10 µm mixed-C and a PL gel 500 Å column, and equipped with a differential refractive index (DRI) detector employing narrow polydispersity polystyrene standards (Agilent Technologies) as a calibration reference. Samples were filtered through a Whatman Puradisc 4 mm syringe filter with 0.45 µm PTFE membrane before injection to equipment, and experiments were carried out with injection volume of 50 µL, flow rate of 1 mL min^{-1}. Results were analyzed using *n*-dodecane as internal marker using Agilent GPC/SEC Software Version 2.2. (N,N'-dimethylacetamide was also used in the system of SEC in the indicated case, in which the condition of method is column temperature of 50 °C, injection volume of 100 µL, flow rate of 0.8 mL min^{-1} and toluene as internal marker.)

Ultrasound experiments were performed using a SONIVCX-130 ultrasonic processor equipped with a 6 mm diameter solid probe. The distance between the titanium tip and the bottom of microwave vial (5 mL) was 1 cm. The ultrasonic intensity was calibrated using the method outlined by Hickenboth *et al*.[6] Solid-state compression experiments were carried out by placing the appropriate polymer material within an International Crystal Laboratories 13mm KBr pellet die and force was applied using a Specac Atlas 15T manual hydraulic press. BMG experiments were performed using Retsch Mixer Mill MM 400 equipped with a stainless steel milling jar (Cavity: diameter × length = 13 × 45 mm).

Analytical TLC was performed on precoated silica gel plates (0.25 mm thick, 60 F254, Merck, Germany) and observed under UV light or stained with a potassium permanganate base solution. Preparative TLC was performed on precoated silica gel plates: 500 µm or 2000 µm, UNIPLATE GF, Analtech Inc., DE, USA. Flash column chromatography was performed with silica gel 60 (230-400 mesh) from Sigma-Aldrich. ^{1}H and ^{13}C NMR spectra were recorded on a Bruker Avance III 700 MHz, 600 MHz, 500 MHz or 400 MHz Prodigy instrument. Chemical shifts are reported in parts per million (ppm) from high to low frequency and referenced to the residual solvent resonance. Coupling constants (*J*) are reported in Hertz (Hz) and splitting patterns are designated as follows: b = broad, s = singlet, d = doublet, t = triplet, q = quartet, p = pentet and m = multiplet. ^{1}H and ^{13}C assignments were made using 1D or 2D NMR methods (HSQC, HMBC, COSY). Mass spectra were obtained through the Mass Spectrometry services in the Department of Chemistry at the University of Manchester.

Abbreviations: APCI: atmospheric-pressure chemical ionization; BMG: ball-mill grinding; CoGEF: constrained geometries simulate external force; DCM: dichloromethane; DMAc: N,N'-dimethylacetamide; DMAP: 4-dimethylaminopyridine; DMSO: dimethylsulfoxide; ESI: electrospray ionization; HRMS: high resolution mass spectrometry; MA: methyl acrylate; MS: mass spectrometry;

Me_6TREN: tris[2-(dimethylamino)ethyl]amine; PE: petroleum ether; TBS: tert-Butyldimethylsilyl; THF: tetrahydrofuran; TLC: thin layer chromatography; TMSCl: chlorotrimethylsilane.

3 Synthesis of Mechanophore, Rotaxane, Control and Reference Compounds

3.1 Synthesis of Mechanophore Compounds

3.1.1 Synthetic Routes to S6

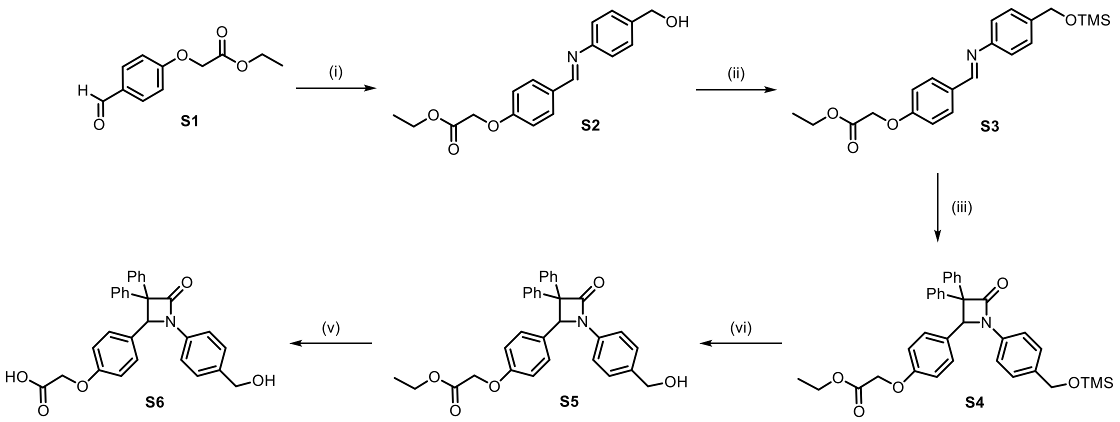

Scheme S1. Synthetic routes to **S6**. Conditions: (i) 4-Aminobenzyl alcohol, DCM, 40 °C, rotavap, 98% yield; (ii) TMSCl, Et_3N, DCM, r.t., 1 h; (iii) Diphenylacetyl chloride, r.t., 16 h; (vi) TBAF, r.t., 10 min, 76% yield for three steps; (v) $LiOH{\cdot}H_2O$, THF/MeOH/H_2O (2/2/1), r.t., 1 h, 88% yield.

3.1.2 Synthesis of S2

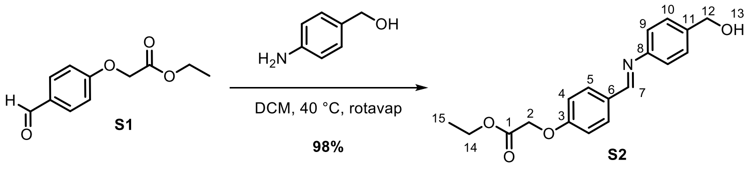

S1 (50 mg, 240 µmol, 1.0 eq.) and p-anisidine (30 mg, 240 µmol, 1.0 eq.) were dissolved in DCM (5 mL) in a round bottom flask. Solvent was evaporated on the rotavap at 40 °C. This process of dissolution and evapoartion was repeated three times to yield **S2** as a pale yellow solid (74 mg, 236 µmol, 98% yield).

^{1}H NMR (400 MHz, Acetone-d_6, 298 K) δ = 8.52 (s, 1H, H_7), 7.95 – 7.88 (m, 2H, H_5), 7.41 – 7.34 (m, 2H, H_{10}), 7.23 – 7.17 (m, 2H, H_9), 7.11 – 7.04 (m, 2H, H_4), 4.83 (s, 2H, H_2), 4.64 (d, J = 4.1 Hz, 2H, H_{12}), 4.28 – 4.16 (m, 3H, $H_{13,14}$), 1.26 (t, J = 7.1 Hz, 3H, H_{15}).
^{13}C NMR (101 MHz, Acetone-d_6, 298 K) δ = 169.07 (C_1), 161.54 (C_3), 159.70 (C_7), 151.94 (C_8), 140.86 (C_{11}), 131.17 ($C_{5,6}$), 128.28 (C_{10}), 121.52 (C_9), 115.63 (C_4), 65.74 (C_2), 64.40 (C_{12}), 61.59 (C_{14}), 14.45 (C_{15}).
HRMS-ESI(+): 314.1377 $[M+H]^+$, calculated for $C_{18}H_{19}NO_4H^+$: 314.1387.

3.1.3 Synthesis of S5

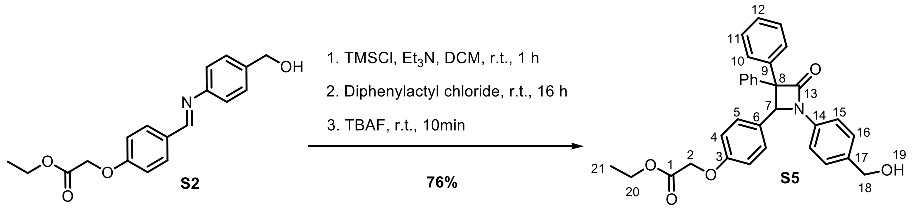

To a solution of **S2** (70 mg, 224 µmol, 1.0 eq.) and TMSCl (31 mg, 291 µmol, 1.3 eq.) in DCM (2 mL) was added Et_3N (136 mg, 1341 µmol, 6.0 eq.). After stirring at room temperature for 1 h, diphenylacetyl chloride (77 mg, 335 µmol, 1.5 eq.) was added and continued to stir for 16 h at room temperature. Then TBAF solution (447 µL, 1 M in THF, 2.0 eq.) was added and the mixture stirred for a further 15 min. After that, the solvent was evaporated. The residue was purified via column chromatography (SiO_2, PE/Acetone, 5/1) to yield **S5** as a white powder (86 mg, 170 µmol, 76% yield).

^{1}H NMR (400 MHz, Acetone-d_6, 298 K) δ = 7.80 – 7.71 (m, 2H, H_{10}), 7.41 (t, J = 8.4 Hz, 4H, $H_{11,15}$) , H_1, 7.33 – 7.25 (m, 3H, $H_{12,16}$), 7.23 – 7.14 (m, 4H, $H_{5,10'}$), 7.11 – 7.02 (m, 3H, $H_{11',12'}$), 6.77 – 6.70 (m, 2H, H_4), 6.07 (s, 1H, H_7), 4.60 (s, 2H, H_2), 4.54 (d, J = 5.9 Hz, 2H, H_{18}), 4.16 (q, J = 7.1 Hz, 2H, H_{20}), 4.11 (t, J = 5.8 Hz, 1H, H_{19}), 1.21 (t, J = 7.1 Hz, 3H, H_{21}).
^{13}C NMR (101 MHz, Acetone-d_6, 298 K) δ = 169.14 (C_1), 167.45 (C_{13}), 158.86 (C_3), 142.27 (C_9), 139.24 (C_{17}), 139.03 ($C_{9'}$), 137.16 (C_{14}), 130.01 (C_5), 129.49 (C_{11}), 129.17 ($C_{10'}$), 128.88 (C_6), 128.74 ($C_{11'}$), 128.31 (C_{10}), 128.17 ($C_{12,15}$), 127.52 ($C_{12'}$), 118.20 (C_{16}), 115.23 (C_4), 72.90 (C_8), 66.78 (C_7), 65.75 (C_2), 64.24 (C_{18}), 61.42 (C_{20}), 14.45 (C_{21}).
HRMS-ESI(+): 530.1929 $[M+Na]^+$, calculated for $C_{32}H_{29}NO_5Na^+$: 530.1938.

3.1.4 Synthesis of S6

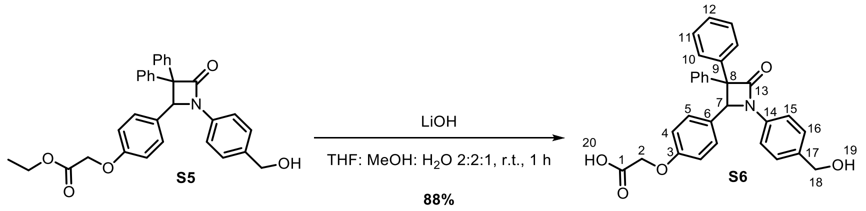

To a solution of **S5** (30 mg, 59 µmol, 1.0 eq.) in a mixture of THF (1 mL), MeOH (1 mL) and water (0.5 mL) was added $LiOH{\cdot}H_2O$ (50 mg, 1182 µmol, 20.0 eq.). The mixture was stirred at room temperature for 1 h. After evaporating the organic solvent in the mixture by rotavap, the pH of this solution was adjusted to ~2 by addition of aqueous 1 M HCl. The mixture was then filtered to yield **S6** as a white powder (25 mg, 52 µmol, 88% yield).

^{1}H NMR (400 MHz, Acetone-d_6, 298 K) δ = 7.79 – 7.71 (m, 2H, H_{10}), 7.44 – 7.36 (m, 4H, $H_{11,15}$), 7.33 – 7.24 (m, 3H, $H_{12,16}$), 7.22 – 7.15 (m, 4H, $H_{15,10'}$), 7.11 – 7.00 (m, 3H, $H_{11',12'}$), 6.79 – 6.72 (m, 2H, H_4), 6.07 (s, 1H, H_7), 4.60 (s, 2H, H_2), 4.53 (s, 2H, H_{18}).
^{13}C NMR (101 MHz, Acetone-d_6, 298 K) δ = 169.88 (C_1), 167.46 (C_{13}), 158.87 (C_3), 142.29 (C_9), 139.19 (C_{17}), 139.02 ($C_{9'}$), 137.16 (C_{14}), 130.01 (C_5), 129.49 (C_{11}), 129.16 ($C_{10'}$), 128.82 (C_6), 128.74 ($C_{11'}$), 128.30 (C_{10}), 128.18 (C_{15}), 128.16 (C_{12}), 127.52 ($C_{12'}$), 118.21 (C_{16}), 115.22 (C_4), 72.89 (C_8), 66.81 (C_7), 65.35 (C_2), 64.12 (C_{18}).
HRMS-ESI(+): 502.1614 $[M+Na]^+$, calculated for $C_{30}H_{25}NO_5Na^+$: 502.1625.

3.2 Synthesis of Rotaxane and Control Compounds

3.2.1 Synthetic Routes to S11 and S14

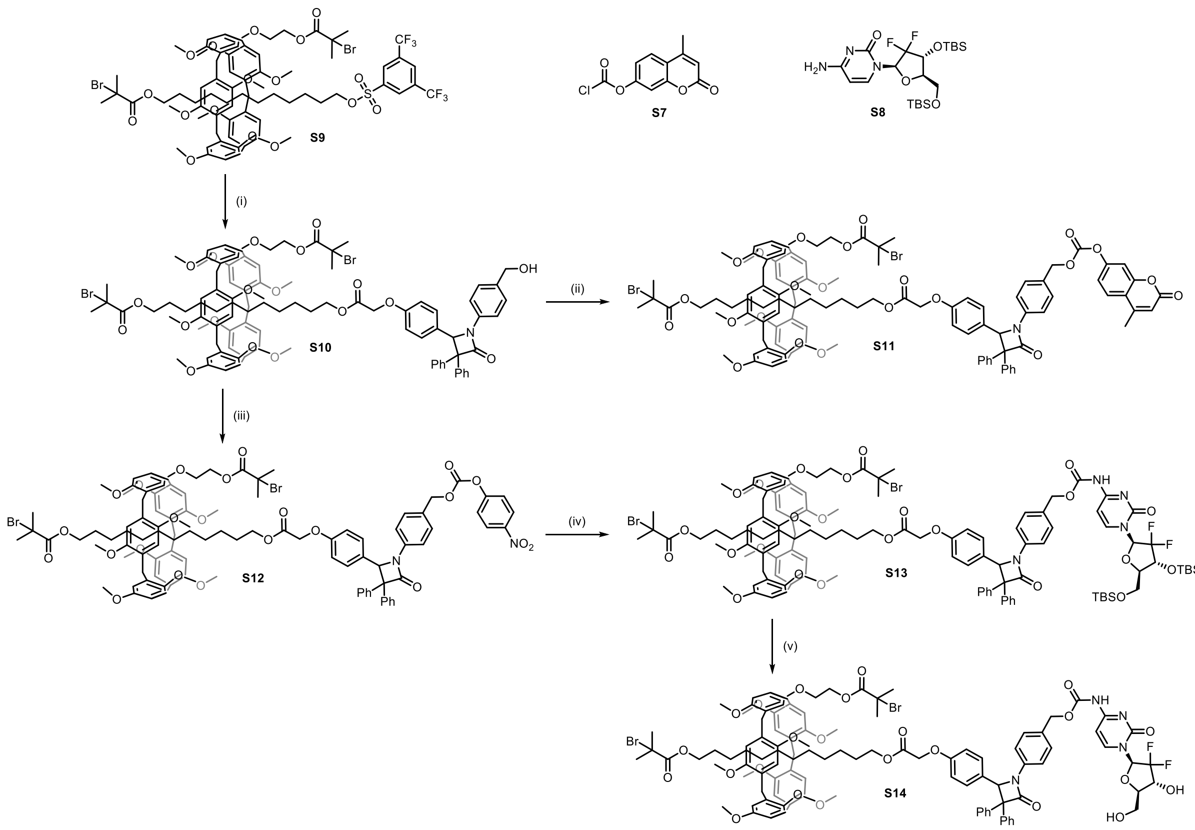

Scheme S2. Synthetic routes to **S11** and **S14**. Conditions: (i) **S6**, K_2CO_3, 18-crown-6, Acetone, r.t., 16 h, 80% yield; (ii) **S7**, Pyridine, DCM, r.t., 16 h, 54% yield; (iii) 4-Nitrophenyl chloroformate, Pyridine, DCM, r.t., 16 h, 91% yield; (iv) **S8**, DMAP, Pyridine, r.t., 16 h; (v) TBAF, r.t., 15 min, 30% yield for two steps.

3.2.2 Synthesis of S10

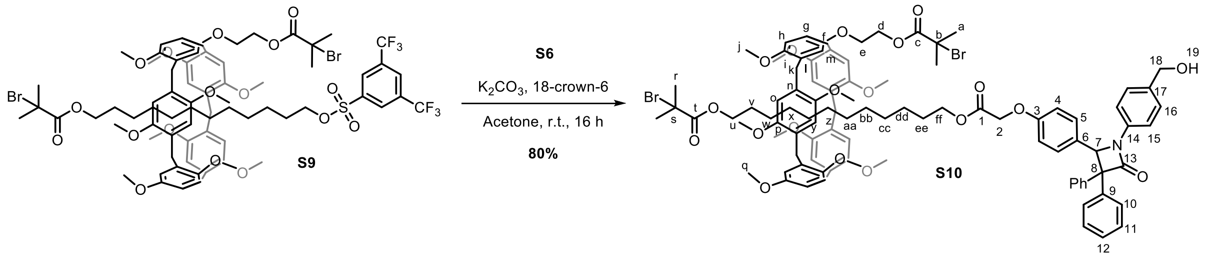

To a solution of **S6** (4.6 mg, 10 µmol, 1.0 eq.) in acetone (1 mL) was added K_2CO_3 (1.3 mg, 10 µmol, 1.0 eq.) and 18-crown-6 (2.5 mg, 10 µmol, 1.0 eq.). The mixture was stirred for 2 h at room temperature. **S9** (15 mg, 10 µmol, 1.0 eq.) was added and the mixture stirred for a further 16 h at room temperature. The solution was filtered, and the filtrate concentrated under vacuum. The residue was purified by preparative TLC (500 µm, PE/acetone, 5/42, eluted twice) to yield **S10** as a white powder (14 mg, 8 µmol, 84% yield).

^{1}H NMR (500 MHz, Acetone-d_6, 298 K) δ = 7.79 – 7.72 (m, 2H, H_{10}), 7.46 – 7.38 (m, 4H, $H_{11,15}$), 7.34 – 7.24 (m, 5H, $H_{12,16,5}$), 7.24 – 7.19 (m, 2H, $H_{10'}$), 7.16 – 7.06 (m, 3H, $H_{11',12'}$), 7.00 – 6.86 (m, 10H, $H_{h,m,o}$), 6.82 – 6.76 (m, 2H, H_4), 6.13 – 6.09 (s, s, 1H, H_7), 4.84 – 4.76 (m, 1H, H_d), 4.68 – 4.59 (m, 2H, H_2), 4.55 – 4.50 (m, 2H, H_{18}), 4.44 – 4.36 (m, 1H, H_d), 4.33 – 4.27 (m, 1H, H_e), 4.13 – 4.04 (m, 2H, $H_{e,19}$), 3.96 – 3.88 (m, 2H, H_u), 3.87 – 3.66 (m, 37H, $H_{j,q,k}$), 3.44 – 3.36 (m, 2H, H_{ff}), 2.00 – 1.94 (m, 12H, $H_{a,r}$), 1.19 – 1.12 (m, 2H, H_v), 0.81 – 0.73 (m, 2H, H_z), 0.70 – 0.56 (m, 4H, $H_{aa,y}$), 0.33 – 0.15 (m, 8H, $H_{w,ee,x,bb}$), -0.53 – -0.65 (m, 2H, H_{cc}), -0.83 – -0.96 (m, 2H, H_{dd}).
^{13}C NMR (126 MHz, Acetone-d_6, 298 K) δ = 172.13 (C_c), 171.90 (C_t), 169.21, 169.18 (C_1), 167.44 (C_{13}), 158.90 (C_3), 151.57, 151.15, 151.12, 151.04, 151.01, 151.01 ($C_{i,p}$), 149.98 (C_f), 142.29, 142.28 (C_9), 139.28, 139.27 (C_{17}), 139.08, 139.07 ($C_{9'}$), 137.15 (C_{14}), 130.16, 129.51, 129.46, 129.25, 129.07, 128.85, 128.81, 128.70, 128.67, 128.62, 128.61, 128.57, 128.55, 128.33, 128.32, 128.19 ($C_{5,6,10,10',11,11',12,15,g,l,n}$), 127.59 ($C_{12'}$), 118.24 ($C_{16}$), 115.34, 115.24, 114.11, 114.08, 113.74, 113.73, 113.69, 113.66, 113.61, 113.59, 113.56, 113.52, 113.50 ($C_{4,h,mo}$), 72.94, 72.93 (C_8), 67.14, 67.09 ($C_{e,u}$), 66.79, 66.76 (C_7), 66.46, 66.45 (C_{ff}), 65.98, 65.95 (C_2), 65.48 (C_d), 64.23, 64.22 (C_{18}), 57.53 (C_s), 57.15 (C_b), 55.83, 55.78, 55.74, 55.71, 55.69, 55.62, 55.60, 55.59 ($C_{j,q}$), 31.44 (C_{aa}), 31.29 (C_z), 31.08, 31.07 ($C_{a,r}$), 31.01, 30.94 ($C_{bb,y}$), 30.27 – 29.42 ($C_{k,cc,x}$, overlapped with solvent peak), 29.11 (C_v), 28.73 (C_{ee}), 25.60, 25.59 (C_w), 24.36, 24.33 (C_{dd}).
HRMS-ESI(+): 1762.6067 $[M+Na]^+$, calculated for $C_{96}H_{111}Br_2N_2O_{19}Na^+$: 1762.6009.

3.2.3 Synthesis of S11

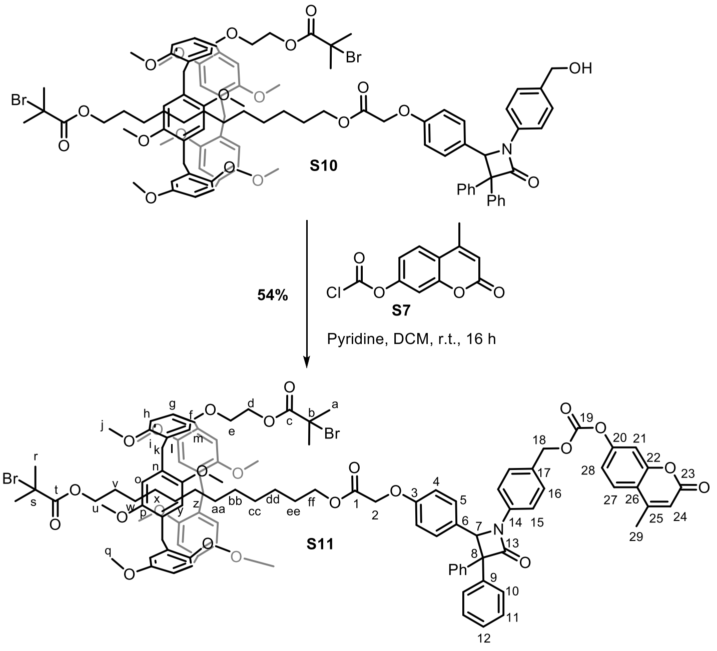

To a solution of **S10** (4.0 mg, 2 µmol, 1.0 eq.) and **S7** (3.3 mg, 14 µmol, 6.0 eq.) in DCM (0.5 mL) was added pyridine (1.1 mg, 14 µmol, 6.0 eq.). The mixture was then stirred at room temperature for 16 h. After the solvent was evaporated, the residue was purified via by preparative TLC (500 µm, PE/DCM/Acetone, 4/4/0.4, eluted twice) to yield **S11** as a white powder (2.4 mg, 1 µmol, 54% yield).

^{1}H NMR (700 MHz, Acetone-d_6, 298 K) δ = 7.79 – 7.75 (m, 3H, $H_{27,10}$), 7.53 – 7.49 (m, 2H, H_{15}), 7.48 – 7.44 (m, 2H, H_{16}), 7.44 – 7.40 (m, 2H, H_{11}), 7.34 – 7.26 (m, 3H, $H_{12,5}$), 7.24 – 7.21 (m, 3H, $H_{10',21}$), 7.20 – 7.18 (m, 1H, H_{28}), 7.16 – 7.11 (m, 2H, $H_{11'}$), 7.11 – 7.08 (m, 1H, $H_{12'}$), 6.99 – 6.87 (m, 10H, $H_{h,m,o}$), 6.83 – 6.78 (m, 2H, H_4), 6.29 (q, J = 1.0 Hz, 1H, H_{24}), 6.17 – 6.15 (s, s, 1H, H_7), 5.24 – 5.21 (s, s, 2H, H_{18}), 4.81 – 4.76 (m, 1H, H_d), 4.69 – 4.60 (m, 2H, H_2), 4.42 – 4.37 (m, 1H, H_d), 4.31 – 4.26 (m, 1H, H_e), 4.10 – 4.05 (m, 1H, H_e), 3.95 – 3.90 (m, 2H, H_u), 3.82 – 3.69 (m, 37H, $H_{j,q,k}$), 3.40 – 3.35 (m, 2H, H_{ff}), 2.47 (t, J = 1.0

Hz, 3H, H_{29}), 1.99 – 1.96 (m, 12H, $H_{a,r}$), 1.19 – 1.17 (m, 2H, H_v), 0.79 – 0.75 (m, 2H, H_z), 0.67 – 0.58 (m, 4H, $H_{aa,y}$), 0.37 – 0.30 (m, 2H, H_w), 0.30 – 0.23 (m, 2H, H_x), 0.23 – 0.14 (m, 4H, $H_{ee,bb}$), -0.58 – -0.67 (m, 2H, H_{cc}), -0.89 – -0.98 (m, 2H, H_{dd}).

^{13}C NMR (176 MHz, Acetone-d_6, 298 K) δ = 172.15 (C_c), 171.92 (C_t), 169.23, 169.21 (C_1), 167.76 (C_{13}), 160.23 (C_{23}), 159.02 (C_3), 155.07 (C_{22}), 154.33 (C_{20}), 153.54, 153.52 (C_{19}), 153.16 (C_{25}), 151.60, 151.20, 151.19, 151.17, 151.15, 151.09, 151.07, 151.05, 151.04 ($C_{i,p}$), 150.00 (C_f), 142.15 (C_9), 138.96, 138.95 ($C_{9'}$), 138.73, 138.72 (C_{14}), 131.66, 131.64 (C_{17}), 130.56, 130.54 (C_{16}), 130.20 (C_5), 129.56, 129.49, 129.24, 128.87, 128.73, 128.70, 128.65, 128.64, 128.61, 128.60, 128.59, 128.34, 128.33, 128.27 ($C_{12,10,10',11,11',12,g,l,n}$), 127.67 ($C_{12'}$), 127.06 ($C_{27}$), 118.85 ($C_{26}$), 118.55 ($C_{15}$), 118.17 ($C_{28}$), 115.39, 115.35, 115.08, 114.15, 114.12, 113.79, 113.76, 113.74, 113.69, 113.65, 113.63, 113.60, 113.57, 113.54 ($C_{4,24,h,m,o}$), 110.32 (C_{21}), 73.19, 73.18 (C_8), 70.87 (C_{18}), 67.17, 67.09 ($C_{e,u}$), 66.89, 66.87 (C_7), 66.50, 66.48 (C_{ff}), 66.04, 66.00 (C_2), 65.51, 65.50 (C_d), 57.55 (C_s), 57.17 (C_b), 55.86, 55.82, 55.81, 55.80, 55.76, 55.74, 55.71, 55.65, 55.63, 55.61, 55.51 ($C_{j,q}$), 31.43 (C_{aa}), 31.30 (C_z), 31.11, 31.09 ($C_{a,r}$), 31.01, 30.94 ($C_{bb,y}$), 30.43 – 29.44 ($C_{k,cc,x}$, overlapped with solvent peak), 29.14 (C_v), 28.72 (C_{ee}), 25.68, 25.67, 25.66 (C_w), 24.35, 24.30 (C_{dd}), 18.58 (C_{29}).

HRMS-ESI(+): 1964.6245 $[M+Na]^+$, calculated for $C_{107}H_{117}Br_2NO_{23}Na^+$: 1964.6275.

3.2.4 Synthesis of S12

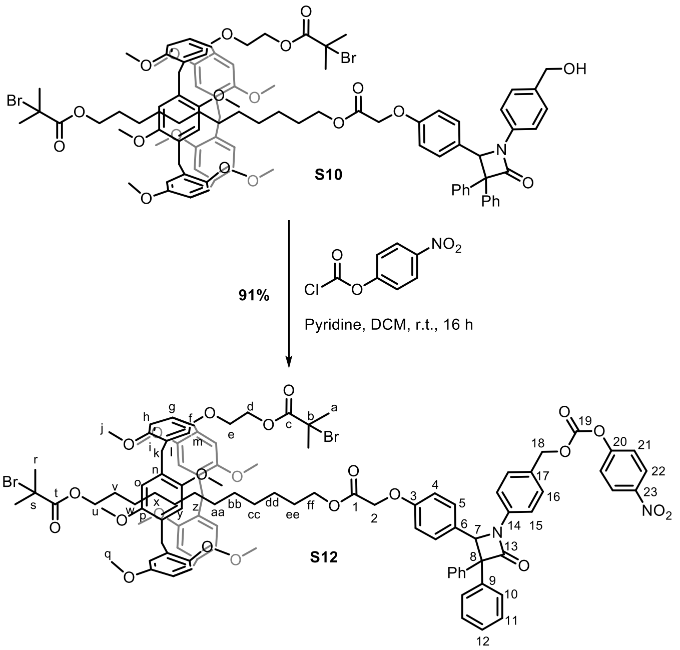

To a solution of **S10** (6.0 mg, 3 µmol, 1.0 eq.) and 4-Nitrophenyl chloroformate (2.1 mg, 10 µmol, 3.0 eq.) in DCM (0.5 mL) was added pyridine (0.8 mg, 10 µmol, 3.0 eq.). The mixture was then stirred at room temperature for 16 h. After the solvent was evaporated, the residue was purified via preparative TLC (500 µm, PE/Acetone, 3/1, eluted twice) to yield **S12** as a white powder (6.0 mg, 3 µmol, 91% yield).

^{1}H NMR (500 MHz, Acetone-d_6, 298 K) δ = 8.33 – 8.25 (m, 2H, H_{22}), 7.80 – 7.74 (m, 2H, H_{10}), 7.54 – 7.39 (m, 8H, $H_{11,15,16,21}$), 7.34 – 7.25 (m, 3H, $H_{12,5}$), 7.24 – 7.19 (m, 2H, $H_{10'}$), 7.17 – 7.07 (m, 3H, $H_{11',12'}$), 6.99 – 6.86 (m, 10H, $H_{h,m,o}$), 6.83 – 6.77 (m, 2H, H_4), 6.18 – 6.13 (s, s, 1H, H_7), 5.25 – 5.20 (s, s, 2H, H_{18}), 4.83 – 4.76 (m, 1H, H_d), 4.68 – 4.59 (m, 2H, H_2), 4.43 – 4.36 (m, 1H, H_d), 4.33 – 4.26 (m, 1H, H_e), 4.11 – 4.04 (m, 1H, H_e), 3.96 – 3.89 (m, 2H, H_u), 3.85 – 3.64 (m, 37H, $H_{j,q,k}$), 3.42 – 3.33 (m, 2H, H_{ff}), 1.99 – 1.95 (m, 12H, $H_{a,r}$), 1.19 – 1.15 (m, 2H, H_v), 0.80 – 0.74 (m, 2H, H_z), 0.69 – 0.58 (m, 4H, $H_{aa,y}$), 0.39 – 0.14 (m, 8H, $H_{w,ee,x,bb}$), -0.55 – -0.70 (m, 2H, H_{cc}), -0.87 – -1.04 (m, 2H, H_{dd}).

^{13}C NMR (126 MHz, Acetone-d_6, 298 K) δ = 172.14 (C_c), 171.90 (C_t), 169.22, 169.20 (C_1), 167.76 (C_{13}), 158.99 (C_3), 156.68 (C_{20}), 153.22, 153.20 (C_{19}), 151.57, 151.16, 151.14, 151.05, 151.03 ($C_{i,p}$), 149.96

(C_f), 146.39 (C_{23}), 142.12, 142.11 (C_9), 138.93, 138.92 ($C_{9'}$), 138.75 (C_{14}), 131.49, 131.48 (C_{17}), 130.62, 130.60, 130.18, 129.56, 129.46, 129.21, 128.85, 128.69, 128.62, 128.61, 128.57, 128.55, 128.32, 128.27 ($C_{5,6,10,10',11,11',12,21,g,l,n}$), 127.67 ($C_{12'}$), 126.04 ($C_{22}$), 123.10, 123.09 ($C_{16}$), 118.53 ($C_{15}$), 115.34, 115.32, 114.10, 114.08, 113.74, 113.73, 113.72, 113.69, 113.65, 113.59, 113.56, 113.52, 113.49 ($C_{4,h,m,o}$), 73.17, 73.16 (C_8), 71.04 (C_{18}), 67.13, 67.07 ($C_{e,u}$), 66.85, 66.83 (C_7), 66.49, 66.47 (C_{ff}), 65.99, 65.96 (C_2), 65.48 (C_d), 57.54 (C_s), 57.16 (C_b), 55.83, 55.78, 55.74, 55.71, 55.69, 55.62, 55.60, 55.58, 55.49 ($C_{j,q}$), 31.42 (C_{aa}), 31.30 (C_z), 31.08, 31.07 ($C_{a,r}$), 31.00, 30.94 ($C_{bb,y}$), 30.42 – 29.32 ($C_{k,cc,x}$, overlapped with solvent peak), 29.12 (C_v), 28.71 (C_{ee}), 25.65, 25.64 (C_w), 24.32, 24.27 (C_{dd}).
HRMS-ESI(+): 1927.6128 $[M+Na]^+$, calculated for $C_{103}H_{114}Br_2NO_{23}Na^+$: 1927.6071.

3.2.5 Synthesis of S14

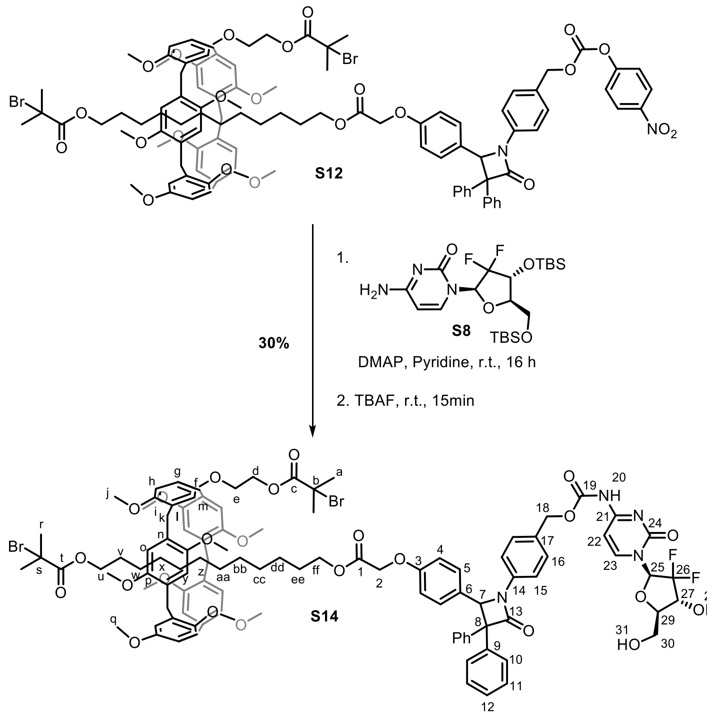

To a solution of **S12** (6.0 mg, 3.2 µmol, 1.0 eq.) and **S8** (15 mg, 31.5 µmol, 10.0 eq.) in dry pyridine (0.5 mL) was added DMAP (0.1 mg, 0.6 µmol, 0.2 eq.). After the mixture stirred at room temperature for 16 h, TBAF solution (189 uL, 1 M in THF, 60.0 eq.) was added and the mixture stirred for a further 15 min. After that, the reaction mixture was washed by $NaHCO_3$ aquous solution (1 x 5 mL) and brine (1 x 5 mL) after adding DCM (5 mL). The organic layer was collected and dried with magnesium sulfate. The mixture was filtered before being concentrated under vacuum. The residue was purified via preparative TLC (500 µm, DCM/MeOH, 20/1, eluted twice) to yield **S14** as a white powder (2.0 mg, 1.0 µmol, 31% yield).

^{1}H NMR (500 MHz, Acetone-d_6, 298 K) δ = 8.25 (d, J = 7.6 Hz, 1H, H_{23}), 7.79 – 7.71 (m, 2H, H_{10}), 7.52 – 7.45 (m, 2H, H_{15}), 7.45 – 7.38 (m, 4H, $H_{11,16}$), 7.33 – 7.24 (m, 3H, $H_{12,5}$), 7.23 – 7.16 (m, 3H, $H_{10',22}$), 7.16 – 7.07 (m, 3H, $H_{11',12'}$), 6.99 – 6.86 (m, 10H, $H_{h,m,o}$), 6.83 – 6.76 (m, 2H, H_4), 6.25 (t, J = 7.4 Hz, 1H, H_{25}), 6.17 – 6.12 (s, s, 1H, H_7), 5.40 (d, J = 6.8 Hz, 1H, H_{28}), 5.17 – 5.09 (s, s, 2H, H_{18}), 4.84 – 4.76 (m, 1H, H_d), 4.68 – 4.59 (m, 2H, H_2), 4.54 – 4.37 (m, 3H, $H_{31,27,d}$), 4.34 – 4.25 (m, 1H, H_e), 4.11 – 4.04 (m, 1H, H_e), 4.04 – 3.97 (m, 2H, $H_{29,30}$), 3.94 – 3.89 (m, 2H, H_u), 3.88 – 3.67 (m, 38H, $H_{j,q,k,30'}$), 3.45 – 3.38 (m, 2H, H_{ff}), 1.98 – 1.96 (m, 12H, $H_{a,r}$), 1.19 – 1.11 (m, 2H, H_v), 0.81 – 0.70 (m, 2H, H_z), 0.68 – 0.54 (m, 4H, $H_{aa,y}$), 0.32 – 0.23 (m, 4H, $H_{w,ee}$), 0.23 – 0.15 (m, 4H, $H_{x,bb}$), -0.51 – -0.64 (m, 2H, H_{cc}), -0.81 – -0.94 (m, 2H, H_{dd}).

^{13}C NMR (151 MHz, Acetone-d_6, 298 K) δ = 172.15 (C_c), 171.91 (C_t), 169.22, 169.20 (C_1), 167.67 (C_{13}), 164.18 (C_{21}), 158.97 (C_3), 154.98 (C_{24}), 153.74 (C_{19}), 151.57, 151.14, 151.04 ($C_{i,p}$), 149.97 (C_f), 145.27 (C_{23}), 142.16 (C_9), 138.95 ($C_{9'}$), 138.35 (C_{14}), 132.47 (C_{17}), 130.18, 130.12, 130.09, 130.07, 130.04, 129.54, 129.46, 129.22, 128.89, 128.85, 128.69, 128.61, 128.57, 128.56, 128.31, 128.24 ($C_{5,6,10,10',11,11',12,16,g,l,n}$), 127.65 ($C_{12'}$), 123.88 (t, J = 338.9 Hz, C_{26}), 118.47 (C_{15}), 115.35, 115.30, 114.10, 114.09, 113.74, 113.72, 113.70, 113.66, 113.61, 113.56, 113.53, 113.49 ($C_{4,h,m,o}$), 95.35 (C_{22}), 86.46 (C_{25}), 82.30 (C_{29}), 73.09 (C_8), 69.86 (C_{27}), 67.64 (C_{18}), 67.14, 67.10 ($C_{e,u}$), 66.84, 66.82 (C_7), 66.46 (C_{ff}), 65.99, 65.96 (C_2), 65.48 (C_d), 60.09 (C_{30}), 59.97 ($C_{30'}$), 57.54 (C_s), 57.16 (C_b), 55.83, 55.78, 55.74, 55.69, 55.62, 55.59 ($C_{j,q}$), 31.43 (C_{aa}), 31.29 (C_z), 31.07 ($C_{a,r}$), 31.01, 30.94 ($C_{bb,y}$), 30.16 – 29.49 ($C_{k,cc,x}$, overlapped with solvent peak), 29.12 (C_v), 28.76 (C_{ee}), 25.59 (C_w), 24.39, 24.35 (C_{dd}).
^{19}F NMR (471 MHz, Acetone-d_6, 298 K) δ = -118.76 (2F, F_{26}).
HRMS-ESI(+): 2051.6558 $[M+Na]^+$, calculated for $C_{106}H_{120}Br_2F_2N_4O_{24}Na^+$: 2051.6520.

3.2.6 Synthetic Routes to S17

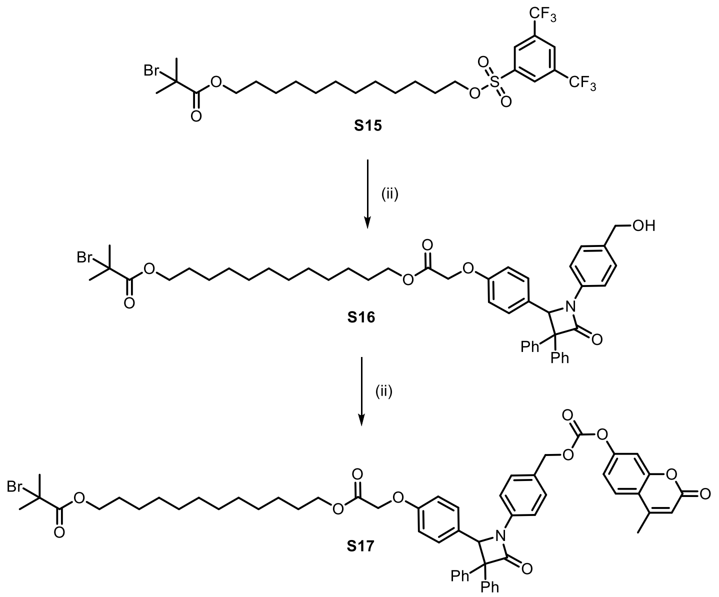

Scheme S3. Synthetic routes to **S17**. Conditions: (i) **S6**, K_2CO_3, 18-crown-6, Acetone, r.t., 16 h, 46% yield; (ii) **S7**, Pyridine, DCM, r.t., 16 h, 60% yield.

3.2.7 Synthesis of S16

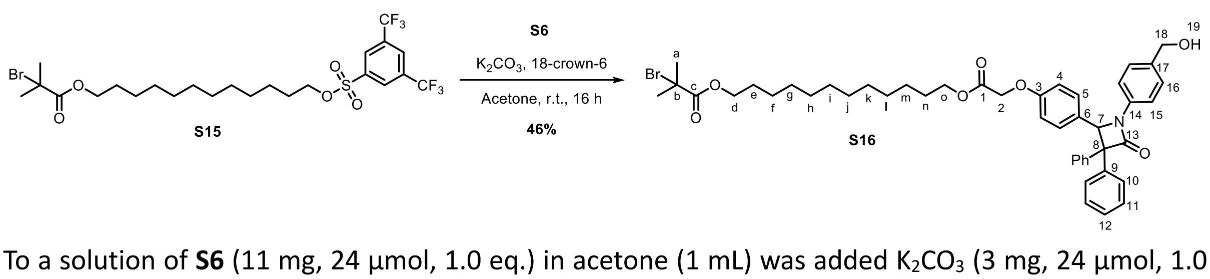

To a solution of **S6** (11 mg, 24 µmol, 1.0 eq.) in acetone (1 mL) was added K_2CO_3 (3 mg, 24 µmol, 1.0 eq.) and 18-crown-6 (6 mg, 24 µmol, 1.0 eq.). The mixture was stirred for 2 h at room temperature. **S15** (15 mg, 24 µmol, 1.0 eq.) was added and the mixture stirred for a further 16 h at room temperature. The solution was filtered, and the filtrate concentrated under vacuum. The residue was purified by preparative TLC (500 µm, PE/Acetone, 4/1, eluted twice) to yield **S16** as a white powder (9 mg, 11 µmol, 46% yield).

^{1}H NMR (500 MHz, Acetone-d_6, 298 K) δ = 7.77 – 7.72 (m, 2H, H_{10}), 7.43 – 7.37 (m, 4H, $H_{11,15}$), 7.32 – 7.25 (m, 3H, $H_{12,16}$), 7.22 – 7.15 (m, 4H, $H_{5,10'}$), 7.10 – 7.01 (m, 3H, $H_{11',12'}$), 6.76 – 6.71 (m, 2H, H_4), 6.06 (s, 1H, H_7), 4.62 (s, 2H, H_2), 4.54 (d, J = 6.3 Hz, 2H, H_{18}), 4.18 – 4.10 (m, 4H, $H_{d,o,19}$), 1.92 (s, 6H, H_a), 1.71 – 1.64 (m, 2H, H_e), 1.64 – 1.58 (m, 2H, H_n), 1.45 – 1.38 (m, 2H, H_f), 1.37 – 1.28 (m, 14H, $H_{g\text{-}m}$).
^{13}C NMR (126 MHz, Acetone-d_6, 298 K) δ = 171.84 (C_c), 169.23 (C_1), 167.45 (C_{13}), 158.89 (C_3), 142.28 (C_9), 139.24, 139.22 (C_{17}), 139.03 ($C_{9'}$), 137.17 (C_{14}), 130.01 (C_5), 129.50 (C_{11}), 129.18 ($C_{10'}$), 128.89 (C_6), 128.75 ($C_{11'}$), 128.30 (C_{10}), 128.17 ($C_{15,12}$), 127.52 ($C_{12'}$), 118.20 (C_{16}), 115.23 (C_4), 72.91 (C_8), 66.81 (C_7), 66.51 (C_d), 65.74 (C_2), 65.49 (C_o), 64.24 (C_{18}), 64.11 ($C_{18'}$), 57.52 (C_b), 30.98 (C_a), 30.46 – 29.24 ($C_{g\text{-}l,n}$, overlapped with solvent peak), 29.10 (C_e), 26.52, 26.51 ($C_{f,m}$).
HRMS-ESI(+): 834.2959 $[M+Na]^+$, calculated for $C_{46}H_{54}BrNO_7Na^+$: 834.2962.

3.2.8 Synthesis of S17

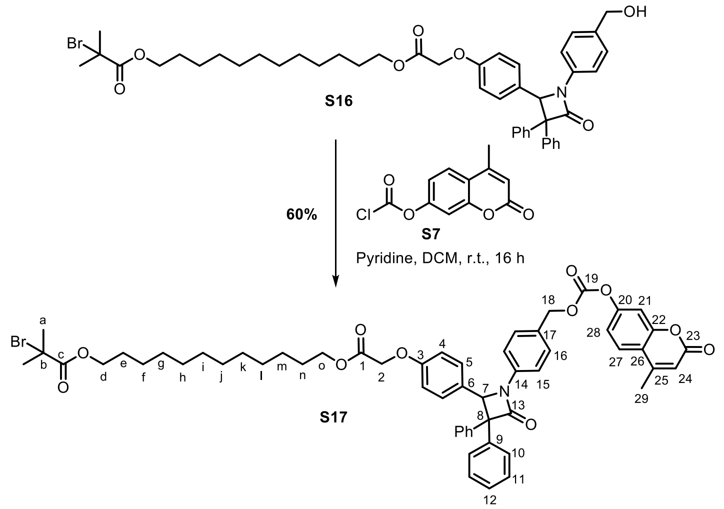

To a solution of **S16** (4.0 mg, 5 µmol, 1.0 eq.) and **S7** (2.3 mg, 10 µmol, 2.0 eq.) in DCM (0.5 mL) was added pyridine (0.8 mg, 10 µmol, 2.0 eq.). The mixture was then stirred at room temperature for 16 h. After the solvent was evaporated, the residue was purified by preparative TLC (500 µm, PE/DCM/EtOAc, 4/4/0.5, eluted twice) to yield **S17** as a white powder (3 mg, 3 µmol, 60% yield).

^{1}H NMR (500 MHz, Acetone-d_6, 298 K) δ = 7.82 (d, J = 8.7 Hz, 1H, H_{27}), 7.78 – 7.73 (m, 2H, H_{10}), 7.51 – 7.46 (m, 2H, H_{15}), 7.46 – 7.39 (m, 4H, $H_{16,11}$), 7.33 – 7.28 (m, 1H, H_{12}), 7.26 – 7.15 (m, 6H, $H_{21,28,5,10'}$), 7.12 – 7.02 (m, 3H, $H_{11',12'}$), 6.78 – 6.72 (m, 2H, H_4), 6.31 (q, J = 1.2 Hz, 1H, H_{24}), 6.11 (s, 1H, H_7), 5.25 (s, 2H, H_{18}), 4.63 (s, 2H, H_2), 4.18 – 4.09 (m, 4H, $H_{d,o}$), 2.49 (d, J = 1.2 Hz, 3H, H_{29}), 1.92 (s, 6H, H_a), 1.71 – 1.65 (m, 2H, H_e), 1.62 – 1.58 (m, 2H, H_n), 1.43 – 1.39 (m, 2H, H_f), 1.37 – 1.30 (m, 14H, $H_{g\text{-}m}$).
^{13}C NMR (126 MHz, Acetone-d_6, 298 K) δ = 171.83 (C_c), 169.22 (C_1), 167.76 (C_{13}), 160.23 (C_{23}), 158.96 (C_3), 155.07 (C_{22}), 154.35 (C_{20}), 153.56 (C_{19}), 153.20 (C_{25}), 142.12 (C_9), 138.90 ($C_{9'}$), 138.75 (C_{14}), 131.59 (C_{17}), 130.55 (C_{16}), 130.02 (C_5), 129.54 (C_{11}), 129.15 ($C_{10'}$), 128.79 ($C_{11'}$), 128.65 (C_6), 128.30 (C_{10}), 128.24 (C_{12}), 127.59 ($C_{12'}$), 127.11 (C_{27}), 118.87 (C_{26}), 118.47 (C_{15}), 118.23 (C_{28}), 115.30 (C_4), 115.08 (C_{24}), 110.35 (C_{21}), 73.12 (C_8), 70.87 (C_{18}), 66.89 (C_7), 66.51 (C_d), 65.74 (C_2), 65.49 (C_o), 57.51 (C_b), 30.98 (C_a), 30.50 – 29.27 ($C_{g\text{-}l,n}$, overlapped with solvent peak), 29.09 (C_e), 26.51 ($C_{f,m}$), 18.58 (C_{29}).
HRMS-ESI(+): 1036.3240 $[M+Na]^+$, calculated for $C_{57}H_{60}BrNO_{11}Na^+$: 1036.3242.

3.3 Synthesis of Reference Compounds

3.3.1 Synthesis of 7

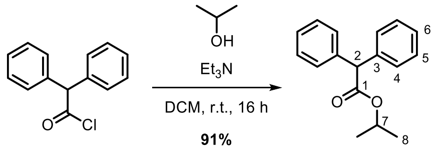

7

To a solution of isopropanol (13 mg, 217 µmol, 1.0 eq.) and diphenylacetyl chloride (50 mg, 217 µmol, 1.0 eq.) in DCM (1 mL) was added Et_3N (44 mg, 435 µmol, 2.0 eq.). The mixture was then stirred at room temperature for 16 h. After the solvent was evaporated, the residue was purified via preparative TLC (500 µm, PE/DCM, 4/1, eluted three times) to yield **7** as a white powder (50 mg, 197 µmol, 91% yield).

^{1}H NMR (400 MHz, Acetone-d_6, 298 K) δ = 7.39 – 7.29 (m, 8H, $H_{4,5}$), 7.29 – 7.23 (m, 2H, H_6), 5.09 – 4.98 (m, 2H, $H_{2,7}$), 1.20 (d, J = 6.2 Hz, 6H, H_8).
^{13}C NMR (101 MHz, Acetone-d_6, 298 K) δ = 172.27 (C_1), 140.34 (C_3), 129.43, 129.28 ($C_{4,5}$), 127.86 (C_6), 68.95 (C_7), 57.72 (C_2), 21.85 (C_8).
HRMS-APCI(+): 277.1192 $[M+Na]^+$, calculated for $C_{17}H_{18}O_2Na^+$: 277.1186.

4 Synthesis of Polymers

4.1 Representative Procedure for SET-LRP of Methyl Acrylate Using Mechanophore Initiators

Methyl acrylate was filtered through basic alumina to remove the inhibitor prior to use. A stock catalytic solution of Me_6TREN (16 µL, 0.060 mmol) and $CuBr_2$ (5.6 mg, 0.025 mmol) in dry DMSO (1 mL) was prepared. To a 5 mL microwave vial was added the appropriate initiator compound along with catalytic solution, methyl acrylate and dry DMSO. This solution was degassed by bubbling with N_2 for 10 min. A Cu(0) wire wrapped around a stirrer bar, having been cleaned in 12 N HCl for 10 min, was added to the reaction mixture. The reaction mixture was degassed for a further 2 min before being allowed to stir for 15 - 40 min (until the extent of polymerization, as determined approximately by the increasing viscosity of the solution, was deemed acceptable). The solution was added dropwise to a solution of vigorously stirred methanol; the precipitated polymer was recovered and dried under vacuum for two days to yield a white material. Molecular weight and polydispersity indices were determined using an analytical SEC that had been calibrated with polystyrene standards.

4.2 Synthesis of Mechanophore and Control Polymers

4.2.1 Synthesis of Polymer 5_{-138} and 5_{-155}

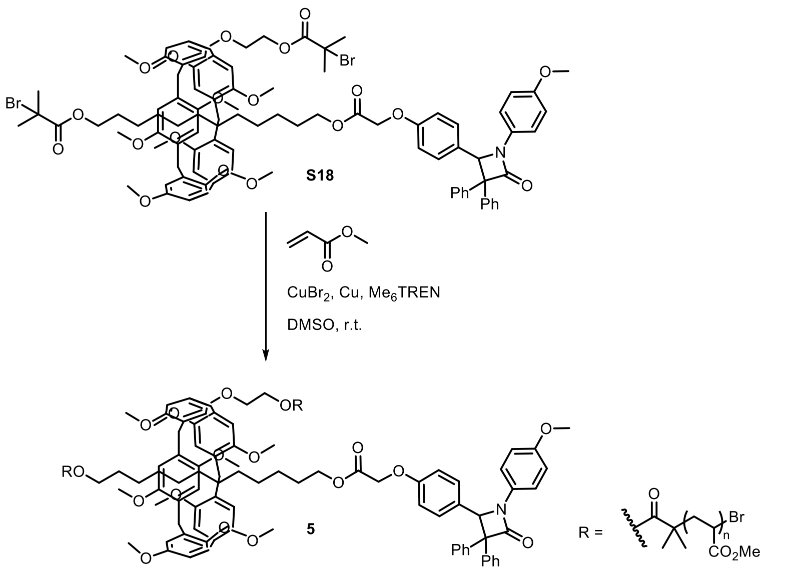

Synthesis followed the representative procedure. **S18** (3.5 mg, 2.0 µmol, 1.0 eq.), 16 µL of catalytic solution ($CuBr_2$: 0.4 µmol, 0.2 eq.; Me_6TREN: 1.0 µmol, 0.5 eq.), methyl acrylate (359 µL, 4.0 mmol, 2000.0 eq.), Cu (0) wire (~3 cm, ~30 mg, 0.5 mmol, ~240.0 eq.) and dry DMSO (359 µL) were used in the reaction to yield polymer **5**$_{-138}$ (108 mg, M_n = 138 kDa; $Đ$ = 1.18) or **5**$_{-155}$ (135 mg, M_n = 155 kDa; $Đ$ = 1.13) in two batches.

4.2.2 Synthesis of Polymer 1_{HYM}

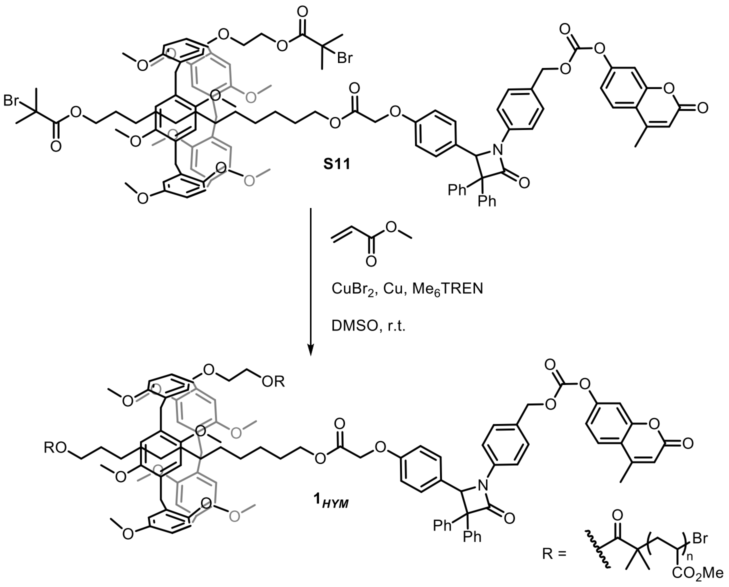

Synthesis followed the representative procedure. **S11** (2.0 mg, 1.0 µmol, 1.0 eq.), 8 µL of catalytic solution ($CuBr_2$: 0.2 µmol, 0.2 eq.; Me_6TREN: 0.5 µmol, 0.5 eq.), methyl acrylate (184 µL, 2.1 mmol, 2000.0 eq.), Cu (0) wire (~3 cm, ~30 mg, 0.5 mmol, ~500.0 eq.) and dry DMSO (184 µL) were used in the reaction to yield polymer $\mathbf{1}_{HYM}$ (85 mg, M_n = 159 kDa; $Đ$ = 1.32).

4.2.3 Synthesis of Polymer 1_{GEM}

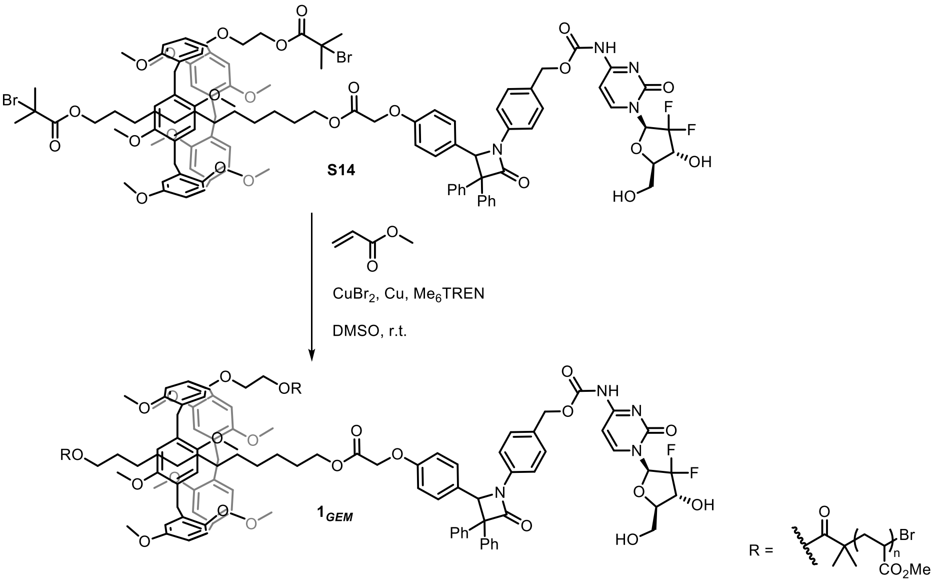

Synthesis followed the representative procedure. **S14** (1.0 mg, 0.5 µmol, 1.0 eq.), 4 µL of catalytic solution ($CuBr_2$: 0.1 µmol, 0.2 eq.; Me_6TREN: 0.2 µmol, 0.5 eq.), methyl acrylate (88 µL, 1.0 mmol, 2000.0 eq.), Cu (0) wire (~3 cm, ~30 mg, 0.5 mmol, ~1000.0 eq.) and dry DMSO (88 µL) were used in the reaction to yield polymer $\mathbf{1}_{GEM}$ (42 mg, M_n = 147 kDa; $Đ$ = 1.30).

4.2.4 Synthesis of Polymer S20

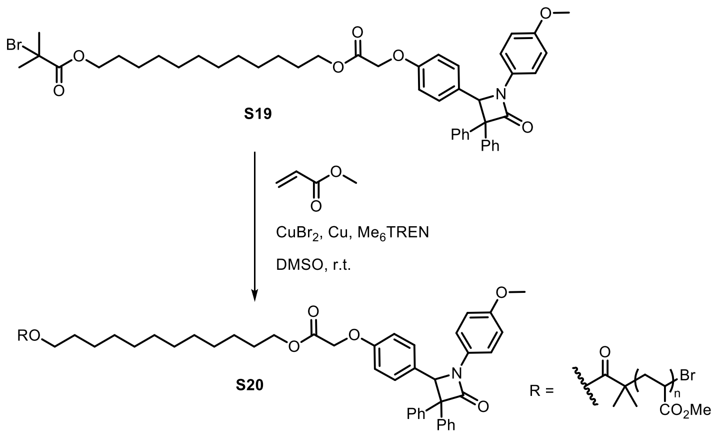

Synthesis followed the representative procedure. **S19** (2.0 mg, 2.5 µmol, 1.0 eq.), 20 µL of catalytic solution ($CuBr_2$: 0.5 µmol, 0.2 eq.; Me_6TREN: 1.2 µmol, 0.5 eq.), methyl acrylate (440 µL, 4.9 mmol, 2000.0 eq.), Cu (0) wire (~3 cm, ~30 mg, 0.5 mmol, ~200.0 eq.) and dry DMSO (440 µL) were used in the reaction to yield polymer **S20** (138 mg, M_n = 128 kDa; $Đ$ = 1.37).

4.2.5 Synthesis of Polymer S21

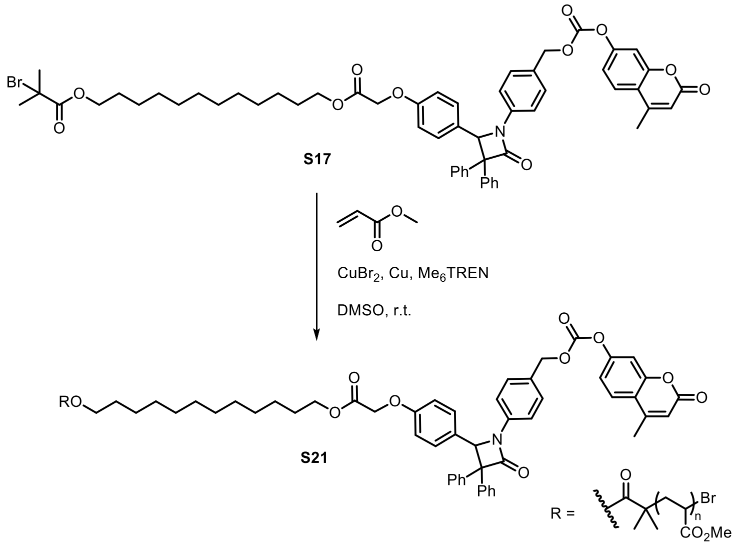

Synthesis followed the representative procedure. **S17** (1.0 mg, 1.0 µmol, 1.0 eq.), 8 µL of catalytic solution ($CuBr_2$: 0.2 µmol, 0.2 eq.; Me_6TREN: 0.5 µmol, 0.5 eq.), methyl acrylate (176 µL, 2.0 mmol, 2000.0 eq.), Cu (0) wire (~3 cm, ~30 mg, 0.5 mmol, ~500.0 eq.) and dry DMSO (176 µL) were used in the reaction to yield polymer **S21** (64 mg, M_n = 125 kDa; $Đ$ = 1.12).

4.2.6 Synthesis of Polymer 9_{-112} and 9_{-114}

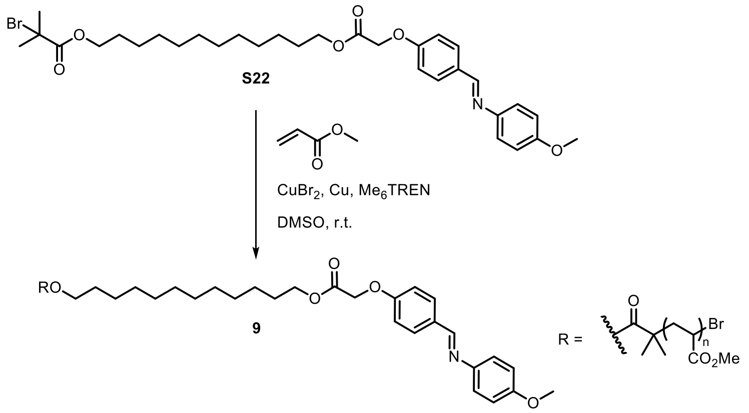

Synthesis followed the representative procedure. **S22** (4.0 mg, 6.5 µmol, 1.0 eq.), 52 µL of catalytic solution ($CuBr_2$: 1.3 µmol, 0.2 eq.; Me_6TREN: 3.1 µmol, 0.5 eq.), methyl acrylate (1156 µL, 13.0 mmol, 2000.0 eq.), Cu (0) wire (~3 cm, ~30 mg, 0.5 mmol, ~80.0 eq.) and dry DMSO (1156 µL) were used in the reaction to yield polymer **9**$_{-112}$ (335 mg, M_n = 112 kDa; *Đ* = 1.40) or **9**$_{-114}$ (325 mg, M_n = 114 kDa; *Đ* = 1.42) in two batches.

4.3 Synthesis of Reference Polymers

4.3.1 Synthesis of Polymer 10

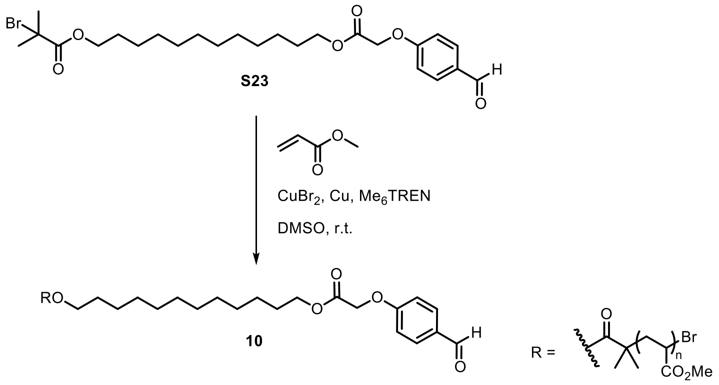

Synthesis followed the representative procedure. **S23** (1.0 mg, 2.0 µmol, 1.0 eq.), 16 µL of catalytic solution ($CuBr_2$: 0.4 µmol, 0.2 eq.; Me_6TREN: 0.9 µmol, 0.5 eq.), methyl acrylate (348 µL, 3.9 mmol, 2000.0 eq.), Cu (0) wire (~3 cm, ~30 mg, 0.5 mmol, ~250.0 eq.) and dry DMSO (348 µL) were used in the reaction to yield polymer **10** (90 mg, M_n = 98 kDa; *Đ* = 1.13).

4.4 SEC Data for Synthesised Polymers

Table S1. M_n and $Đ$ values for all synthesised polymers.

**Polymer**	***M*_n / kDa**	***Đ***
$\mathbf{5}_{\text{-138}}$	138	1.18
$\mathbf{5}_{\text{-155}}$	155	1.13
$\mathbf{1}_{HYM}$	159	1.32
$\mathbf{1}_{GEM}$	147	1.30
**S20**	128	1.37
**S21**	125	1.12
$\mathbf{9}_{\text{-112}}$	112	1.40
$\mathbf{9}_{\text{-114}}$	114	1.42
**10**	98	1.13

4.5 SEC Traces for Mechanophore and Control Polymers

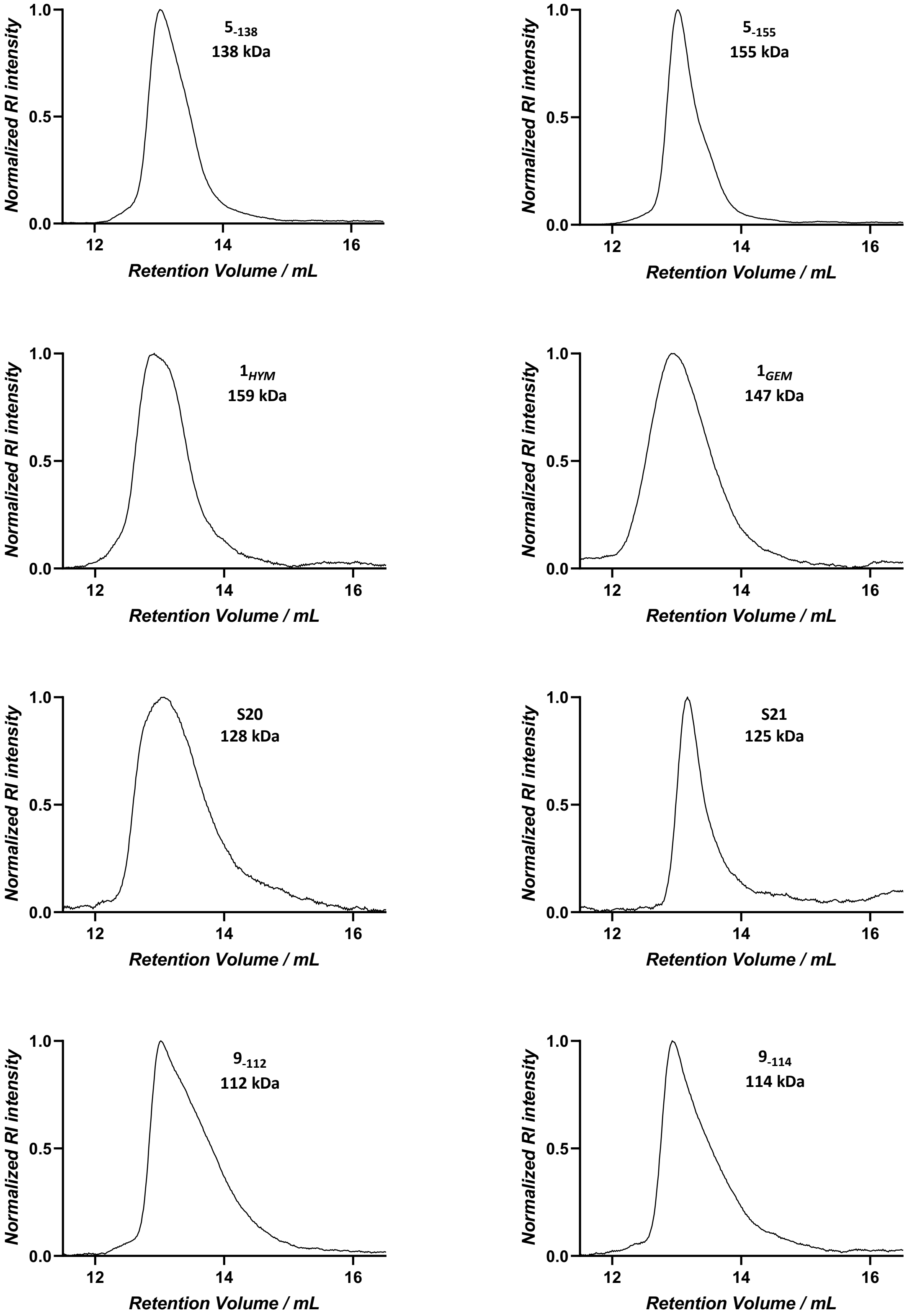

Figure S1. SEC traces for polymers $\mathbf{5}_{-138}$, $\mathbf{5}_{-155}$, $\mathbf{1}_{HYM}$, $\mathbf{1}_{GEM}$, **S20**, **S21**, $\mathbf{9}_{-112}$ and $\mathbf{9}_{-114}$.

4.6 SEC Traces for Reference Polymers

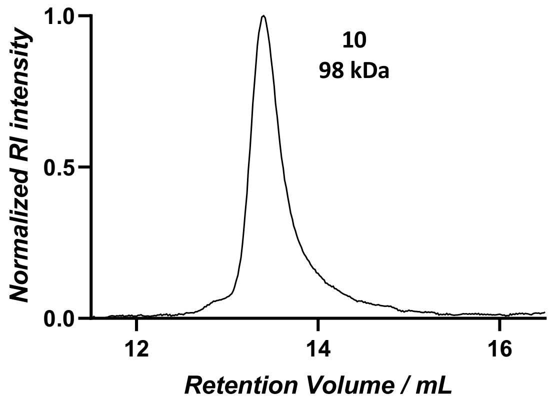

Figure S2. SEC trace of reference polymer **10**.

5 Mechanophore Activation via Ultrasound

5.1 General Procedure for Sonication Experiments

The appropriate polymer (10 mg) was added to a 5 mL microwave vial and dissolved in the appropriate solvent (3 mL). The solution was degassed by bubbling N_2 through it for a minimum of 2 min prior to the start of sonication. The microwave vial was cooled with an ice bath throughout the duration of the sonication to maintain a temperature of ~ 5-10 °C inside the cell. Pulsed ultrasound was applied to the system (1 s ON / 1 s OFF, 25% amplitude (13.5 W cm^{-2}), 20 kHz) for 105 min. After sonication, the solution was analysed by NMR spectroscopy directly after incubation for 16 h as post-sonication polymer before being washed with MeOH. Then the solvent was evaporated, and the polymer was analysed by SEC. The post-sonication polymer was recovered and washed with MeOH to extract any small molecules not attached to polymer chains. The remaining MeOH-washed polymer and the concentrated MeOH washings were then analysed by NMR spectroscopy.

5.2 Sonication of Mechanophore Polymer 5_{-138}

Sonication of mechanophore polymer $\mathbf{5_{-138}}$, using the methodology described in the general procedure (*Section 5.1*) and with the solvent used being CD_3CN/H_2O (9/1), was carried out twice to determine the extent of activation by retro-[2+2] cycloaddition of the β-lactam mechanophore and the hydrolysis of the imine polymer to release the amine. SEC analysis of the sonicated polymers showed complete cleavage (M_n of the post-sonication material was less than half of that of the pre-sonication polymer).

Comparison of the ^{1}H NMR spectra of polymer $\mathbf{5_{-138}}$ before and after sonication showed that the retro-[2+2] cycloaddition of the β-lactam mechanophore occurred. It is evidenced by presence of diphenylacetic acid (*n-q*) signals in both the crude reaction mixture and the MeOH extracts. The formation of the aldehyde was confirmed by the emergence diagnostic peaks (*j-m*) in the crude mixture. Anisidine itself can be distinguished (*r*, *s*) in the crude mixture but is lost during MeOH extaction. No imine polymer was observed in the crude mixture, confirming its complete hydrolysis.

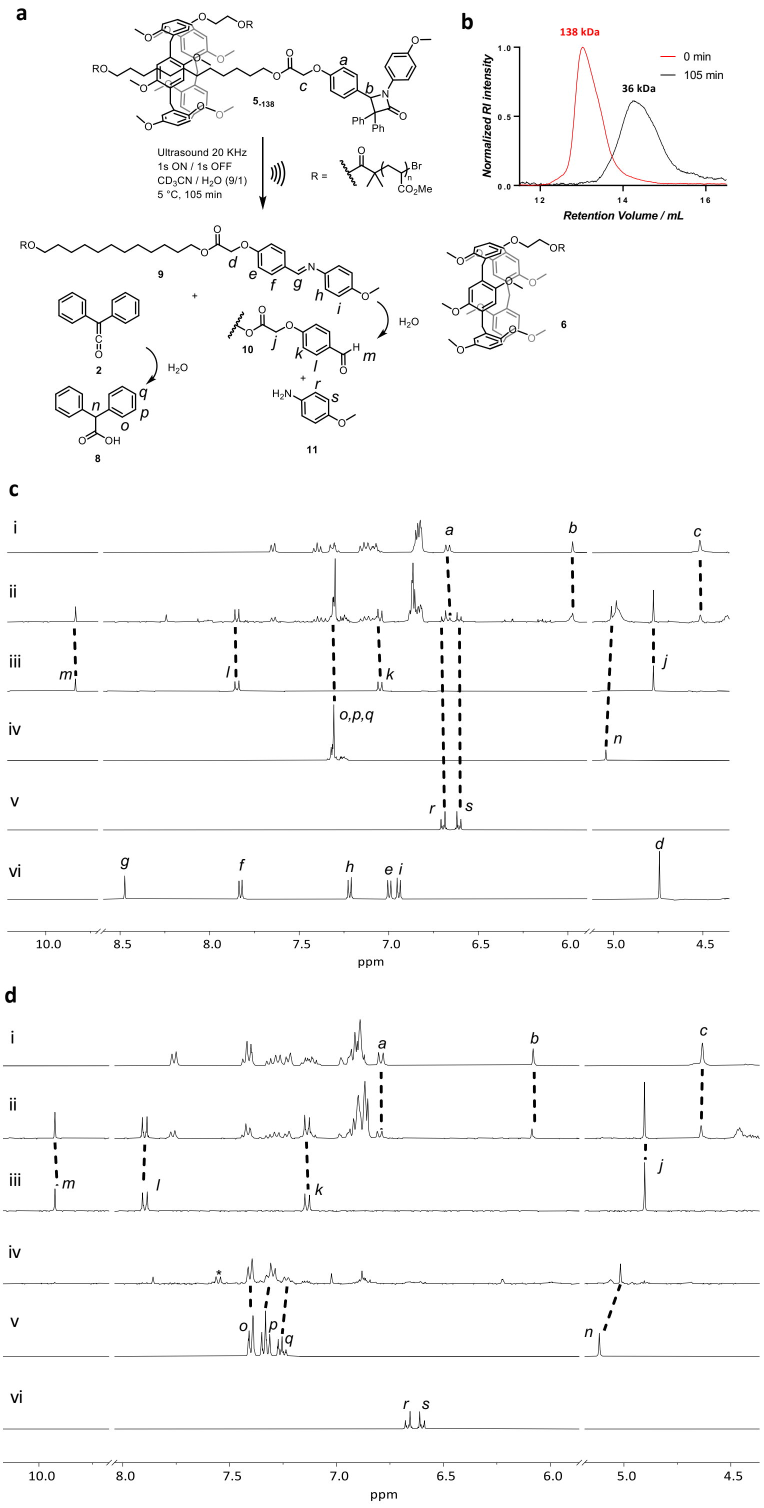

Figure S3. Sonication (run 1) of polymer **5**$_{\mathbf{-138}}$ in CD_3CN/H_2O (9/1). Sonication of polymer **5**$_{\mathbf{-138}}$ affords fragments **10** and **11**, upon hydrolysis of **9**$_{\mathbf{-114}}$, **8**, upon hydrolysis of **2**, and **6** (a). SEC traces of polymer **5**$_{\mathbf{-138}}$ (b) before (red) and after (black) sonication. Partial ^1H NMR (500 MHz, Acetonitrile-*d*$_3$/H_2O (9/1), 298 K) spectra comparison of (c) polymer **5**$_{\mathbf{-138}}$ before (i) and after sonication (ii), reference polymer **10** (iii), reference compound **8** (iv), reference compound **11** (v), and reference polymer **9**$_{\mathbf{-114}}$ (vi). Partial ^1H NMR (500 MHz, Acetone-*d*$_6$, 298 K) spectra comparison of (d) polymer **5**$_{\mathbf{-138}}$ before sonication (i), and after sonication and MeOH wash (ii), reference polymer **10** (iii), concentrated methanol washings (iv), reference compound **8** (v), and reference compound **11** (vi). * Peaks marked with star are external impurities. Details see *Section 5.8*.

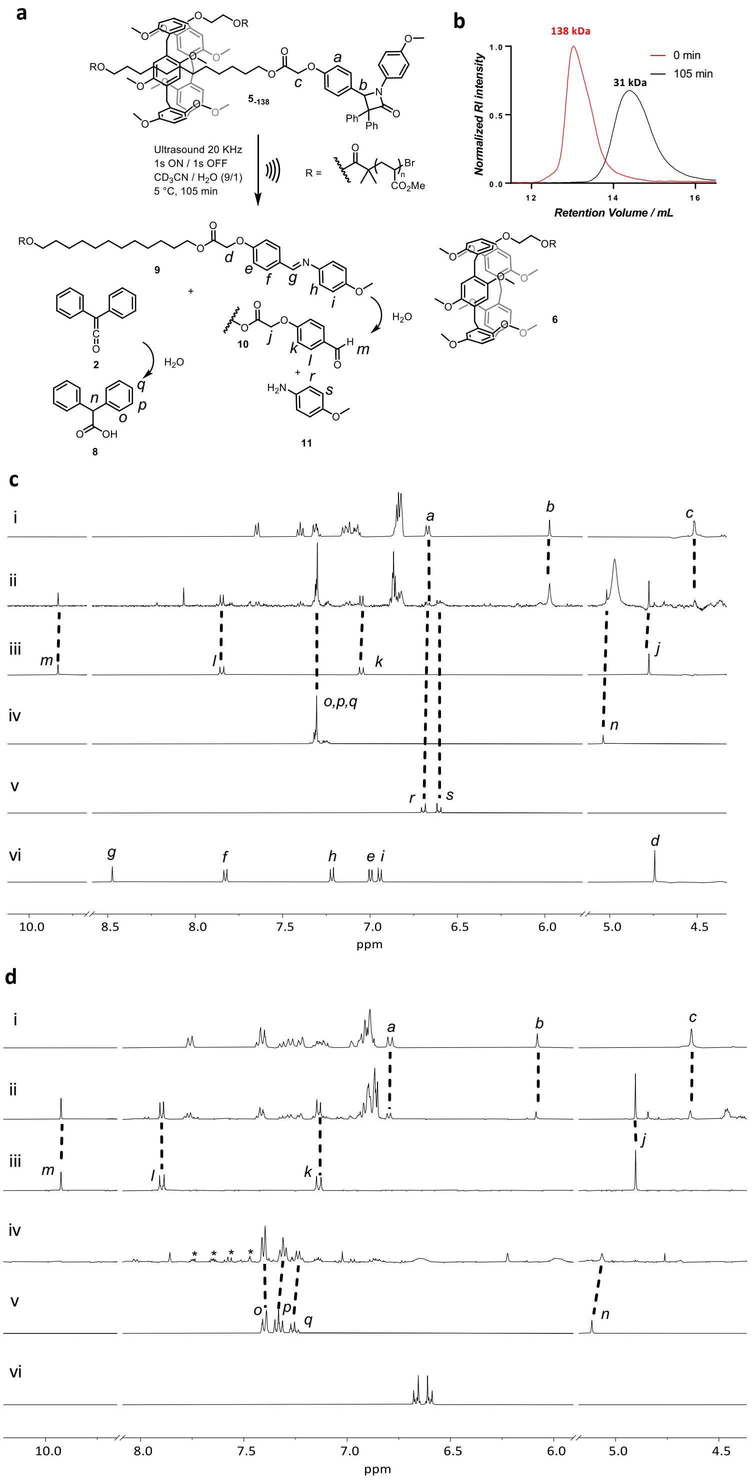

Figure S4. Sonication (run 2) of polymer $\mathbf{5}_{\text{-}\mathbf{138}}$ in CD_3CN/H_2O (9/1). Sonication of polymer $\mathbf{5}_{\text{-}\mathbf{138}}$ affords fragments **10** and **11**, upon hydrolysis of $\mathbf{9}_{\text{-}\mathbf{114}}$, **8**, upon hydrolysis of **2**, and **6** (a). SEC traces of polymer $\mathbf{5}_{\text{-}\mathbf{138}}$ (b) before (red) and after (black) sonication. Partial ^{1}H NMR (500 MHz, Acetonitrile-d_3/H_2O (9/1), 298 K) spectra comparison of (c) polymer $\mathbf{5}_{\text{-}\mathbf{138}}$ before (i), and after sonication (ii), reference polymer **10** (iii), reference compound **8** (iv), reference compound **11** (v), and reference polymer $\mathbf{9}_{\text{-}\mathbf{114}}$ (vi). Partial ^{1}H NMR (500 MHz, Acetone-d_6, 298 K) spectra comparison of (d) polymer $\mathbf{5}_{\text{-}\mathbf{138}}$ before sonication (i), after sonication and MeOH wash (ii), reference polymer **10** (iii), concentrated methanol washings (iv), reference compound **8** (v), and reference compound **11** (vi). * Peaks marked with star are external impurities. Details see *Section 5.8*.

5.3 Sonication of Mechanophore Polymer 5_{-155} at the Absence of Water

Additionally, the sonication of mechanophore polymer **5**$_{-155}$, using the methodology described in the general procedure (*Section 5.1*) and with the solvent used being CD_3CN/*i*PrOH (9/1), was carried out twice to see the stability of imine polymer generated from the retro-[2+2] cycloaddition of the β-lactam mechanophore. The analysis of the post-sonication spectra of polymer **5**$_{-155}$ before and after being washed with methanol successfully revealed that no hydrolysis of imine polymer occured in the absence of water after activation of the β-lactam mechanophore. In this case, the generated diphenylketene was trapped with *i*PrOH to form ester **7**.

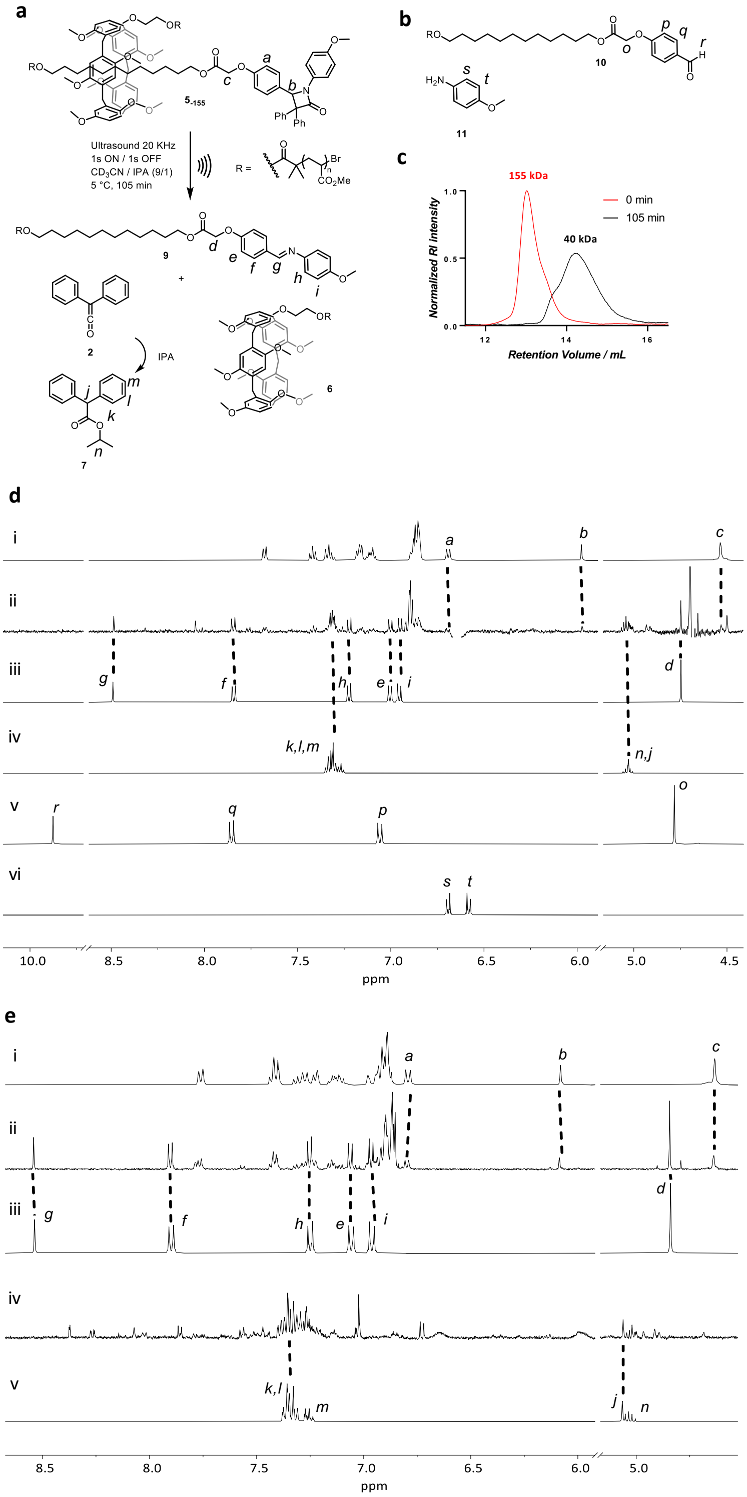

Figure S5. Sonication (run 1) of polymer **5**$_{\text{-}155}$ in CD_3CN/*i*PrOH (9/1). Sonication of polymer **5**$_{\text{-}155}$ affords fragments **9**$_{\text{-}114}$, **7** after **2** trapping *i*PrOH, and **6** (a). Reference species **10** and **11** (b). SEC traces of polymer **5**$_{\text{-}155}$ (c) before (red) and after (black) sonication. Partial ^{1}H NMR (500 MHz, Acetonitrile-*d*$_6$ (or CD_3CN/*i*PrOH (9/1) where indicated), 298 K) spectra comparison of (d) polymer **5**$_{\text{-}155}$ before (i) and after sonication (ii), reference polymer **9**$_{\text{-}114}$ (iii), reference compound **7** (iv), reference compound **10** (v), and reference polymer **11** (vi). Partial ^{1}H NMR (500 MHz, Acetone-*d*$_6$, 298 K) spectra comparison of (e) polymer **5**$_{\text{-}155}$ before sonication (i), and after sonication and MeOH wash (ii), reference polymer **9**$_{\text{-}114}$ (iii), concentrated methanol washings (iv), and reference compound **7** (v).

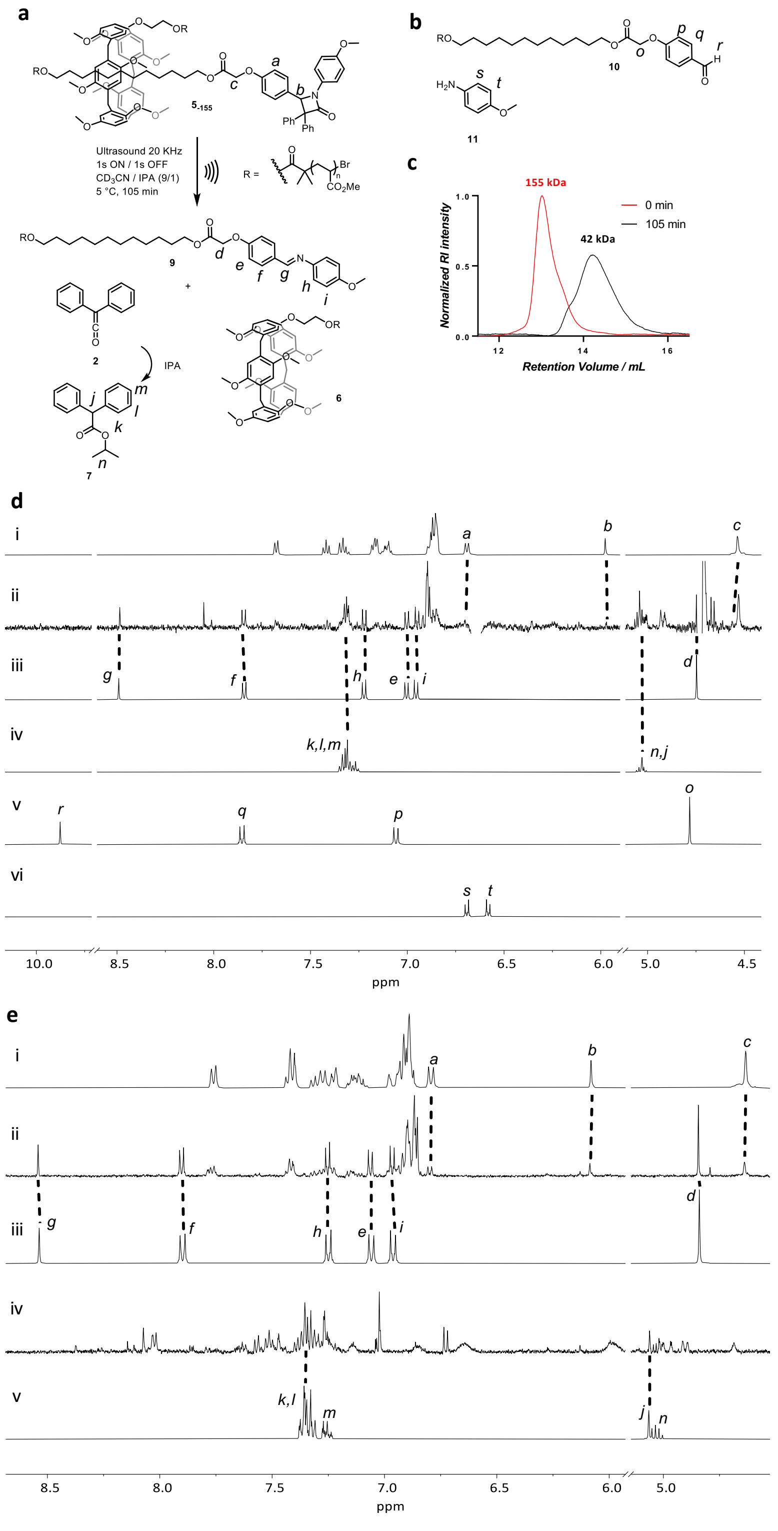

Figure S6. Sonication (run 2) of polymer **5**$_{\text{-155}}$ in CD_3CN/*i*PrOH (9/1). Sonication of polymer **5**$_{\text{-155}}$ affords fragments **9**$_{\text{-114}}$, **7** after **2** trapping *i*PrOH, and **6** (a). Reference species **10** and **11** (b). SEC traces of polymer **5**$_{\text{-155}}$ (c) before (red) and after (black) sonication. Partial ^{1}H NMR (500 MHz, Acetonitrile-*d*$_6$ (or CD_3CN/*i*PrOH (9/1) where indicated), 298 K) spectra comparison of (d) polymer **5**$_{\text{-155}}$ before (i) and after sonication (ii), reference polymer **9**$_{\text{-114}}$ (iii), reference compound **7** (iv), reference compound **10** (v), and reference polymer **11** (vi). Partial ^{1}H NMR (500 MHz, Acetone-*d*$_6$, 298 K) spectra comparison of (e) polymer **5**$_{\text{-155}}$ before sonication (i), and after sonication and MeOH wash (ii), reference polymer **9**$_{\text{-114}}$ (iii), concentrated methanol washings (iv), and reference compound **7** (v).

5.4 Sonication of Mechanophore Polymer $\mathbf{1}_{HYM}$

Sonication of mechanophore polymer $\mathbf{1}_{HYM}$, containing hymecromone (drug/fluorescent indicator) attached to the β-lactam core via a self-immolative linker, using the methodology described in the general procedure (*Section 5.1*) and with CD_3CN/H_2O (9/1), was carried out twice to determine the extent of activation of mechanophore activation and cargo release. SEC analysis of the sonicated polymers showed complete cleavage (M_n of the post-sonication material was less than half of that of the pre-sonication polymer).

Comparison of the ^{1}H NMR spectra of polymer $\mathbf{1}_{HYM}$ before and after sonication showed that retro-[2+2] cycloaddition of β-lactam mechanophore occurred upon pushing activation by the rotaxane actuator, which triggered the cascade reaction leading to the release of hymecromone. It is evidenced by the emergence of signals pertaining to an aromatic aldehyde (*e-g*), diphenylacetic acid (*n-q*), and hymecromone (*k-q*), while signals of the intial β-lactam mechanophore decreased in intensity. Peaks (*e-g*) were confirmed to be aldehyde polymer after comparing with reference **10**. Diphenylacetic acid and hymecromone were identified in the crude mixture and later in the MeOH extracts (the 4-aminobenzyl alcohol linker could not be indentified). The fluorescence of hymecromone is revealed upon release (Figure S9).

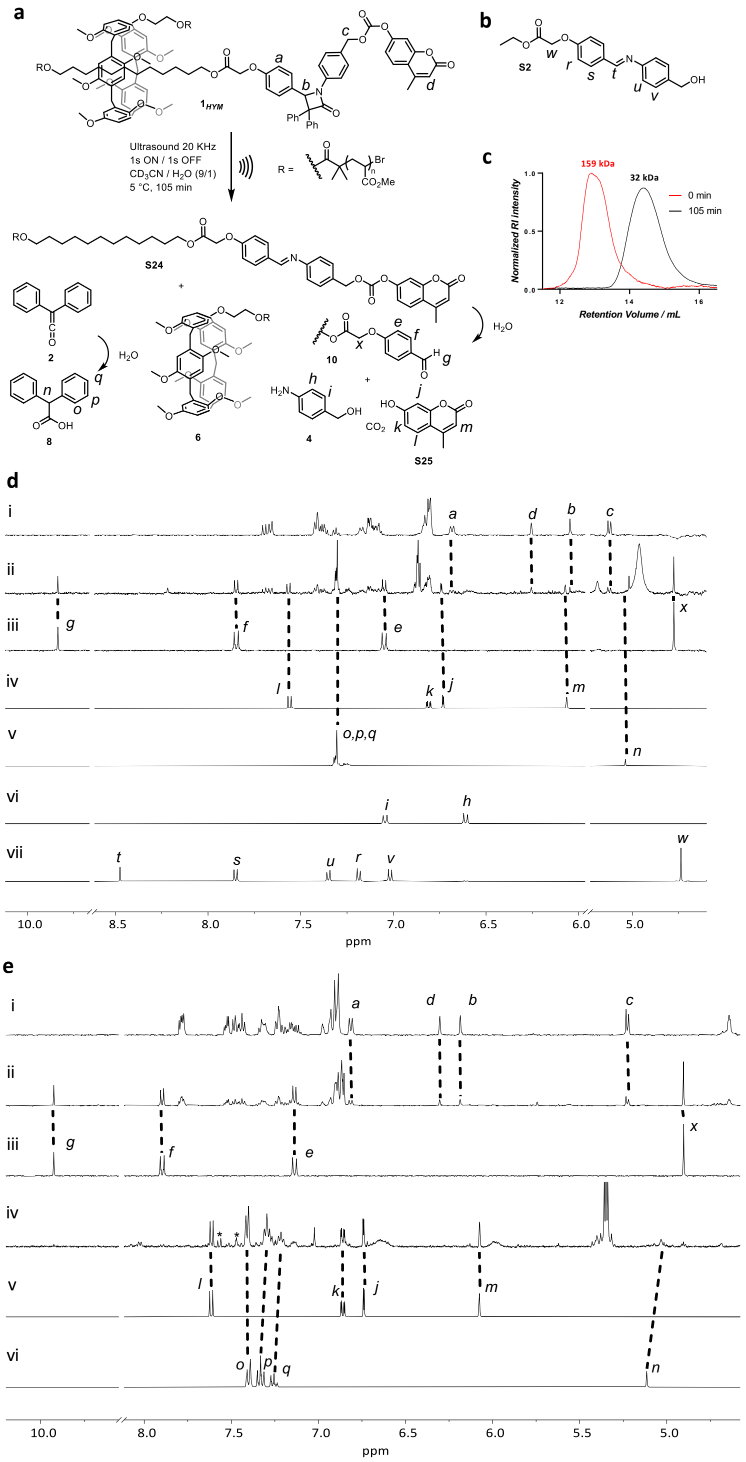

Figure S7. Sonication (run 1) of polymer $\mathbf{1}_{HYM}$ in CD_3CN/H_2O (9/1). Sonication of polymer $\mathbf{1}_{HYM}$ affords fragments **10**, **4**, **S25** and CO_2 from cascade reaction, **8** upon hydrolysis of **2**, and **6** (a). Reference compound **S2** (b). SEC traces of polymer $\mathbf{1}_{HYM}$ (c) before (red) and after (black) sonication. Partial ^1H NMR (500 MHz, Acetonitrile-d_3/H_2O (9/1), 298 K) spectra comparison of (d) polymer $\mathbf{1}_{HYM}$ before (i), and after sonication (ii), reference polymer **10** (iii), reference compound **8** (iv), reference compound **4** (v), and reference compound **S2** (vi). Partial ^1H NMR (500 MHz, Acetone-d_6, 298 K) spectra comparison of (e) polymer $\mathbf{1}_{HYM}$ before (i), and after sonication and MeOH wash (ii), reference polymer **10** (iii), concentrated methanol washings (iv), reference compound **S25** (v), and reference compound **8** (vi). * Peaks marked with star are external impurities. Details see *Section 5.8*.

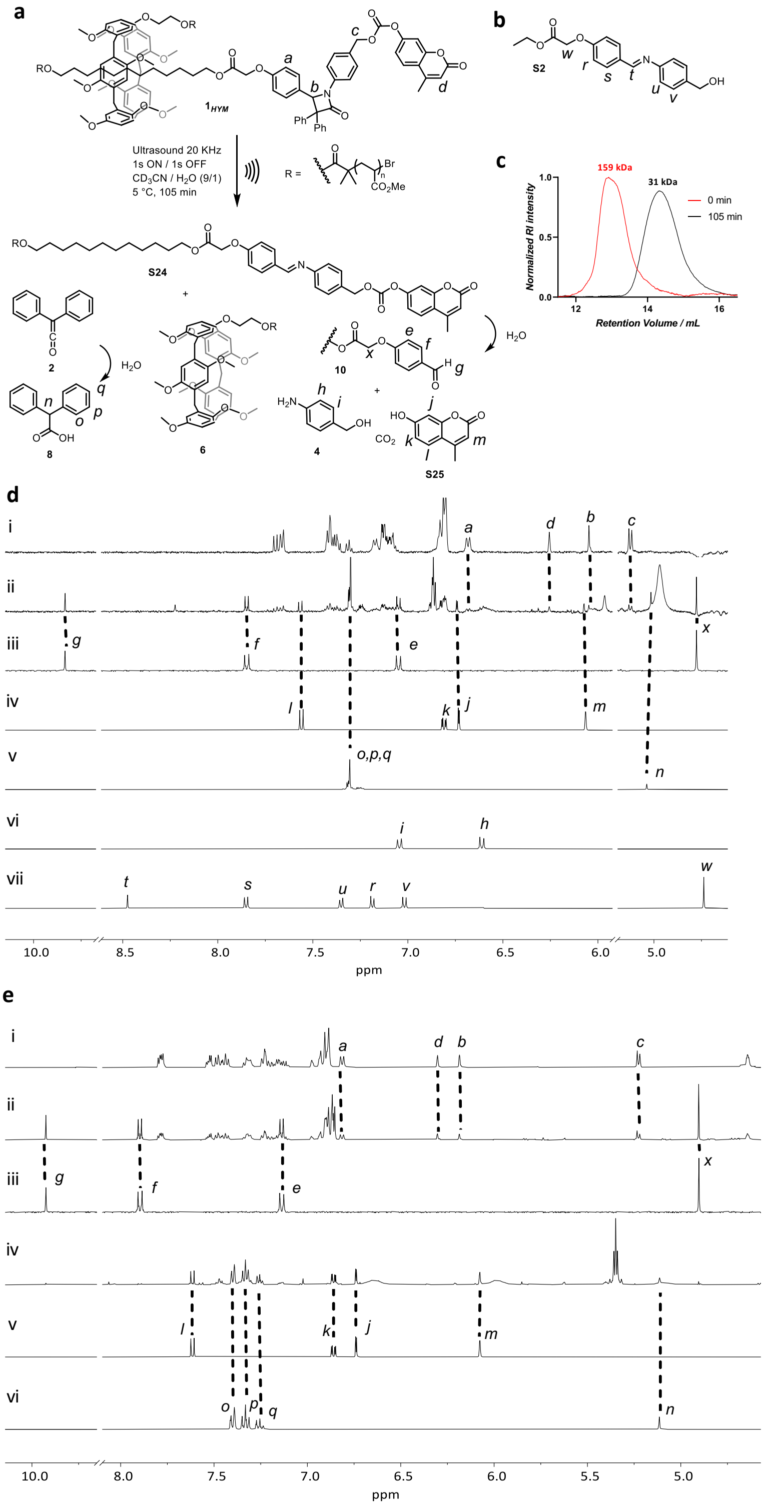

Figure S8. Sonication (run 2) of polymer $\mathbf{1}_{HYM}$ in CD_3CN/H_2O (9/1). Sonication of polymer $\mathbf{1}_{HYM}$ affords fragments **10**, **4**, **S25** and CO_2 from cascade reaction, **8** upon hydrolysis of **2**, and **6** (a). Reference compound **S2** (b). SEC traces of polymer $\mathbf{1}_{HYM}$ (c) before (red) and after (black) sonication. Partial ^{1}H NMR (500 MHz, Acetonitrile-d_3/H_2O (9/1), 298 K) spectra comparison of (d) polymer $\mathbf{1}_{HYM}$ before (i), and after sonication (ii), reference polymer **10** (iii), reference compound **8** (iv), reference compound **4** (v), and reference compound **S2** (vi). Partial ^{1}H NMR (500 MHz, Acetone-d_6, 298 K) spectra comparison of (e) polymer $\mathbf{1}_{HYM}$ before (i), and after sonication and MeOH wash (ii), reference polymer **10** (iii), concentrated methanol washings (iv), reference compound **S25** (v), and reference compound **8** (vi).

The comparison of fluorescence spectra of polymer **1***HYM* before and after sonication in CD_3CN/H_2O (9/1) also confirms the release of hymecromone as its fluorescence is quenched when attached to the mechanophore.
Additionally, we also confirmed this phenomenon in the BMG activation (section 7.4).

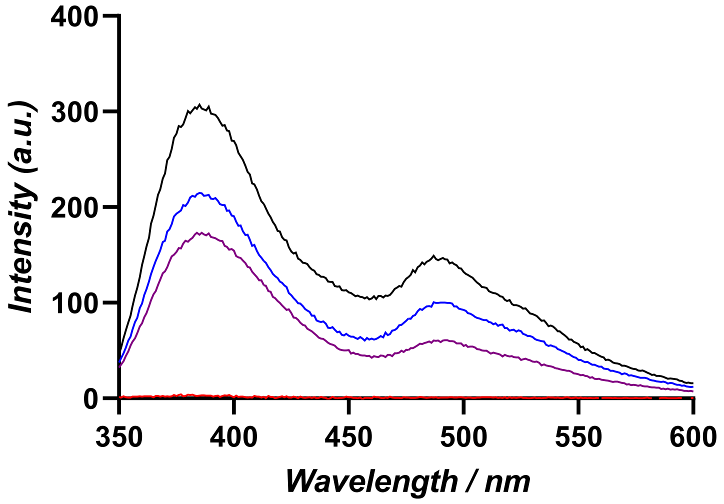

Figure S9. Fluorescence spectra, taken in CD_3CN/H_2O (9/1), of the pre-sonication polymer **1***HYM* (red, 20 nmol/mL), post-sonication polymer before being washed with methanol (blue, 20 nmol/mL for polymer **1***HYM*), post-BMG polymer washing phase (purple, 20 nmol/mL for polymer **1***HYM*, see section 7.4) and reference **S25** (black, 15 nmol/mL). The excitation wavelength was 320 nm.

5.5 Sonication of Mechanophore Polymer 1*GEM*

Sonication of mechanophore polymer **1***GEM*, containing gemcitabine (chemotherapy drug) attached to the β-lactam core via a self-immolative liner, using the methodology described in the general procedure (*Section 5.1*) and with CD_3CN/H_2O (9/1), was carried out twice to determine the extent of activation of mechanophore activation and cargo release. SEC analysis of the sonicated polymers showed complete cleavage (M_n of the post-sonication material was less than half of that of the pre-sonication polymer).

Similar to polymer **1***HYM*, sonication results of polymer **1***GEM* also showed that retro-[2+2] cycloaddition of β-lactam mechanophore occurred and triggered the cascade reaction leading to the release of gemcitabine. It is evidenced by the emergence of signals pertaining to an aromatic aldehyde (*e*-*g*), diphenylacetic acid (*n*-*q*) and gemcitabine (*j*-*l*) while signals of the initial β-lactam mechanophore decreased in intensity. Peaks (*e*-*g*) were confirmed to be aldehyde polymer after comparing with reference **10**. Diphenylacetic acid and gemcitabine were identified in the crude mixture and later in MeOH extracts. ^{19}F NMR spectra of concentrated methanol washings also confirmed the releasing of gemcitabine.

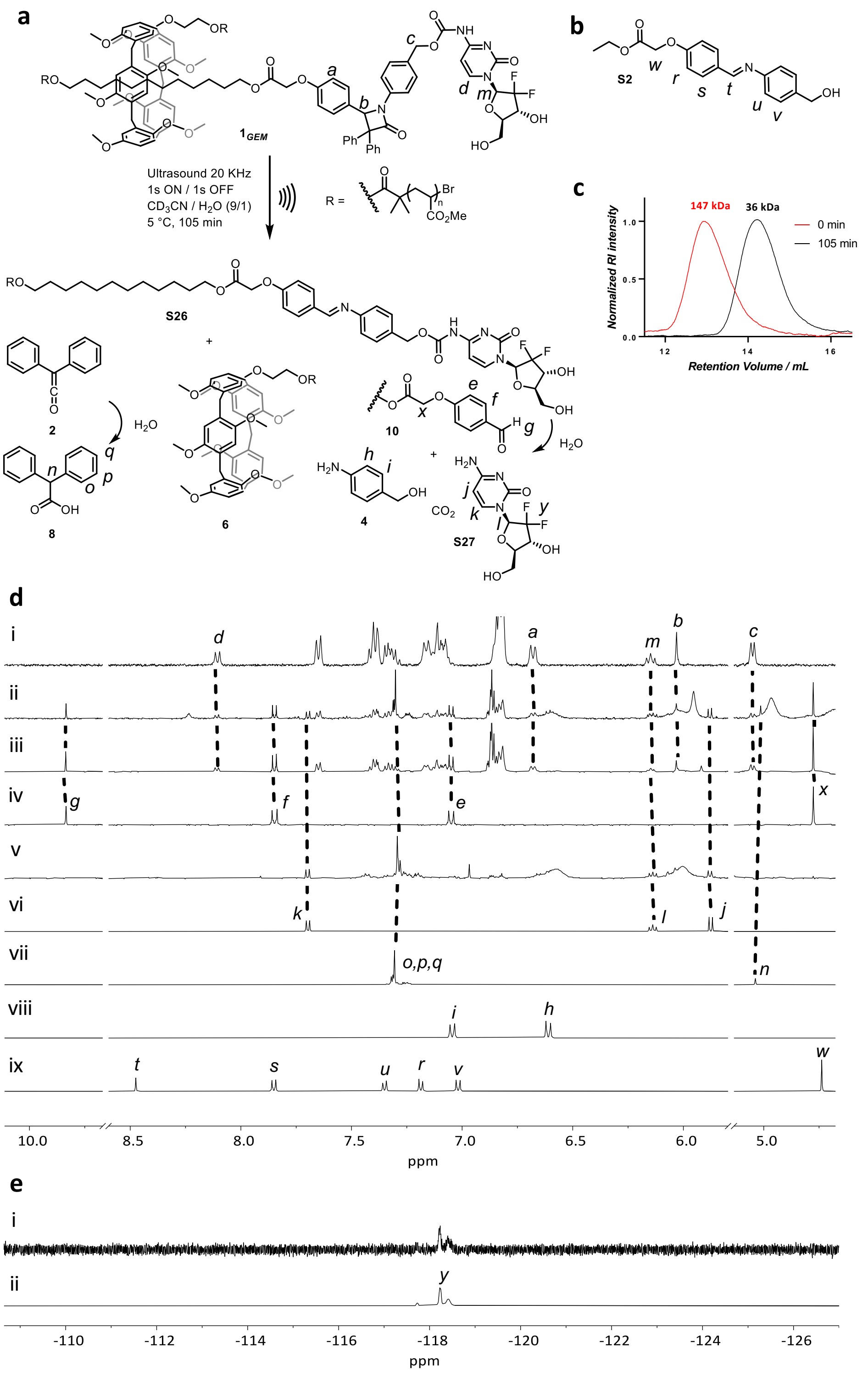

Figure S10. Sonication (run 1) of polymer $\mathbf{1}_{GEM}$ in CD_3CN/H_2O (9/1). Sonication of polymer $\mathbf{1}_{GEM}$ affords fragments **10**, **4**, **S27** and CO_2 from cascade reaction, **8** upon hydrolysis of **2**, and **6** (a). Reference compound **S2** (b). SEC traces of polymer $\mathbf{1}_{GEM}$ (c) before (red) and after (black) sonication. Partial ^{1}H NMR (500 MHz, Acetonitrile-d_3/H_2O (9/1), 298 K) spectra comparison of (d) polymer $\mathbf{1}_{GEM}$ before (i), after sonication (ii), after sonication and MeOH wash (iii), reference polymer **10** (iv), concentrated methanol washings (v), reference compound **S27** (vi), reference compound **8** (vii), reference compound **4** (viii), and reference polymer $\mathbf{9}_{\text{-}114}$ (ix). Partial ^{19}F NMR (471 MHz, Acetonitrile-d_6/H_2O (9/1), 298 K) spectra comparison (e) of concentrated methanol washings (i), and reference compound **S27** (ii).

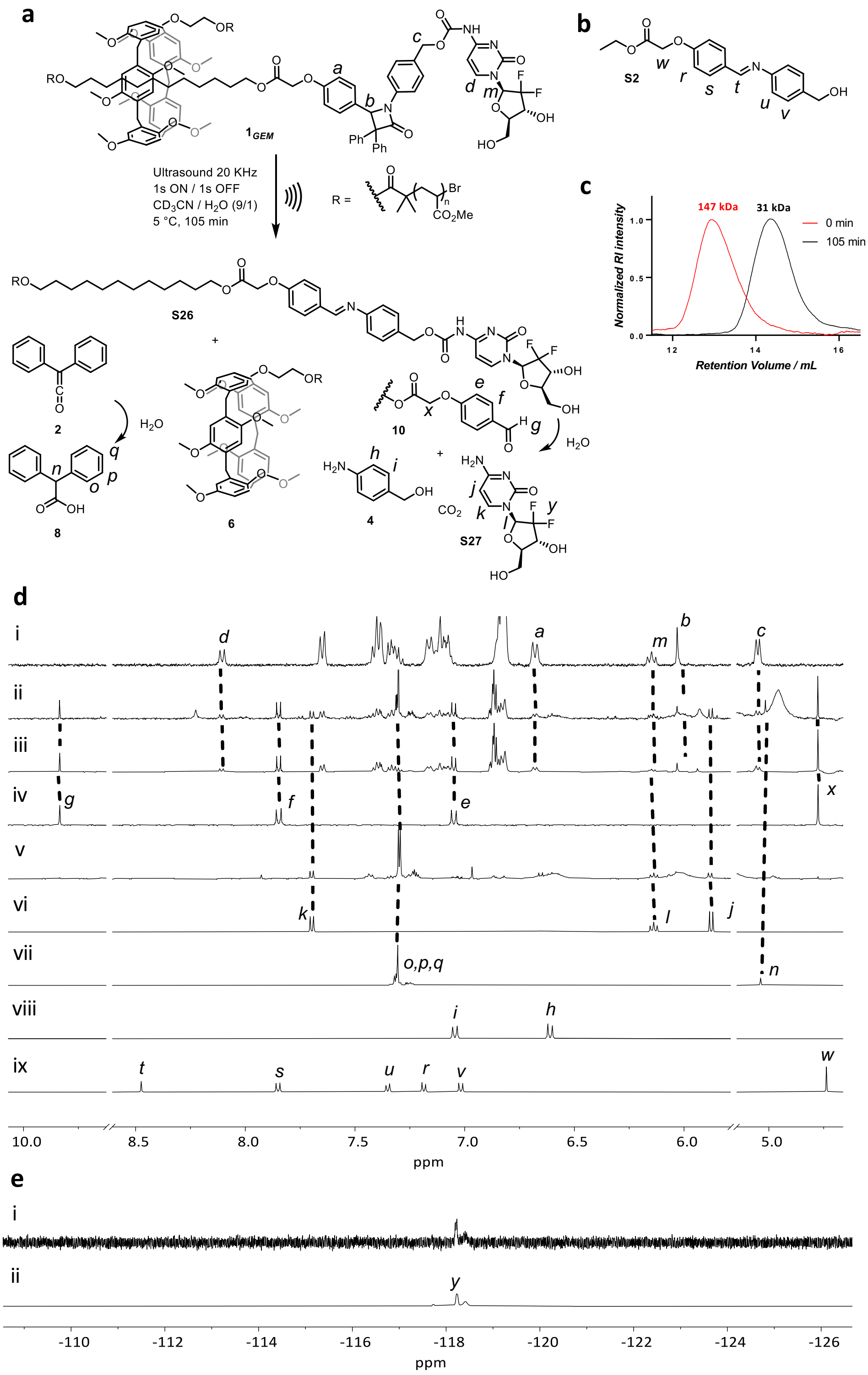

Figure S11. Sonication (run 2) of polymer **1*GEM*** in CD_3CN/H_2O (9/1). Sonication of polymer **1*GEM*** affords fragments **10**, **4**, **S27** and CO_2 from cascade reaction, **8** upon hydrolysis of **2**, and **6** (a). Reference compound **S2** (b). SEC traces of polymer **1*GEM*** (c) before (red) and after (black) sonication. Partial ^{1}H NMR (500 MHz, Acetonitrile-*d*$_3$/H_2O (9/1), 298 K) spectra comparison of (d) polymer **1*GEM*** before (i), after sonication (ii), after sonication and MeOH wash (iii), reference polymer **10** (iv), concentrated methanol washings (v), reference compound **S27** (vi), reference compound **8** (vii), reference compound **4** (viii), and reference polymer **9**$_{\text{-114}}$ (ix). Partial ^{19}F NMR (471 MHz, Acetonitrile-*d*$_6$/H_2O (9/1), 298 K) spectra comparison (e) of concentrated methanol washings (i), and reference compound **S27** (ii).

5.6 Sonication of Control Polymers S20 and S21

Sonication of control polymers **S20** and **S21**, using the methodology described in the general procedure (*Section 5.1*) in CD_3CN/H_2O (9/1), was carried out to determine the extent of activation for retro-[2+2] cycloaddition of β-lactam mechanophore and stability of carbonate linker in **S21**. SEC analysis of the sonicated polymers showed complete cleavage (M_n of the post-sonication material was less than half of that of the pre-sonication polymer).

Comparison of the ^{1}H NMR spectra of control polymers **S20** and **S21** before and after sonication confirms the stability of the β-lactam mechanophore in the absence of a rotaxane actuator.

Additionally, hymocromone was not observed in the crude mixture or the MeOH extracts from the sonication of **S21**, confirming the stability of the carbonate linker during sonication.

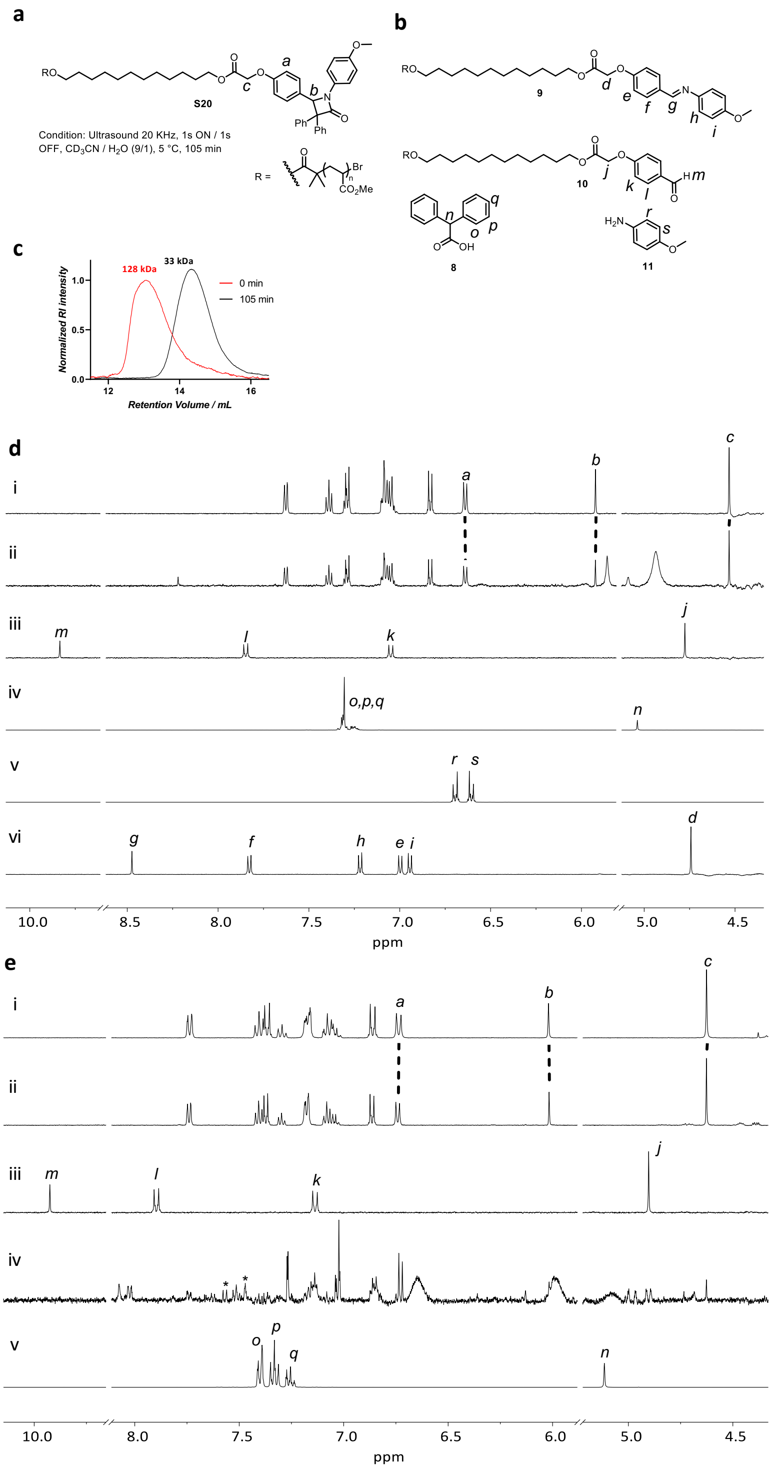

Figure S12. Sonication of polymer **S20** in CD_3CN/H_2O (9/1). Structure of polymer **S20** and condition of sonication (a). Reference species **9**$_{-114}$, **10**, **11** and **8** (b). SEC traces of polymer **S20** (c) before (red) and after (black) sonication. Partial ^1H NMR (500 MHz, Acetonitrile-*d*$_3$/H_2O (9/1), 298 K) spectra comparison of (d) polymer **S20** before (i), and after sonication (ii), reference polymer **10** (iii), reference compound **8** (iv), reference compound **11** (v), and reference polymer **9**$_{-114}$ (vi). Partial ^1H NMR (500 MHz, Acetone-*d*$_6$, 298 K) spectra comparison of (e) polymer **S20** before (i), and after sonication and MeOH wash (ii), reference polymer **10** (iii), concentrated methanol washings (iv), and reference compound **8** (v). * Peaks marked with star are external impurities. Details see *Section 5.8*.

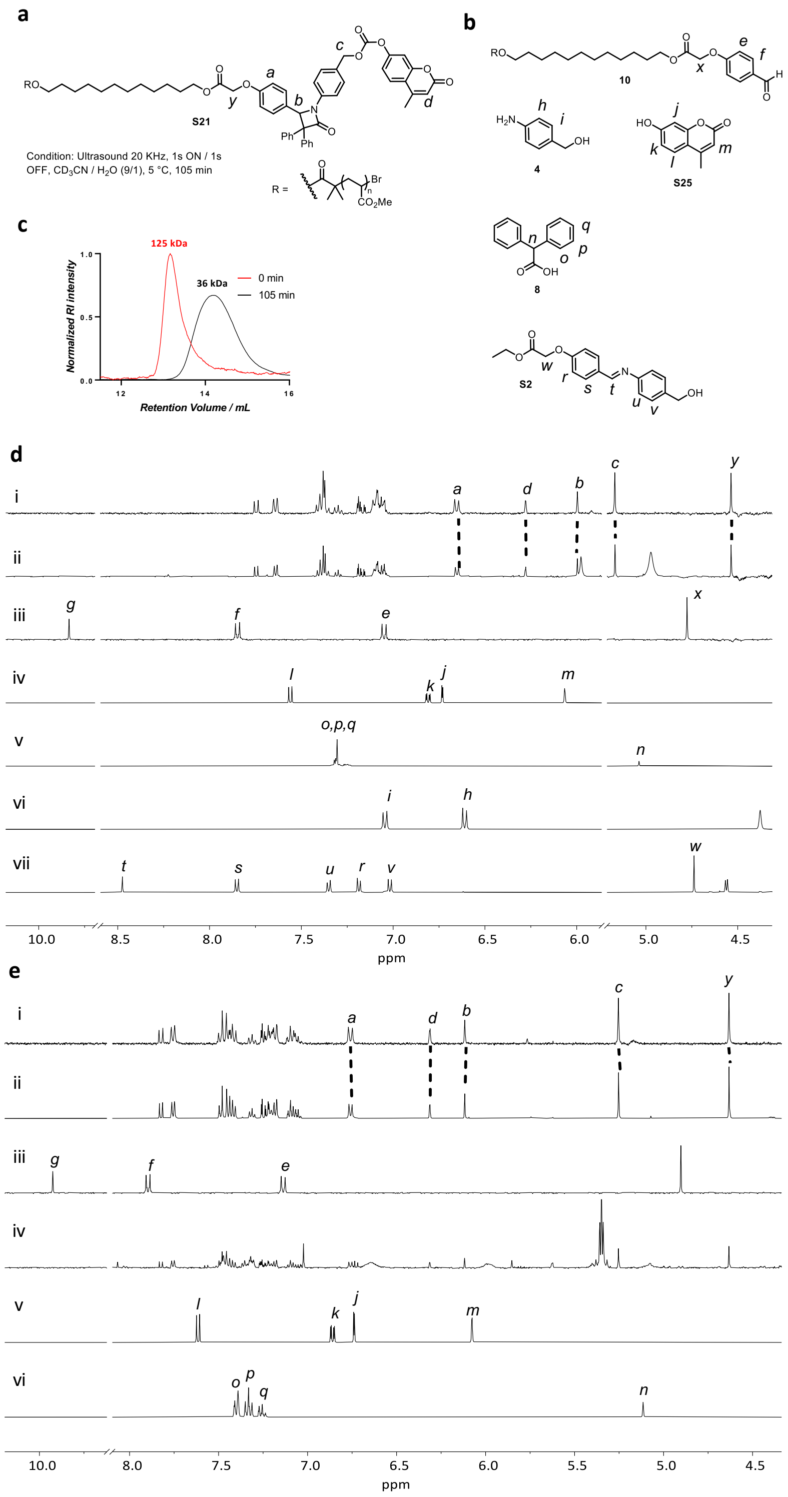

Figure S13. Sonication of polymer **S21** in CD_3CN/H_2O (9/1). Structure of polymer **S21** and condition of sonication (a). Reference species **10**, **4**, **S25**, **8** and **S2** (b). SEC traces of polymer **S21** (c) before (red) and after (black) sonication. Partial ^{1}H NMR (500 MHz, Acetonitrile-d_3/H_2O (9/1), 298 K) spectra comparison of (d) polymer **S21** before (i), and after sonication (ii), reference polymer **10** (iii), reference compound **S25** (iv), reference compound **8** (v), reference compound **4** (vi), and reference polymer **S2** (vii). Partial ^{1}H NMR (500 MHz, Acetone-d_6, 298 K) spectra comparison of (e) polymer **S21** before (i), and after sonication and MeOH wash (ii), reference polymer **10** (iii), concentrated methanol washings (iv), reference compound **S25** (v), and reference compound **8** (vi).

5.7 Sonication of Control Polymer $\mathbf{9}_{\text{-}112}$

Sonication of control polymer $\mathbf{9}_{\text{-}112}$, using the methodology described in the general procedure (*Section 5.1*) in CD_3CN/H_2O (9/1), was carried out to determine the hydrolysis of imine polymer. SEC analysis of the sonicated polymer showed complete cleavage (M_n of the post-sonication material was less than half of that of the pre-sonication polymer).

Comparison of the ^{1}H NMR spectra of polymer $\mathbf{9}_{\text{-}112}$ before and after sonicaiton and reference compounds showed it fully hydrolysed after sonication and incubation.

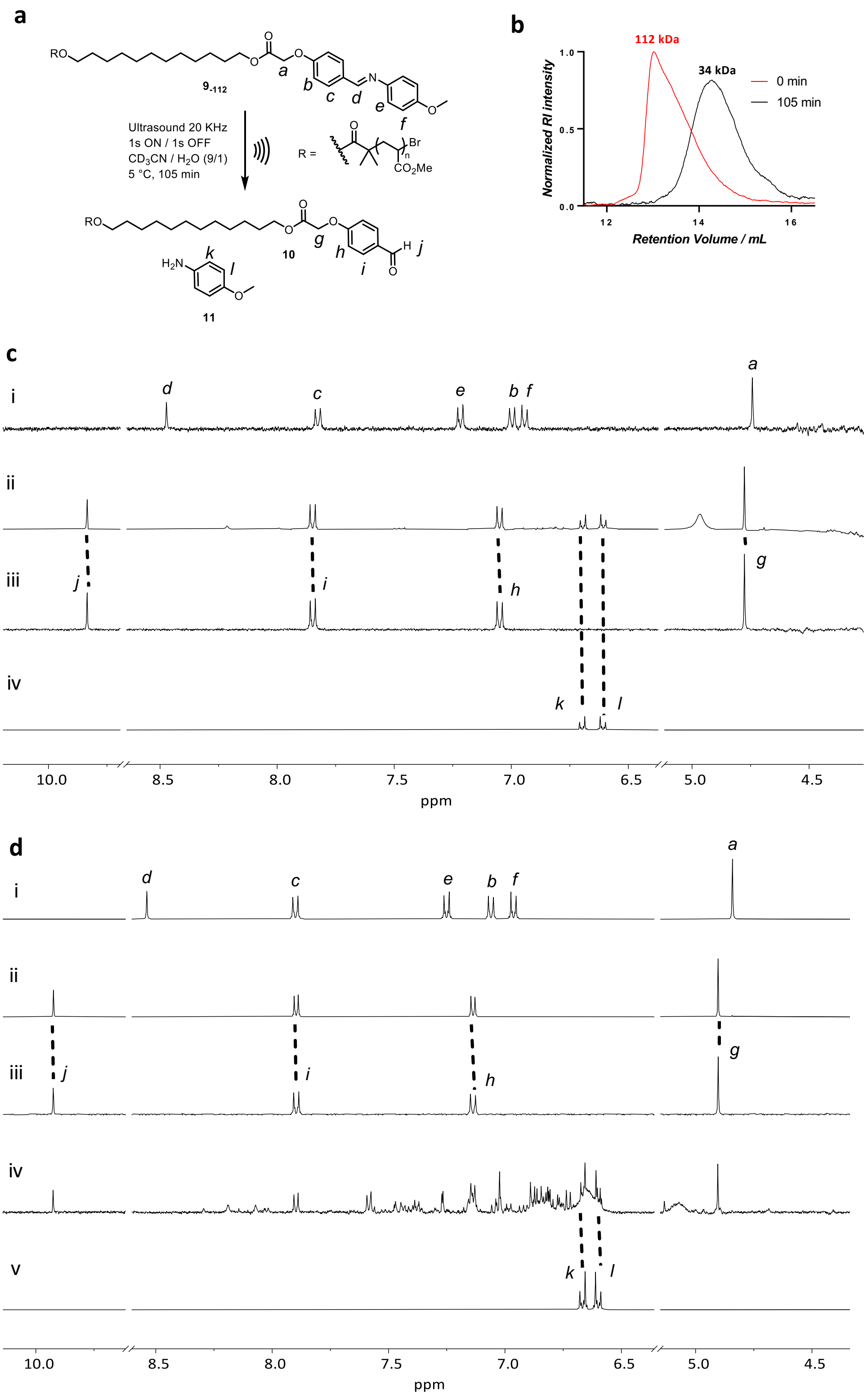

Figure S14. Sonication of polymer $\mathbf{9}_{\mathbf{-112}}$ in CD_3CN/H_2O (9/1). Sonication of polymer $\mathbf{9}_{\mathbf{-112}}$ affords fragments **10** and **11** (a). SEC traces of polymer $\mathbf{9}_{\mathbf{-112}}$ (b) before (red) and after (black) sonication. Partial ^1H NMR (500 MHz, Acetonitrile-d_3/H_2O (9/1), 298 K) spectra comparison of (c) polymer $\mathbf{9}_{\mathbf{-112}}$ before (i), after sonication (ii), reference polymer **10** (iii), and reference compound **11** (v). Partial ^1H NMR (500 MHz, Acetone-d_6, 298 K) spectra comparison of (d) polymer $\mathbf{9}_{\mathbf{-112}}$ before (i), and after sonication and MeOH (ii), reference polymer **10** (iii), concentrated methanol washings (iv), and reference compound **11** (v).

5.8 Analysis of Impurities in MeOH Extract from Polymer after Activation

Here we use MeOH extract from post-sonication polymer **5_{-138}** (run 2) as an example to analyze the external impurities. We confirmed that peaks of impurities are from extraction of syringe or trace contaminant in methanol after ^{1}H NMR comparison.

In the extraction of syringe, 30 mL MeCN in a beaker was taken up in a 24 mL plastic syringe (HENKE-JECT) and then eject MeCN back to beaker after waiting for 5 min as one cycle. After three cycyles of this process, collect ^{1}H NMR after evaporation of the solvent. For determination of trace contaminant in methanol, 500 mL methanol (Honeywell) was evaporated and then run ^{1}H NMR.

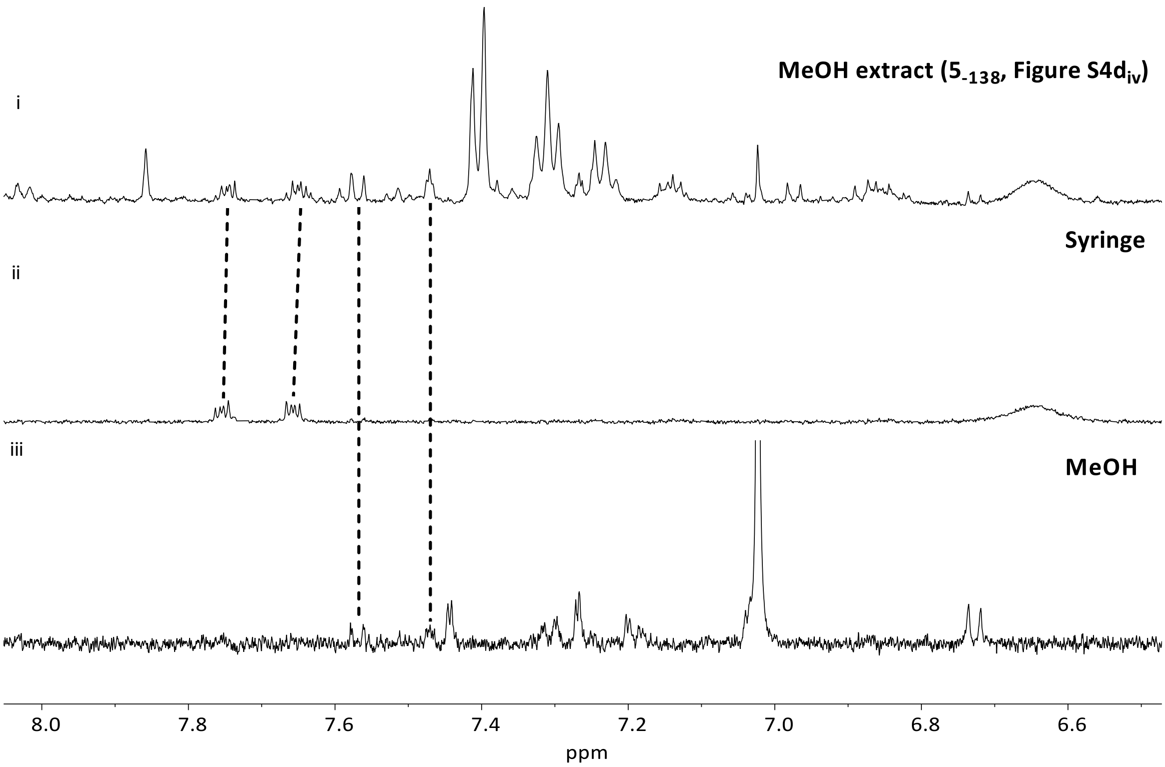

Figure S15. Partial ^{1}H NMR (500 MHz, Acetone-d_6, 298 K) spectra comparison of MeOH extract from post-sonication of polymer **5_{-138}** (i), extract from syringe (ii), and methanol residue after evaporation (iii).

6 Activation in Bulk by Compression

6.1 General Procedure for Compression Experiments

The appropriate polymer (8 mg) was formed into a rough spherical shape by hand. The material was placed inbetween the anvils within a standard 13 mm KBr pellet die. 10 tonnes of compressive force was then applied; as the material was compressed, the pressure was relieved gradually by rearrangement of the material so over the course of a period of up to 60 minutes it was ensured that 10 tonnes of force was being continuously applied. The pressure was then released and the flattened material folded in half as many times as possible before being placed back into the pellet die and compressed further. This cyclical process of folding followed by compression was repeated until SEC analysis of the material showed adequate reduction in the M_n (at least 50% of the initial pre-compression M_n). At this point, the material was dissolved in acetonitrile and carefully filtered (0.45 µm PTFE membrane) to remove any metal particulate before being condensed *in vacuo*. The crude polymer material was analysed by ^{1}H NMR spectroscopy before being thoroughly dried once more and subsequently directly washed over with MeOH (at least 5 x 10 mL). The MeOH washings were collected, condensed *in vacuo* and analysed by ^{1}H NMR spectroscopy along with the washed polymer material itself.

6.2 Compression of Mechanophore Polymer 5_{-138}

Polymer $\mathbf{5}_{-138}$ were subjected to the general compression activation methodology described in *Section 6.1*. Analysis of the material post-activation was carried out in an identical manner to that of the sonication of the polymer with the same mechanophore, as described in *Section 5.2*.

Comparison of the ^{1}H NMR spectra of polymer $\mathbf{5}_{-138}$ before and after compression showed that the retro-[2+2] cycloaddition of the β-lactam mechanophore occurred upon compression. Compared with the result from sonication, compression activation gave a lower conversion as evidenced by the formation of small amount of imine and aldehyde polymers and a small decrease in the signlas of the intact β-lactam mechanophore. Partial hydrolysis of imine polymer is due to the trace water in the polymer and ambient humidity during the process of compression (no care was taken to prevent this). Peaks (*g, m*) were confirmed to belong to the imine and aldehyde polymers respectively. Peaks (*n-q*) belonging to diphenylacetic acid are clearly present in the concentrated methanol exctracts.

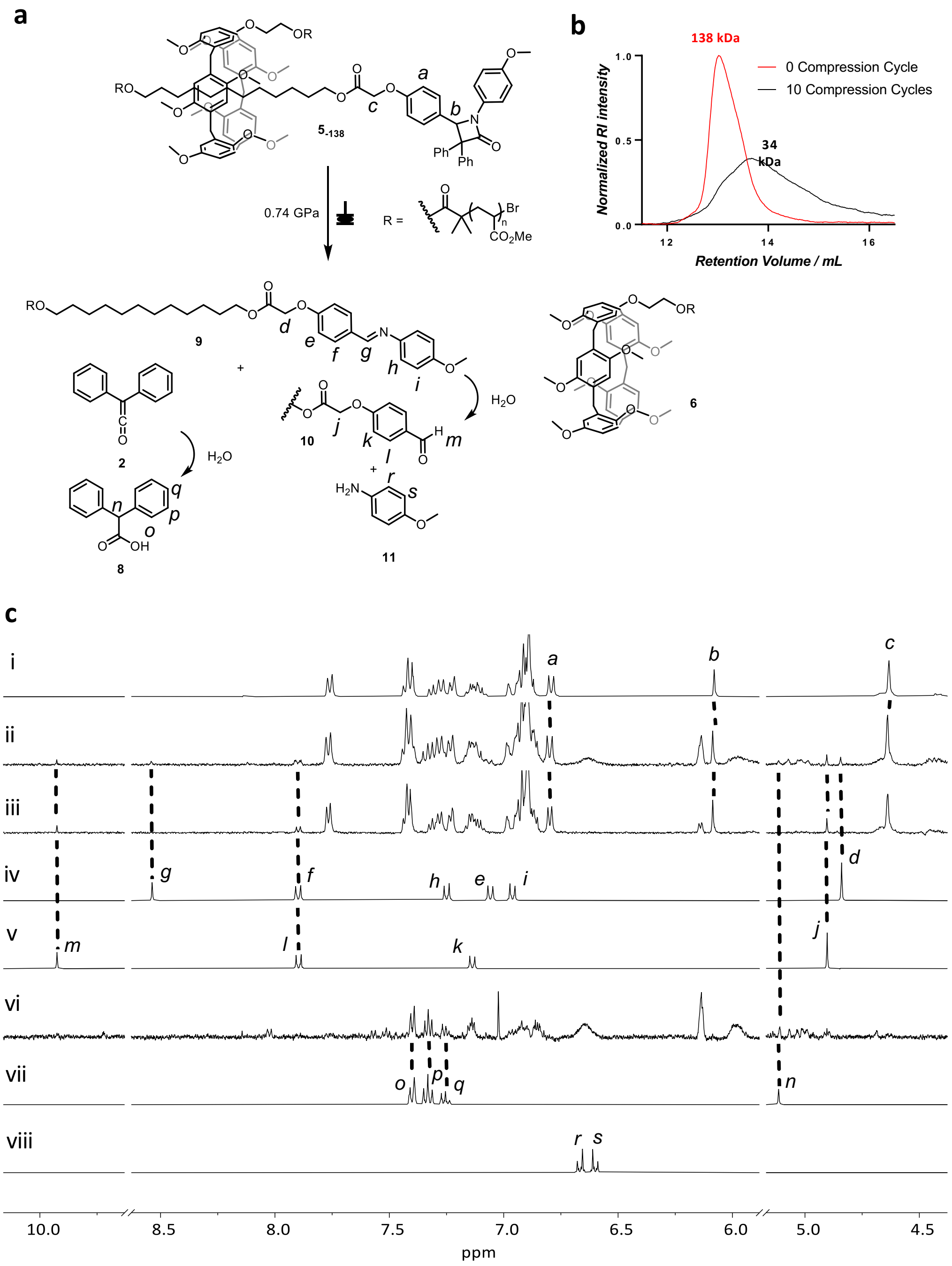

Figure S16. Compression activation (run 1) of polymer **5**$_{-138}$. Compression activation of polymer **5**$_{-138}$ affords fragments **10** and **11**, upon hydrolysis of **9**$_{-114}$, **8**, upon hydrolysis of **2**, and **6** (a). SEC traces of polymer **5**$_{-138}$ (b) before (red) and after (black) compression. Partial NMR (500 MHz, Acetone-d_6, 298 K) spectra comparison of (c) polymer **5**$_{-138}$ before (i), after compression (ii), and after compression and MeOH wash (iii), reference polymer **9**$_{-114}$ (iv), reference polymer **10** (v), concentrated methanol washings (vi), reference compound **8** (vii), and reference compound **11** (viii).

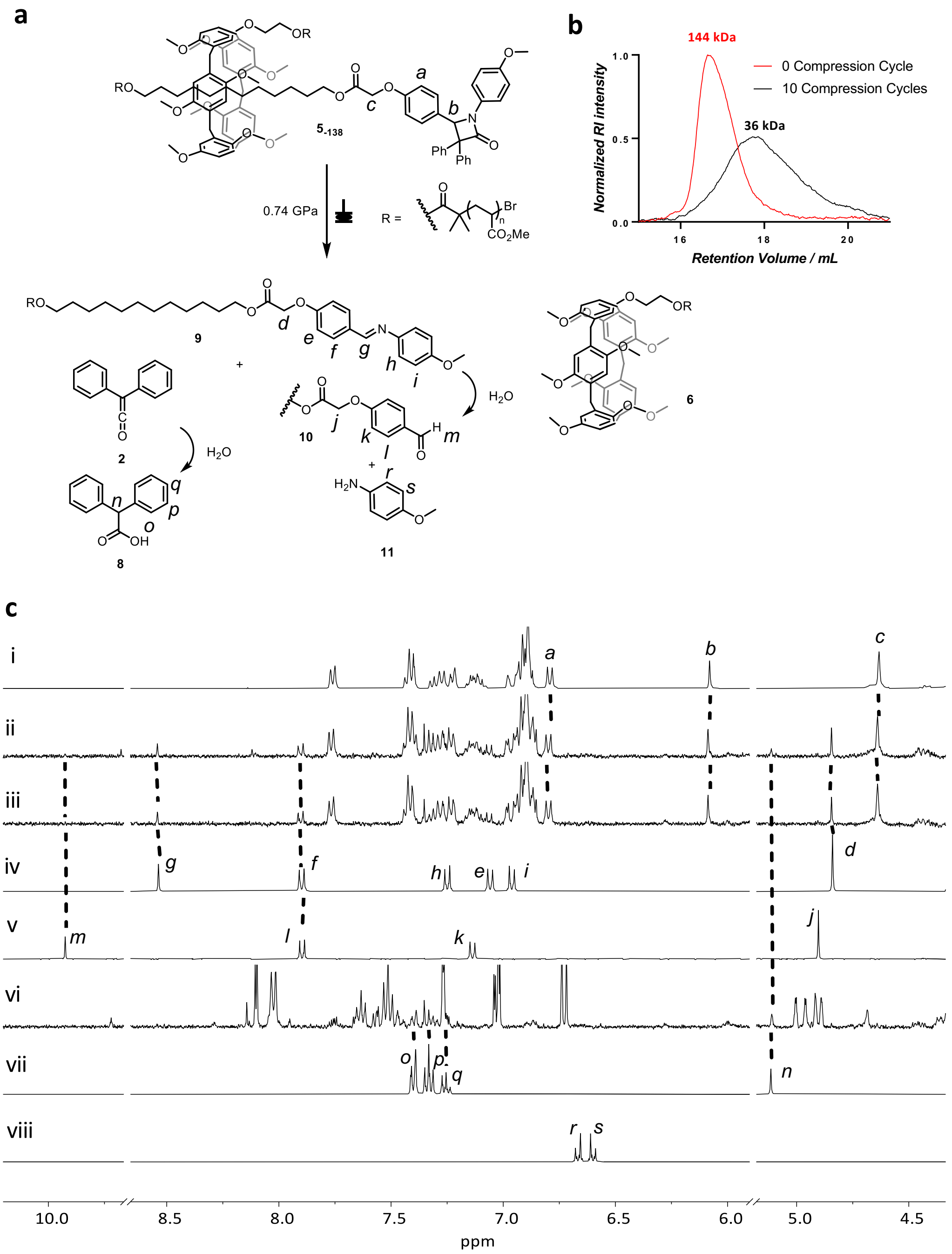

Figure S17. Compression activation (run 2) of polymer **5**$_{-138}$. Compression activation of polymer **5**$_{-138}$ affords fragments **10** and **11**, upon hydrolysis of **9**$_{-114}$, **8**, upon hydrolysis of **2**, and **6** (a). SEC traces of polymer **5**$_{-138}$ (b) before (red) and after (black) compression (DMAc system was used in this case). Partial NMR (500 MHz, Acetone-d_6, 298 K) spectra comparison of (c) polymer **5**$_{-138}$ before (i), after compression (ii), and after compression and MeOH wash (iii), reference polymer **9**$_{-114}$ (iv), reference polymer **10** (v), concentrated methanol washings (vi), reference compound **8** (vii), and reference compound **11** (viii).

6.3 Compression of Control Polymer S20

Polymer **S20** were subjected to the general bulk activation methodology described in *Section 6.1*. Analysis of the material post-activation was carried out to confirm the stability of the β-lactam mechanophore during the compression.

Comparison of the ^{1}H NMR spectra of polymer **S20** before and after compression and reference compounds showed that retro-[2+2] cycloaddition reaction did not occur, which indicates the β-lactam mechanophore in the control polymer without rotaxane actuator is stable during compression.

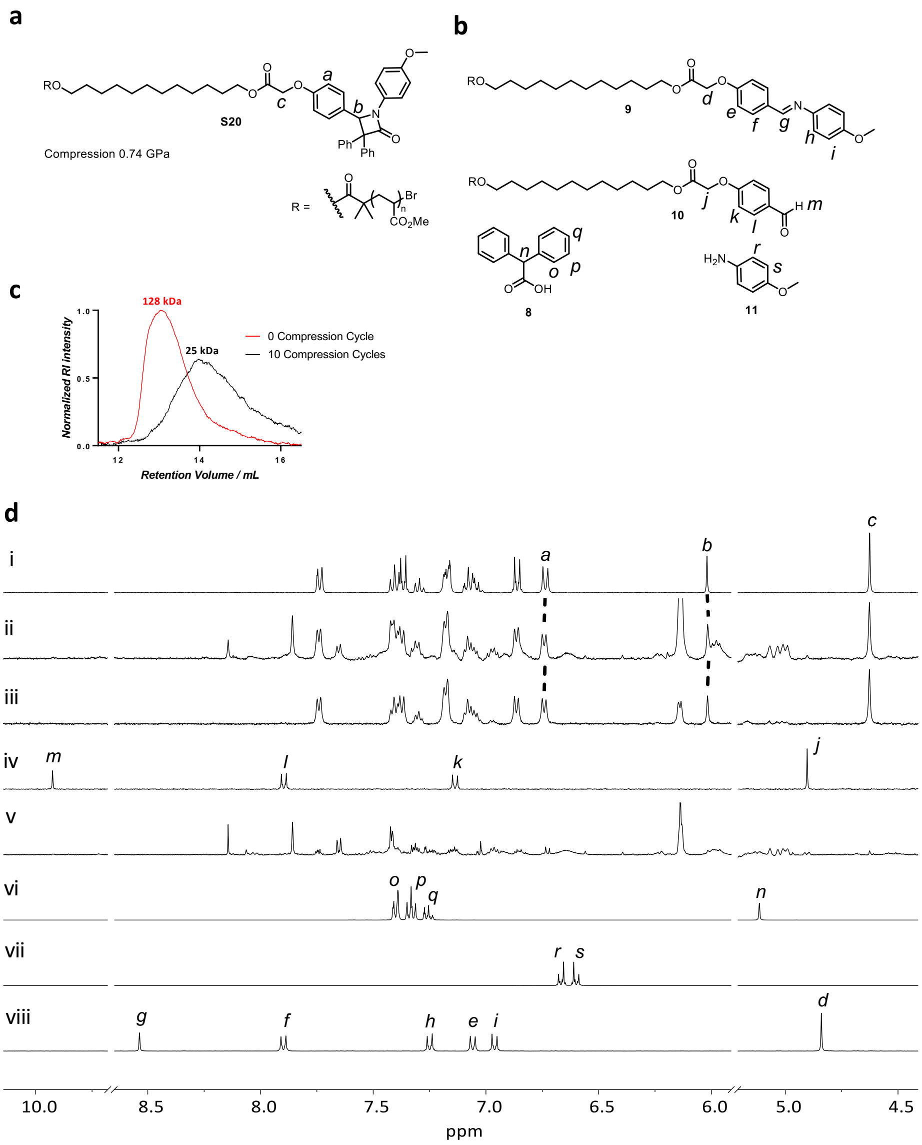

Figure S18. Compression activation of polymer **S20**. Structure of polymer **S20** and condition of compression (a). Reference species **9**$_{\text{-114}}$, **10**, **11** and **8** (b). SEC traces of polymer **S20** (c) before (red) and after (black) compression. Partial ^{1}H NMR (500 MHz, Acetone-d_6, 298 K) spectra comparison of (e) polymer **S20** before (i), after compression (ii), and after compression and MeOH wash (iii), reference polymer **10** (iv), concentrated methanol washings (v), reference compound **8** (vi), reference compound **11** (vii), and reference polymer **9**$_{\text{-114}}$ (viii).

6.4 Compression of Control Polymer $\mathbf{9}_{\text{-}112}$

Polymer $\mathbf{9}_{\text{-}112}$ were subjected to the general bulk activation methodology described in *Section 6.1*. Analysis of the material post-activation was carried out to confirm the stability of imine polymer during the compression.

Comparison of the ^{1}H NMR spectra of polymer $\mathbf{9}_{\text{-}112}$ before and after compression and reference compounds showed it partially hydrolysed to amine polymer during the compression due to the trace water in the polymer and ambient humidity.

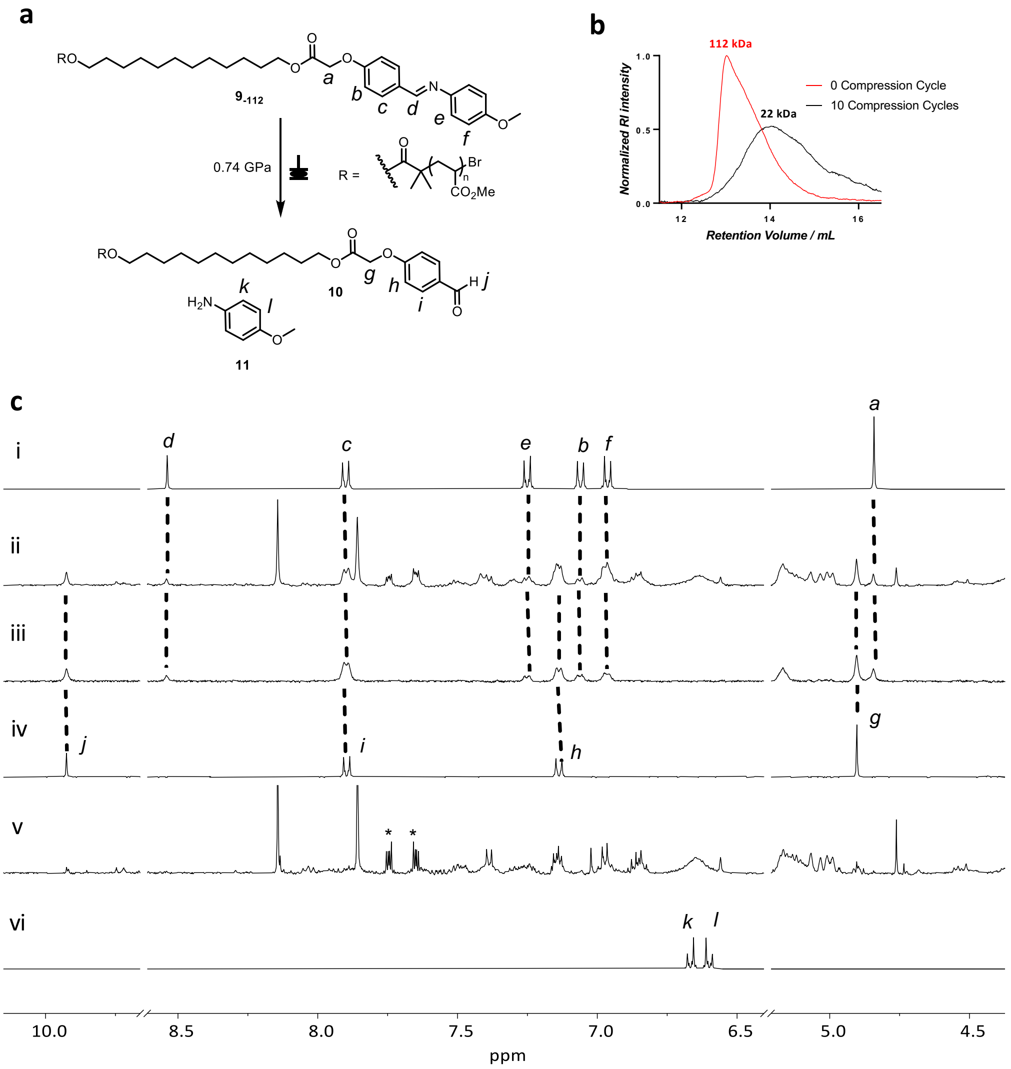

Figure S19. Compression activation of polymer $\mathbf{9}_{\text{-}112}$. Compression of polymer $\mathbf{9}_{\text{-}112}$ affords fragments **10** and **11** (a). SEC traces of polymer $\mathbf{9}_{\text{-}112}$ (b) before (red) and after (black) compression. Partial ^{1}H NMR (500 MHz, Acetone-d_6, 298 K) spectra comparison of (c) polymer $\mathbf{9}_{\text{-}112}$ before (i), after compression (ii), and after compression and MeOH wash (iii), reference polymer **10** (iv), concentrated methanol washings (v), and reference compound **11** (vi). * Peaks marked with star are external impurities. Details see *Section 5.8*.

7 Activation in Bulk by BMG

7.1 General Procedure for BMG Experiments

Dried polymer (a stainless steel jar containing 12mg of polymer was placed in the vacuum oven at 45 °C for 48 h before use = dry condition) or polymer/water (1/1, w/w, 12 mg = wet condition) with two stainless steel balls (diameter 7 mm) were placed in a stainless steel BMG jar. Then the BMG jar was frozen in liquid nitrogen for 3 min before milling (30 kHz) for 15 min. The freezing and milling were repeated for 4 cycles (60 min total milling time). After incubation for 16 h at room temperature, the material was dissolved in deuterium solvent and carefully filtered (0.45 µm PTFE membrane) to remove any metal particulate before analysis by ^{1}H NMR spectroscopy. The polymer material was also analysed by ^{1}H NMR spectroscopy after being dried and subsequently directly washed with MeOH (at least 5 x 10 mL). The MeOH washings were collected, condensed *in vacuo* and analysed by ^{1}H NMR spectroscopy along with the washed polymer material.

7.2 BMG of Mechanophore Polymer $\mathbf{5_{-155}}$ in Dry Condition

Polymer $\mathbf{5_{-155}}$ was subjected to the general bulk activation methodology in dry conditions described in *Section 7.1*. Analysis of the material post-activation was carried out in an identical manner to that of the polymer sonication with the same mechanophore, as described in *Section 5.2*.

Comparison of the ^{1}H NMR spectra of polymer $\mathbf{5_{-155}}$ before and after BMG showed that retro-[2+2] cycloaddition of β-lactam mechanophore occureed. It is evidenced by presence of imine polymer while the initial β-lactam mechanophore decreased in intensity. Peaks (*d-i*) confirmed to be imine polymer after comparing with reference $\mathbf{9_{-114}}$. Although the polymer was dried, we still can see a tiny peak (*j,m*) which was confirmed to be aldehyde polymer from hydrolysis of imine polymer after comparing with reference **10** due to adventitious water in the experimental setup. However, this did not lead to observal quantities of diphenylacetic acid.

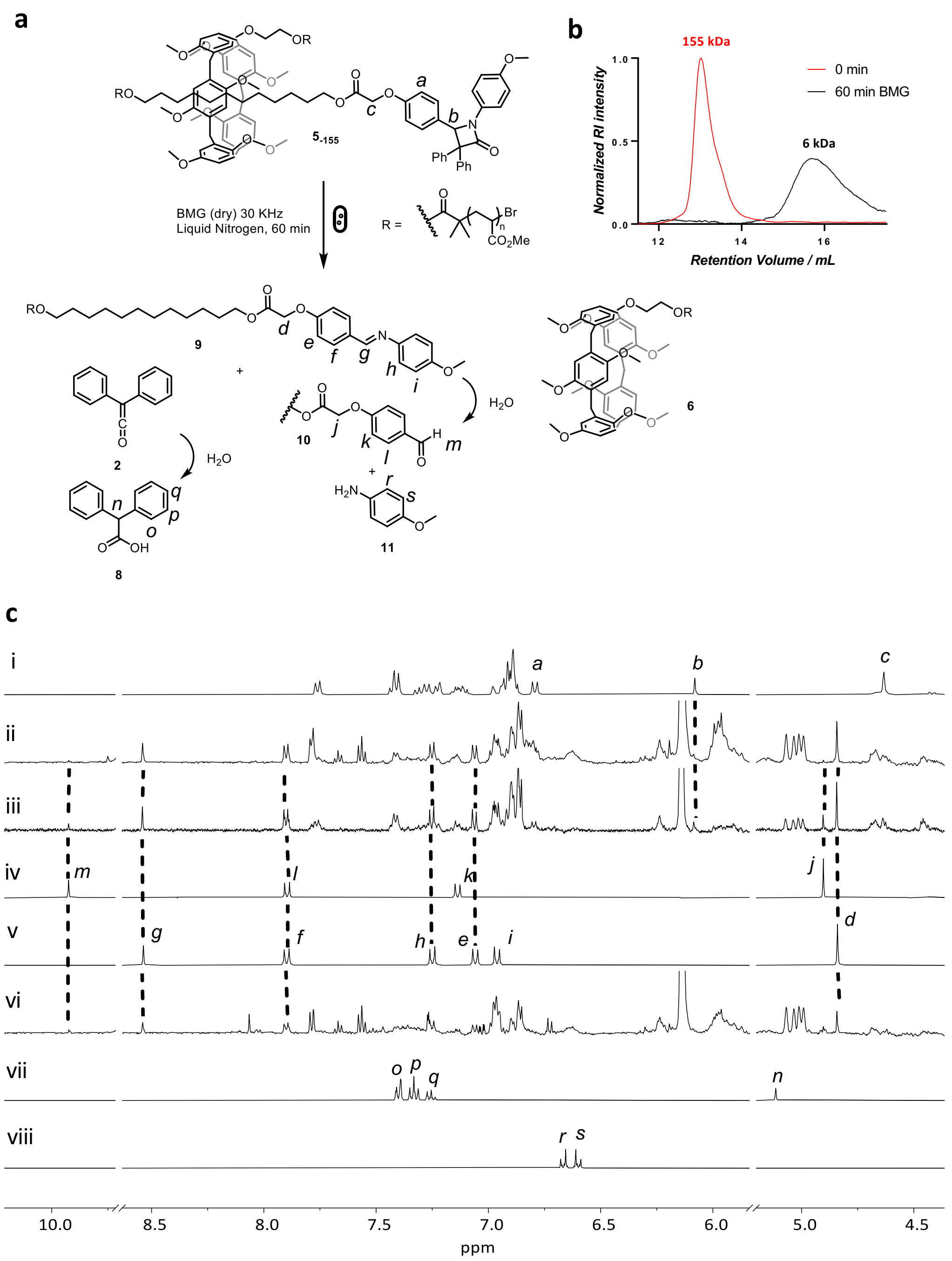

Figure S20. BMG activation (run 1) of polymer **5**-155 in dry condition. BMG activation of polymer **5**-155 affords fragments **10** and **11**, upon hydrolysis of **9**-114, **8**, upon hydrolysis of **2**, and **6** (a). SEC traces of polymer **5**-155 (b) before (red) and after (black) BMG. Partial NMR (500 MHz, Acetone-d_6, 298 K) spectra comparison of (c) polymer **5**-155 before (i), after BMG (ii), and after BMG and MeOH wash (iii), reference polymer **10** (iv), reference polymer **9**-114 (v), concentrated methanol washings (vi), reference compound **8** (vii), and reference compound **11** (viii).

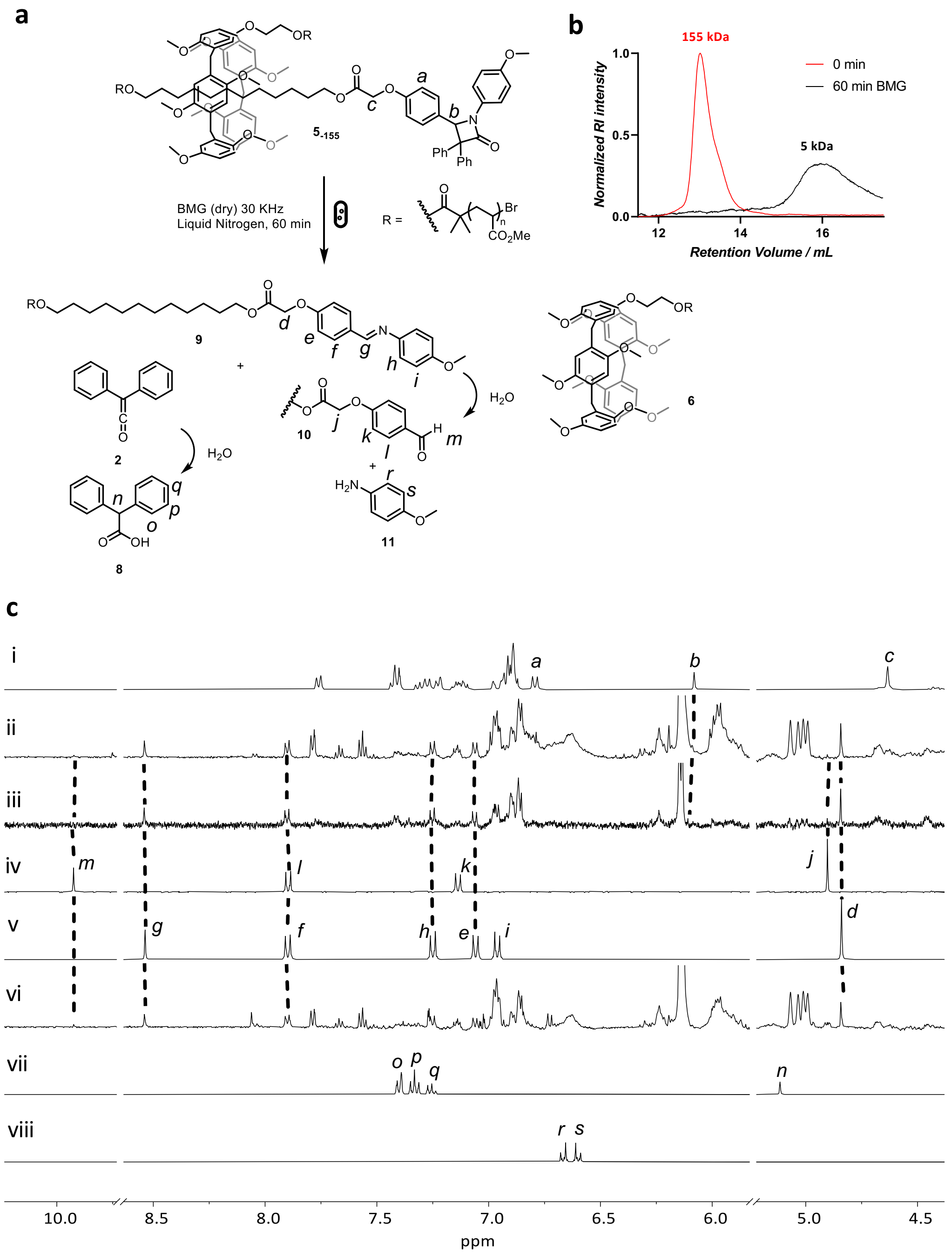

Figure S21. BMG activation (run 2) of polymer **5**$_{-155}$ in dry condition. BMG activation of polymer **5**$_{-155}$ affords fragments **10** and **11**, upon hydrolysis of **9**$_{-114}$, **8**, upon hydrolysis of **2**, and **6** (a). SEC traces of polymer **5**$_{-155}$ (b) before (red) and after (black) BMG. Partial NMR (500 MHz, Acetone-d_6, 298 K) spectra comparison of (c) polymer **5**$_{-155}$ before (i), after BMG (ii), and after BMG and MeOH wash (iii), reference polymer **10** (iv), reference polymer **9**$_{-114}$ (v), concentrated methanol washings (vi), reference compound **8** (vii), and reference compound **11** (viii).

7.3 BMG of Mechanophore Polymer 5_{-155} in Wet Condition

Polymer **5**$_{-155}$ in wet condition were subjected to the general bulk activation methodology described in *Section 7.1*. Analysis of the material post-activation was carried out in an identical manner to that of the sonication of the polymer with same mechanophore, as described in *Section 5.2*.

In BMG under wet conditions, the performance of retro-[2+2] cycloaddition of β-lactam mechanophore is similar to that in the dry conditions. However, since there is enough water in the system, generated imine polymer was fully hydrolysed to aldehyde polymer, and peaks (*o, p*) of diphenylacetic acid were observed in concentrated methanol washings.

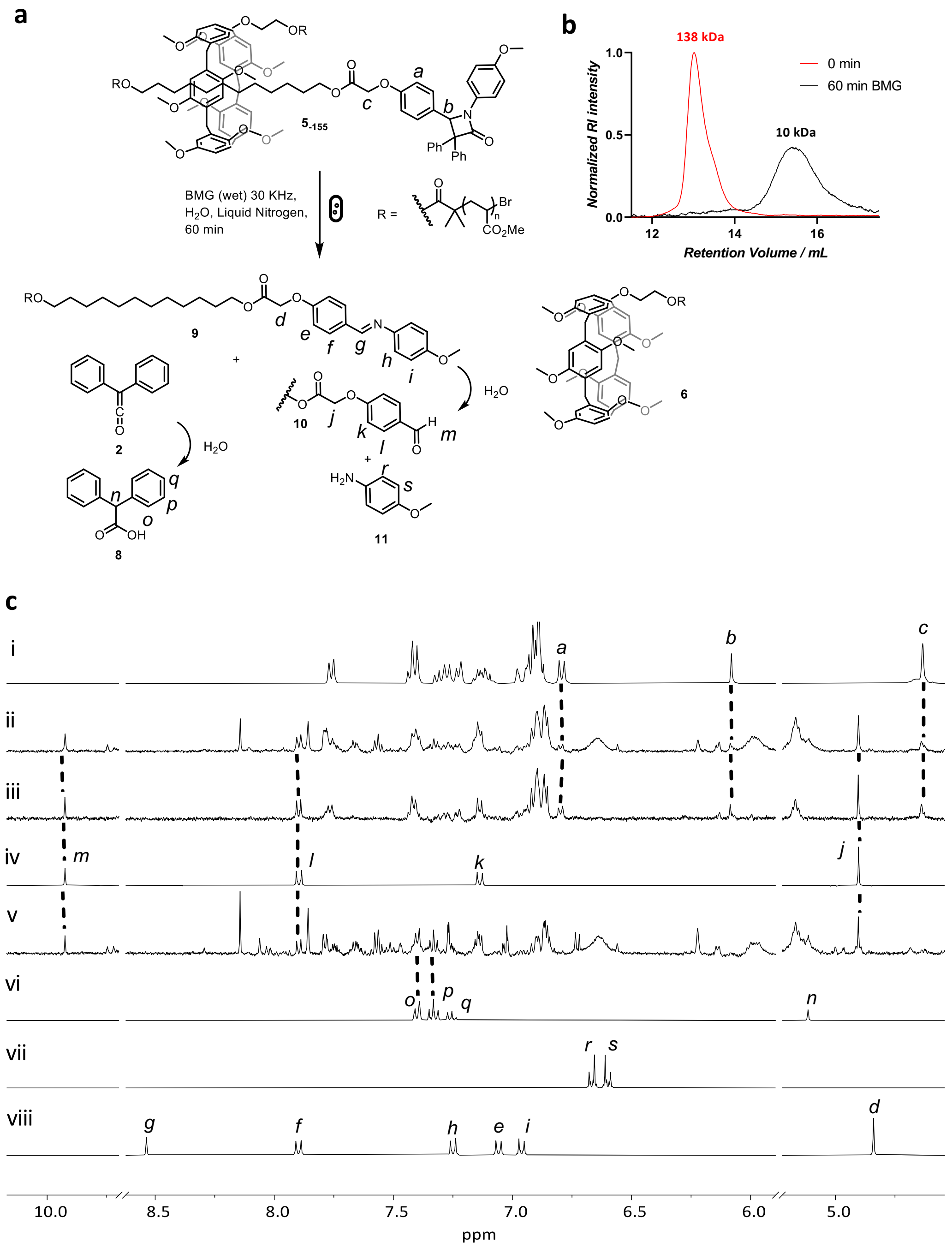

Figure S22. BMG activation (run 1) of polymer **5**$_{-155}$ in wet condition. BMG activation of polymer **5**$_{-155}$ affords fragments **10** and **11**, upon hydrolysis of **9**$_{-114}$, **8**, upon hydrolysis of **2**, and **6** (a). SEC traces of polymer **5**$_{-155}$ (b) before (red) and after (black) BMG. Partial NMR (500 MHz, Acetone-d_6, 298 K) spectra comparison of (c) polymer **5**$_{-155}$ before (i), after BMG (ii), and after BMG and MeOH wash (iii), reference polymer **10** (iv), concentrated methanol washings (v), reference compound **8** (vi), reference compound **11** (vii), and reference polymer **9**$_{-114}$ (viii).

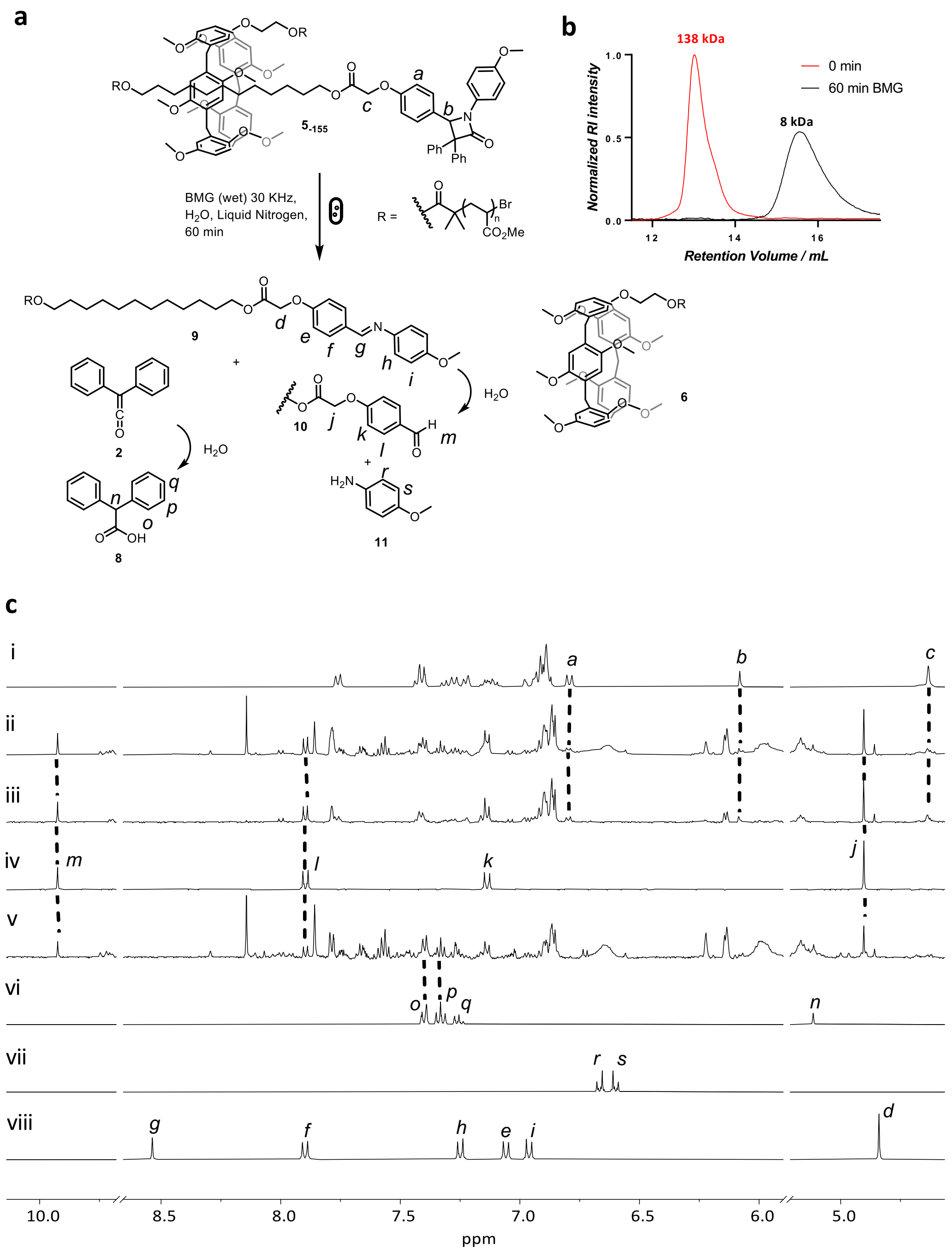

Figure S23. BMG activation (run 2) of polymer **5**$_{\text{-155}}$ in wet condition. BMG activation of polymer **5**$_{\text{-155}}$ affords fragments **10** and **11**, upon hydrolysis of **9**$_{\text{-114}}$, **8**, upon hydrolysis of **2**, and **6** (a). SEC traces of polymer **5**$_{\text{-155}}$ (b) before (red) and after (black) BMG. Partial NMR (500 MHz, Acetone-d_6, 298 K) spectra comparison of (c) polymer **5**$_{\text{-155}}$ before (i), after BMG (ii), and after BMG and MeOH wash (iii), reference polymer **10** (iv), concentrated methanol washings (v), reference compound **8** (vi), reference compound **11** (vii), and reference polymer **9**$_{\text{-114}}$ (viii).

7.4 BMG of Mechanophore Polymer $\mathbf{1}_{HYM}$ in Wet Condition

Polymer $\mathbf{1}_{HYM}$ in wet condition were subjected to the general bulk activation methodology described in *Section 7.1*. Analysis of the material post-activation was carried out in an identical manner to that of the sonication of the polymer with same mechanophore, as described in *Section 5.4*.

Similar to the ultrasounic activation of $\mathbf{1}_{HYM}$ (*Section 5.4*), in BMG under wet conditions, imine polymer was fully hydrolysed to aldehyde polymer after retro-[2+2] cycloaddition of β-lactam mechanophore. The cascade reaction was then triggered to release hymecromone.

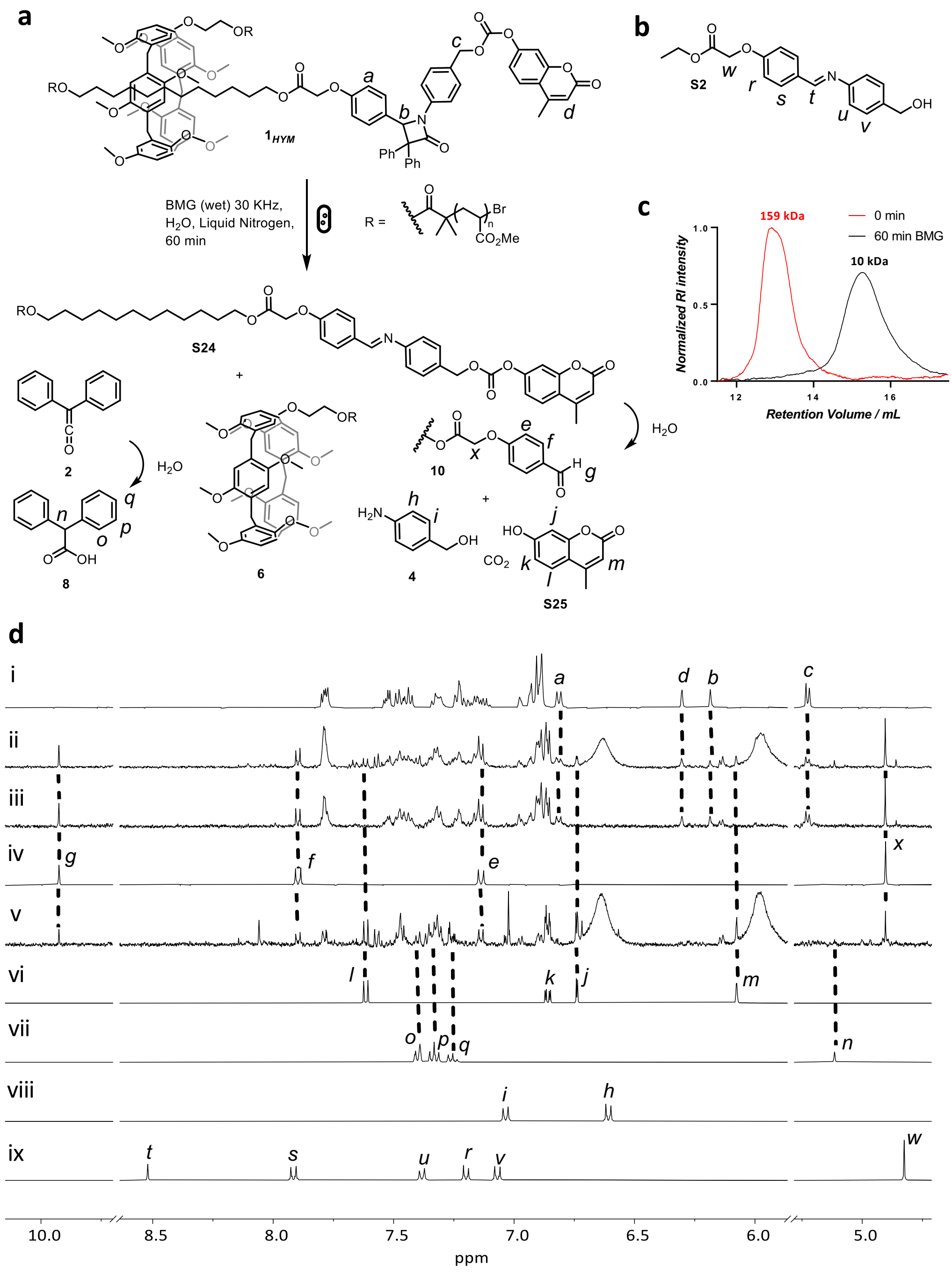

Figure S24. BMG activation (run 1) of polymer $\mathbf{1}_{HYM}$ in wet condition. BMG activation of polymer $\mathbf{1}_{HYM}$ affords fragments **10**, **4**, **S25** and CO_2 from cascade reaction, **8**, upon hydrolysis of **2**, and **6** (a). Reference compound **S2** (b). SEC traces of polymer $\mathbf{1}_{HYM}$ (c) before (red) and after (black) BMG. Partial NMR (500 MHz, Acetone-d_6, 298 K) spectra comparison of (d) polymer $\mathbf{1}_{HYM}$ before (i), after BMG (ii), and after BMG and MeOH wash (iii), reference polymer **10** (iv), concentrated methanol washings (v), reference compound **S25** (vi), reference compound **8** (vii), reference compound **4** (viii), and reference polymer **S2** (ix).

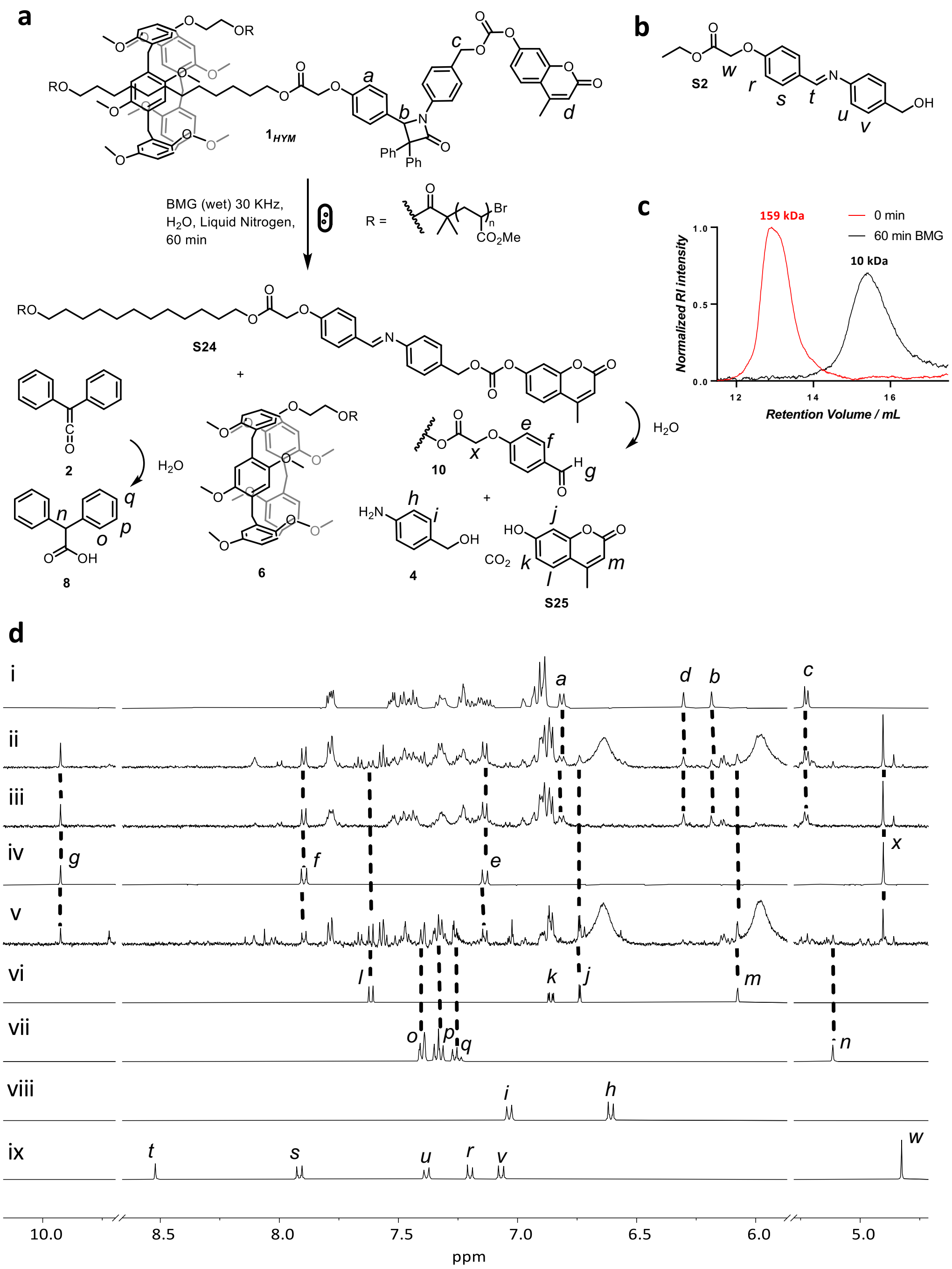

Figure S25. BMG activation (run 2) of polymer $\mathbf{1}_{HYM}$ in wet condition. BMG activation of polymer $\mathbf{1}_{HYM}$ affords fragments **10**, **4**, **S25** and CO_2 from cascade reaction, **8**, upon hydrolysis of **2**, and **6** (a). Reference compound **S2** (b). SEC traces of polymer $\mathbf{1}_{HYM}$ (c) before (red) and after (black) BMG. Partial NMR (500 MHz, Acetone-d_6, 298 K) spectra comparison of (d) polymer $\mathbf{1}_{HYM}$ before (i), after BMG (ii), and after BMG and MeOH wash (iii), reference polymer **10** (iv), concentrated methanol washings (v), reference compound **S25** (vi), reference compound **8** (vii), reference compound **4** (viii), and reference polymer **S2** (ix).

7.5 BMG of Mechanophore Polymer $\mathbf{1}_{GEM}$ in Wet Condition

Polymer $\mathbf{1}_{GEM}$ in wet condition were subjected to the general bulk activation methodology described in *Section 7.1*. Analysis of the material post-activation was carried out in an identical manner to that of the sonication of the polymer with same mechanophore, as described in *Section 5.5*.

Similar to the ultrasounic activation of $\mathbf{1}_{GEM}$ (*Section 5.5*), in BMG under wet conditions, imine polymer was fully hydrolysed to aldehyde polymer after retro-[2+2] cycloaddition of β-lactam mechanophore. The cascade reaction was then triggered to release gemcitabine.

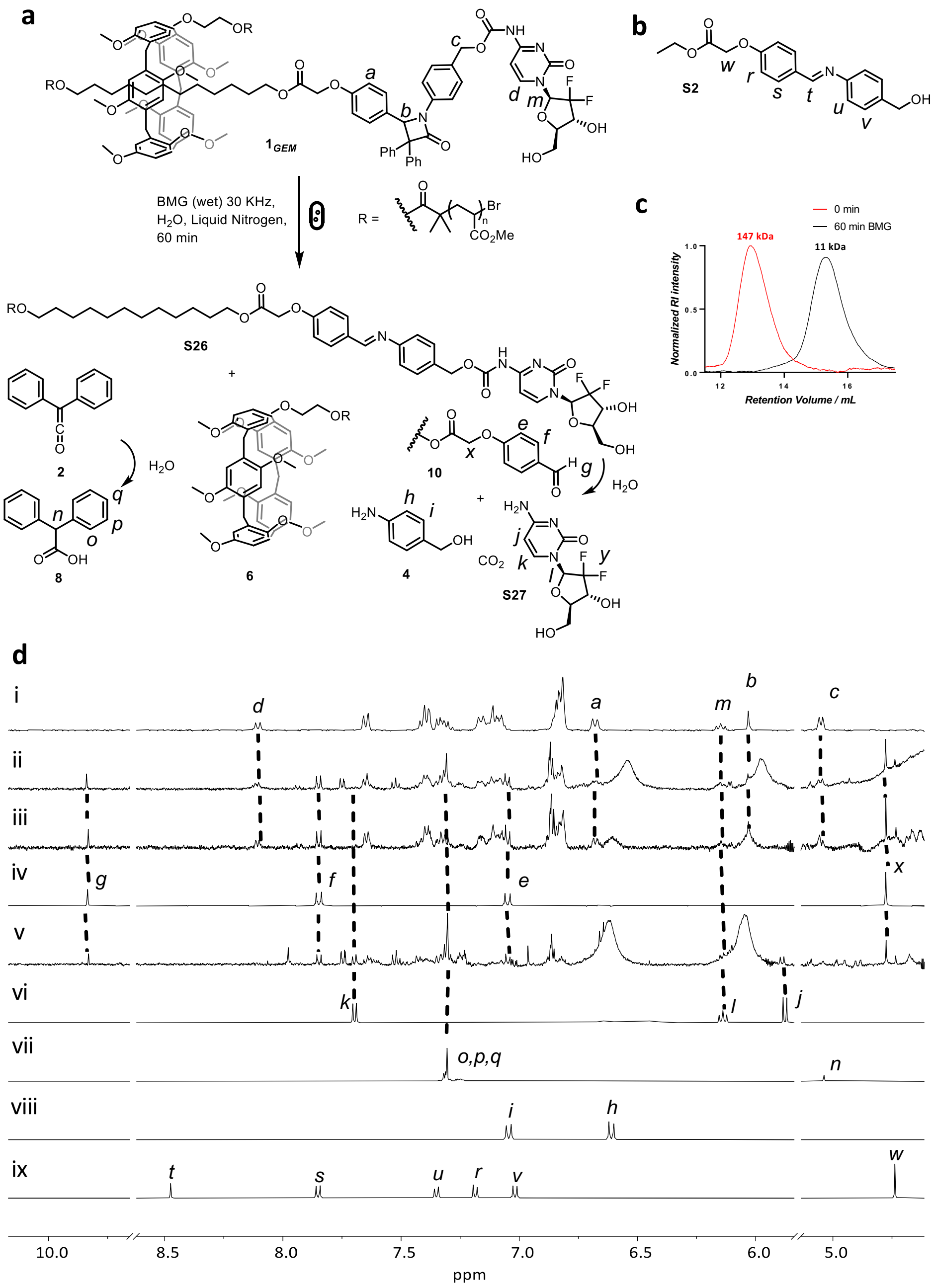

Figure S26. BMG activation (run 1) of polymer **1*GEM*** in wet condition. BMG activation of polymer **1*GEM*** affords fragments **10**, **4**, **S27** and CO_2 from cascade reaction, **8**, upon hydrolysis of **2**, and **6** (a). Reference compound **S2** (b). SEC traces of polymer **1*GEM*** (c) before (red) and after (black) BMG. Partial NMR (500 MHz, Acetonitrile-*d*$_3$/H_2O (9/1), 298 K) spectra comparison of (d) polymer **1*GEM*** before (i), after BMG (ii), and after BMG and MeOH wash (iii), reference polymer **10** (iv), concentrated methanol washings (v), reference compound **S27** (vi), reference compound **8** (vii), reference compound **4** (viii), and reference polymer **S2** (ix).

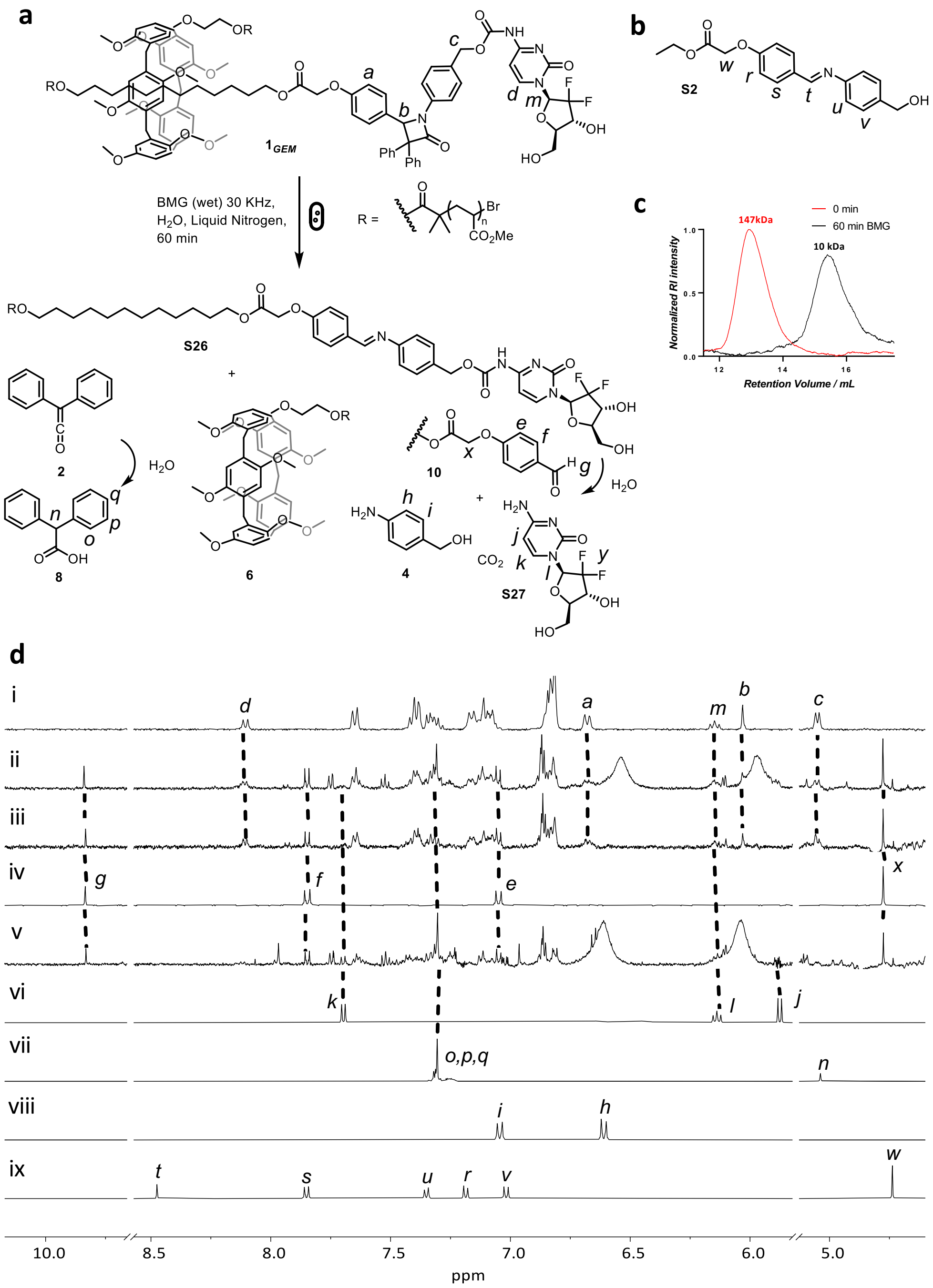

Figure S27. BMG activation (run 2) of polymer **1*GEM*** in wet condition. BMG activation of polymer **1*GEM*** affords fragments **10**, **4**, **S27** and CO_2 from cascade reaction, **8**, upon hydrolysis of **2**, and **6** (a). Reference compound **S2** (b). SEC traces of polymer **1*GEM*** (c) before (red) and after (black) BMG. Partial NMR (500 MHz, Acetonitrile-*d₃*/H_2O (9/1), 298 K) spectra comparison of (d) polymer **1*GEM*** before (i), after BMG (ii), and after BMG and MeOH wash (iii), reference polymer **10** (iv), concentrated methanol washings (v), reference compound **S27** (vi), reference compound **8** (vii), reference compound **4** (viii), and reference polymer **S2** (ix).

7.6 BMG of Mechanophore Polymer S20 in Dry Condition

Polymer **S20** in dry condition were subjected to the general bulk activation methodology described in *Section 7.1*. Analysis of the material post-activation was carried out to confirm the stability of β-lactam mechanophore during the BMG in dry condition.

Comparison of the ^1H NMR spectra of polymer **S20** before and after BMG and reference compounds showed a small amount of mechanophore (less then 6 %) underwent retro-[2+2] cycloaddition. Although the polymer was pretreated by drying under vacuum, generated imine polymer was still fully hydrolysed to aldehyde polymer due to adventitious water in the experimental setup.

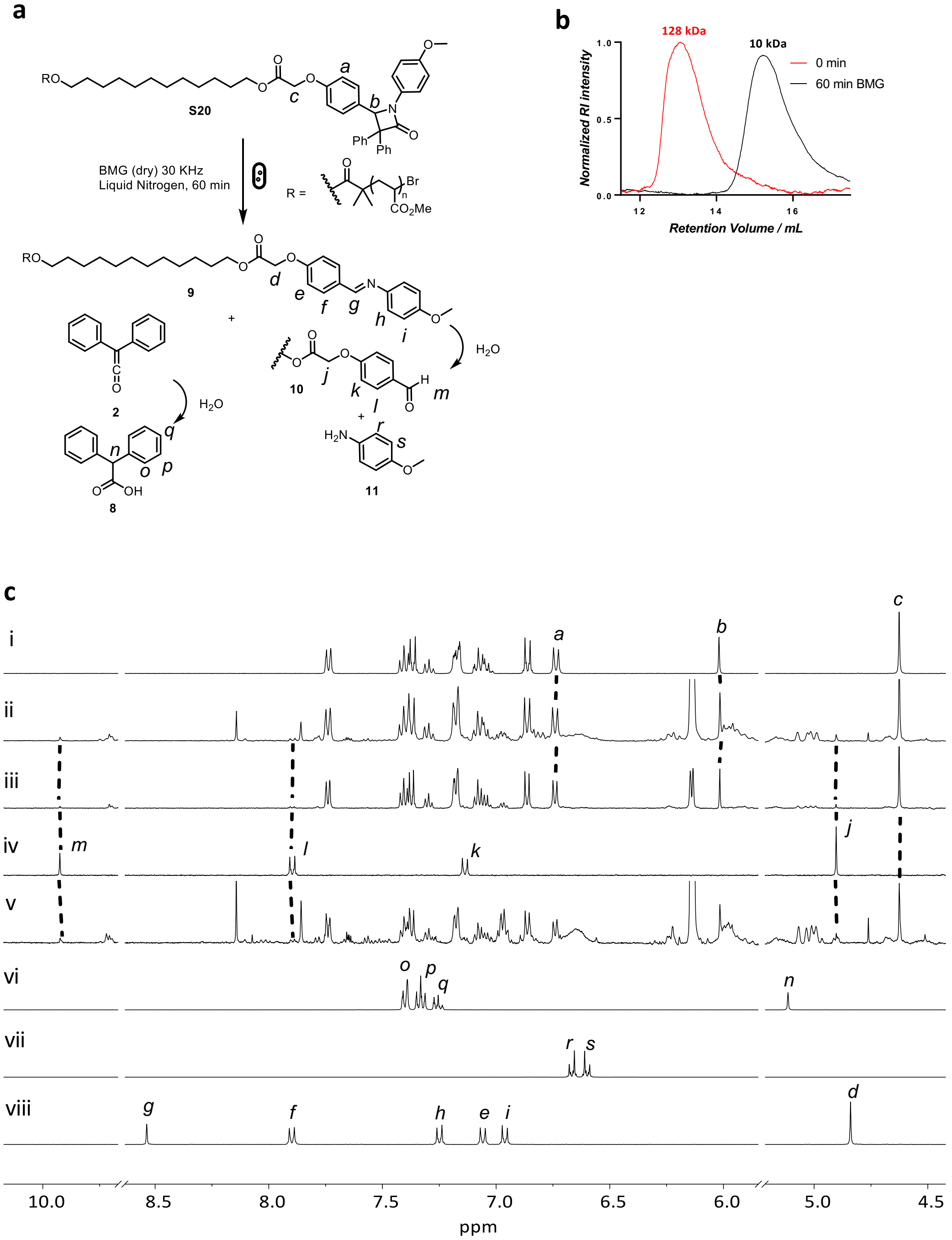

Figure S28. BMG activation of polymer **S20** in dry condition. BMG activation of polymer **S20** affords fragments **10** and **11**, upon hydrolysis of **9**$_{\text{-114}}$, and **8**, upon hydrolysis of **2** (a). SEC traces of polymer **S20** (b) before (red) and after (black) BMG. Partial NMR (500 MHz, Acetone-d_6, 298 K) spectra comparison of (c) polymer **S20** before (i), after BMG (ii), and after BMG and MeOH wash (iii), reference polymer **10** (iv), concentrated methanol washings (v), reference compound **8** (vi), reference compound **11** (vii), and reference polymer **9**$_{\text{-114}}$ (viii).

7.7 BMG of Mechanophore Polymer S20 in Wet Condition

Polymer **S20** in wet condition were subjected to the general bulk activation methodology described in *Section 7.1*. Analysis of the material post-activation was carried out to confirm the stability of β-lactam mechanophore during the BMG in wet condition.

BMG under wet conditions of polymer **S20** is almost the same as that with the dry condition (*Section 7.6*). A small amount of mechanophore (less then 6 %) underwent retro-[2+2] cycloaddition, and generated imine polymer was fully hydrolysed to aldehyde polymer.

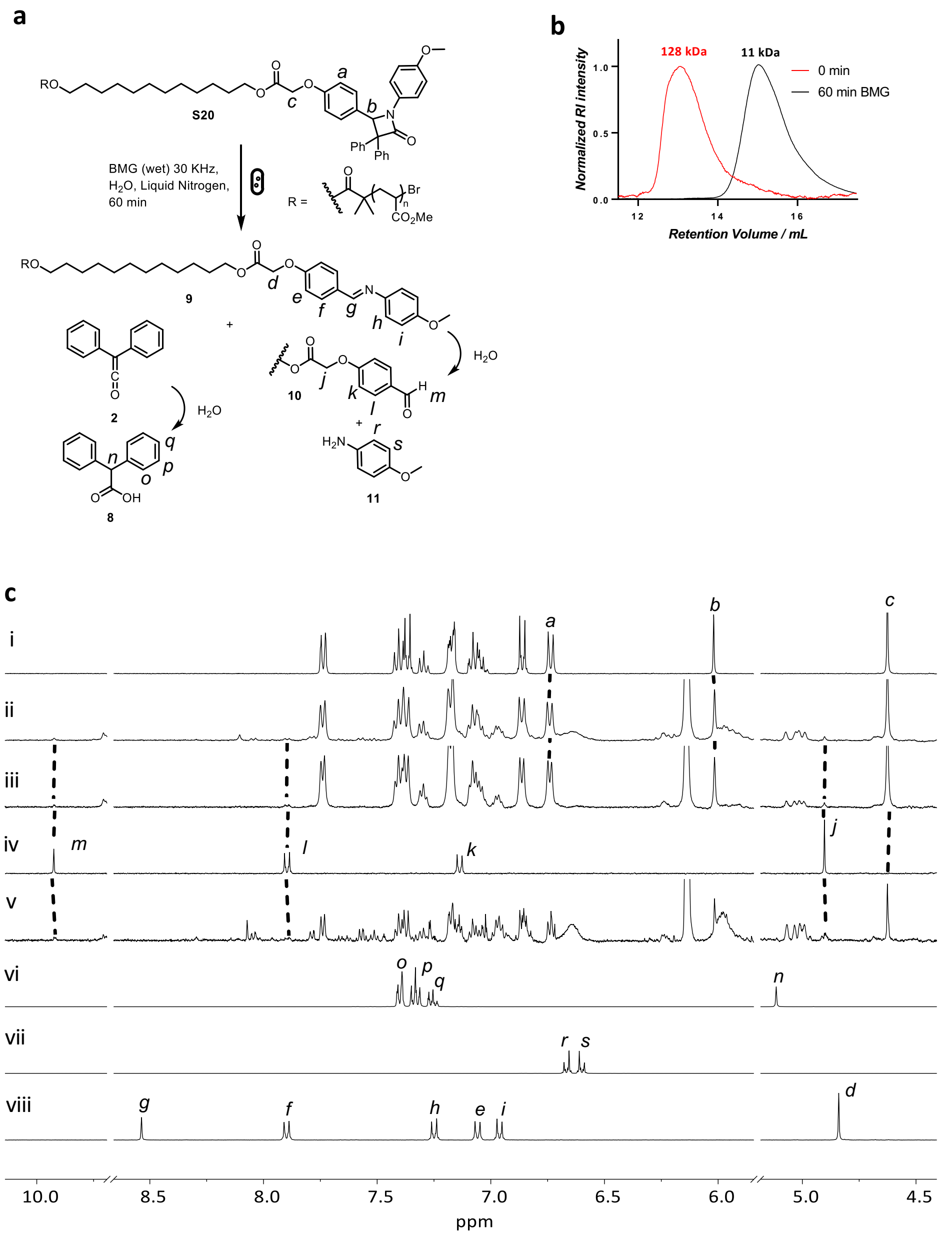

Figure S29. BMG activation of polymer **S20** in wet condition. BMG activation of polymer **S20** affords fragments **10** and **11**, upon hydrolysis of **9**$_{\text{-114}}$, and **8**, upon hydrolysis of **2** (a). SEC traces of polymer **S20** (b) before (red) and after (black) BMG. Partial NMR (500 MHz, Acetone-*d*$_6$, 298 K) spectra comparison of (c) polymer **S20** before (i), after BMG (ii), and after BMG and MeOH wash (iii), reference polymer **10** (iv), concentrated methanol washings (v), reference compound **8** (vi), reference compound **11** (vii), and reference polymer **9**$_{\text{-114}}$ (viii).

7.8 BMG of Mechanophore Polymer S21 under Wet Conditions

Polymer **S21** under wet conditions were subjected to the general bulk activation methodology described in *Section 7.1*. Analysis of the material post-activation was carried out confirm the stability of β-lactam mechanophore and carbonate structure during the BMG under wet conditions.

The BMG result of polymer **S21** is similar to **S20** (*Section 0*). Small amount of mechanophore (less then 6 %) went through retro-[2+2] cycloaddition reaction, and then hydrolysis of imine polymer triggered the cascade reaction to release small amount of hymecromone confirmed by tiny peaks (*l, m*) of hymecromone in concentrated methanol washings.

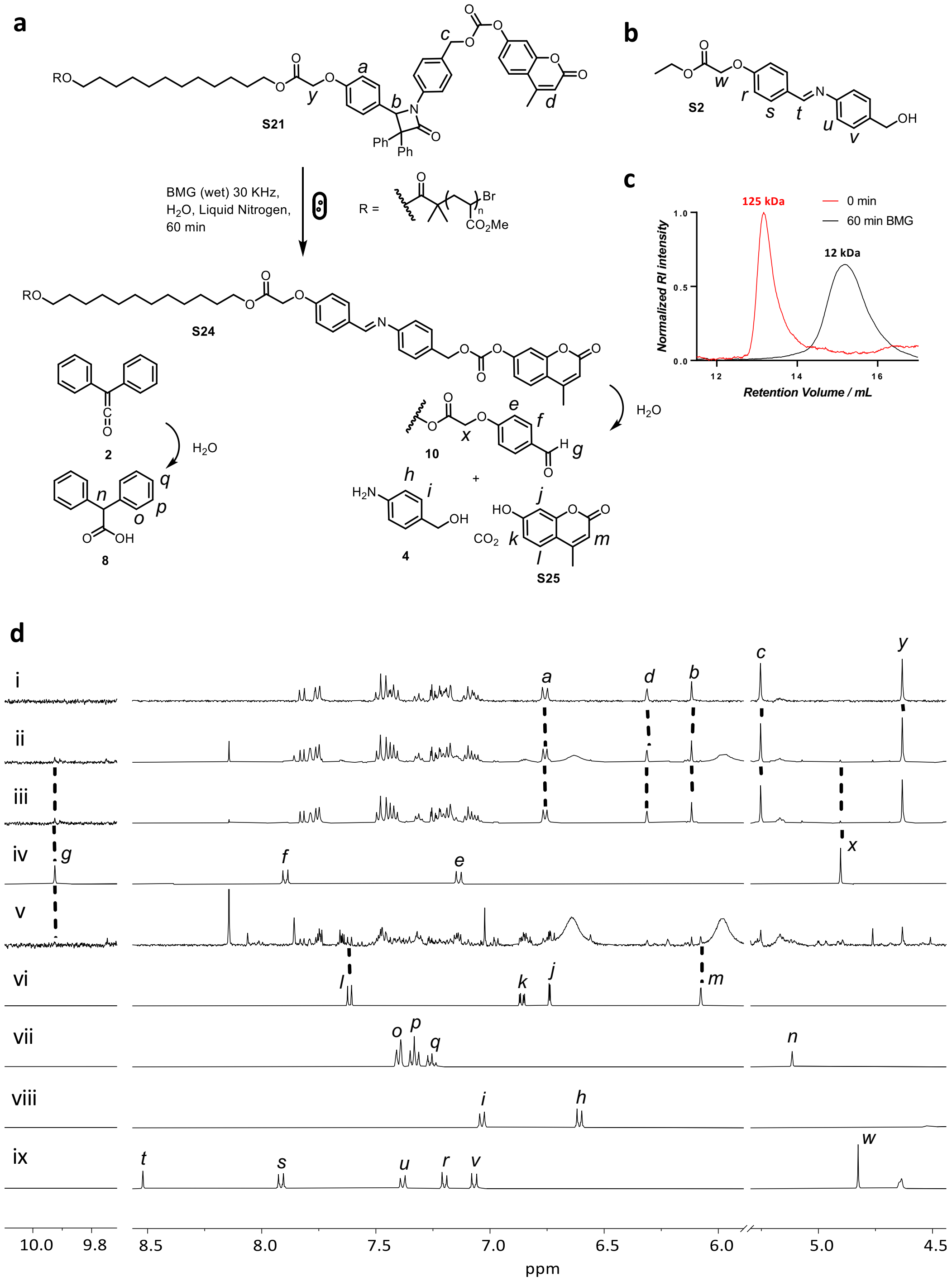

Figure S30. BMG activation of polymer **S21** in wet condition. BMG activation of polymer **S21** affords fragments **10**, **4**, **S25** and CO_2 from cascade reaction, and **8**, upon hydrolysis of **2** (a). Reference compound **S2** (b). SEC traces of polymer **S21** (c) before (red) and after (black) BMG. Partial NMR (500 MHz, Acetone-*d*$_6$, 298 K) spectra comparison of (c) polymer **S21** before (i), after BMG (ii), and after BMG and MeOH wash (iii), reference polymer **10** (iv), concentrated methanol washings (v), reference compound **S25** (vi), reference compound **8** (vii), reference compound **4** (viii), and reference polymer **S2** (ix).

7.9 BMG of Mechanophore Polymer 9_{-114} under Dry Conditions

Polymer **9_{-114}** under dry conditions were subjected to the general bulk activation methodology described in *Section 7.1*. Analysis of the material post-activation was carried out to confirm the stability of imine polymer during the BMG under dry conditions.

Comparison of the ^{1}H NMR spectra of polymer **9_{-114}** before and after BMG and reference compounds showed 10% of imine polymer was hydrolysed after BMG and incubation due to adventitious water in the experimental setup.

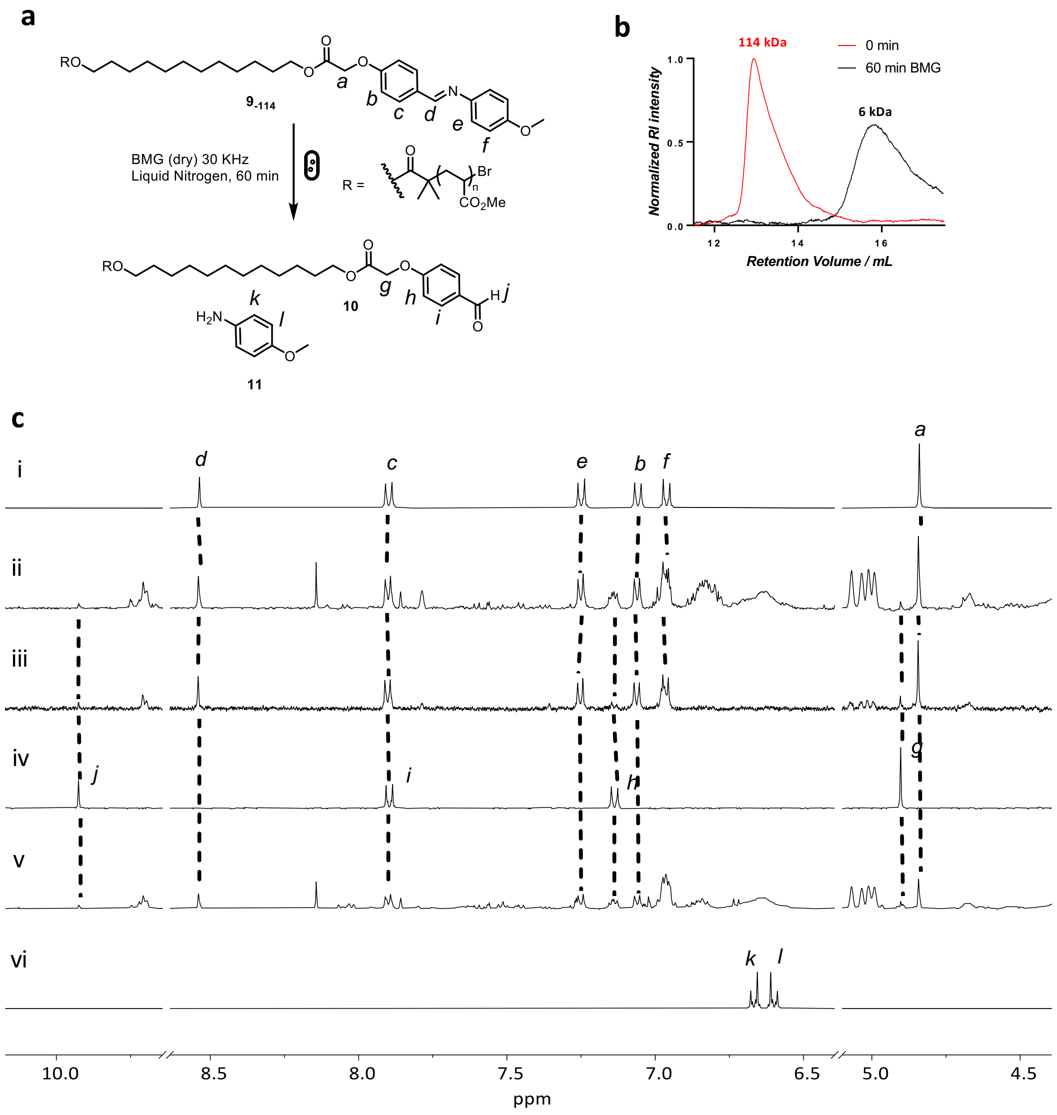

Figure S31. BMG activation of polymer **9_{-114}** in dry condition. BMG activation of polymer **9_{-114}** affords fragments **10** and **11** (a). SEC traces of polymer **9_{-114}** (b) before (red) and after (black) BMG. Partial ^{1}H NMR (500 MHz, Acetone-d_6, 298 K) spectra comparison of (c) polymer **9_{-114}** before (i), after BMG (ii), and after BMG and MeOH wash (iii), reference polymer **10** (iv), concentrated methanol washings (v), and reference compound **11** (vi).

7.10 BMG of Mechanophore Polymer 9_{-114} in Wet Condition

Polymer **9**$_{-114}$ in wet condition were subjected to the general bulk activation methodology described in *Section 7.1*. Analysis of the material post-activation was carried out to confirm the stability of imine polymer during the BMG in wet condition.

The BMG result showed polymer **9**$_{-114}$ was fully hydrolysed to aldehyde polymer after BMG and incubation in wet condition.

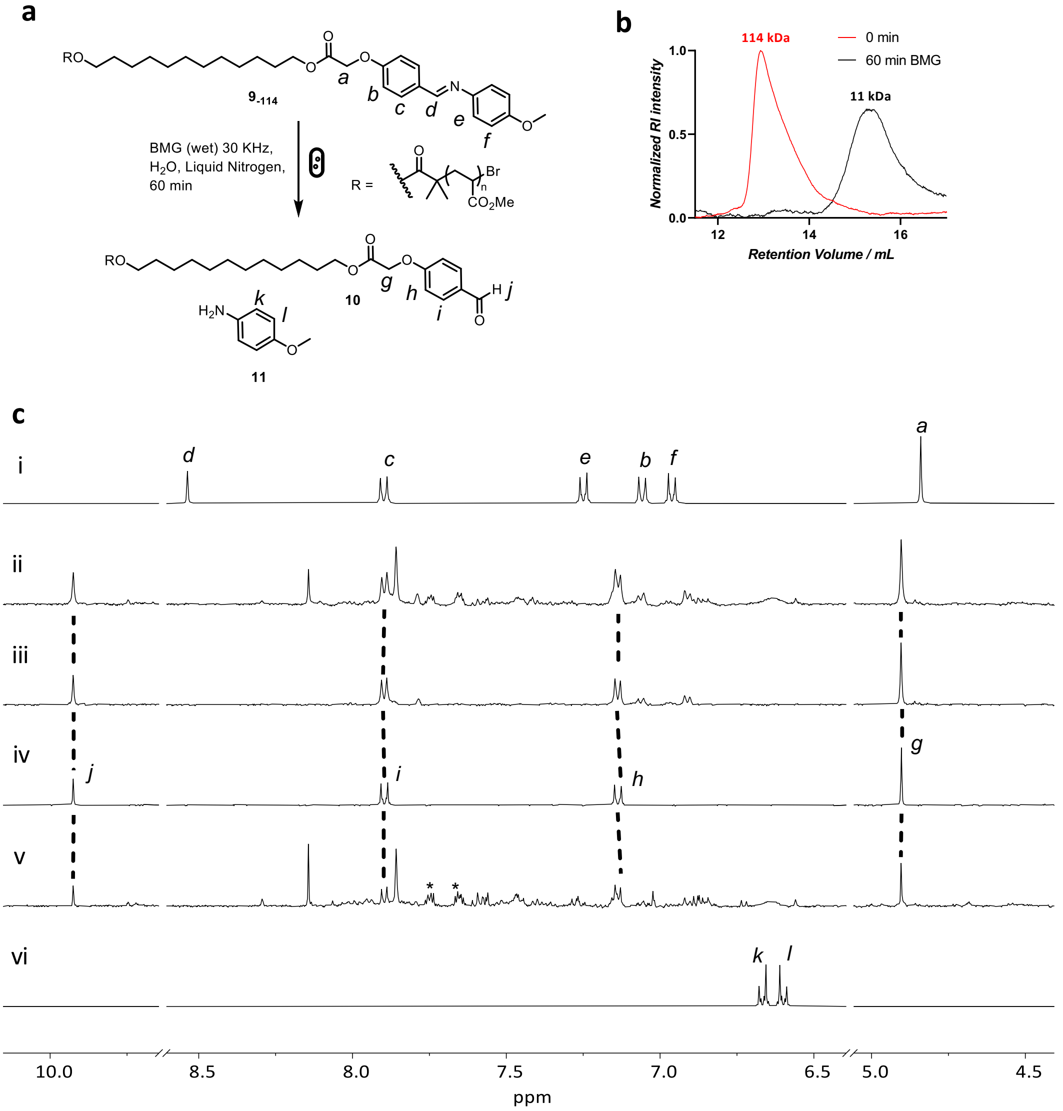

Figure S32. BMG activation of polymer **9**$_{-114}$ in wet condition. BMG activation of polymer **9**$_{-114}$ affords fragments **10** and **11** (a). SEC traces of polymer **9**$_{-114}$ (b) before (red) and after (black) BMG. Partial ^{1}H NMR (500 MHz, Acetone-d_6, 298 K) spectra comparison of (c) polymer **9**$_{-114}$ before (i), after BMG (ii), and after BMG and MeOH wash (iii), reference polymer **10** (iv), concentrated methanol washings (v), and reference compound **11** (vi). * Peaks marked with stars are external impurities. Details see *Section 5.8*.

7.11 Stability of compounds 4 and 11 in BMG in Wet Condition

Compound **4** or **11** was mixed with compound **8** and PMA was subjected to the general bulk activation in wet condition described in *Section 7.1*. Analysis of the material post-activation was carried out to confirm the stability of compound **4** or **11** during milling in these conditions.

The results show that compounds **4** and **11** were fully or mostly decomposed respectively, which explains the fact that we could not see these two compounds in the bulk activation experiment.

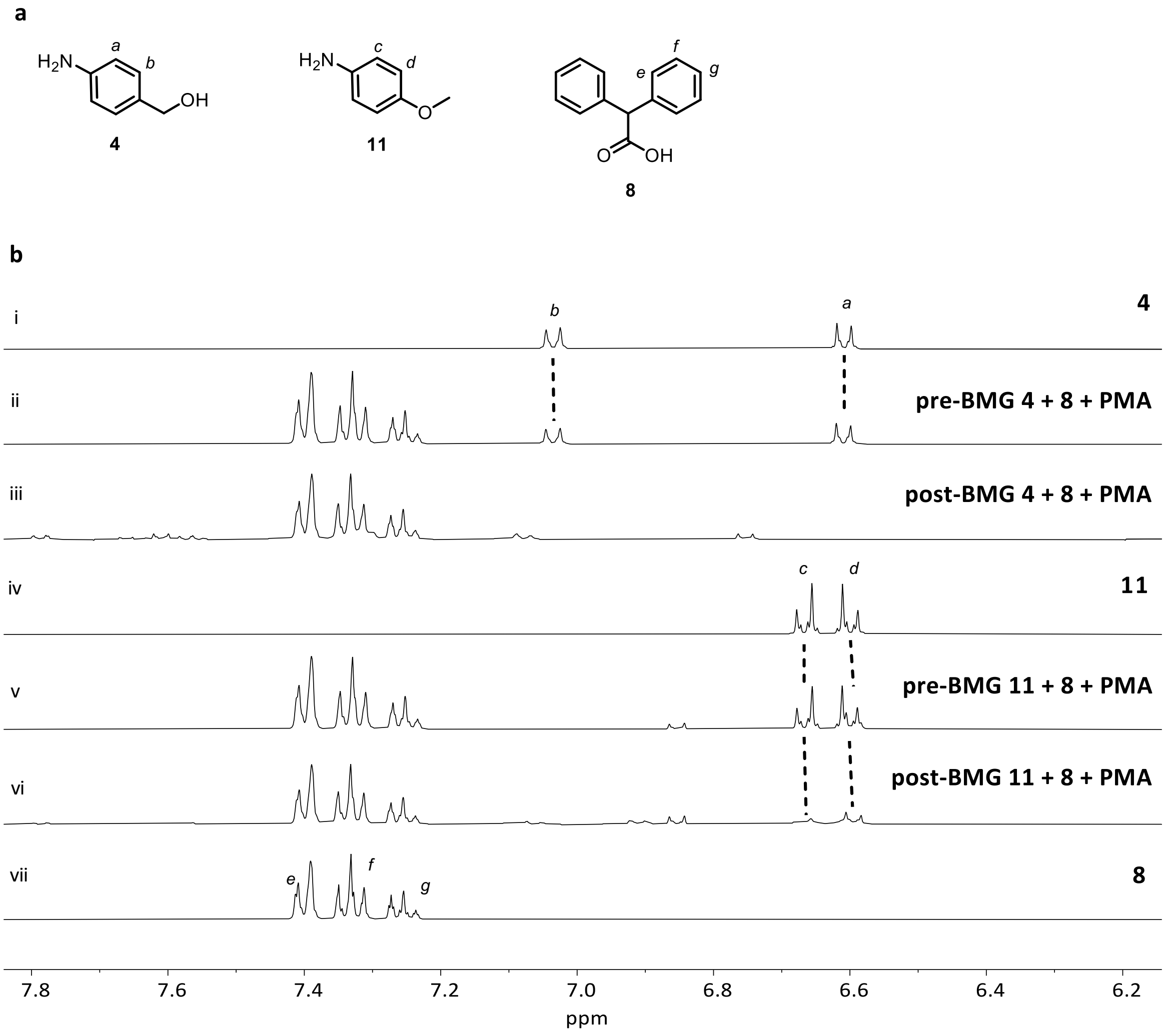

Figure S33. BMG activation of compounds **4** or **11** mixed with compound **8** and plain PMA in wet condition. Structure of compounds **4**, **11** and **8** (a). Partial ^{1}H NMR (400 MHz, Acetone-d_6, 298 K) spectra comparison of (b) reference compound **4** (i), pre-BMG (ii) and post-BMG (iii) of compound **4** mixed with **8** and plain PMA, reference compound **11** (iv), pre-BMG (v) and post-BMG (vi) of compound **11** mixed with **8** and plain PMA, and reference compound **8** (vii).

8 Calculation of Extent of Mechanophore Activation

8.1 Calculations for Polymer 5_{-138} in Sonication Experiments

Sonication (run 1) of polymer **5-138** is used as an example to show of how the extent of mechanophore activation is calculated for polymer **5-138** in sonication experiments.

a

b

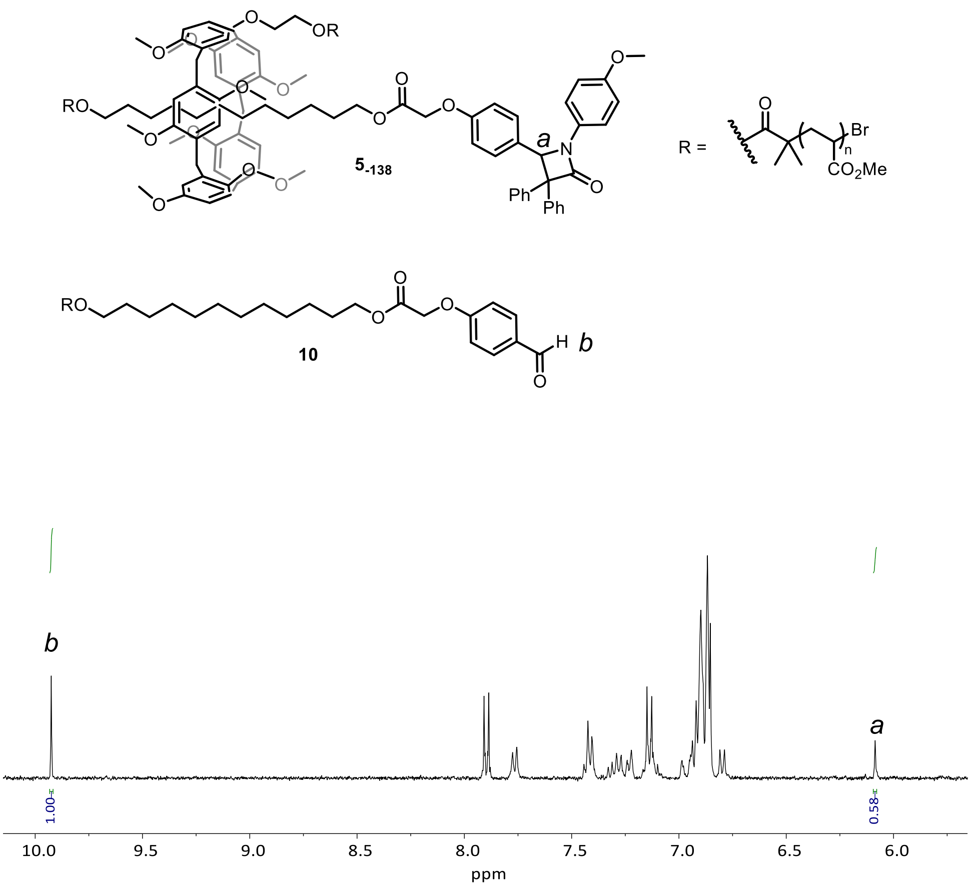

Figure S34. Polymer **5-138** affords polymer fragment **10** after activation (a). And, partial ^{1}H NMR (500 MHz, Acetone-d_6, 298 K) spectra of post-sonication polymer **5-138** after being washed with methanol (b).

Here, we use values determined from integration of peaks a (I_a) and b (I_b) in the ^{1}H NMR spectra of post-sonication polymer **5-138** after being washed with methanol.

The extent of retro-[2+2] cycloaddition (C_r) having occurred during the sonication was determined by the formula below:

$$C_r = \frac{I_b}{I_a + I_b} \times 100\%$$

In this case, $\boldsymbol{C_r}$ **= 63%** [(1.00 / (0.58 + 1.00)) × 100%].

8.2 Calculations for Polymer 5_{-155} in Sonication Experiments in the Absence of Water

Here we used sonication (run 1) of polymer **5_{-155}** as an example to to show of how the extent of mechanophore activation is calculated for polymer **5_{-155}** in sonication experiments with absence of water.

a

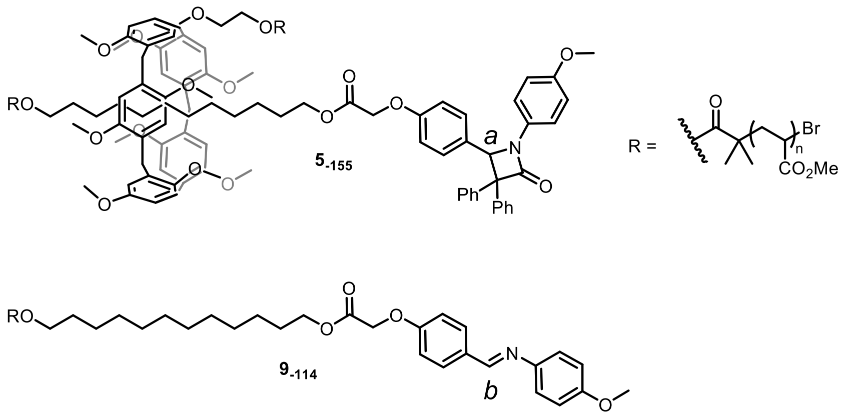

b

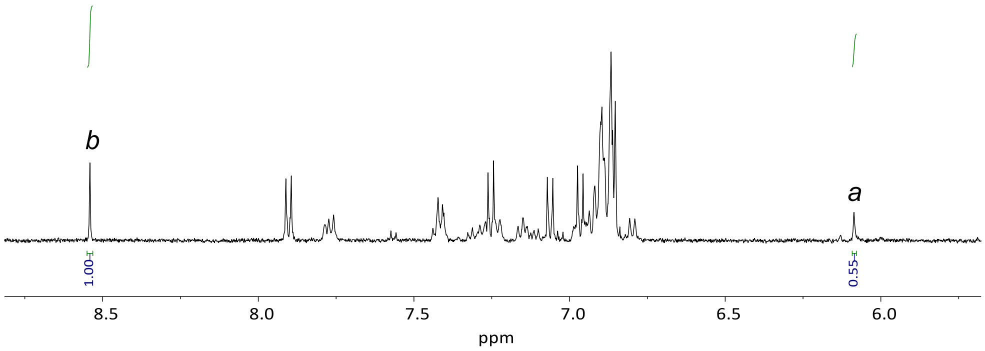

Figure S35. Polymer **5_{-155}** affords polymer fragment **9_{-114}** after activation (a). And, partial ^{1}H NMR (500 MHz, Acetone-d_6, 298 K) spectra of post-sonication polymer **5_{-155}** after being washed with methanol (b).

Here, we use values determined from integration of peaks a (I_a) and b (I_b) in the ^{1}H NMR spectra of post-sonication polymer **5_{-155}** after being washed with methanol.
The extent of retro-[2+2] cycloaddition (C_r) having occurred during the sonication was determined by the formula below:

$$C_r = \frac{I_b}{I_a + I_b} \times 100\%$$

In this case, **C_r = 65%** [(1.00 / (0.55 + 1.00)) × 100%].

8.3 Calculations for Polymer 5_{-138} in Compression Experiments, Polymer 5_{-155} in BMG Experiments, and Polymers S20, 9_{-112}, 9_{-114} in all types of activation

Here we used compression of polymer **5_{-138}** as an example to show of how the extent of mechanophore activation and hydrolysis of imine is calculated for polymer **5_{-138}** in compression experiments, polymer **5_{-155}** in BMG experiments, and polymers **S20**, **9_{-112}**, **9_{-114}** in all types of activation.

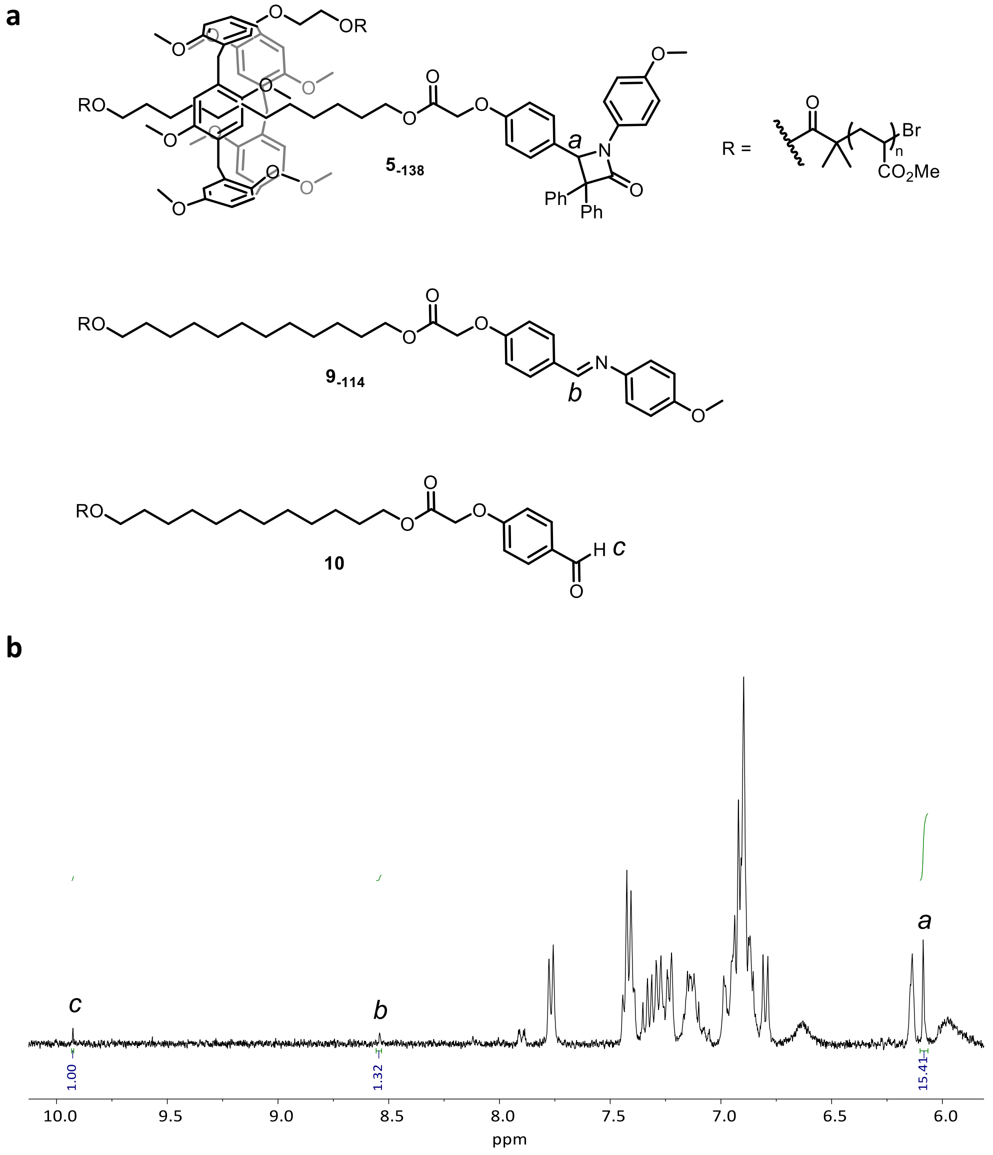

Figure S36. Polymer **5_{-138}** affords polymer fragments **9_{-114}** and **10** after activation (a). And, partial ^{1}H NMR (500 MHz, Acetone-d_6, 298 K) spectra of post-sonication polymer **5_{-138}** before being washed with methanol (b).

Here, we use values determined from integration of peaks a (I_a), b (I_b) and c (I_c) in the ^{1}H NMR spectra of post-sonication polymer **5_{-138}** before being washed with methanol.

The extent of retro-[2+2] cycloaddition (C_r), hydrolysis of imine (C_h) and survived imine (C_i) having occurred during the activation were determined by the formula below:

$$C_r = \frac{I_b + I_c}{I_a + I_b + I_c} \times 100\%$$

$$C_h = \frac{I_c}{I_a + I_b + I_c} \times 100\%$$

$$C_i = \frac{I_b}{I_a + I_b + I_c} \times 100\%$$

In this case, $\boldsymbol{C_r}$ **= 13%** [(1.32 + 1.00) / (15.41 + 1.32 + 1.00) × 100%], $\boldsymbol{C_h}$ **= 6%** [1.00 / (15.41 + 1.32 + 1.00) × 100%], and $\boldsymbol{C_i}$ **= 7%** [1.32 / (15.41 + 1.32 + 1.00) × 100%].

8.4 Calculations for Polymers $\mathbf{1}_{HYM}$ and S21

Here we used sonication (run 1) of polymer $\mathbf{1}_{HYM}$ as an example to show of how the extent of mechanophore activation and functional molecule release via cascade reaction is calculated for polymers $\mathbf{1}_{HYM}$ and **S21** after activation.

a

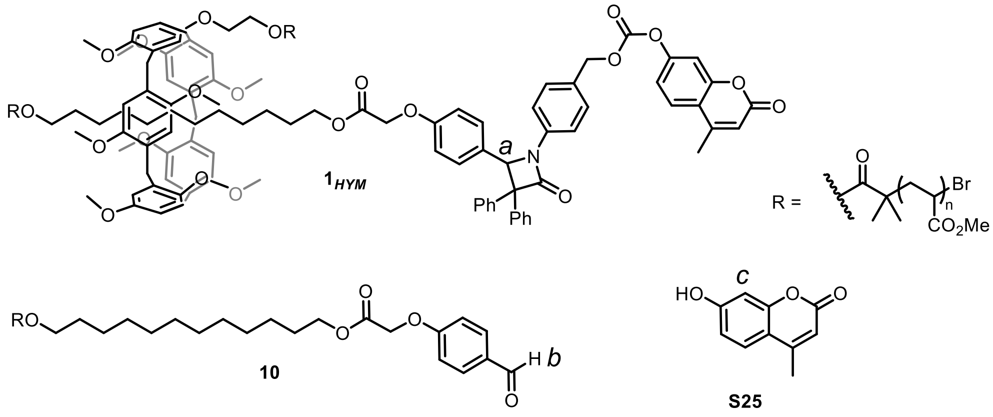

b

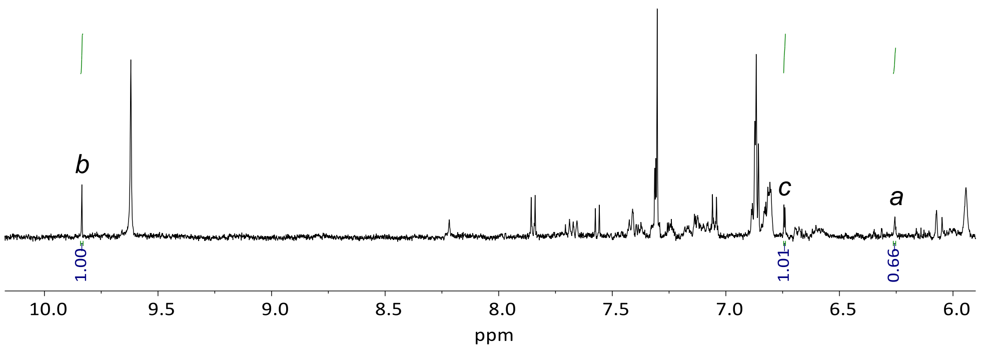

Figure S37. Polymer $\mathbf{1}_{HYM}$ affords fragments **10** and **S25** after activation (a). And, partial ^{1}H NMR (500 MHz, Acetonitrile-d_6/H_2O (9/1), 298 K) spectra of post-sonication polymer $\mathbf{1}_{HYM}$ before being washed with methanol (b).

Here, we use values determined from integration of peaks a (I_a), b (I_b) and c (I_c) in the ^{1}H NMR spectra

of post-sonication polymer **1*HYM*** before being washed with methanol.
The percentage of retro-[2+2] cycloaddition (C_r) and released hymecromone (C_{hym}) having occurred during the activation were determined by the formula below:

$$C_r = \frac{I_b}{I_a + I_b} \times 100\%$$

$$C_{hym} = \frac{I_c}{I_a + I_c} \times 100\%$$

In this case, $\boldsymbol{C_r}$ **= 60%** [1.00 / (0.66 + 1.00) × 100%], and $\boldsymbol{C_{hym}}$ **= 60%** [1.01 / (0.66 + 1.01) × 100%].

8.5 Calculations for Polymer 1*GEM* in the Sonication

Here we used sonication (run 1) of polymer **1*GEM*** as an example to show of how the extent of mechanophore activation and functional molecule release via cascade reaction is calculated for polymer **1*GEM*** in the sonication.

a

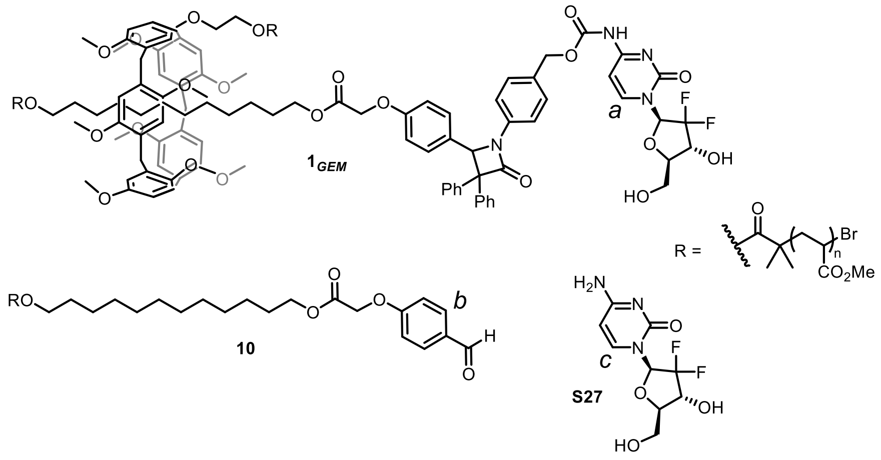

b

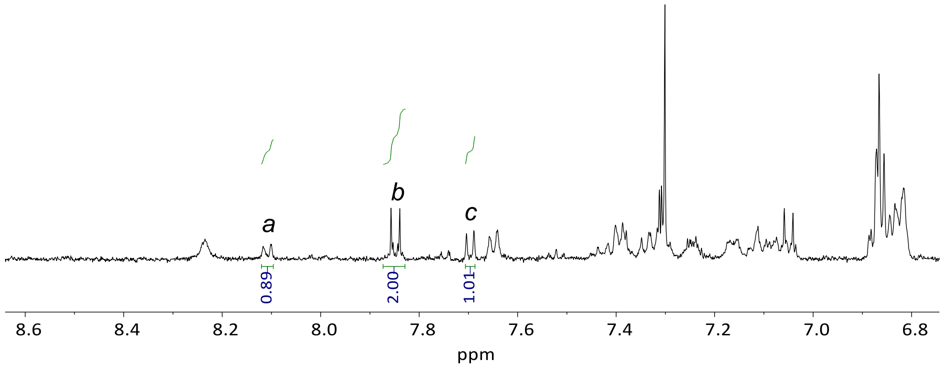

Figure S38. Polymer **1*GEM*** affords fragments **10** and **S27** after activation (a). And, partial ^{1}H NMR (500 MHz, Acetonitrile-d_6/H_2O (9/1), 298 K) spectra of post-sonication polymer **1*GEM*** before being washed with methanol (b).

Here, we use values determined from integration of peaks a (I_a), b (I_b) and c (I_c) in the ^{1}H NMR spectra of post-sonication polymer **1*GEM*** before being washed with methanol.
The pencentage of retro-[2+2] cycloaddition (C_r) and released gemcitabine (C_{gem}) having occurred during the activation were determined by the formula below:

$$C_r = \frac{I_b}{2I_a + I_b} \times 100\%$$

$$C_{gem} = \frac{I_c}{I_a + I_c} \times 100\%$$

In this case, $\boldsymbol{C_r}$ **= 53%** [2.00 / (2 × 0.89 + 2.00) × 100%], and $\boldsymbol{C_{gem}}$ **= 53%** [1.01 / (0.89 + 1.01) × 100%].

8.6 Calculations for Polymer 1*GEM* in the BMG

Here we used BMG (run 1) of polymer **1*GEM*** as an example to show of how the extent of mechanophore activation and functional molecule release via cascade reaction is calculated for polymer **1*GEM*** in the BMG.

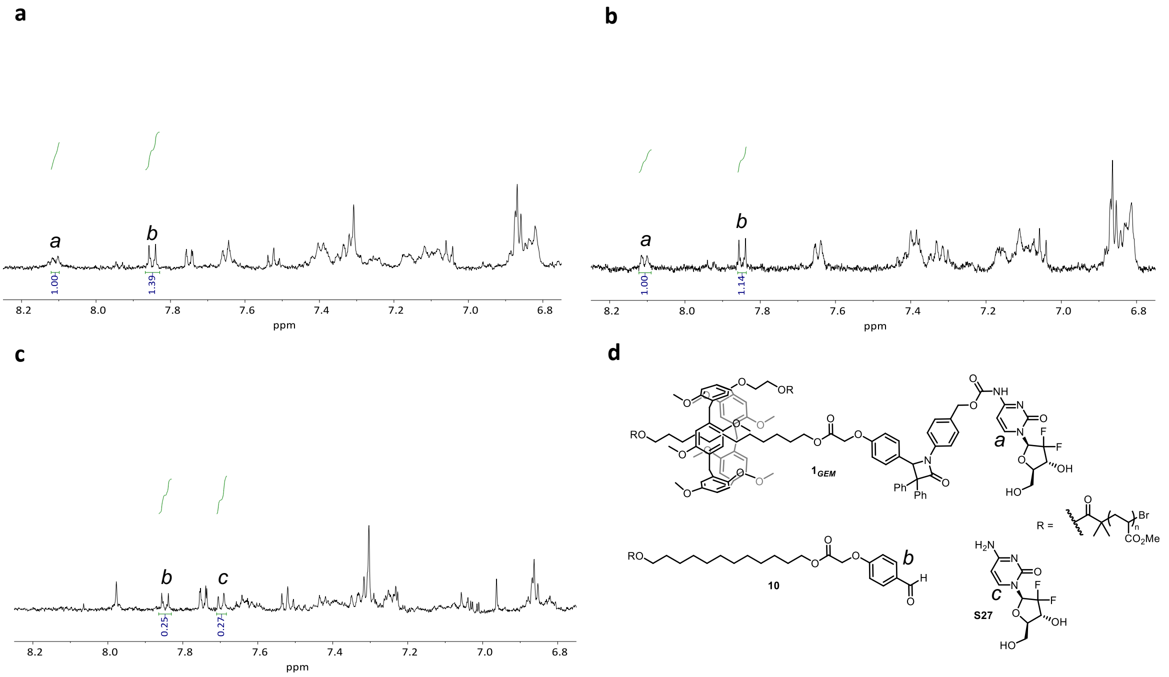

Figure S39. Partial ^{1}H NMR (500 MHz, Acetonitrile-d_6/H_2O (9/1), 298 K) spectra of post-BMG polymer **1*GEM*** before being washed with methanol (a), post-BMG polymer **1*GEM*** after being washed with methanol (b), and the concentrated methanol washings (c). Polymer **1*GEM*** affords fragments **10** and **S27** after activation (d).

Here, we use values determined from integration of various specific signals in the ^{1}H NMR spectra of the mechanophore-containing polymer post-BMG, both before and after being washed with methanol,

along with the concentrated methanol wash itself. The nomenclature of the designation for each of these values is as follows:

$\boldsymbol{I^{x}}_{\mathbf{y}}$, where ***I*** represents a total numerical value of integration, **x** represents the ^{1}H NMR spectrum being used (α = post-BMG polymer before being washed with methanol –*Figure S39a*, β = post-BMG polymer after being washed with methanol – *Figure S39b*, and γ = the concentrated methanol washings – *Figure S39c*), and **y** represents a list of the signal designations being integrated.

We set value of integration of peak *a* as 1.00 in the ^{1}H NMR spectra of post-BMG polymer $\mathbf{1}_{GEM}$ both before and after being washed with methanol. Since there is no peak *a* in the concentrated methanol washings, the integration of peak *b* in the the concentrated methanol washings can be the difference (0.25 [1.39 - 1.14]) of integration of peak *b* of post-BMG polymer before and after being washed with methanol.

The percentage of retro-[2+2] cycloaddition (C_r) and released gemcitabine (C_{gem}) occurring during the activation were determined by the formula below:

$$C_r = \frac{I^{\alpha}{}_b}{2I^{\alpha}{}_a + I^{\alpha}{}_b} \times 100\%$$

$$C_{gem} = \frac{I^{\gamma}{}_c}{I^{\alpha}{}_a + I^{\gamma}{}_c} \times 100\%$$

Note: Released gemcitabine did not show well in the post-BMG polymer $\mathbf{1}_{GEM}$ before being washed with methanol but showed clearly in the concentrated methanol washings.

In this case, $\boldsymbol{C_r}$ **= 41%** [1.39 / (2 × 1.00 + 1.39) × 100%], and $\boldsymbol{C_{gem}}$ **= 21%** [0.27 / (1.00 + 0.27) × 100%].

8.7 Summary of Mechanophores Activated by Sonication

Table S2. Analysis of mechanical activation *via* ultrasound of mechanophore and control polymers.

No.	Mechanophore			Method	Pre-sonication		Post-sonication		Conversion (%)						
					M_n (kDa)	Đ	M_n (kDa)	Đ	Intact mechanophore [a]	Imine species	Aldehyde species	Total cleavage conversion	Average cleavage conversion	Functional cargo release	Average cargo release
1	Mechanophore polymer	5_{-155}	Run 1	Sonication (*i*PrOH) [b]	155	1.13	40	1.30	35	65	0	65	64	NA	
2			Run 2				42	1.21	37	63	0	63			
3	Mechanophore polymer	5_{-138}	Run 1	Sonication (H_2O) [c]	138	1.18	36	1.27	37	0	63	63	63		
4			Run 2				31	1.31	38	0	62	62			
5	Mechanophore polymer with hymecromone	1_{HYM}	Run 1		159	1.32	32	1.31	40	0	60	60	61	61	61
6			Run 2				31	1.35	38	0	62	62		61	
7	Mechanophore polymer with gemcitabine	1_{GEM}	Run 1		147	1.30	36	1.34	47	0	53	53	53	53	54
8			Run 2				31	1.33	47	0	53	53		54	
9	Control polymer	S20	NA		128	1.37	33	1.33	100	0	0	0	NA		
10	Control polymer with hymecromone	S21			125	1.11	36	1.37	100	0	0	0			
11	Imine polymer	9_{-112}			112	1.40	34	1.38	NA	0	100	NA			

Notes:

a) This value is given as the remaining percentage that is unaccounted for those that undergo the desired activation.

b) Sonication was carried out in CD_3CN/*i*PrOH (9/1).

c) Sonication was carried out in CD_3CN/H_2O (9/1).

8.8 Summary of Mechanophores Activated by Compression

Table S3. Analysis of mechanical activation *via* compression of mechanophore and control polymers.

No.	Mechanophore			Method	Pre-compression		Post-compression		Conversion (%)				
					M_n (kDa)	Đ	M_n (kDa)	Đ	Intact mechanophore [a]	Imine species	Aldehyde species	Total cleavage conversion	Average cleavage conversion
**1**	**Mechanophore polymer**	$\mathbf{5}_{-138}$	**Run 1**	**Compression**	138	1.18	34	2.70	87	7	6	13	20
**2**			**Run 2**[b]		144	1.15	36	2.12	72	23	3	26	
**3**	**Control polymer**	**S20**	**NA**		128	1.37	25	2.53	100	0	0	0	NA
**4**	**Imine polymer**	$\mathbf{9}_{-112}$			112	1.40	22	2.69	NA	34	66	NA	

Notes:

a) This value is given as the remaining percentage that is unaccounted for those that undergo the desired activation.

b) GPC data were collected in the DMAc system.

8.9 Summary of Mechanophores Activated by BMG

Table S4. Analysis of mechanical activation *via* BMG of mechanophore and control polymers.

No.	Mechanophore			Method	Pre-BMG M_n (kDa)	Pre-BMG *Đ*	Post-BMG M_n (kDa)	Post-BMG *Đ*	Conversion (%) Intact mechanophore [a]	Imine species	Aldehyde species	Total cleavage conversion	Average cleavage conversion	Functional cargo release	Average cargo release
**1**	**Mechanophore polymer**	**5**$_{-155}$	**Run 1**	**BMG** [b] **(H_2O)**	155	1.13	10	1.35	34	0	66	66	68	NA	
**2**			**Run 2**				8	1.36	31	0	69	69			
**3**			**Run 1**	**BMG** [c] **(Dry)**			6	1.53	33	62	5	67	65		
**4**			**Run 2**				5	1.49	37	58	5	63			
**5**	**Mechanophore polymer with hymecromone**	**1**$_{HYM}$	**Run 1**	**BMG** [b] **(H_2O)**	159	1.32	10	1.69	48	0	52	52	51	52	51
**6**			**Run 2**				10	1.49	49	0	51	51		51	
**7**	**Mechanophore polymer with gemcitabine**	**1**$_{GEM}$	**Run 1**		147	1.30	11	1.49	59	0	41	41	41	16	19
**8**			**Run 2**				10	1.39	60	0	40	40		21	
**9**	**Control polymer**	**S20**	**NA**	**BMG** [b] **(H_2O)**	128	1.37	11	1.59	96	0	4	4	5	NA	
**10**				**BMG** [c] **(Dry)**			10	1.54	94	0	6	6			
**11**	**Control polymer with hymecromone**	**S21**		**BMG** [b] **(H_2O)**	125	1.11	12	1.55	96	0	4	4	NA		
**12**	**Imine polymer**	**9**$_{-114}$		**BMG** [b] **(H_2O)**	114	1.42	11	1.33	NA	0	100	NA			
**13**				**BMG** [c] **(Dry)**			6	1.33		90	10				

Notes:

a) This value is given as the remaining percentage that is unaccounted for those that undergo the desired activation.

b) Milled as a 1/1 w/w mix of polymers and water.

c) Polymer was dried in the milling jar for 48 h at 45 °C in a vacuum oven before use.

9 NMR Spectra

9.1 Small Molecule NMR Spectra

9.1.1 Spectra of S2

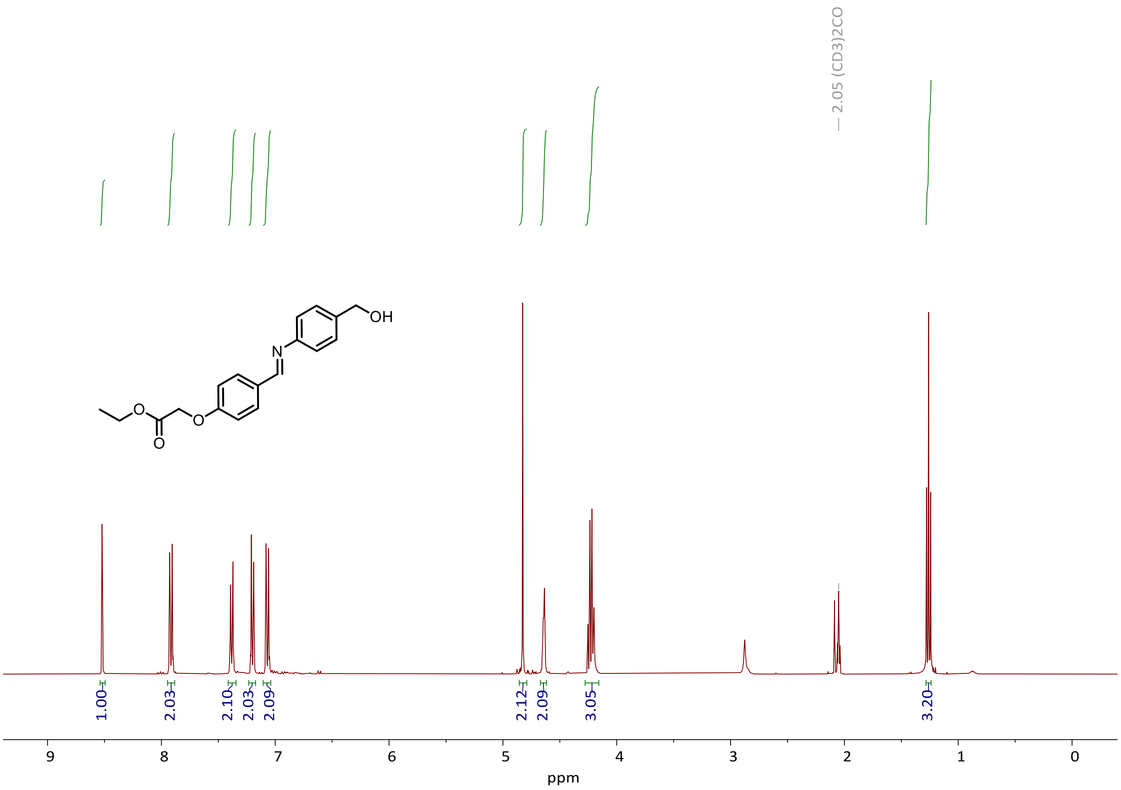

Spectrum S1. ^{1}H NMR (400 MHz, Acetone-d_6, 298 K) spectrum of compound **S2**.

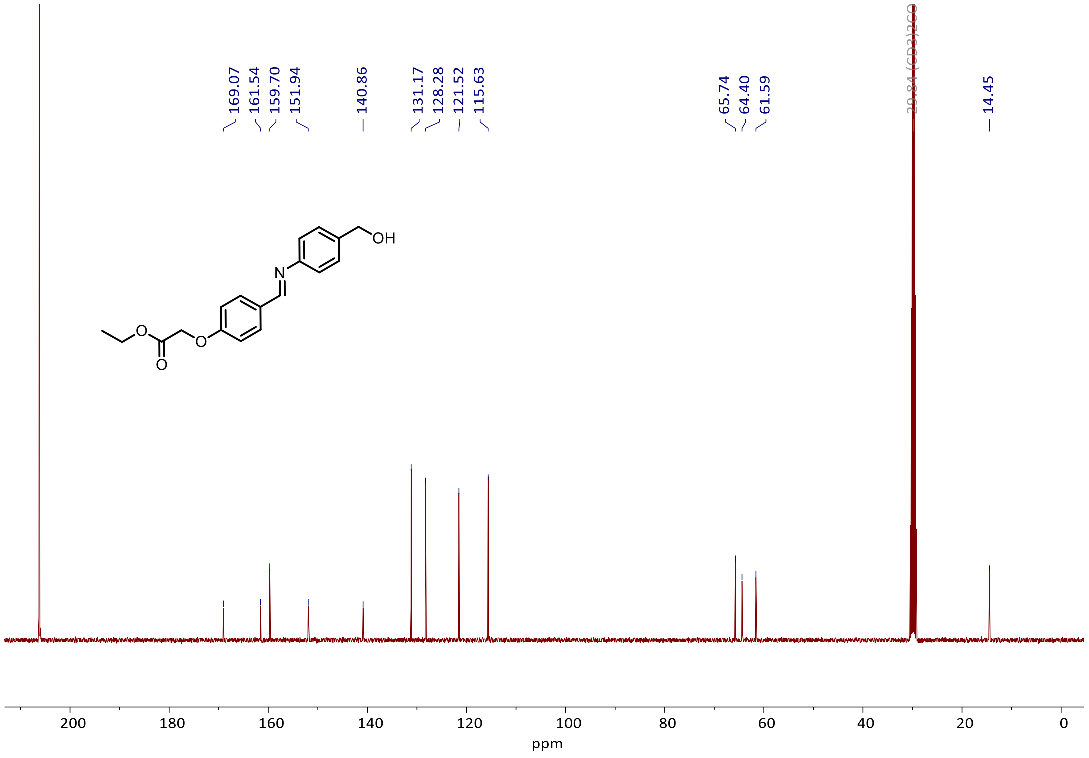

Spectrum S2. ^{13}C NMR (101 MHz, Acetone-d_6, 298 K) spectrum of compound **S2**.

9.1.2 Spectra of S5

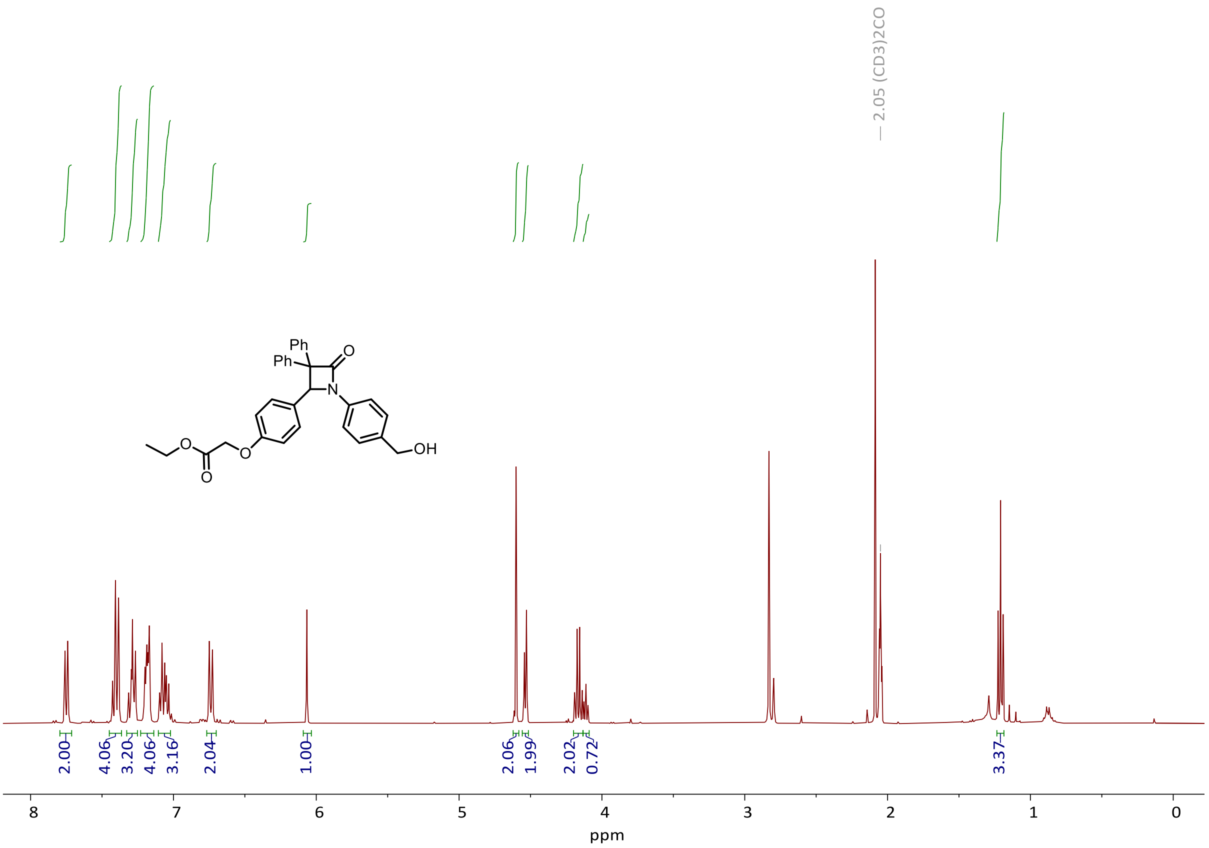

Spectrum S3. ^{1}H NMR (400 MHz, Acetone-d_6, 298 K) spectrum of compound **S5**.

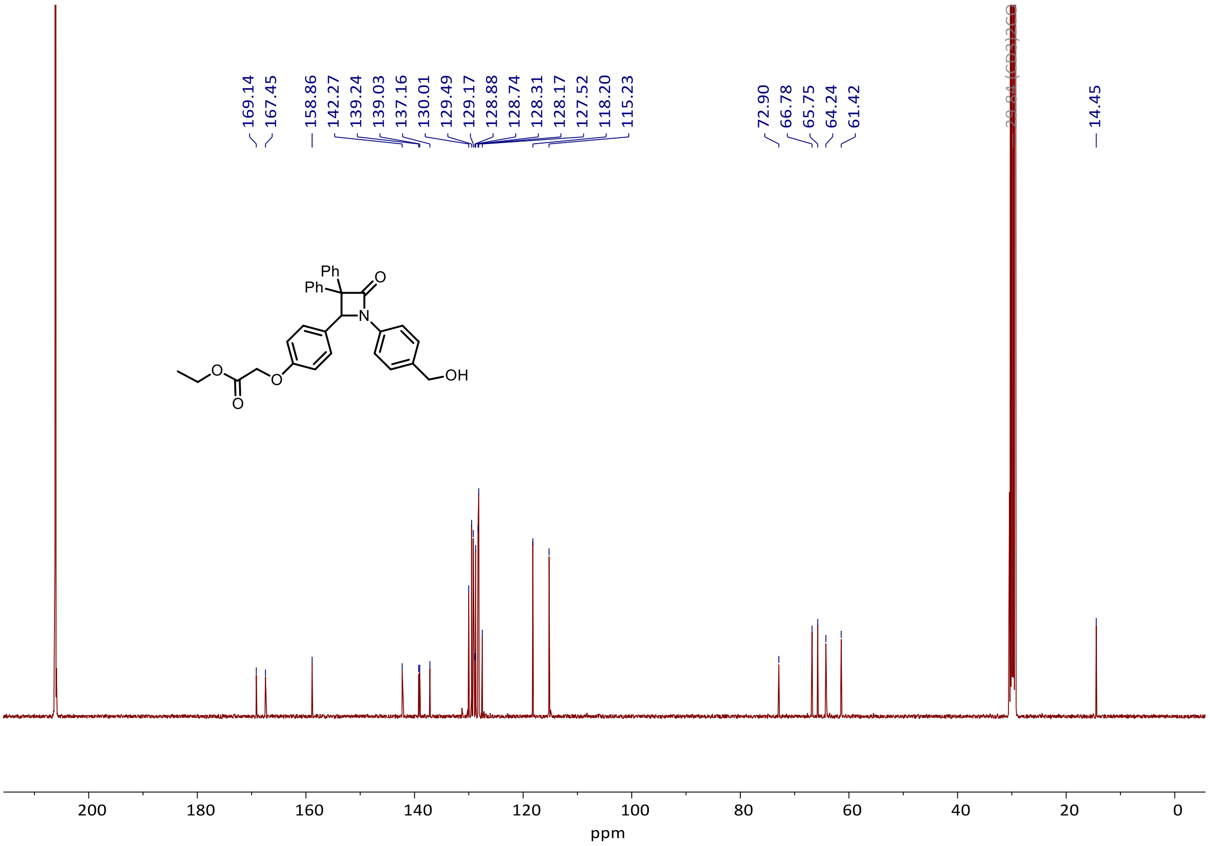

Spectrum S4. ^{13}C NMR (101 MHz, Acetone-d_6, 298 K) spectrum of compound **S5**.

9.1.3 Spectra of S6

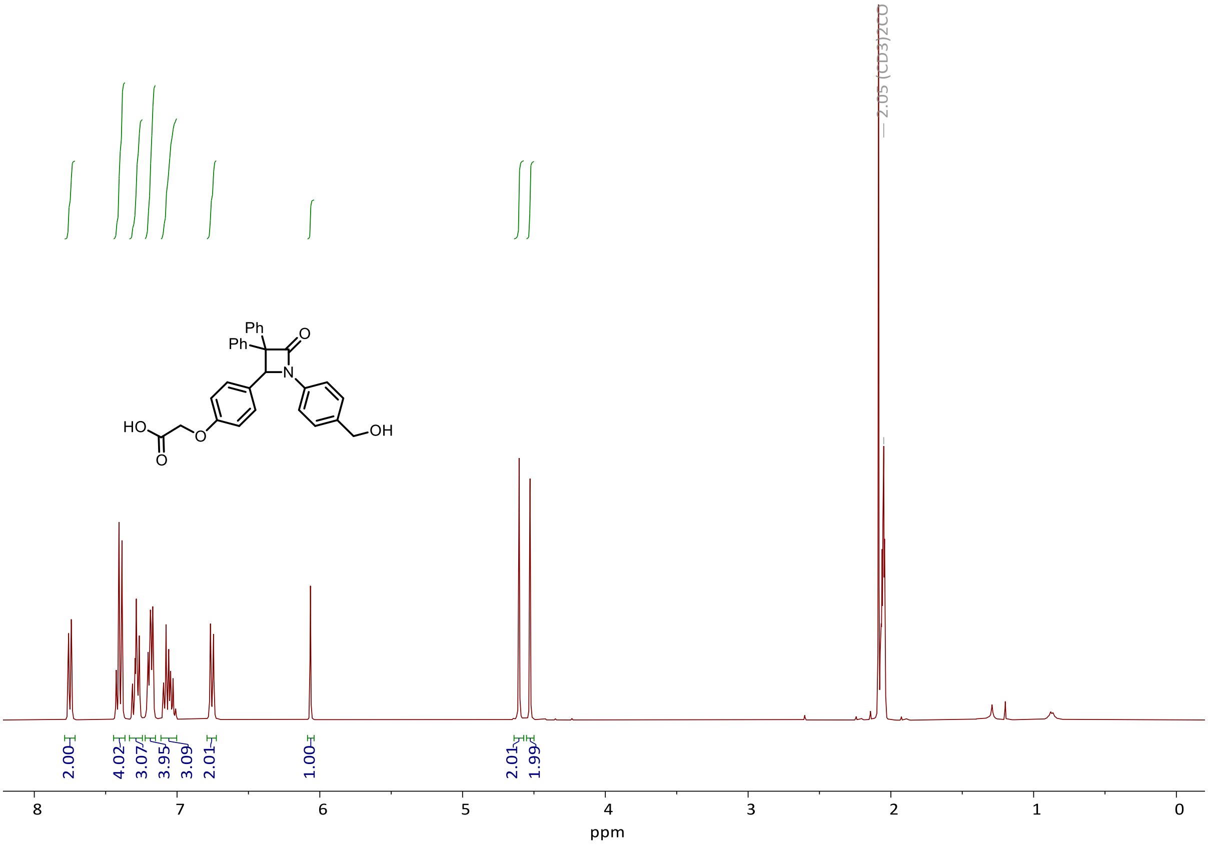

Spectrum S5. ^{1}H NMR (400 MHz, Acetone-d_6, 298 K) spectrum of compound **S6**.

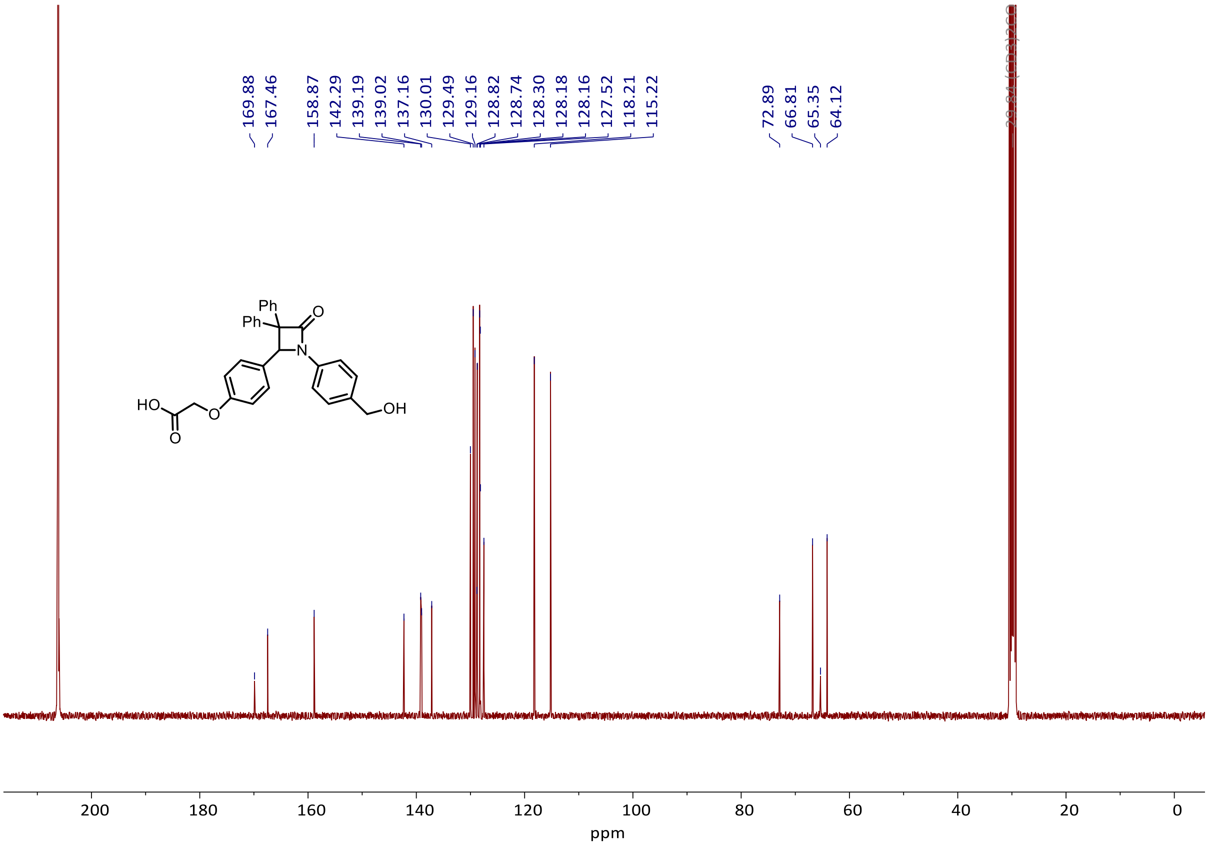

Spectrum S6. ^{13}C NMR (101 MHz, Acetone-d_6, 298 K) spectrum of compound **S6**.

9.1.4 Spectra of S10

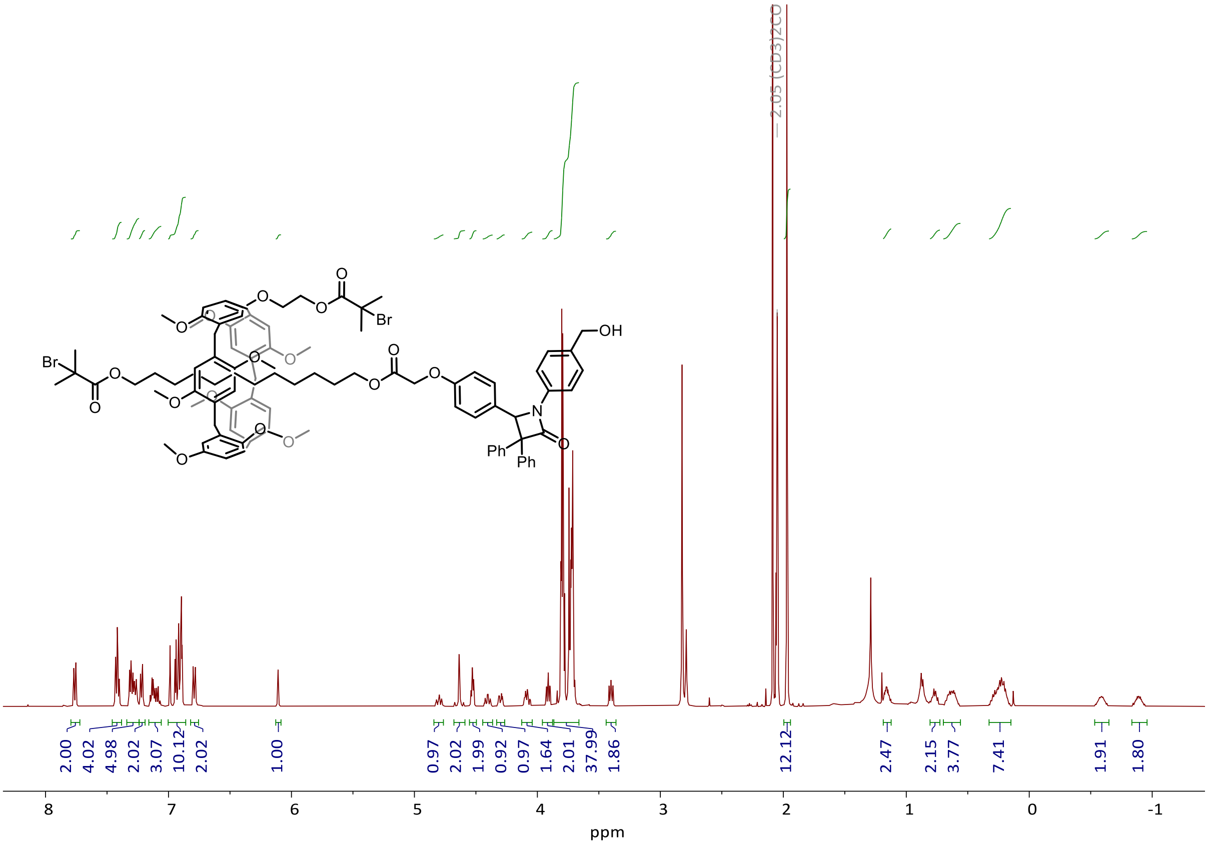

Spectrum S7. ^{1}H NMR (500 MHz, Acetone-d_6, 298 K) spectrum of compound **S10**.

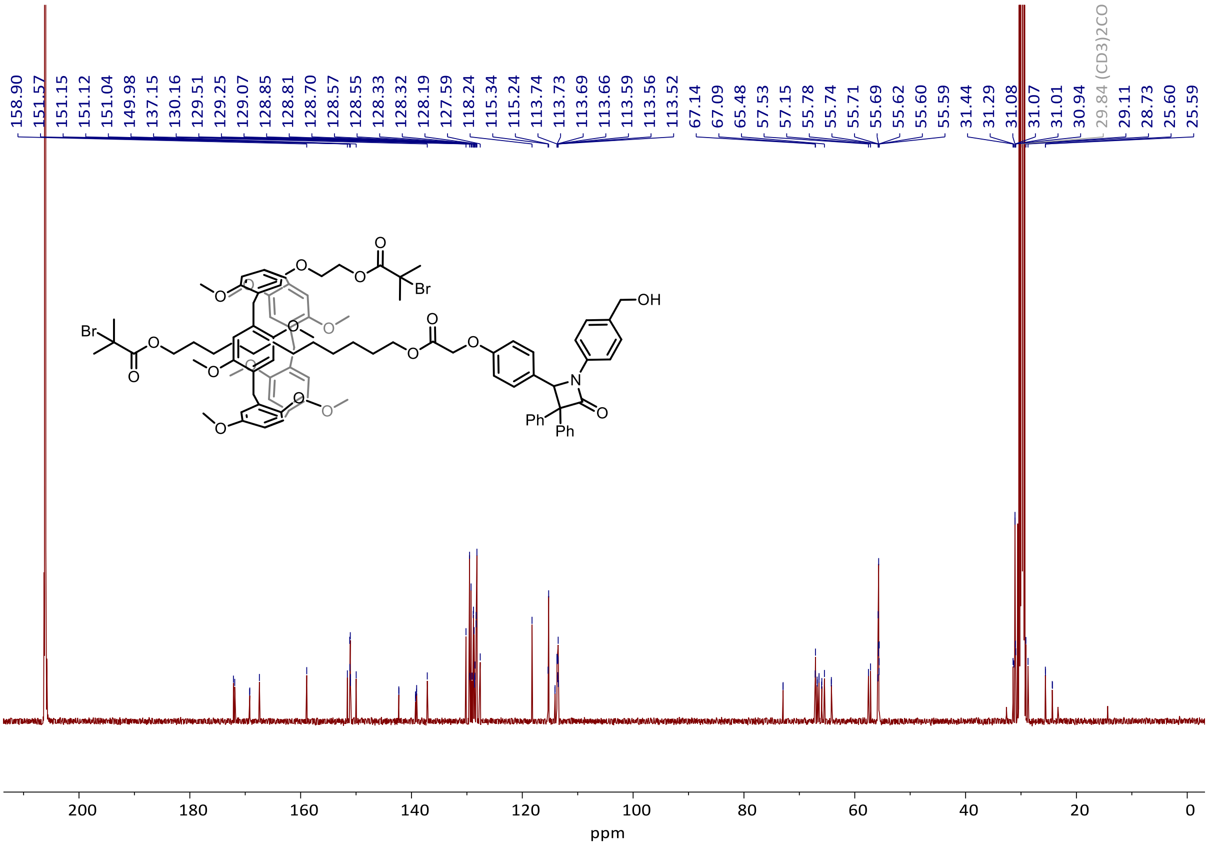

Spectrum S8. ^{13}C NMR (126 MHz, Acetone-d_6, 298 K) spectrum of compound **S10**.

9.1.5 Spectra of S11

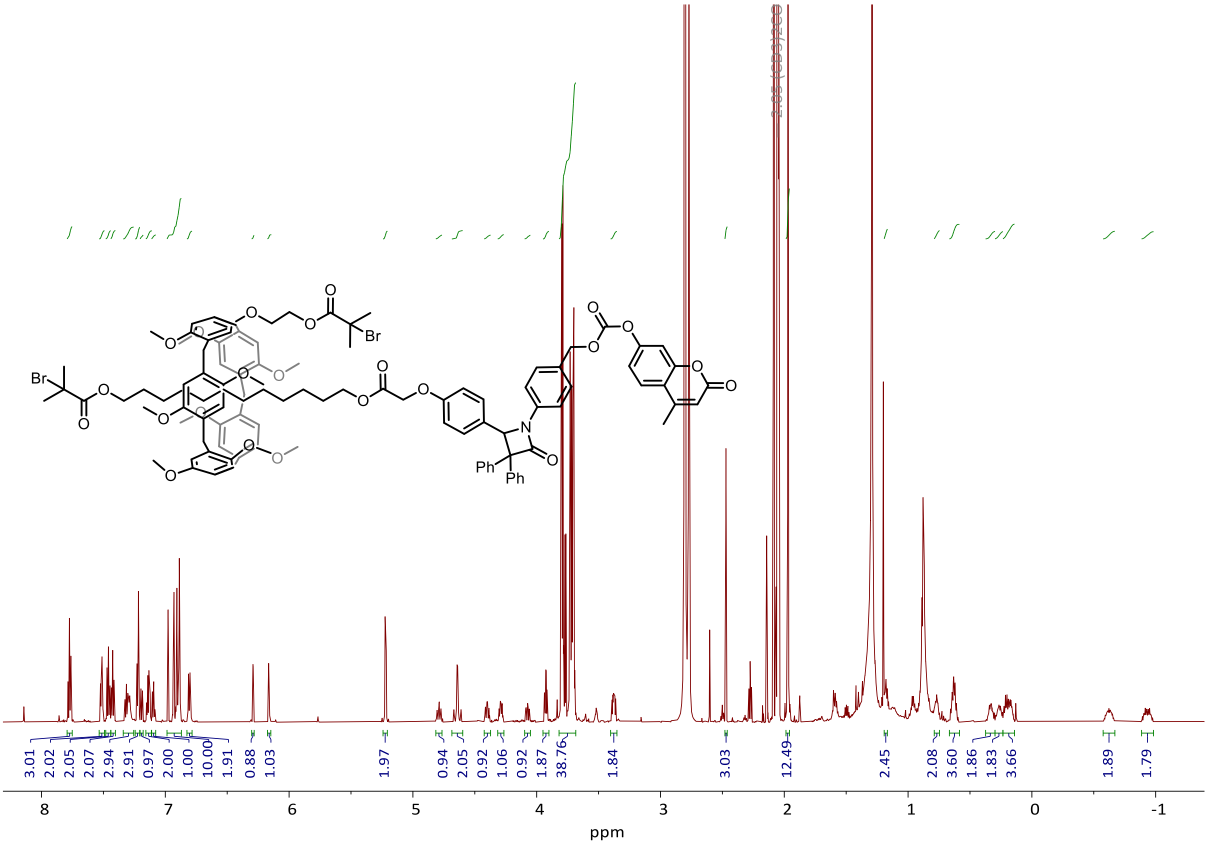

Spectrum S9. ^{1}H NMR (700 MHz, Acetone-d_6, 298 K) spectrum of compound **S11**.

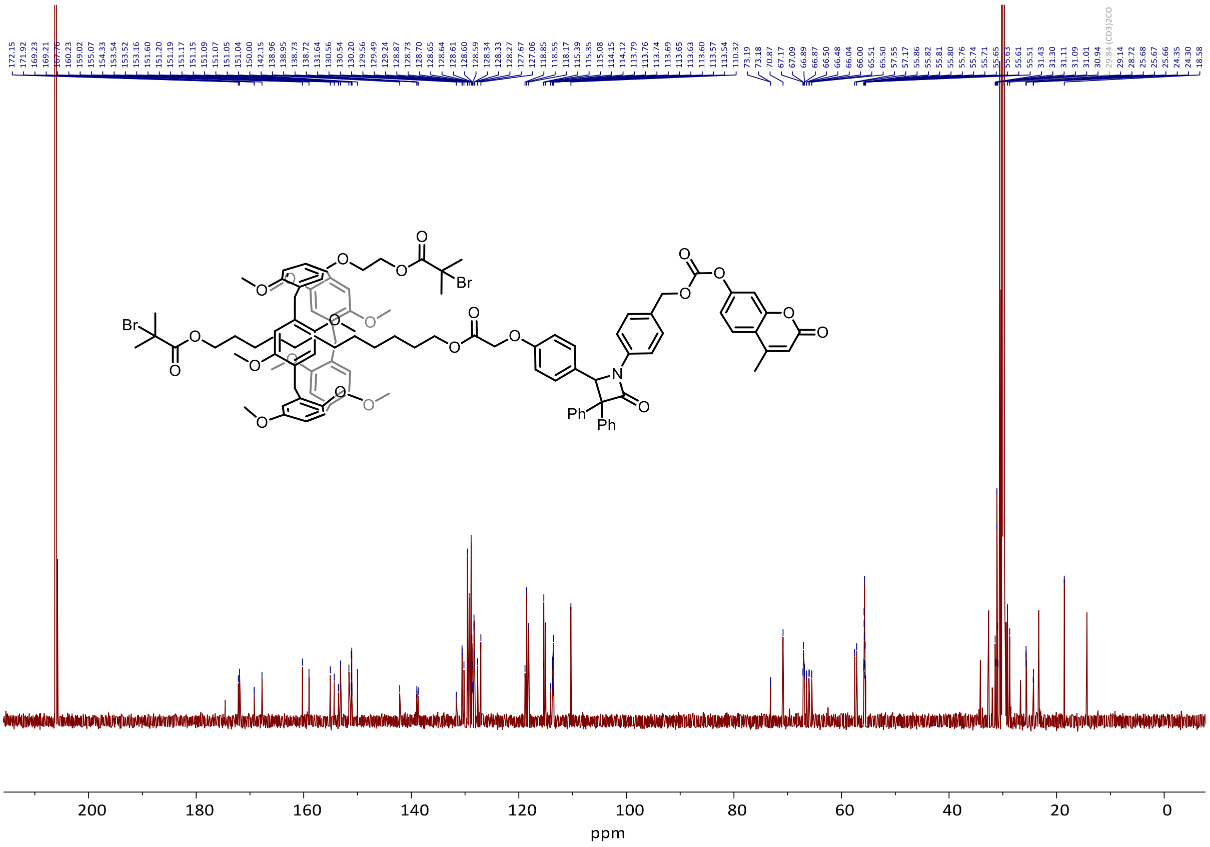

Spectrum S10. ^{13}C NMR (176 MHz, Acetone-d_6, 298 K) spectrum of compound **S11**.

9.1.6 Spectra of S12

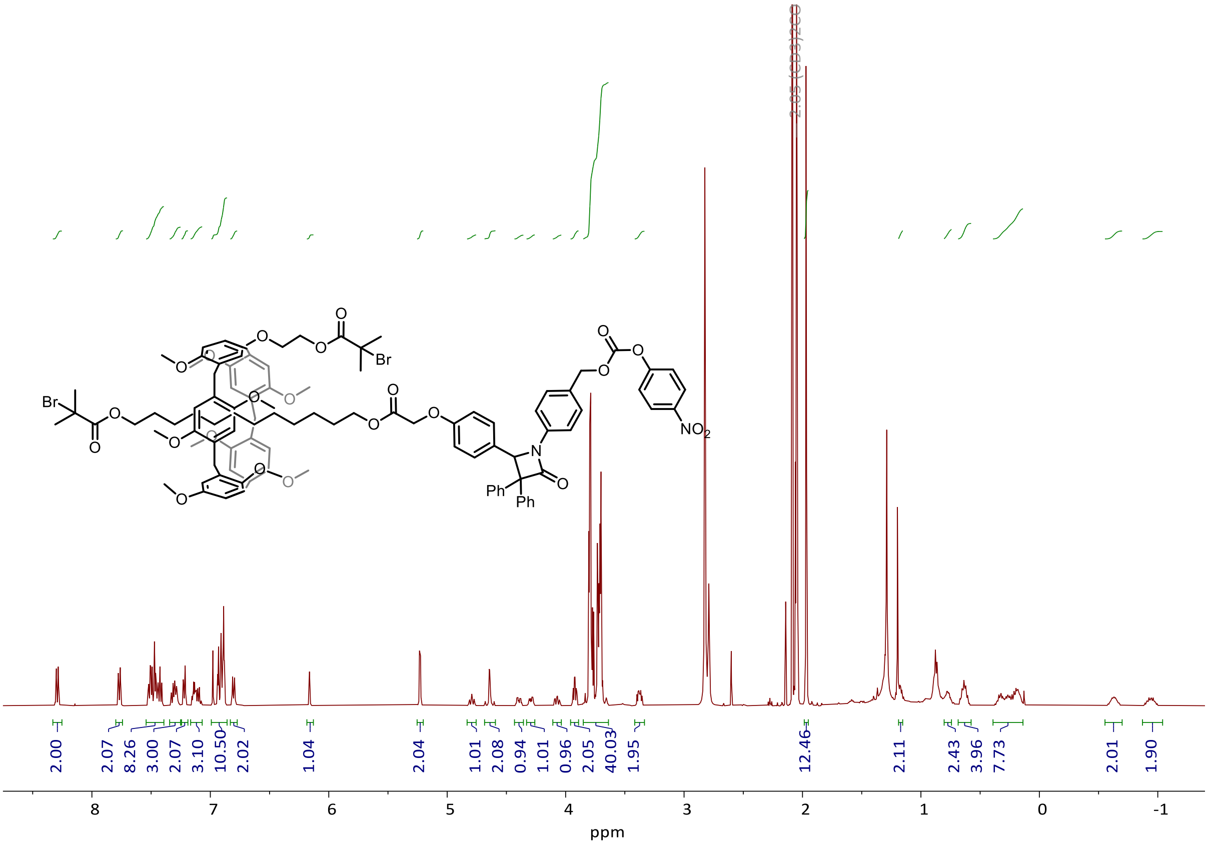

Spectrum S11. ^{1}H NMR (500 MHz, Acetone-d_6, 298 K) spectrum of compound **S12**.

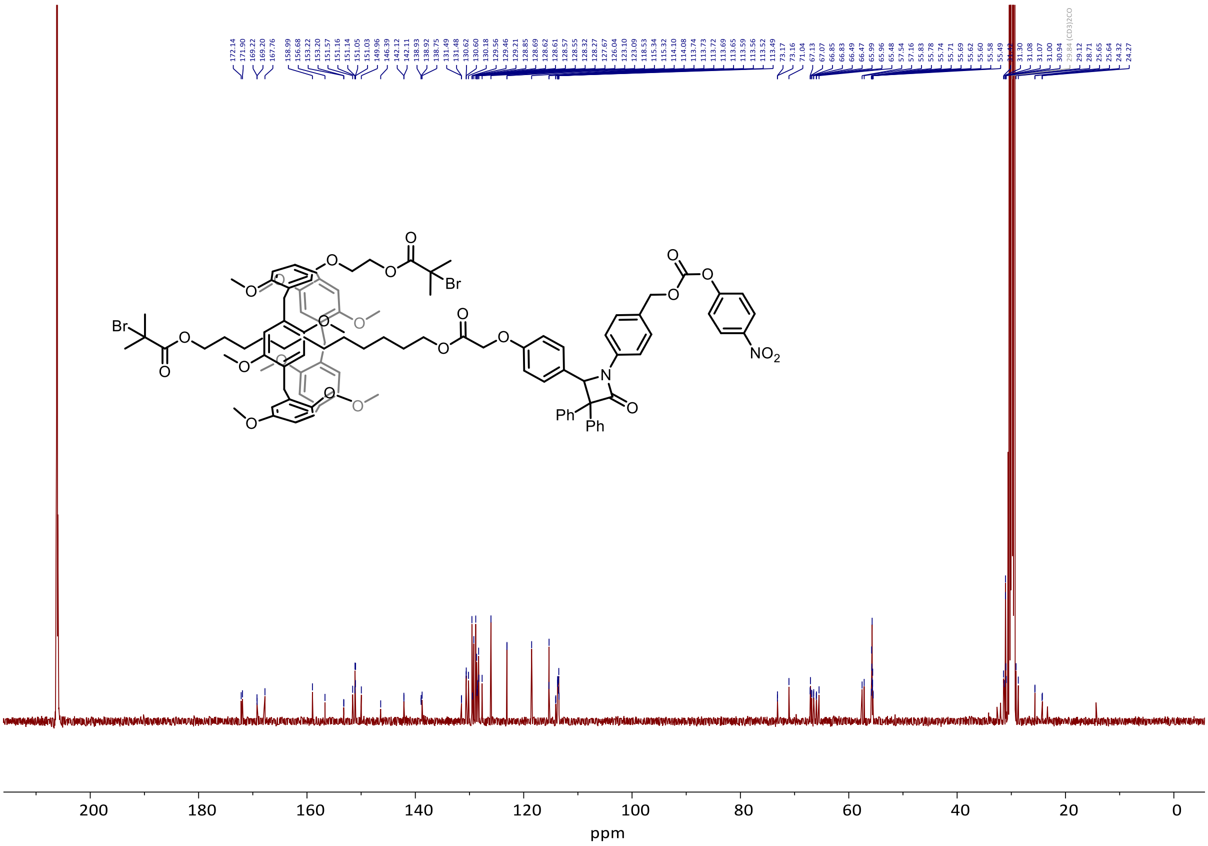

Spectrum S12. ^{13}C NMR (126 MHz, Acetone-d_6, 298 K) spectrum of compound **S12**.

9.1.7 Spectra of S14

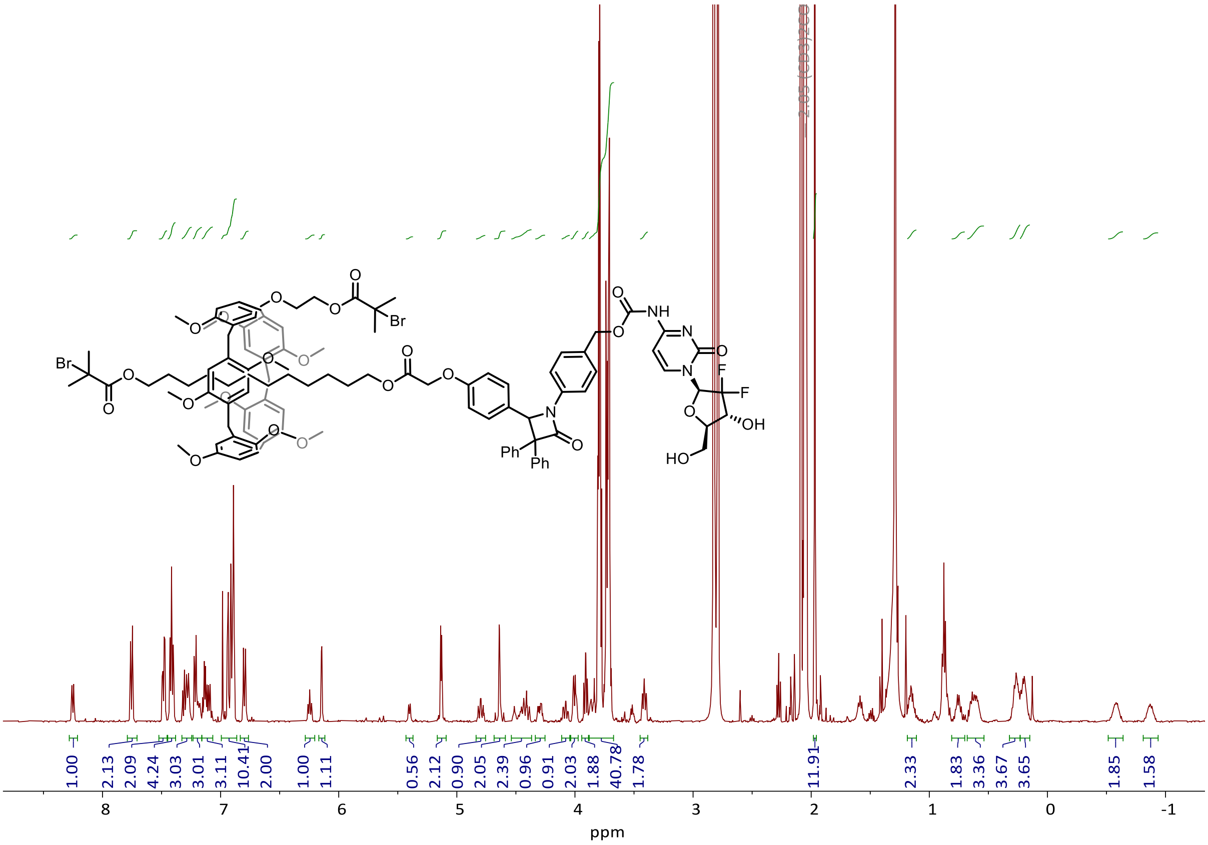

Spectrum S13. ^{1}H NMR (500 MHz, Acetone-d_6, 298 K) spectrum of compound **S14**.

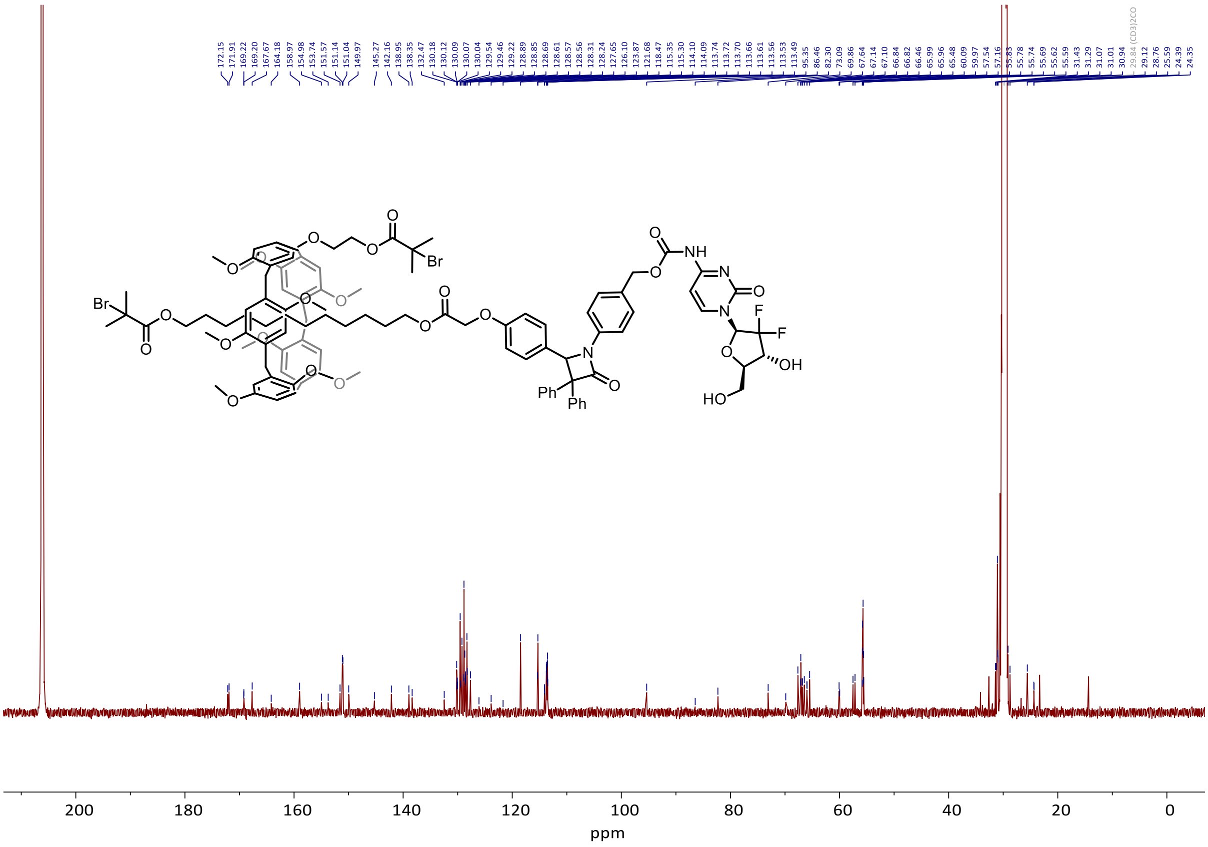

Spectrum S14. ^{13}C NMR (151 MHz, Acetone-d_6, 298 K) spectrum of compound **S14**.

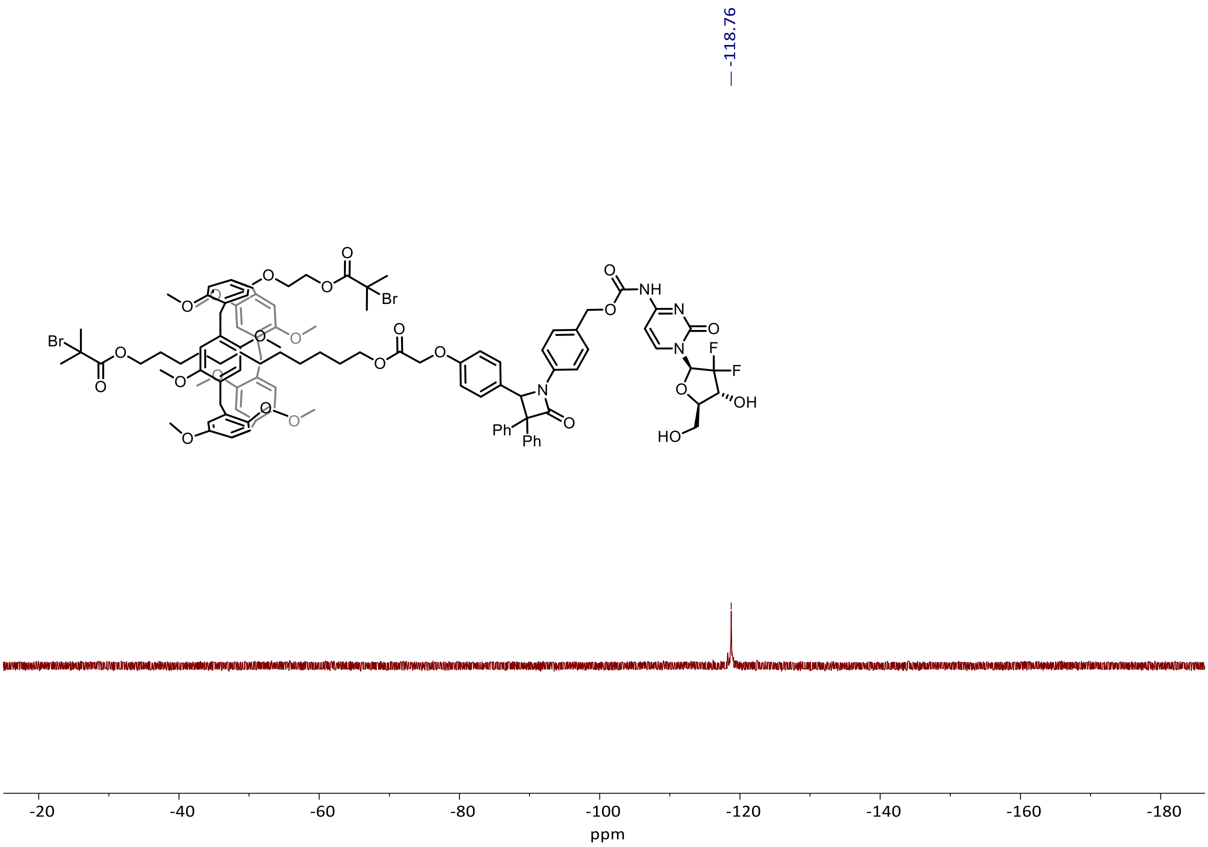

Spectrum S15. ^{19}F NMR (471 MHz, Acetone-d_6, 298 K) spectrum of compound **S14**.

9.1.8 Spectra of S16

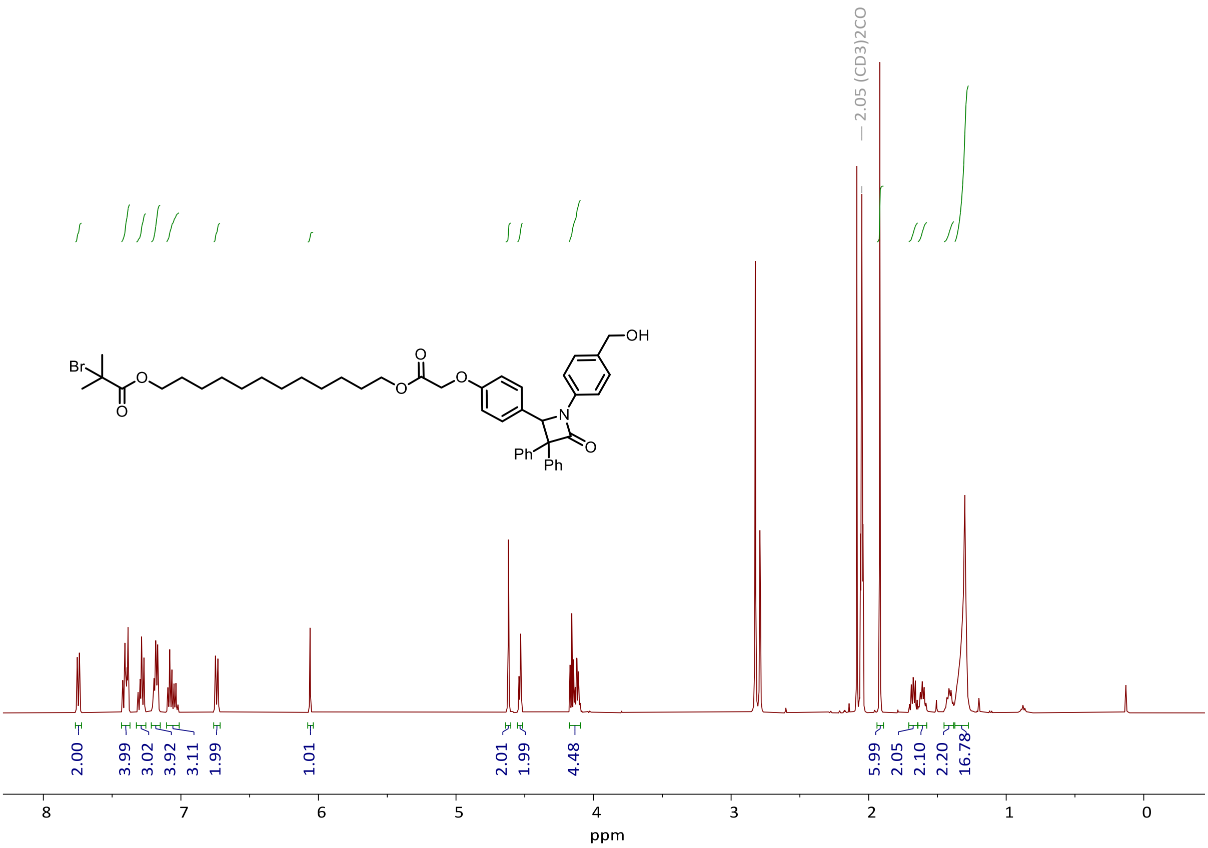

Spectrum S16. ^{1}H NMR (500 MHz, Acetone-d_6, 298 K) spectrum of compound **S16**.

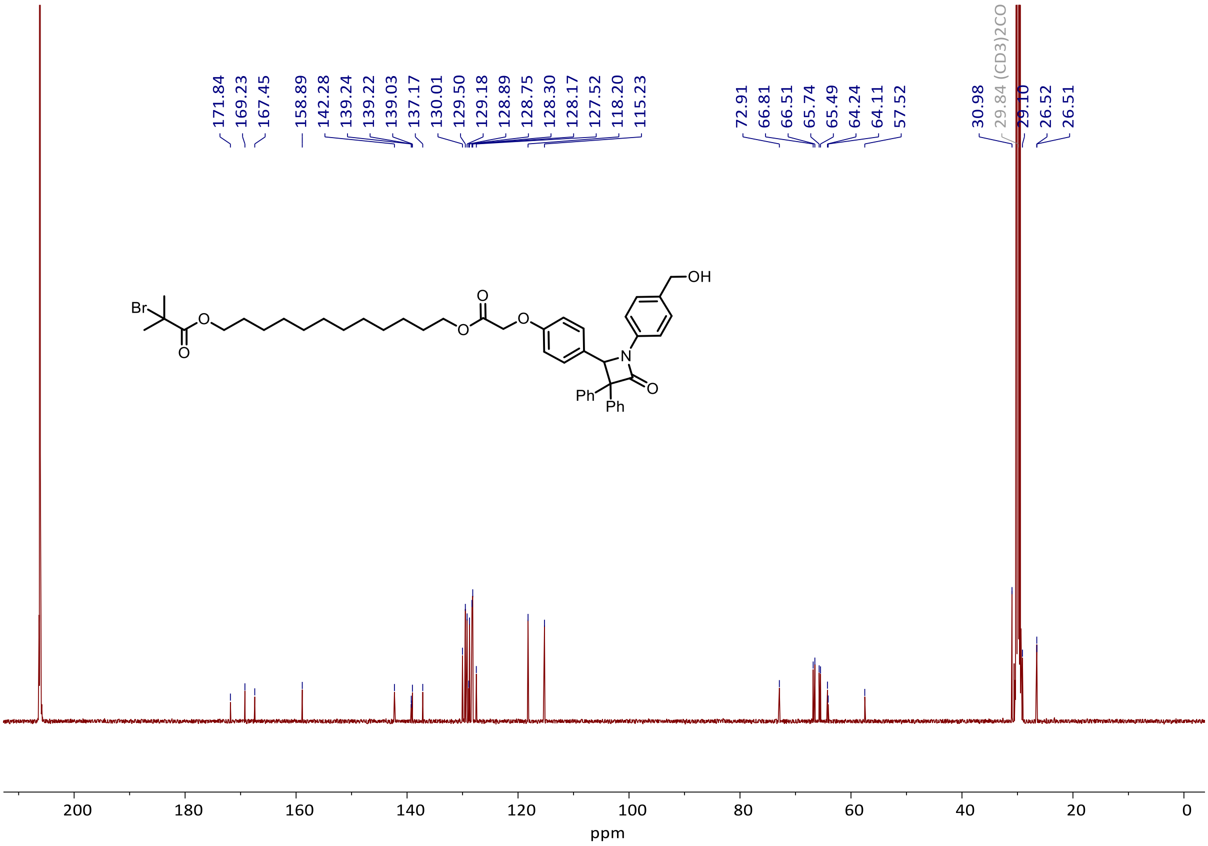

Spectrum S17. ^{13}C NMR (126 MHz, Acetone-d_6, 298 K) spectrum of compound **S16**.

9.1.9 Spectra of S17

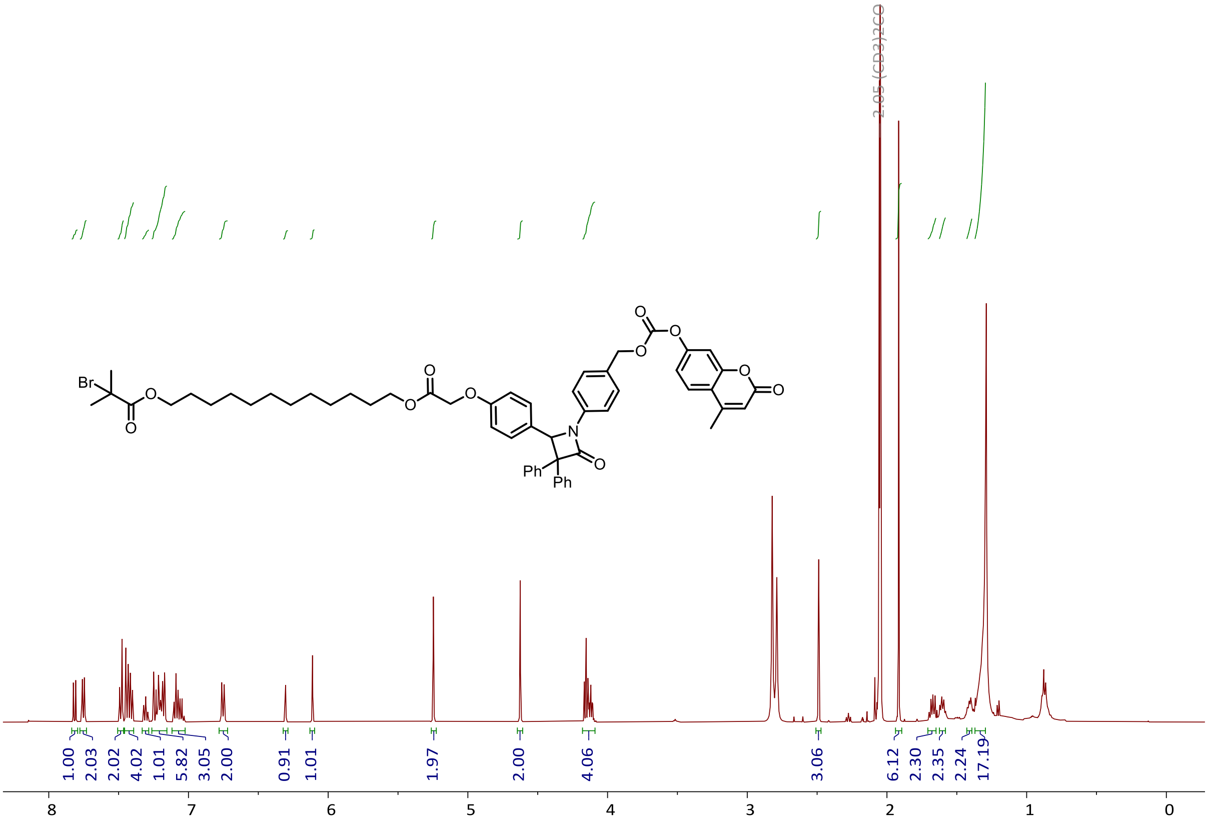

Spectrum S18. ^{1}H NMR (500 MHz, Acetone-d_6, 298 K) spectrum of compound **S17**.

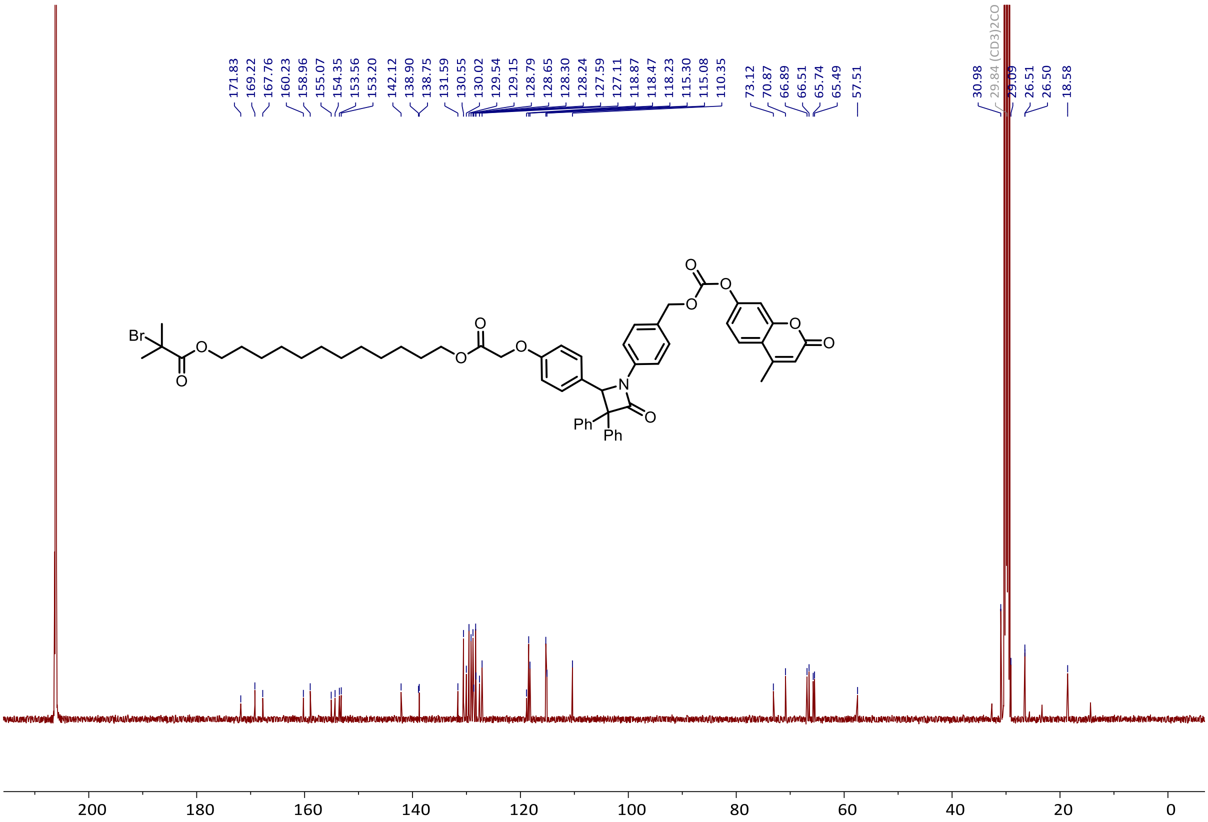

Spectrum S19. ^{13}C NMR (126 MHz, Acetone-d_6, 298 K) spectrum of compound **S17**.

9.1.10 Spectra of 7

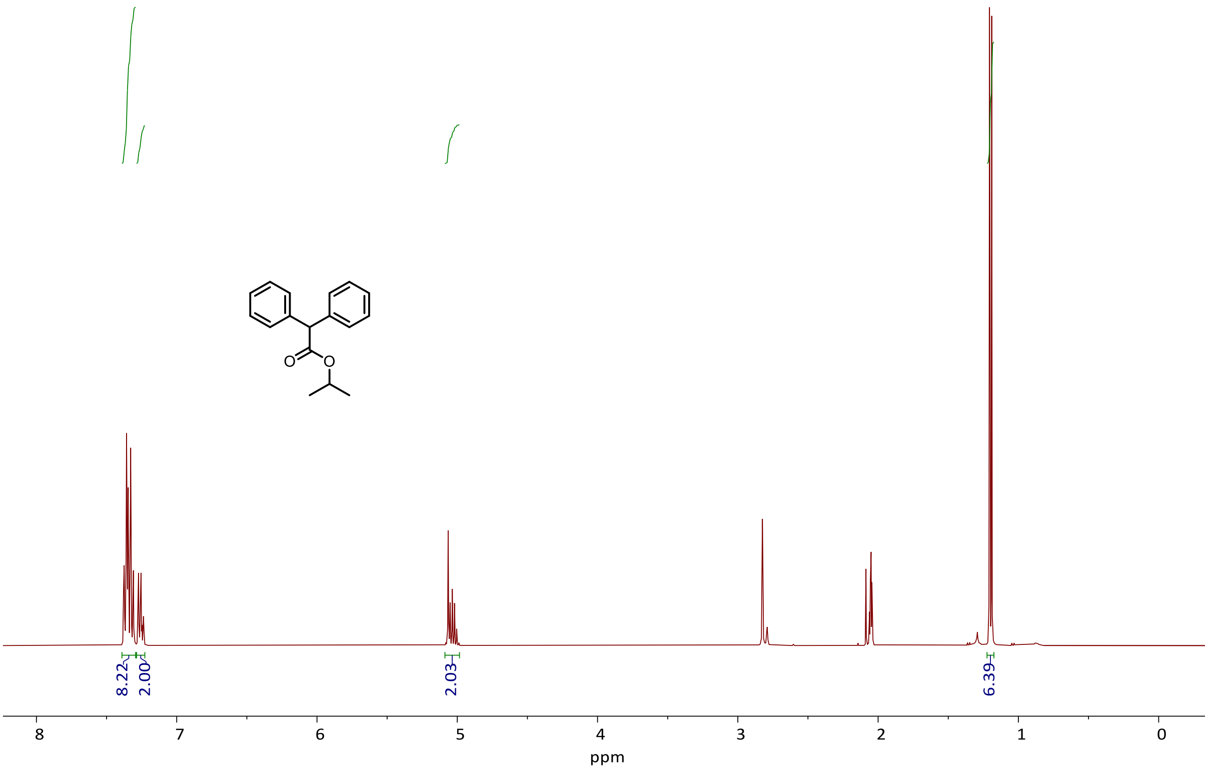

Spectrum S20. ^{1}H NMR (400 MHz, Acetone-d_6, 298 K) spectrum of compound **7**.

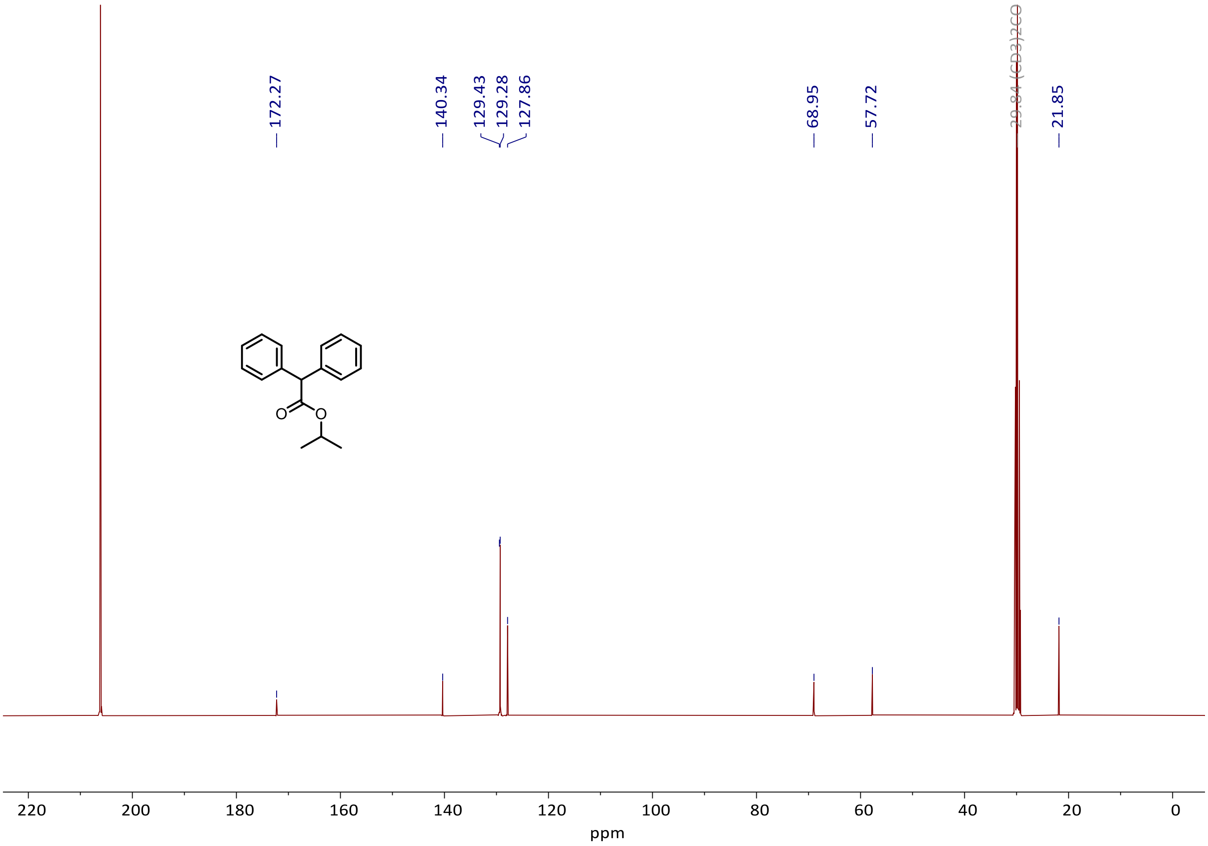

Spectrum S21. ^{13}C NMR (101 MHz, Acetone-d_6, 298 K) spectrum of compound **7**.

9.2 Polymer NMR Spectra

9.2.1 Spectra of polymer 5-138

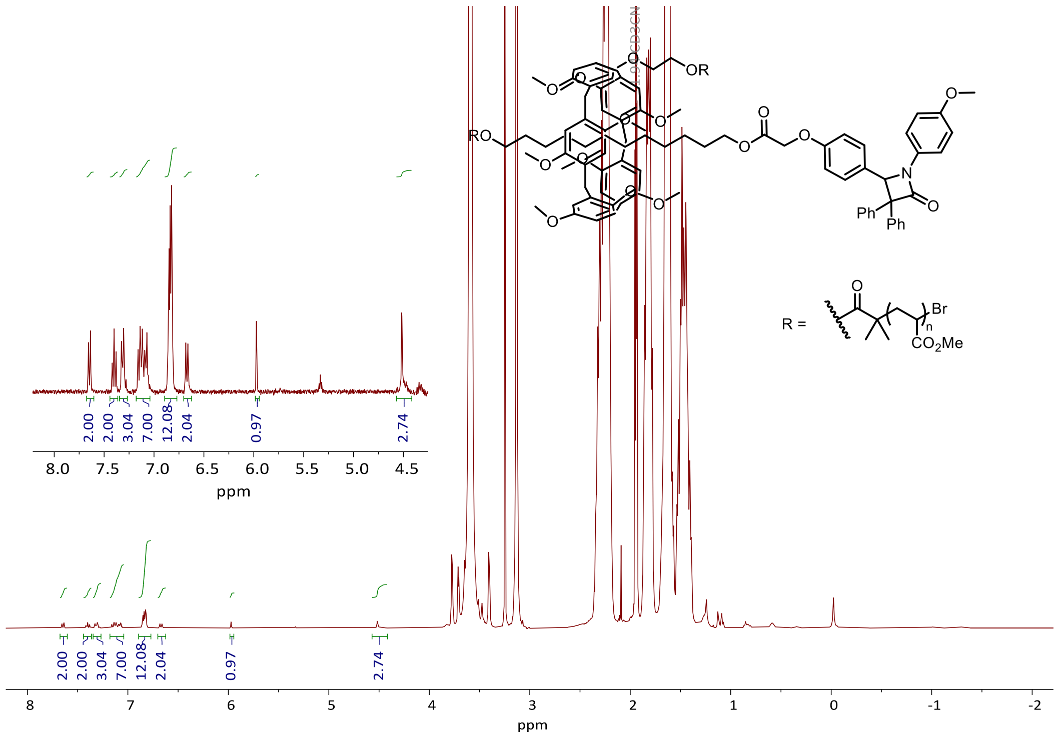

Spectrum S22. ^{1}H NMR (400 MHz, Acetonitrile-d_3/H_2O (9/1), 298 K) spectrum of polymer **5**$_{-138}$

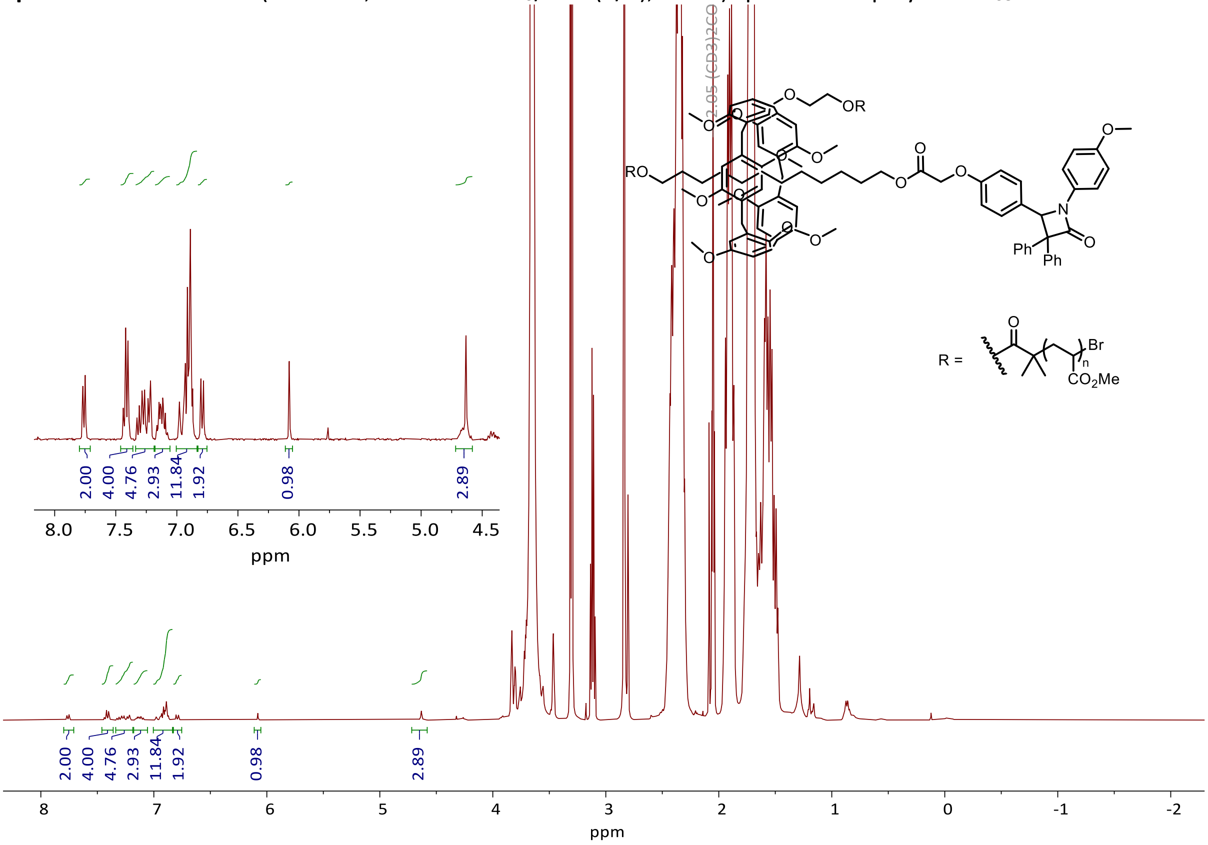

Spectrum S23. ^{1}H NMR (400 MHz, Acetone-d_6, 298 K) spectrum of polymer **5**$_{-138}$.

9.2.2 Spectra of polymer 5-155

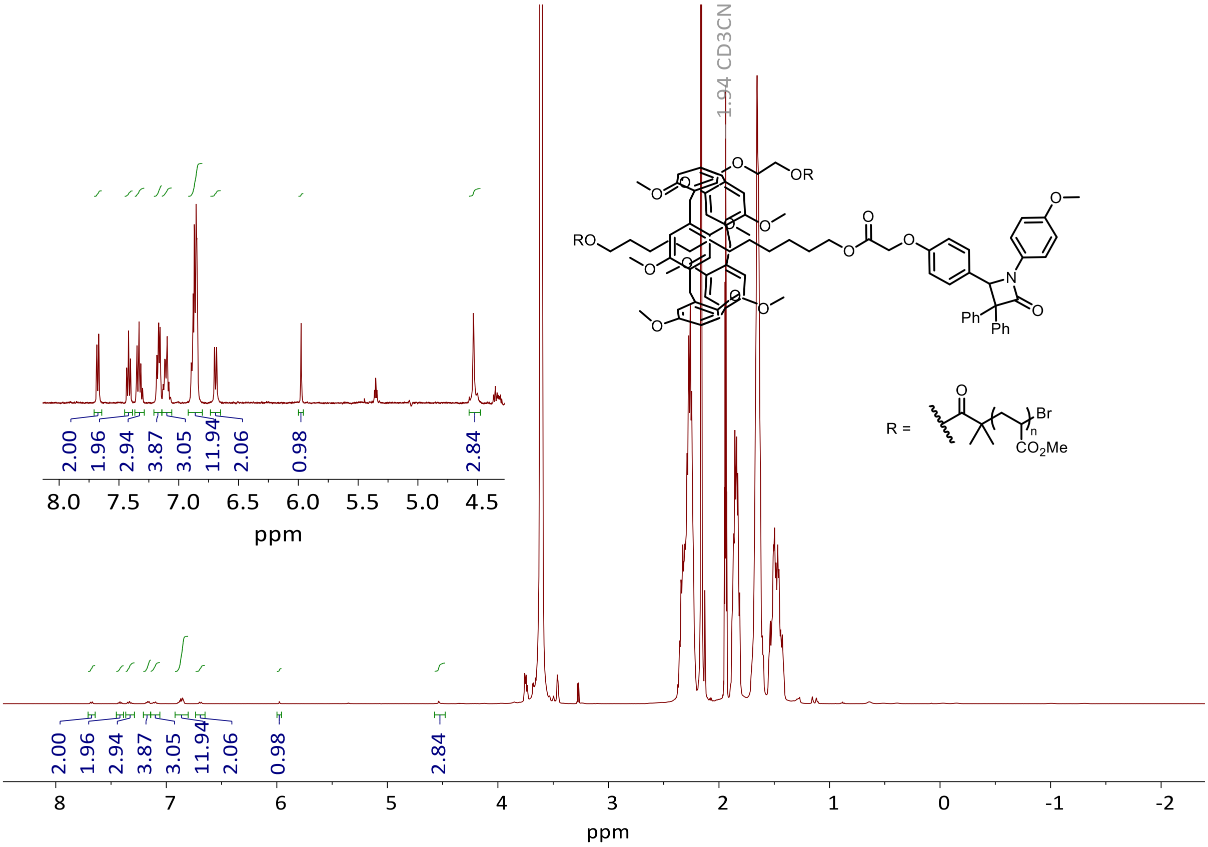

Spectrum S24. ^{1}H NMR (500 MHz, Acetonitrile-d_3, 298 K) spectrum of polymer **5-155**

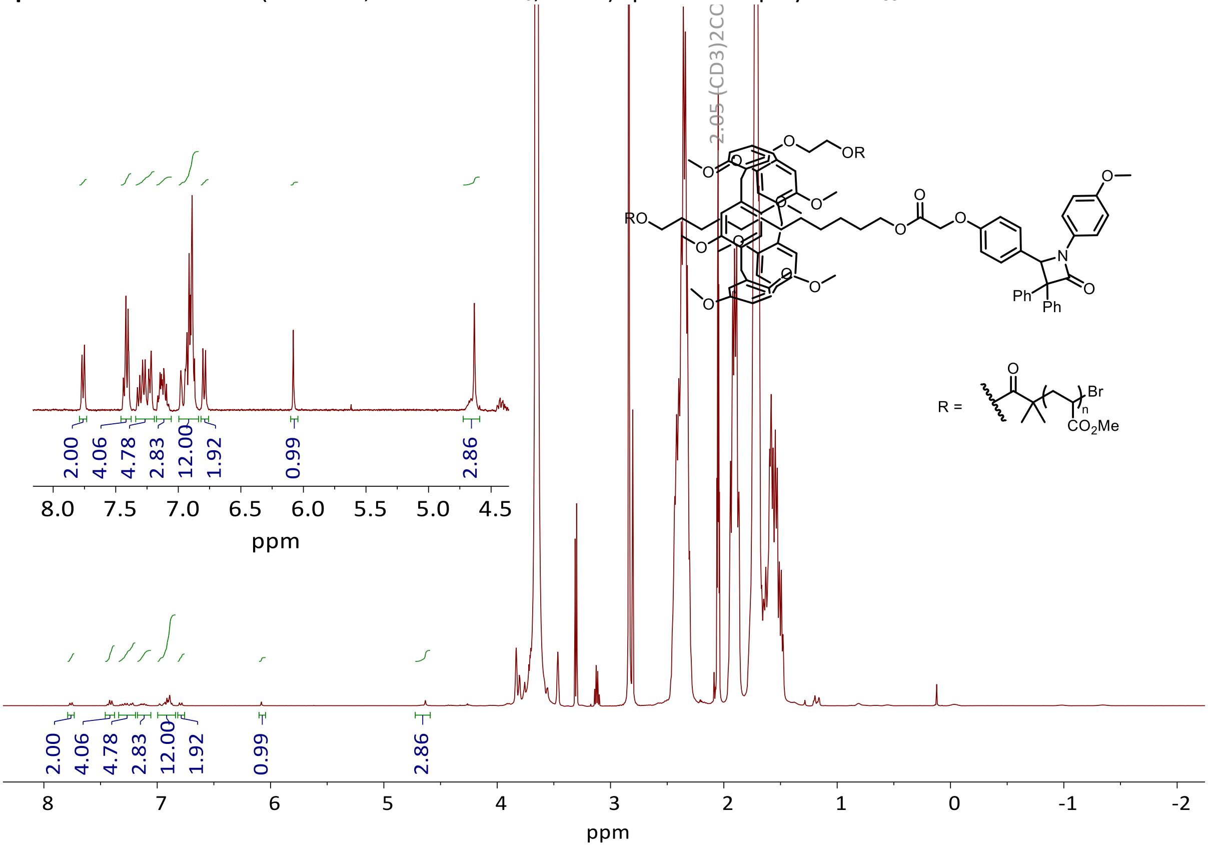

Spectrum S25. ^{1}H NMR (400 MHz, Acetone-d_6, 298 K) spectrum of polymer **5-155**.

9.2.3 Spectra of polymer 1_{HYM}

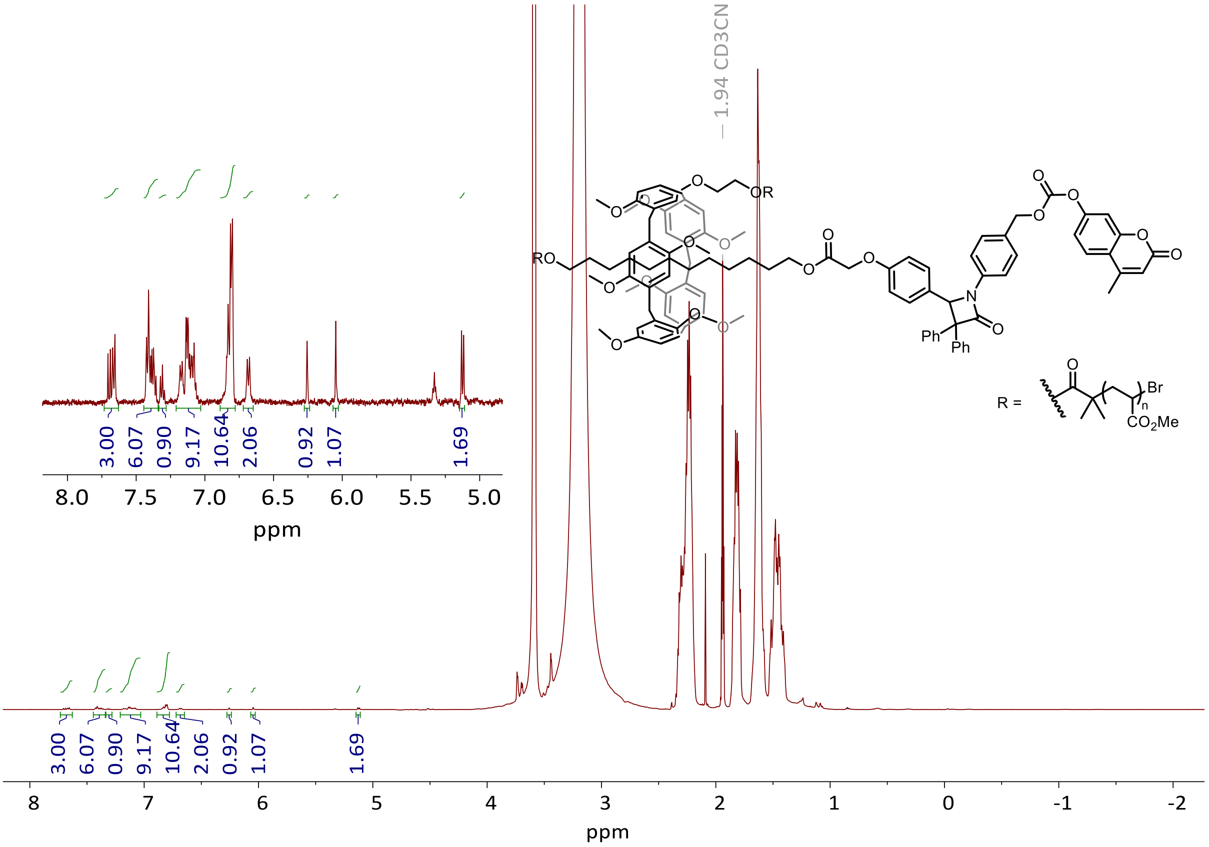

Spectrum S26. ^{1}H NMR (500 MHz, Acetonitrile-d_3/H_2O (9/1), 298 K) spectrum of polymer $\mathbf{1}_{HYM}$

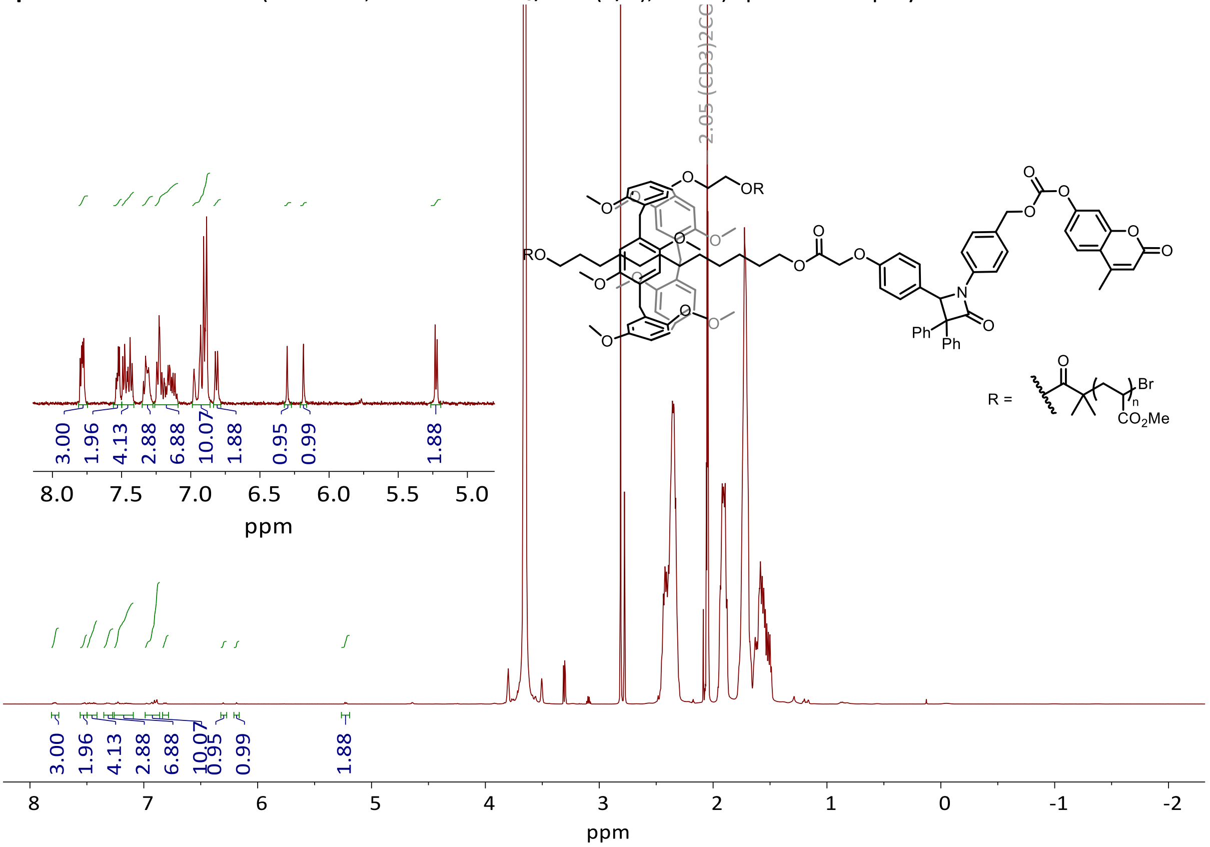

Spectrum S27. ^{1}H NMR (500 MHz, Acetone-d_6, 298 K) spectrum of polymer $\mathbf{1}_{HYM}$.

9.2.4 Spectra of polymer 1_{GEM}

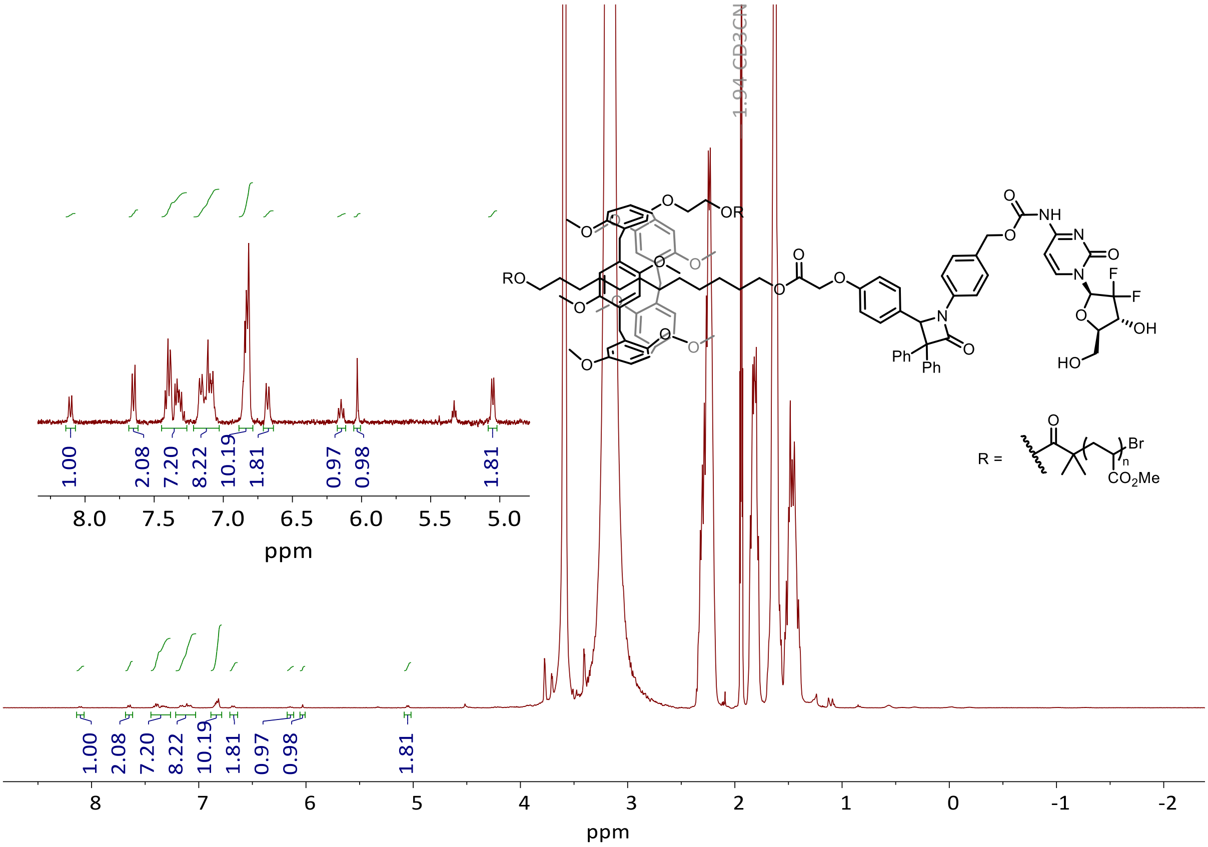

Spectrum S28. ^{1}H NMR (400 MHz, Acetonitrile-d_3/H_2O (9/1), 298 K) spectrum of polymer $\mathbf{1}_{GEM}$

9.2.5 Spectra of polymer S20

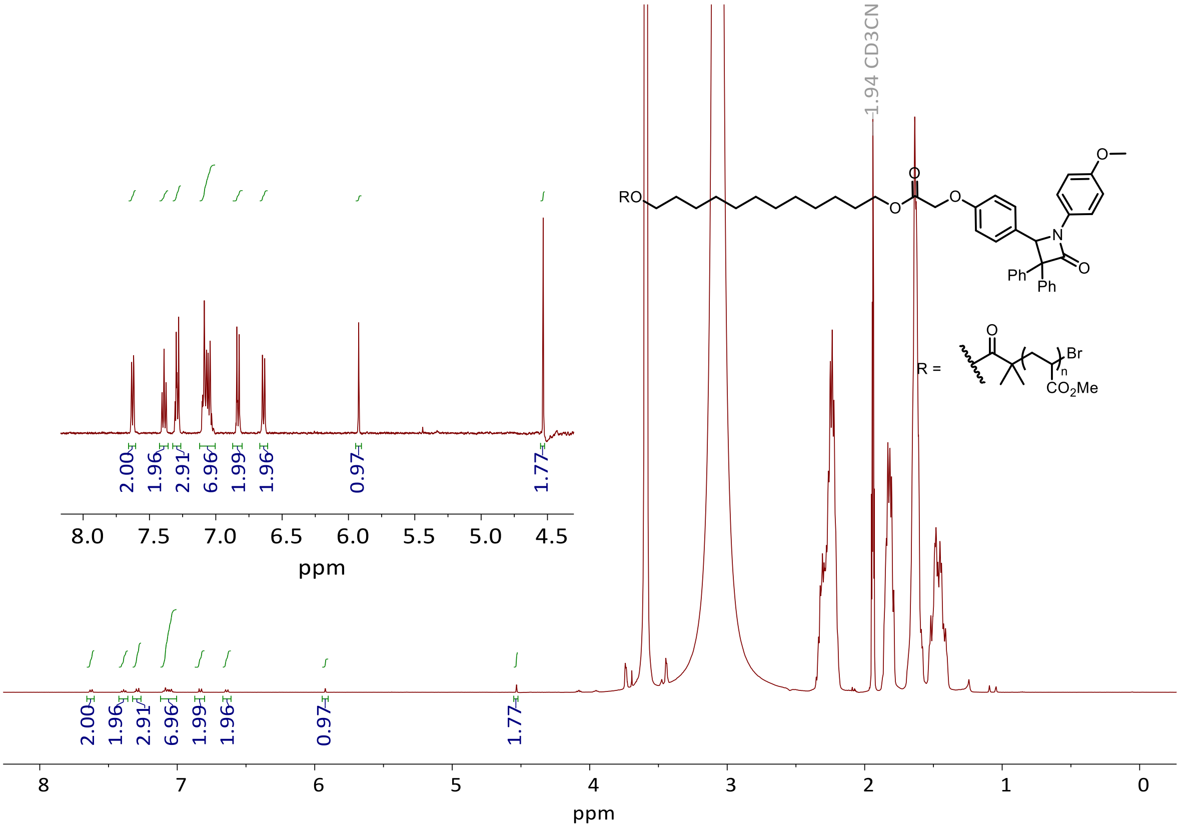

Spectrum S29. ^{1}H NMR (500 MHz, Acetonitrile-d_3/H_2O (9/1), 298 K) spectrum of polymer **S20**

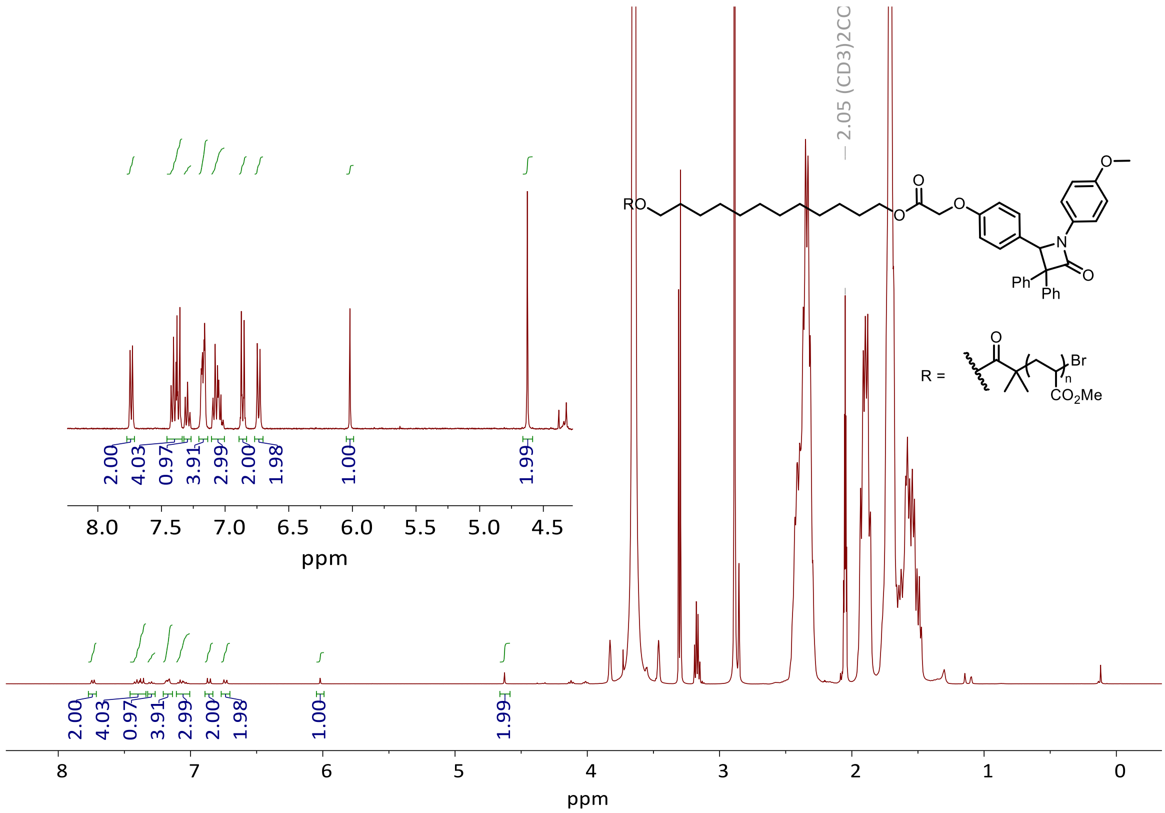

Spectrum S30. ^{1}H NMR (400 MHz, Acetone-d_6, 298 K) spectrum of polymer **S20**.

9.2.6 Spectra of polymer S21

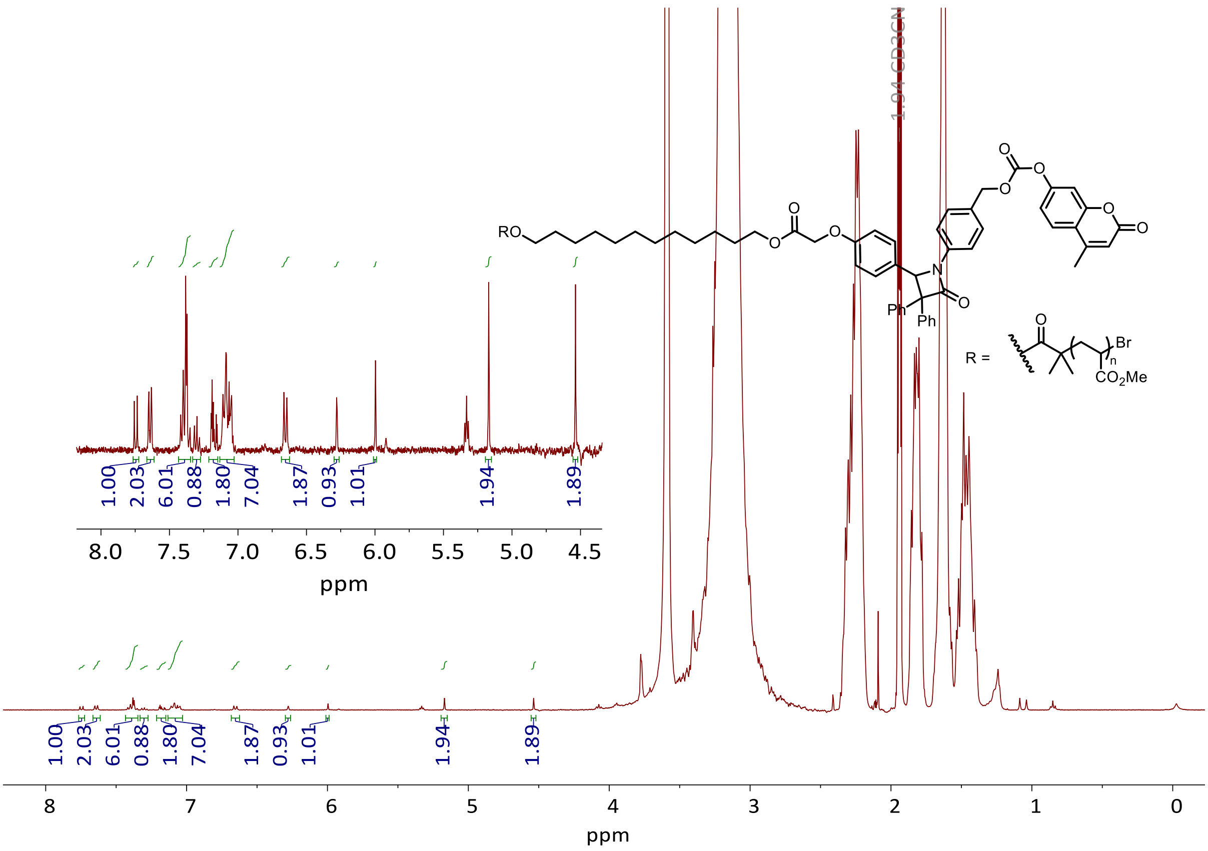

Spectrum S31. ^{1}H NMR (400 MHz, Acetonitrile-d_3/H_2O (9/1), 298 K) spectrum of polymer **S21**

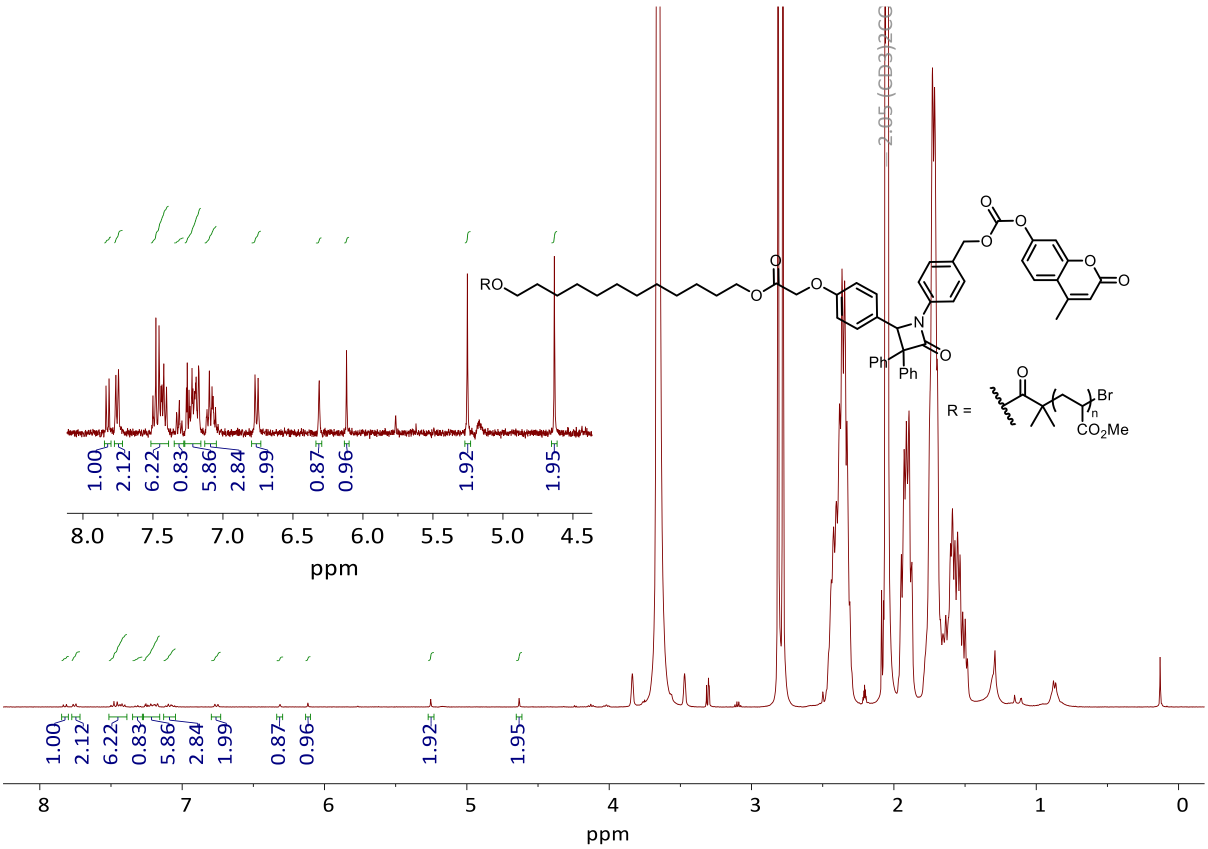

Spectrum S32. ^{1}H NMR (400 MHz, Acetone-d_6, 298 K) spectrum of polymer **S21**.

9.2.7 Spectra of polymer $\mathbf{9}_{\text{-112}}$

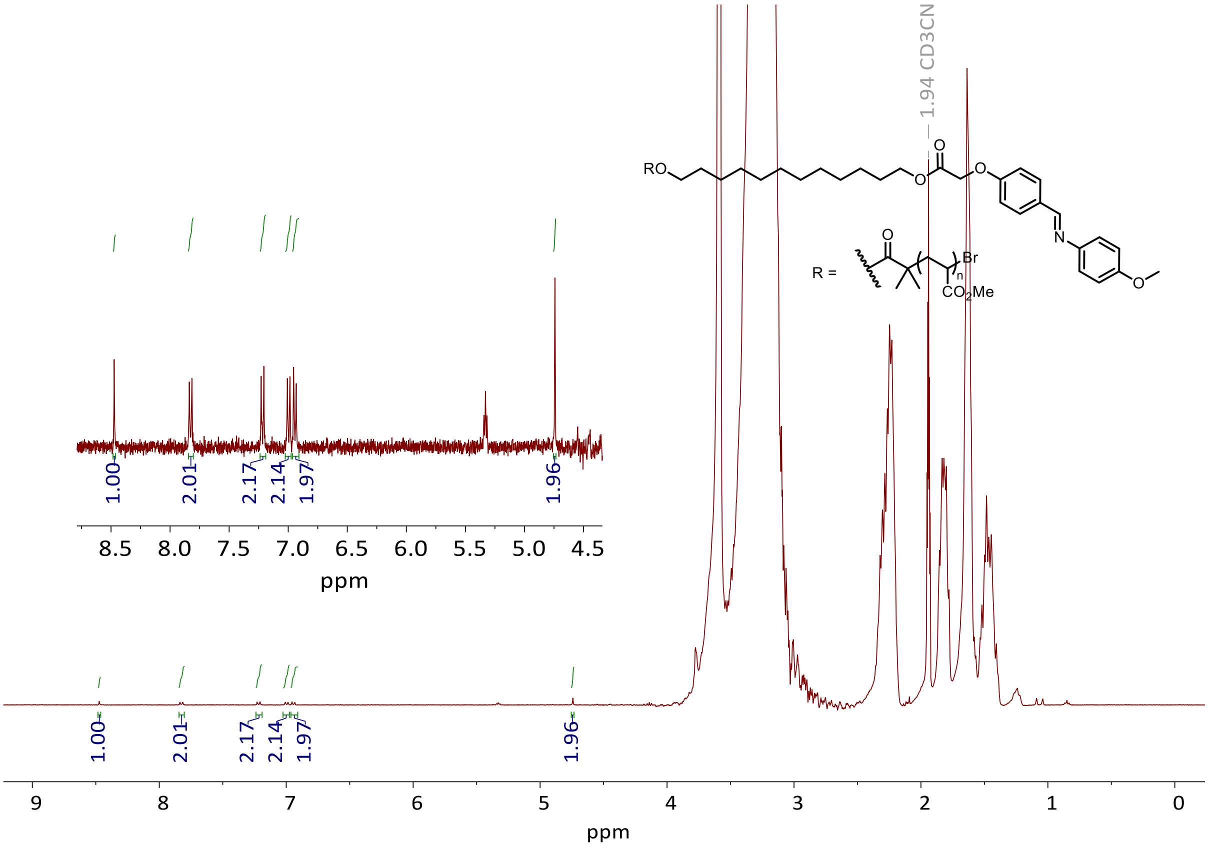

Spectrum S33. ^{1}H NMR (400 MHz, Acetonitrile-d_3/H_2O (9/1), 298 K) spectrum of polymer $\mathbf{9}_{\text{-112}}$

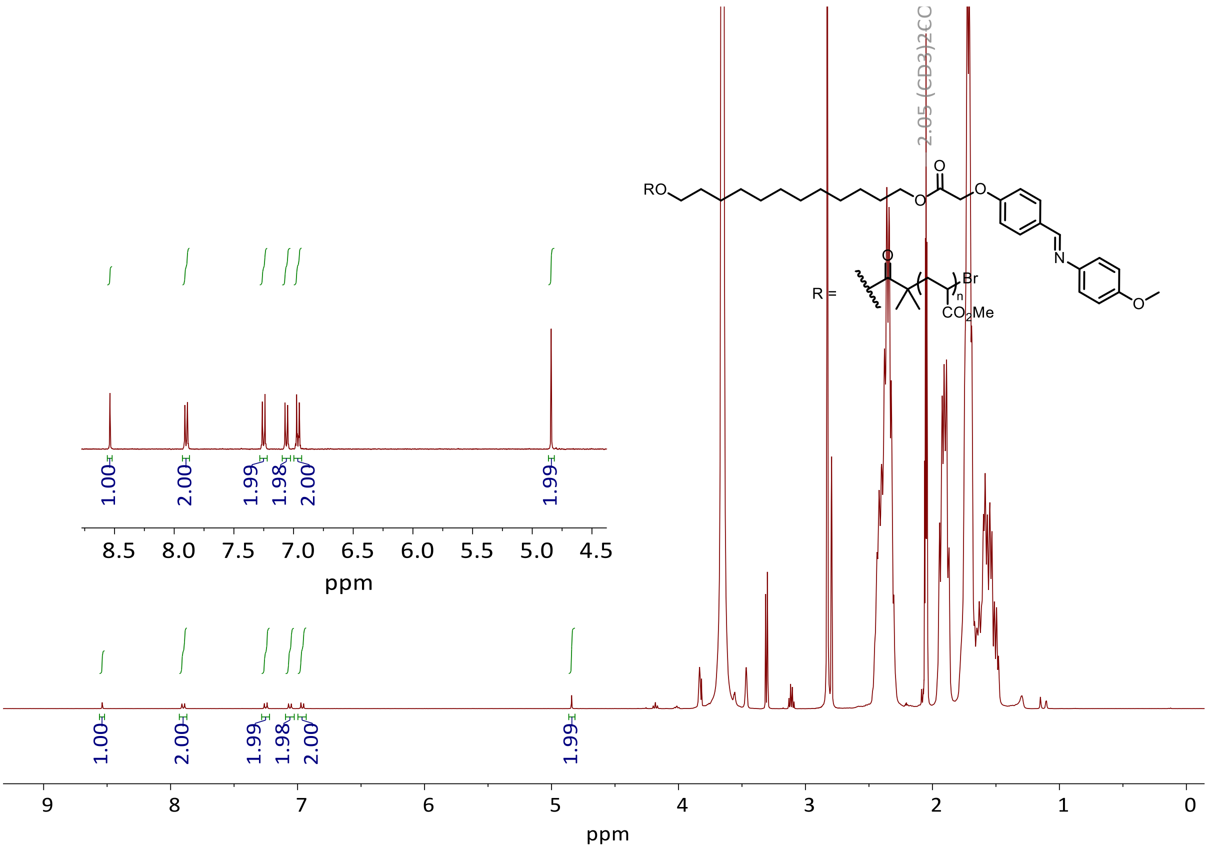

Spectrum S34. ^{1}H NMR (400 MHz, Acetone-d_6, 298 K) spectrum of polymer **9$_{-112}$**.

9.2.8 Spectra of polymer 9$_{-114}$

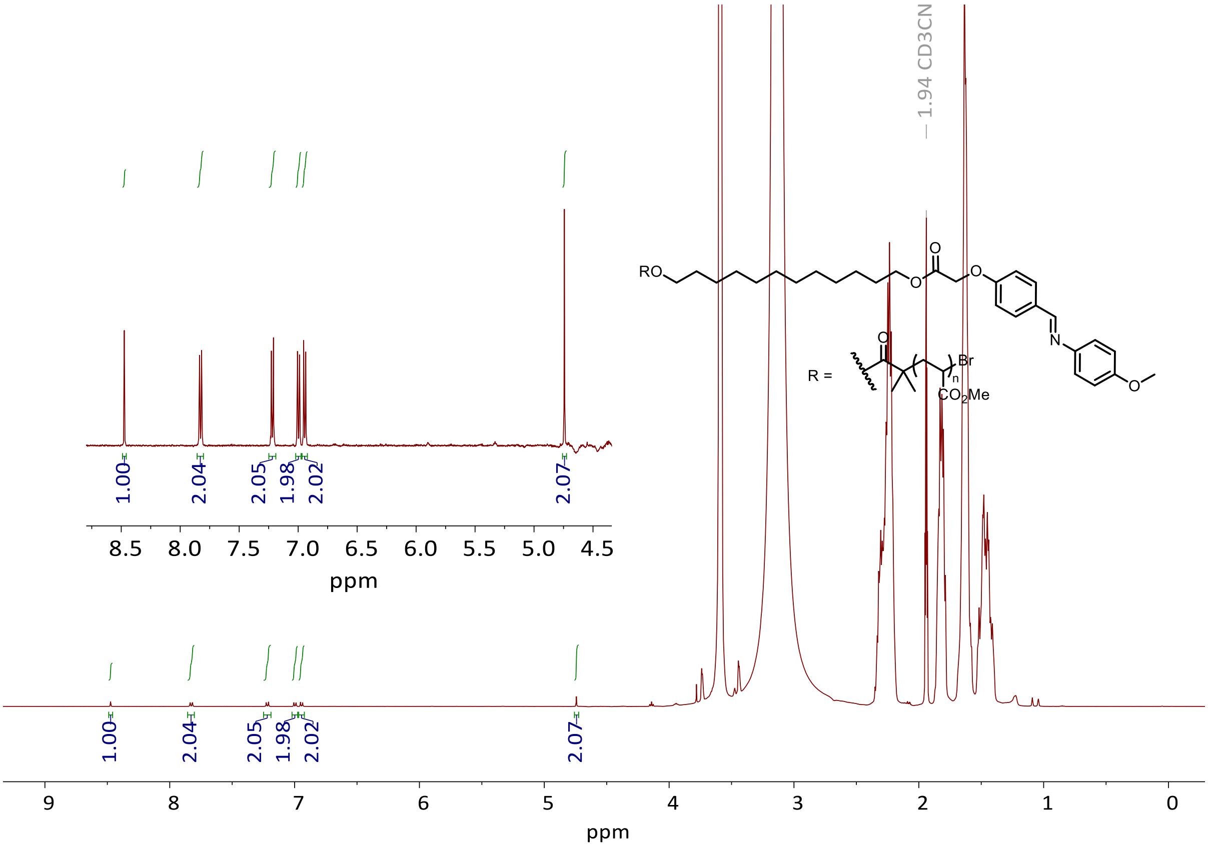

Spectrum S35. ^{1}H NMR (500 MHz, Acetonitrile-d_3/H_2O (9/1), 298 K) spectrum of polymer **9$_{-114}$**

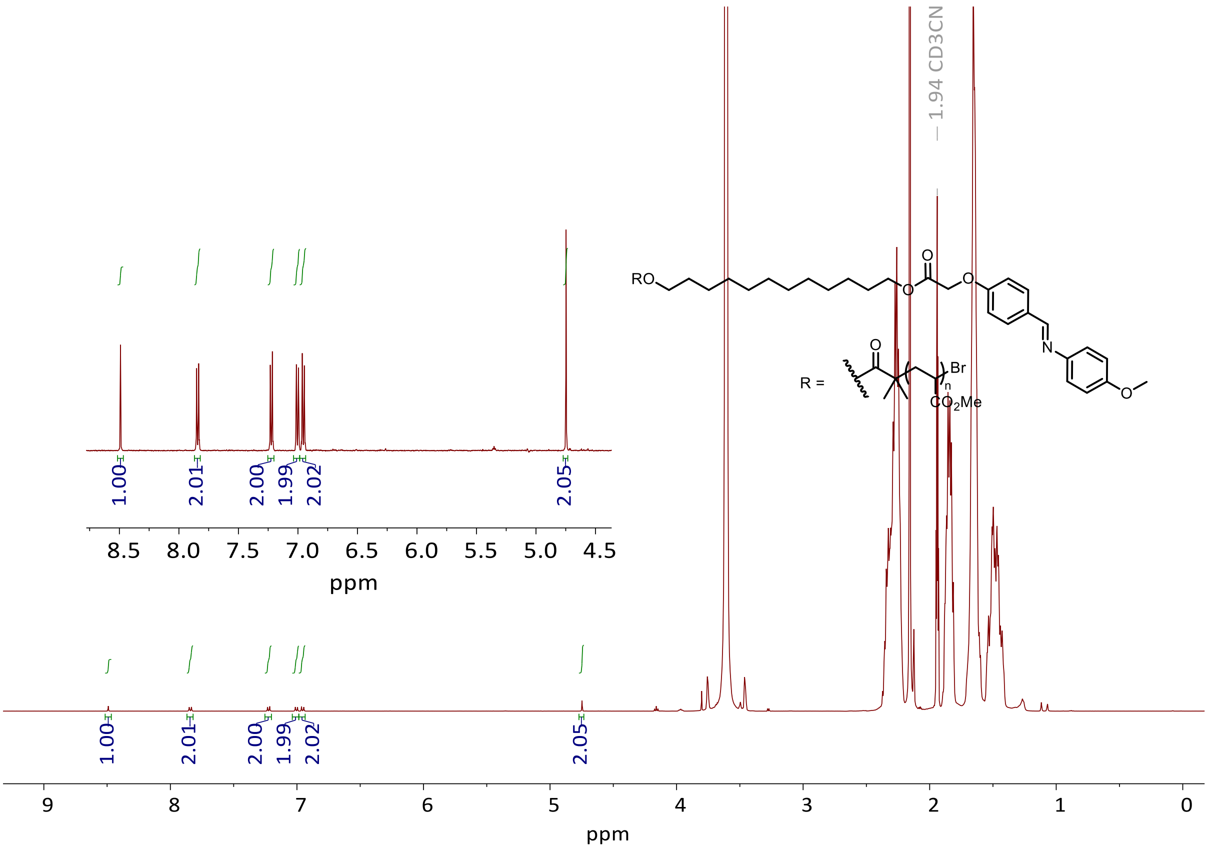

Spectrum S36. ^{1}H NMR (500 MHz, Acetonitrile-d_3, 298 K) spectrum of polymer **9$_{-114}$**.

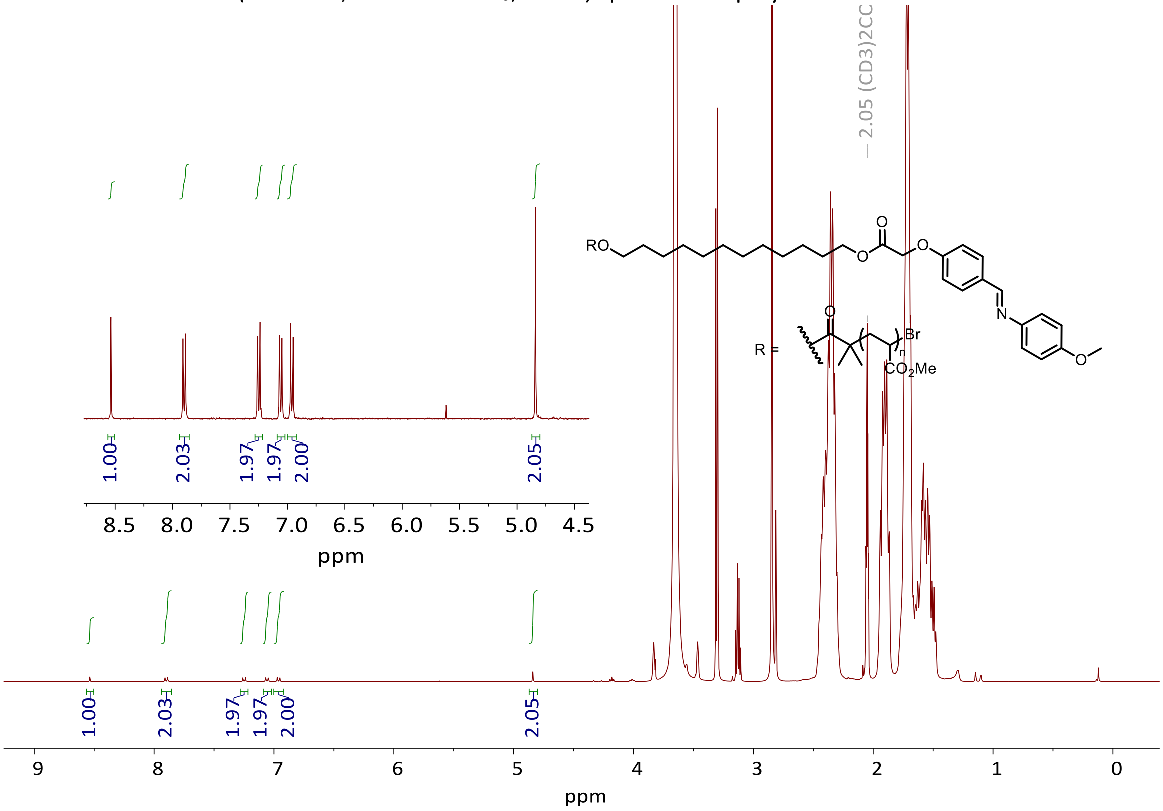

Spectrum S37. ^{1}H NMR (400 MHz, Acetone-d_6, 298 K) spectrum of polymer **9$_{-114}$**.

9.2.9 Spectra of polymer 10

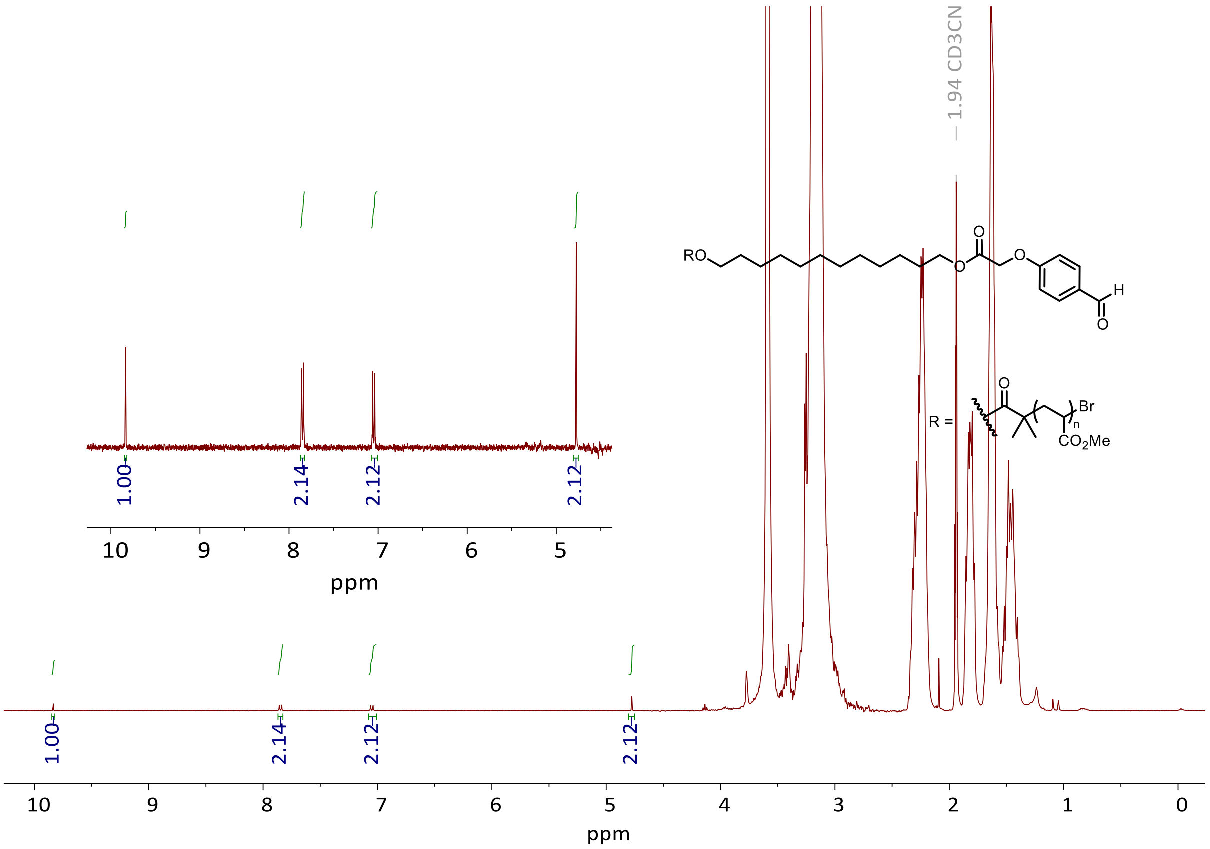

Spectrum S38. ^{1}H NMR (400 MHz, Acetonitrile-d_3/H_2O (9/1), 298 K) spectrum of polymer **10**

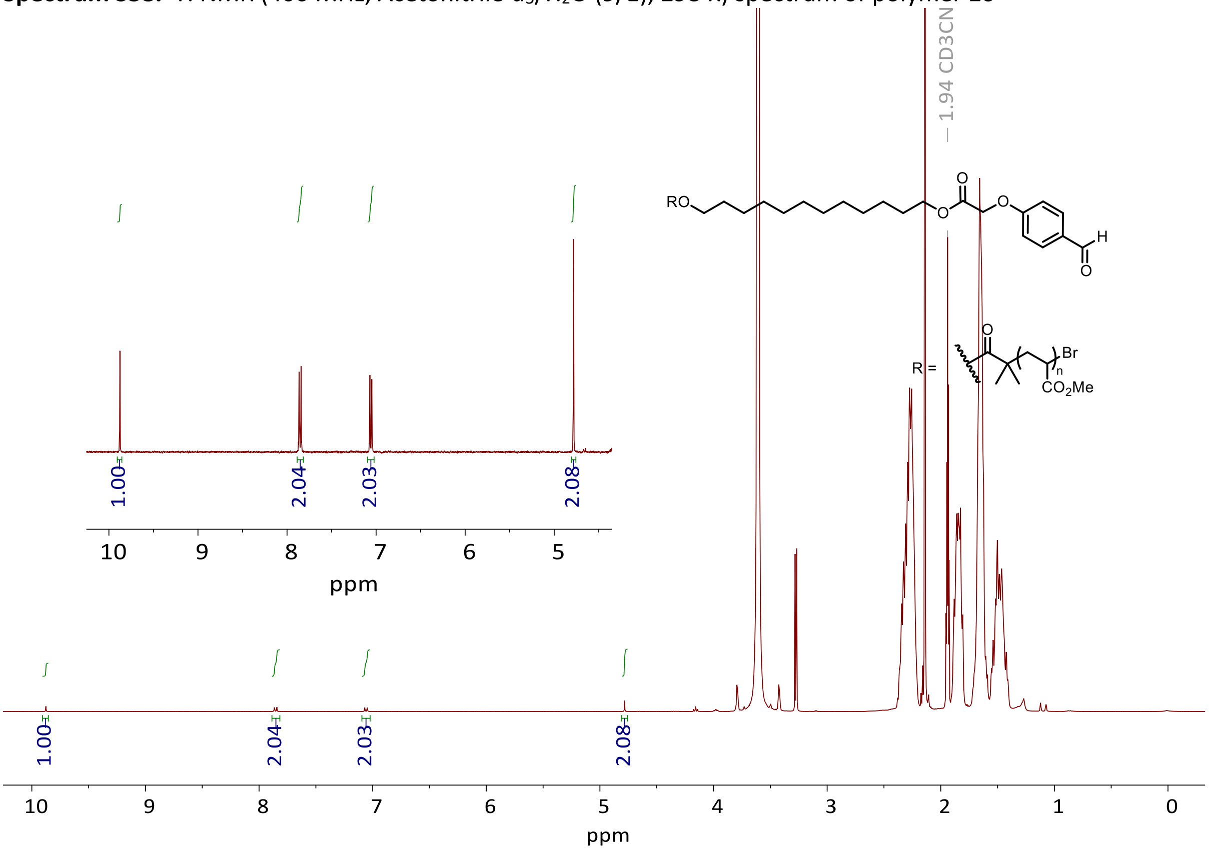

Spectrum S39. ^{1}H NMR (400 MHz, Acetonitrile-d_3, 298 K) spectrum of polymer **10**.

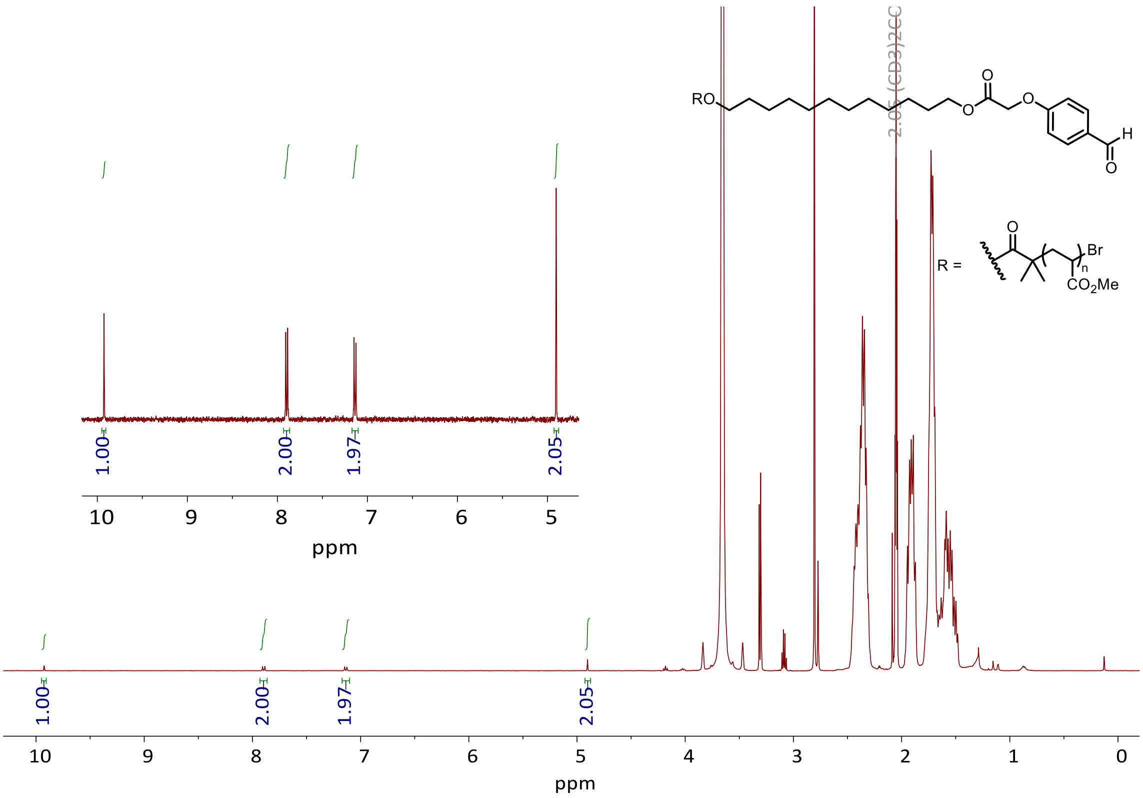

Spectrum S40. ^{1}H NMR (400 MHz, Acetone-d_6, 298 K) spectrum of polymer **10**.

9.3 Post-Sonication NMR Spectra

9.3.1 Post-Sonication ^1H NMR Spectra of Polymer 5-138 (Run 1)

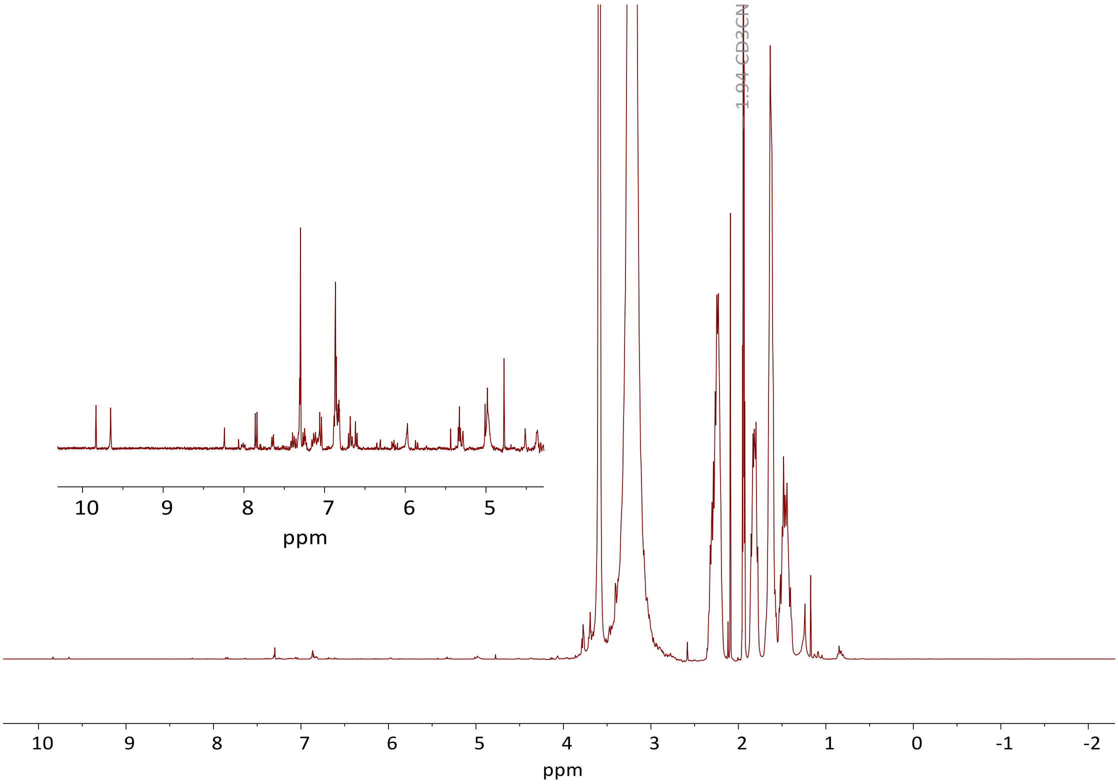

Spectrum S41. ^1H NMR (400 MHz, Acetonitrile-d_3/H_2O (9/1), 298 K) spectrum of post-sonication polymer **5**-138 before being washed with methanol.

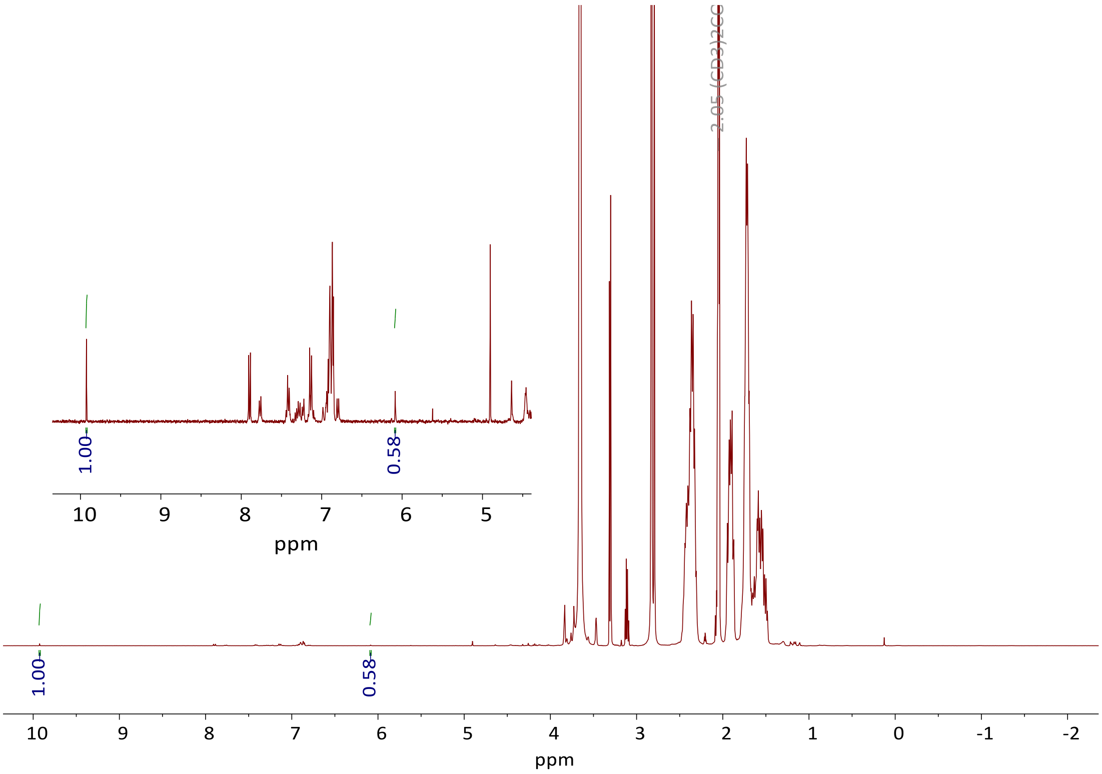

Spectrum S42. ^1H NMR (400 MHz, Acetone-d_6, 298 K) spectrum of post-sonication polymer **5**-138 after being washed with methanol.

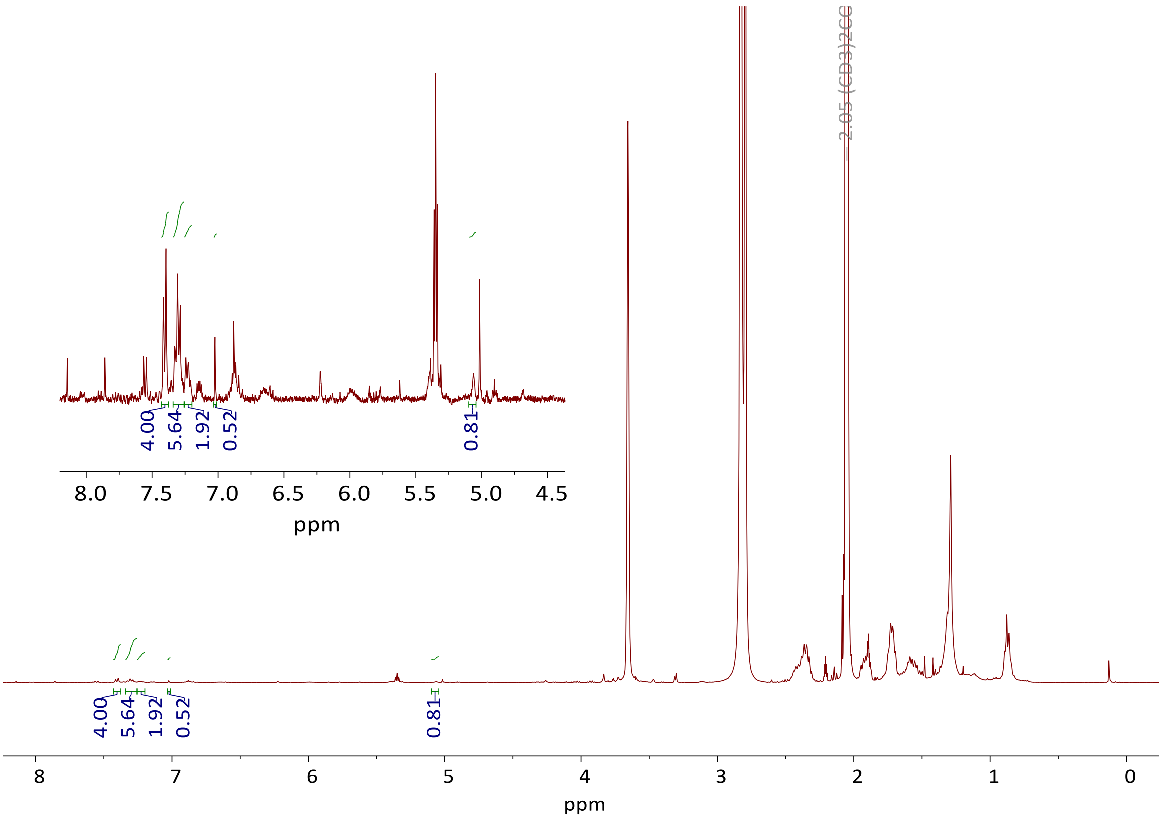

Spectrum S43. ^{1}H NMR (400 MHz, Acetone-d_6, 298 K) spectrum of the concentrated methanol washings from post-sonication polymer $\mathbf{5}_{\text{-}138}$.

9.3.2 Post-Sonication ^{1}H NMR Spectra of Polymer $\mathbf{5}_{\text{-}138}$ (Run 2)

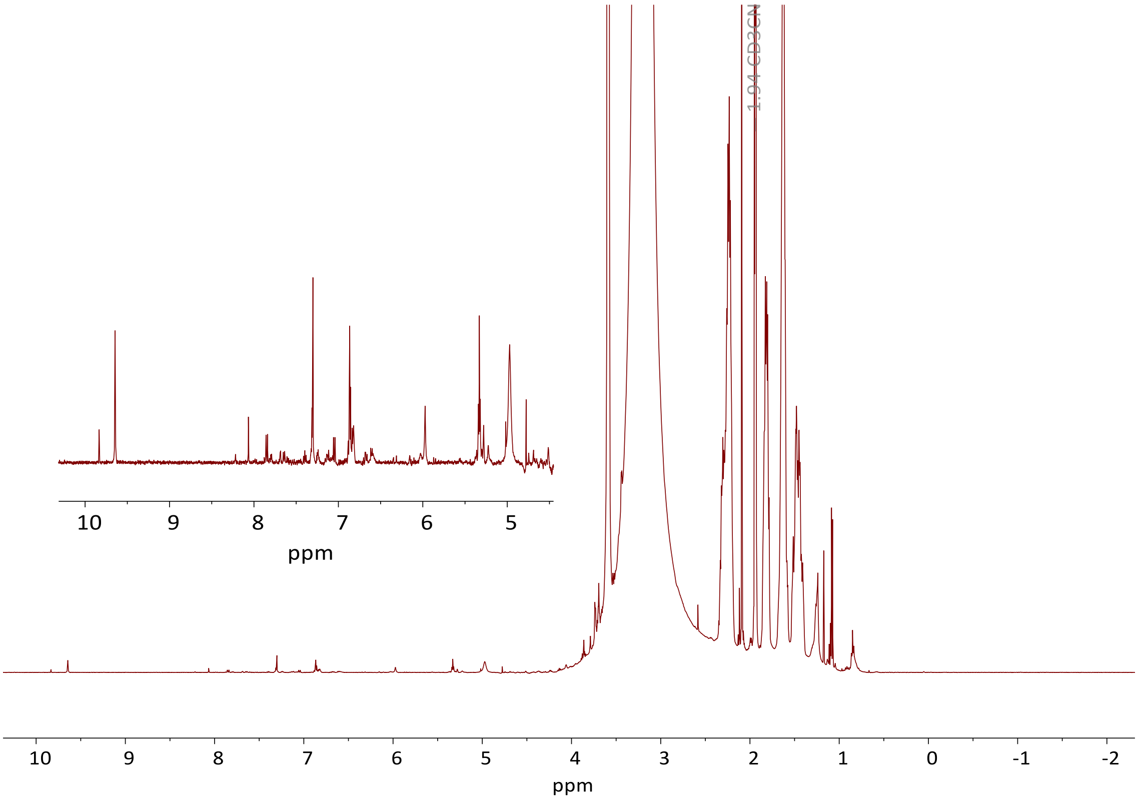

Spectrum S44. ^{1}H NMR (500 MHz, Acetonitrile-d_3/H_2O (9/1), 298 K) spectrum of post-sonication polymer $\mathbf{5}_{\text{-}138}$ before being washed with methanol.

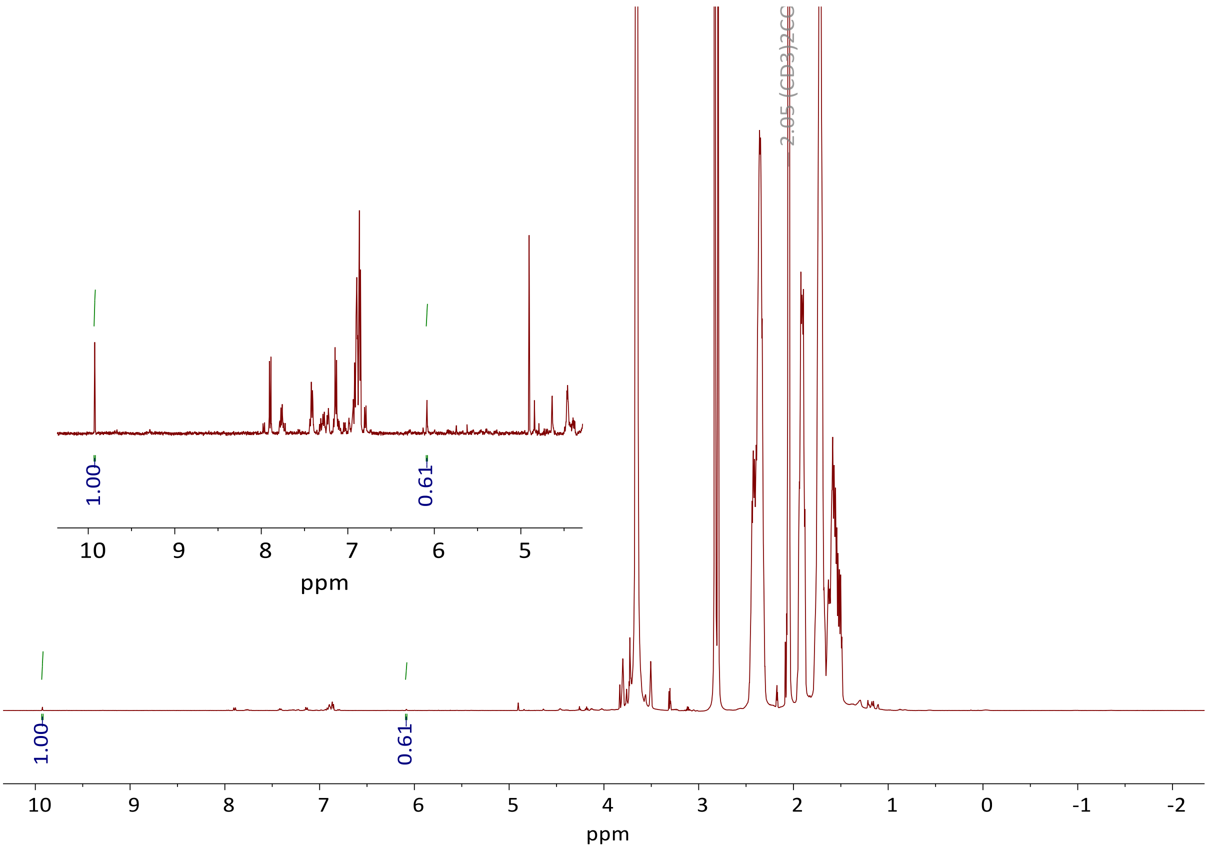

Spectrum S45. ^{1}H NMR (500 MHz, Acetone-d_6, 298 K) spectrum of post-sonication polymer **5**$_{\mathbf{-138}}$ after being washed with methanol.

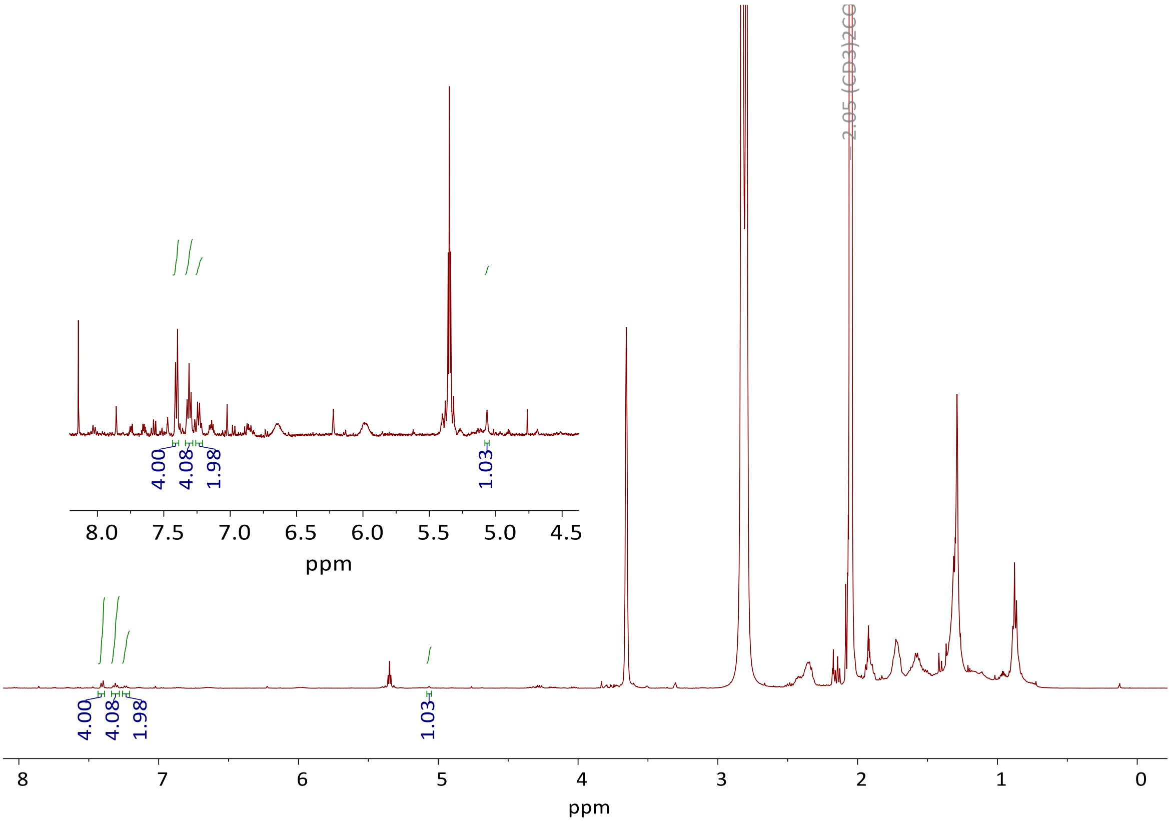

Spectrum S46. ^{1}H NMR (500 MHz, Acetone-d_6, 298 K) spectrum of the concentrated methanol washings from post-sonication polymer **5**$_{\mathbf{-138}}$.

9.3.3 Post-Sonication ^{1}H NMR Spectra of Polymer $5_{\text{-}155}$ (Run 1)

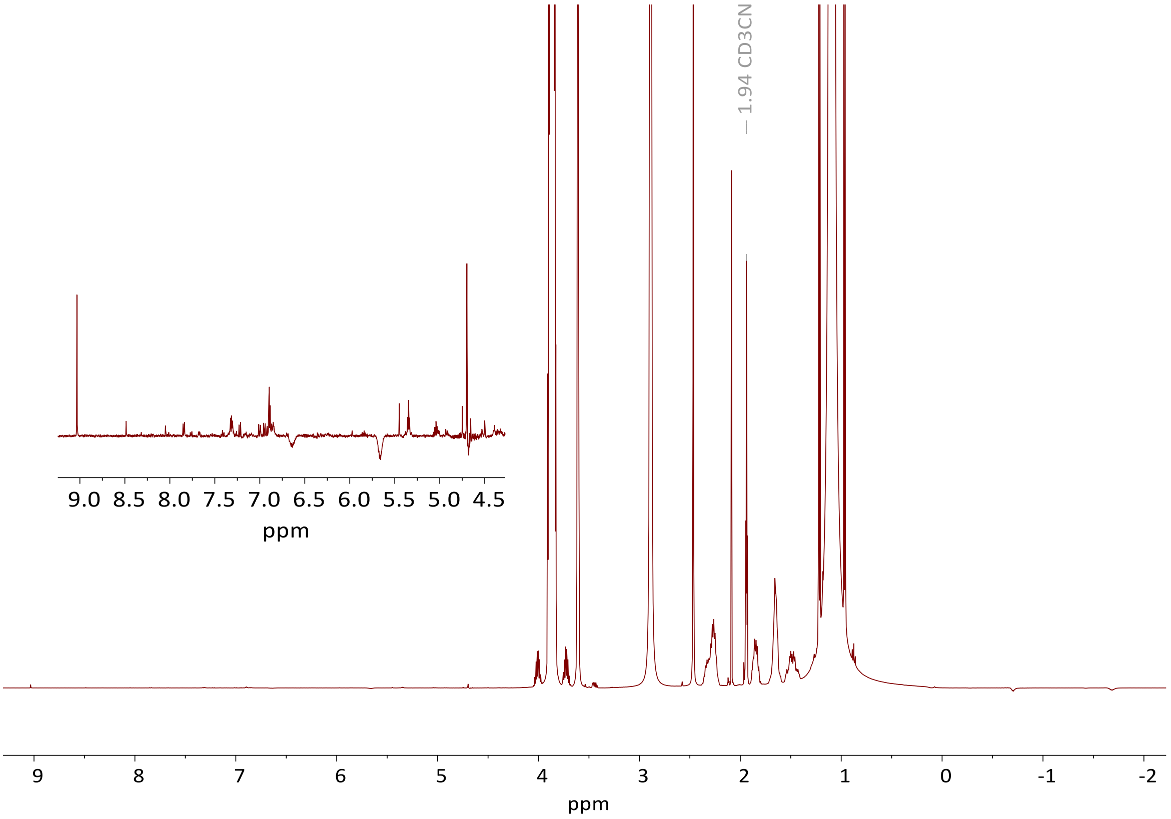

Spectrum S47. ^{1}H NMR (500 MHz, Acetonitrile-d_3/*i*PrOH (9/1), 298 K) spectrum of post-sonication polymer **$5_{\text{-}155}$** before being washed with methanol.

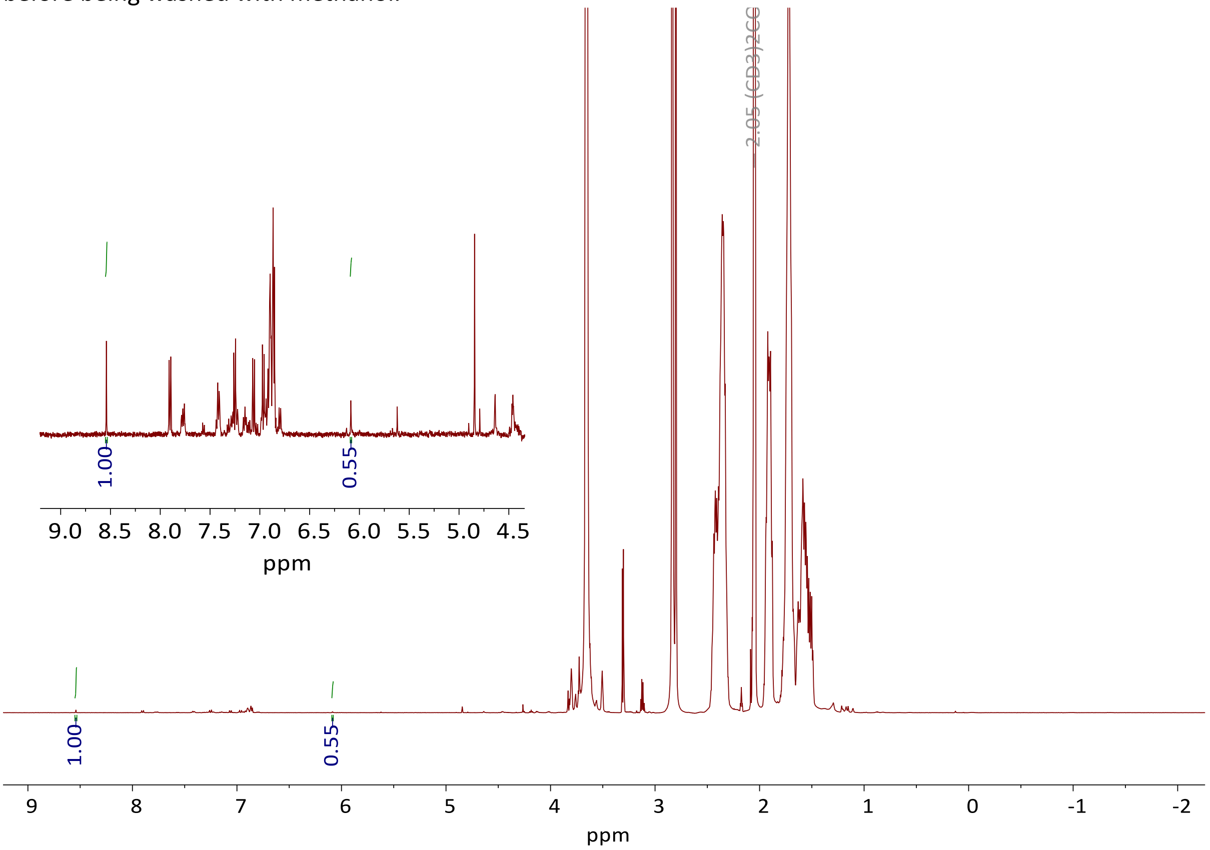

Spectrum S48. ^{1}H NMR (500 MHz, Acetone-d_6, 298 K) spectrum of post-sonication polymer **$5_{\text{-}155}$** after being washed with methanol.

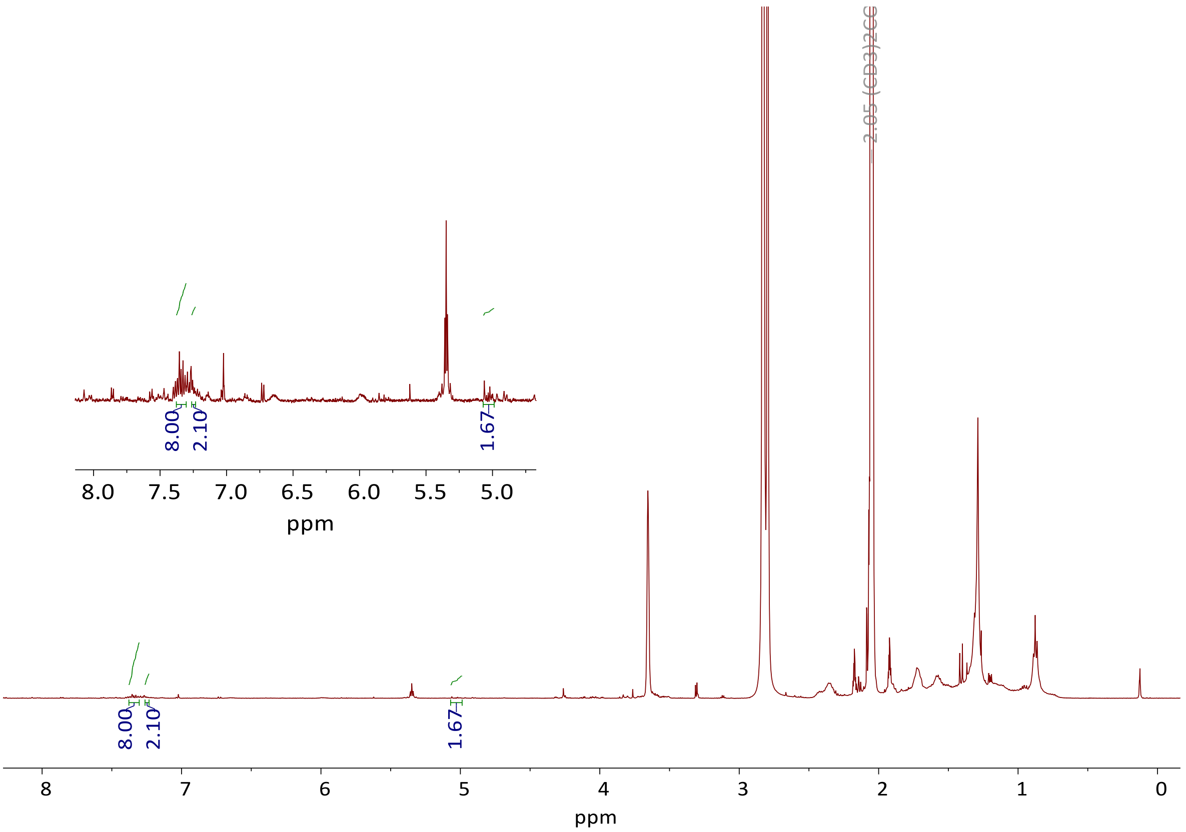

Spectrum S49. ^{1}H NMR (500 MHz, Acetone-d_6, 298 K) spectrum of the concentrated methanol washings from post-sonication polymer **5**$_{\text{-155}}$.

9.3.4 Post-Sonication ^{1}H NMR Spectra of Polymer 5$_{\text{-155}}$ (Run 2)

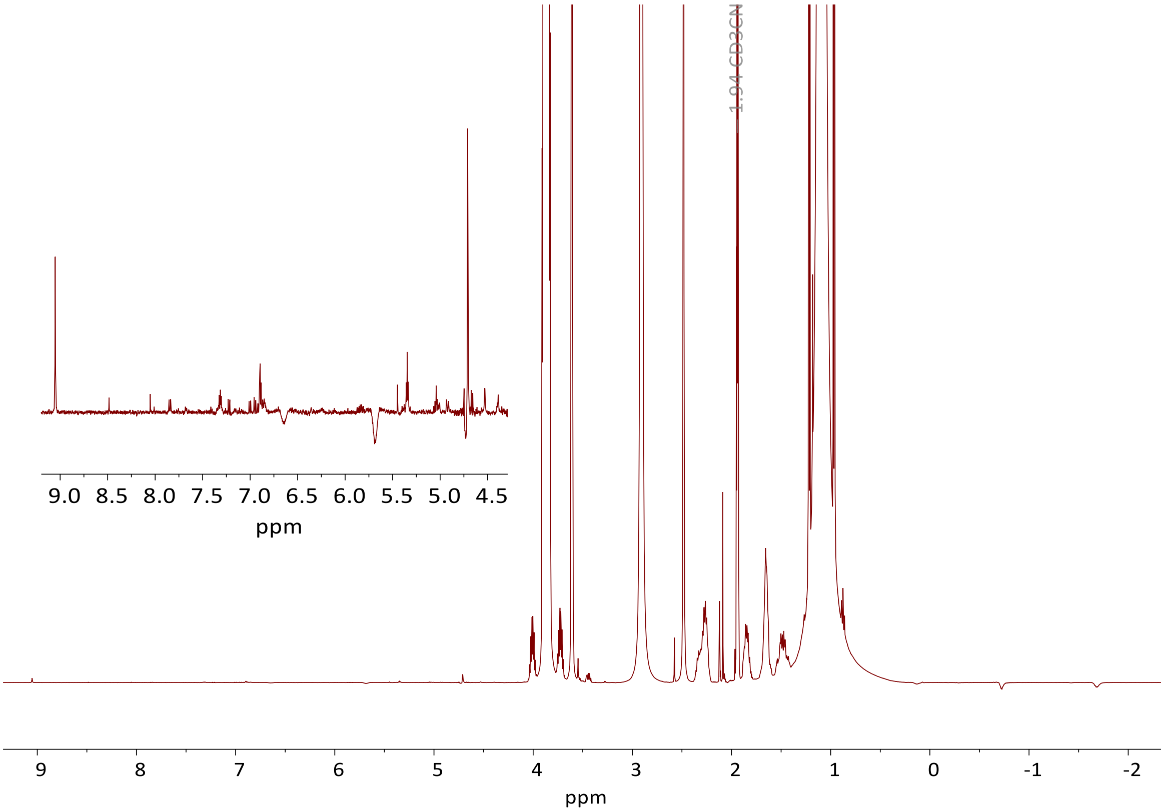

Spectrum S50. ^{1}H NMR (500 MHz, Acetonitrile-d_3/*i*PrOH (9/1), 298 K) spectrum of post-sonication polymer **5**$_{\text{-155}}$ before being washed with methanol.

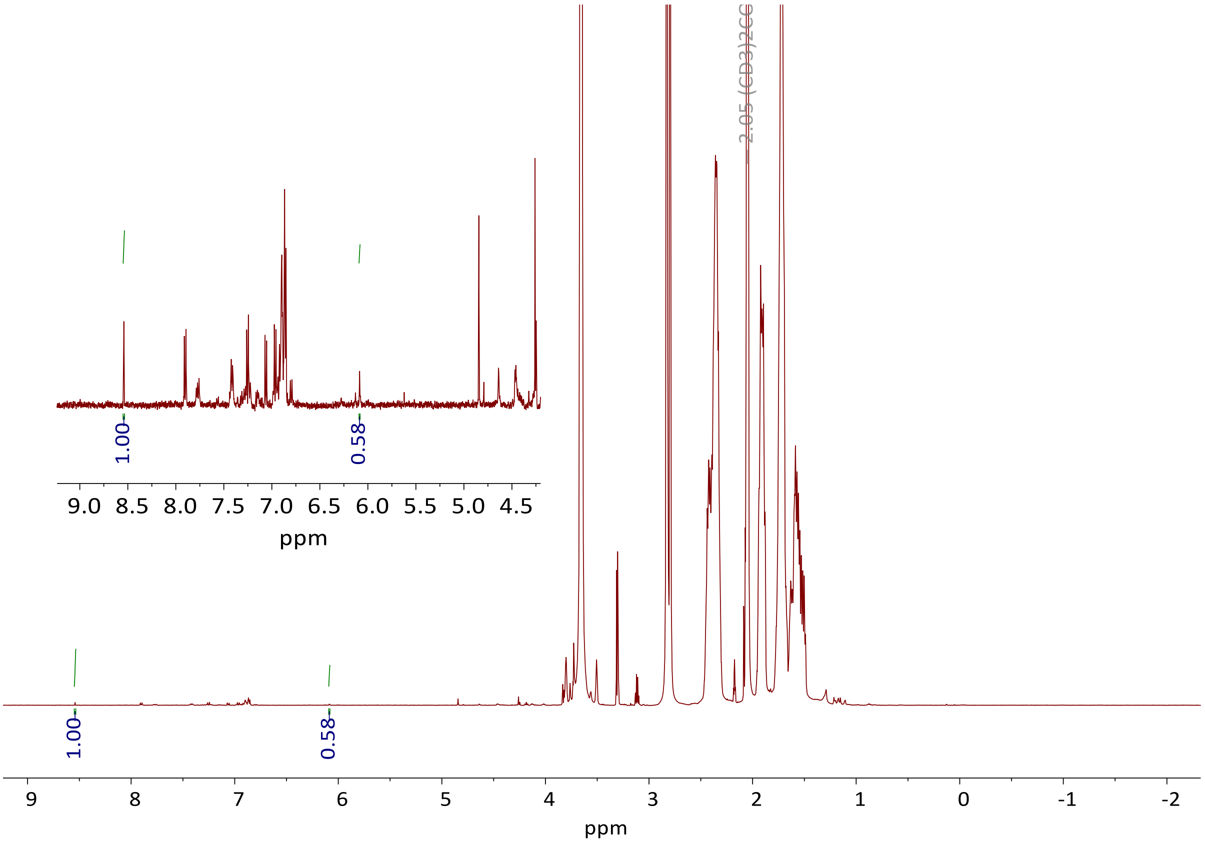

Spectrum S51. ^{1}H NMR (500 MHz, Acetone-d_6, 298 K) spectrum of post-sonication polymer $\mathbf{5}_{\text{-}155}$ after being washed with methanol.

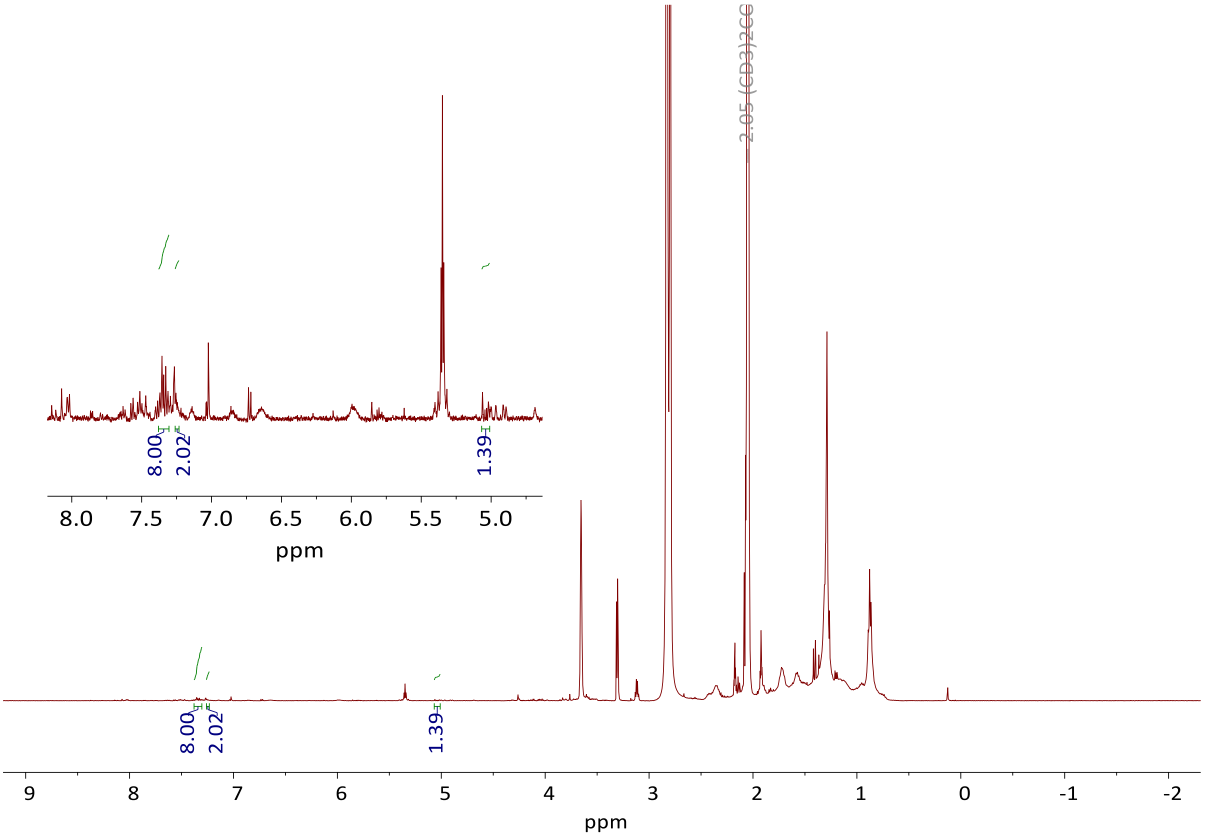

Spectrum S52. ^{1}H NMR (500 MHz, Acetone-d_6, 298 K) spectrum of the concentrated methanol washings from post-sonication polymer $\mathbf{5}_{\text{-}155}$.

9.3.5 Post-Sonication ^{1}H NMR Spectra of Polymer 1_{HYM} (Run 1)

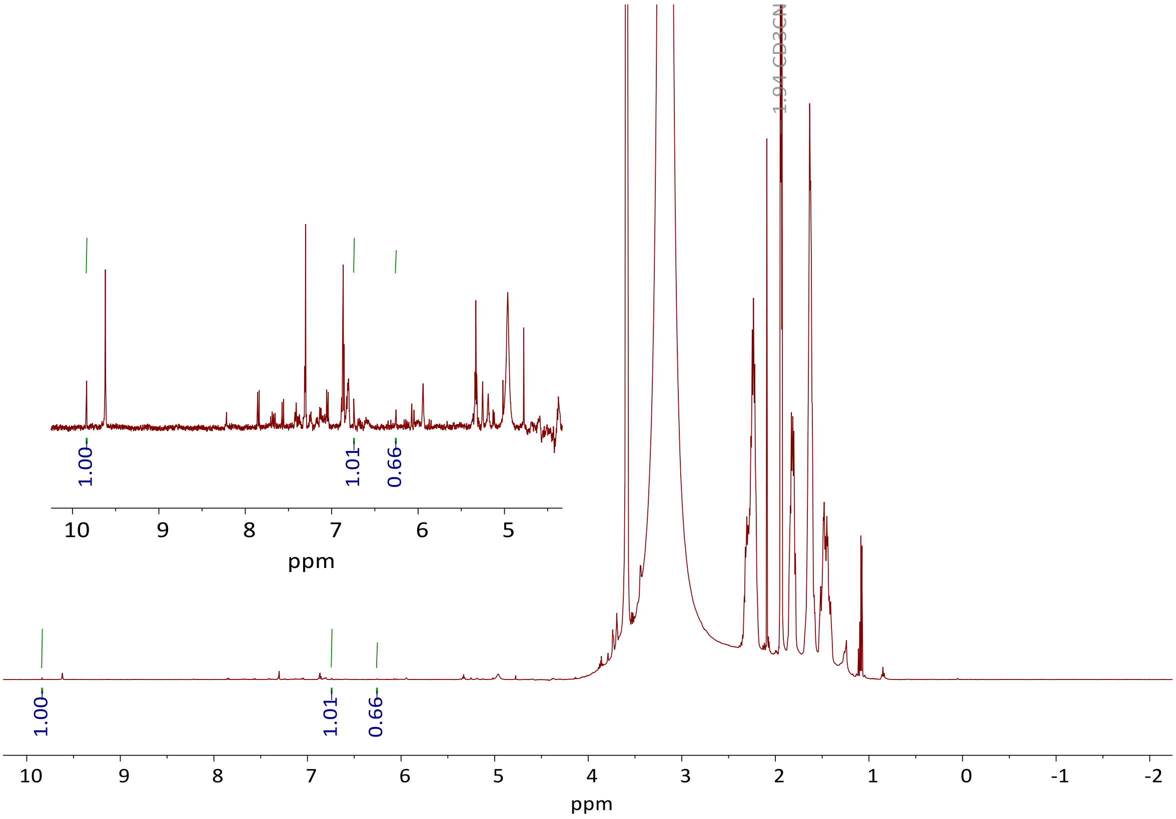

Spectrum S53. ^{1}H NMR (500 MHz, Acetonitrile-d_3/H_2O (9/1), 298 K) spectrum of post-sonication polymer $\mathbf{1}_{HYM}$ before being washed with methanol.

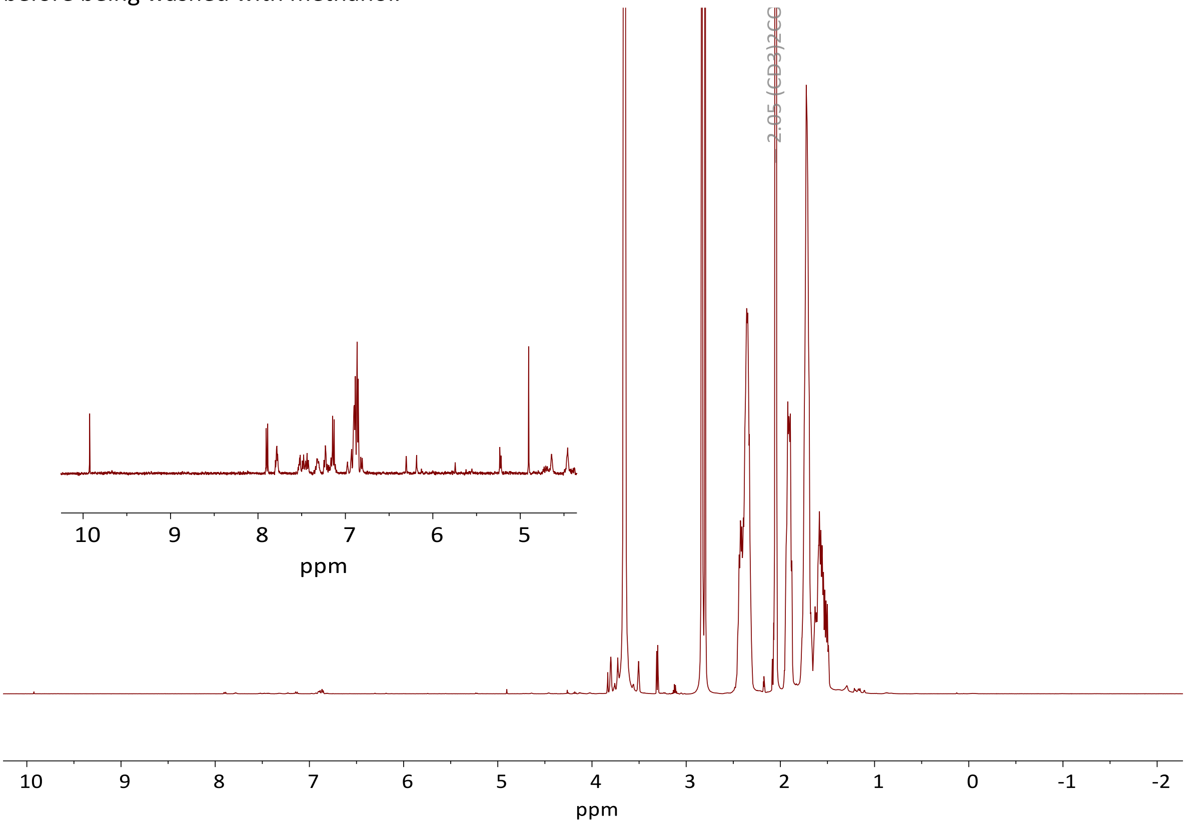

Spectrum S54. ^{1}H NMR (500 MHz, Acetone-d_6, 298 K) spectrum of post-sonication polymer $\mathbf{1}_{HYM}$ after being washed with methanol.

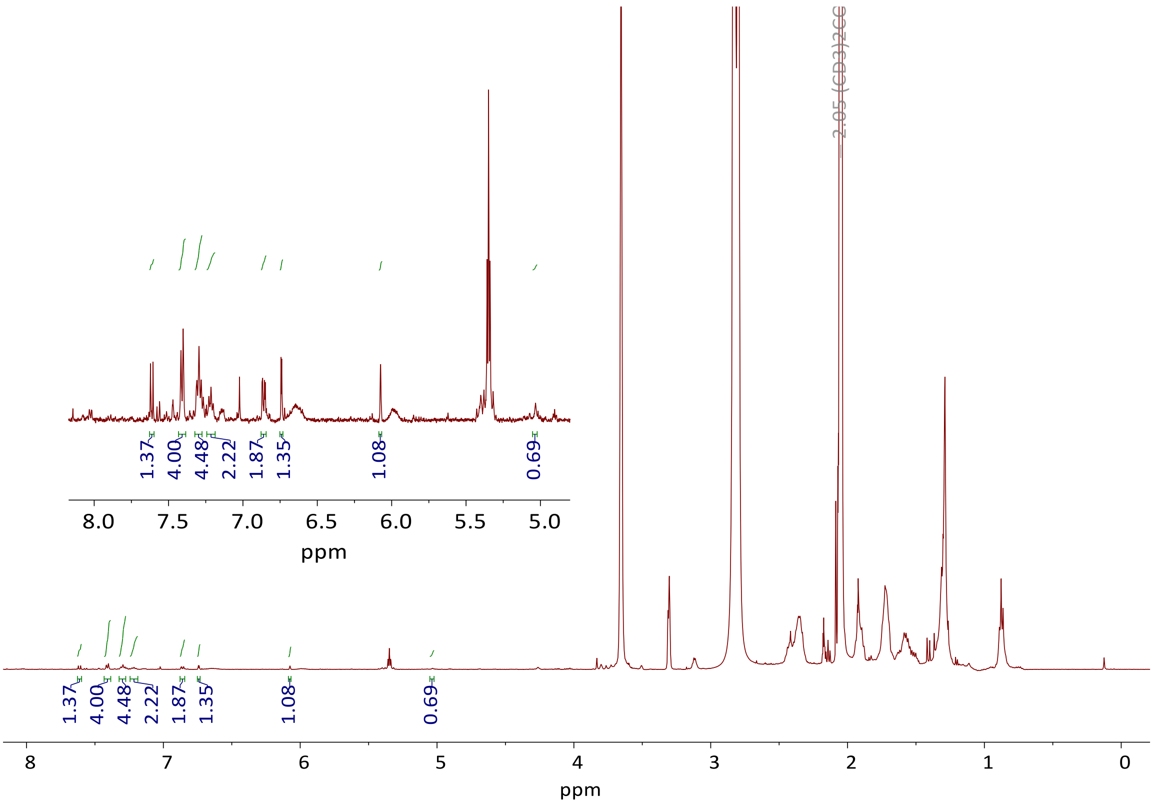

Spectrum S55. ^{1}H NMR (500 MHz, Acetone-d_6, 298 K) spectrum of the concentrated methanol washings from post-sonication polymer $\mathbf{1}_{HYM}$.

9.3.6 Post-Sonication ^{1}H NMR Spectra of Polymer $\mathbf{1}_{HYM}$ (Run 2)

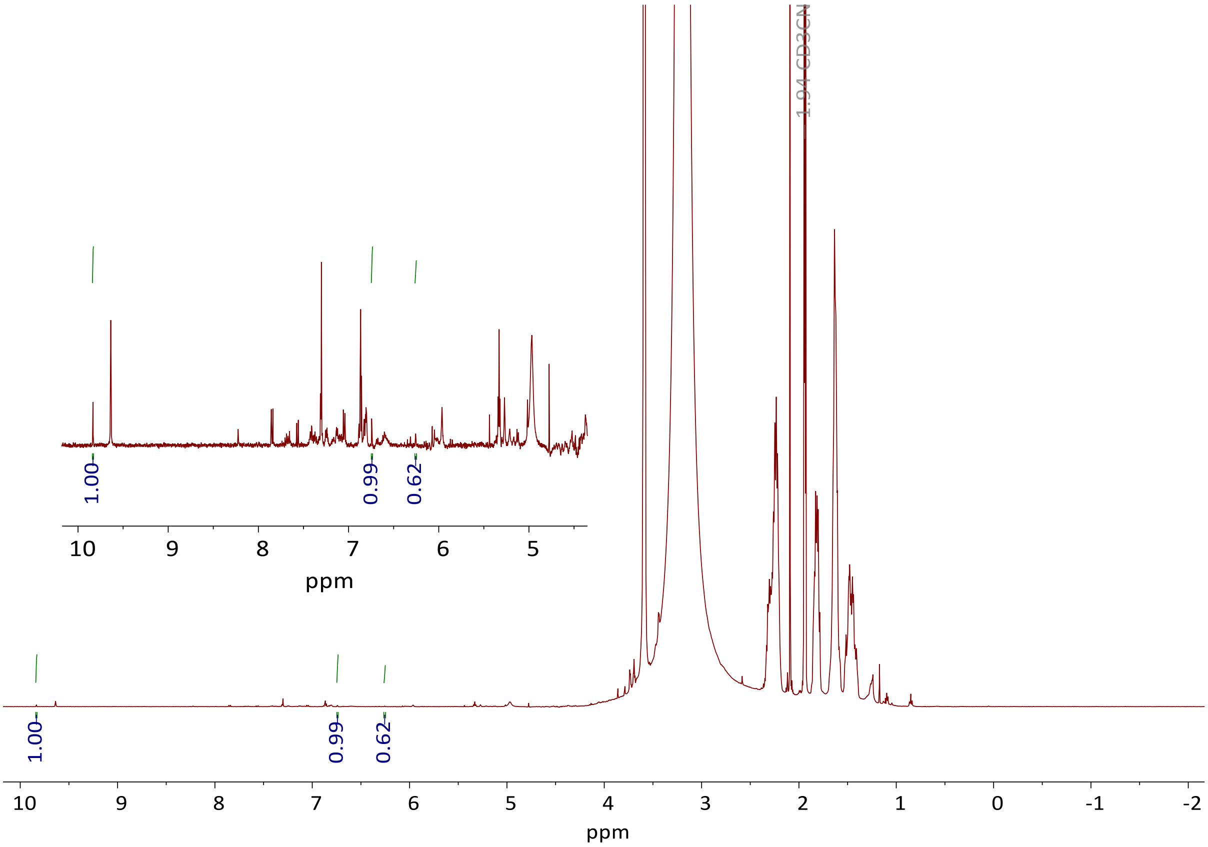

Spectrum S56. ^{1}H NMR (500 MHz, Acetonitrile-d_3/H_2O (9/1), 298 K) spectrum of post-sonication polymer $\mathbf{1}_{HYM}$ before being washed with methanol.

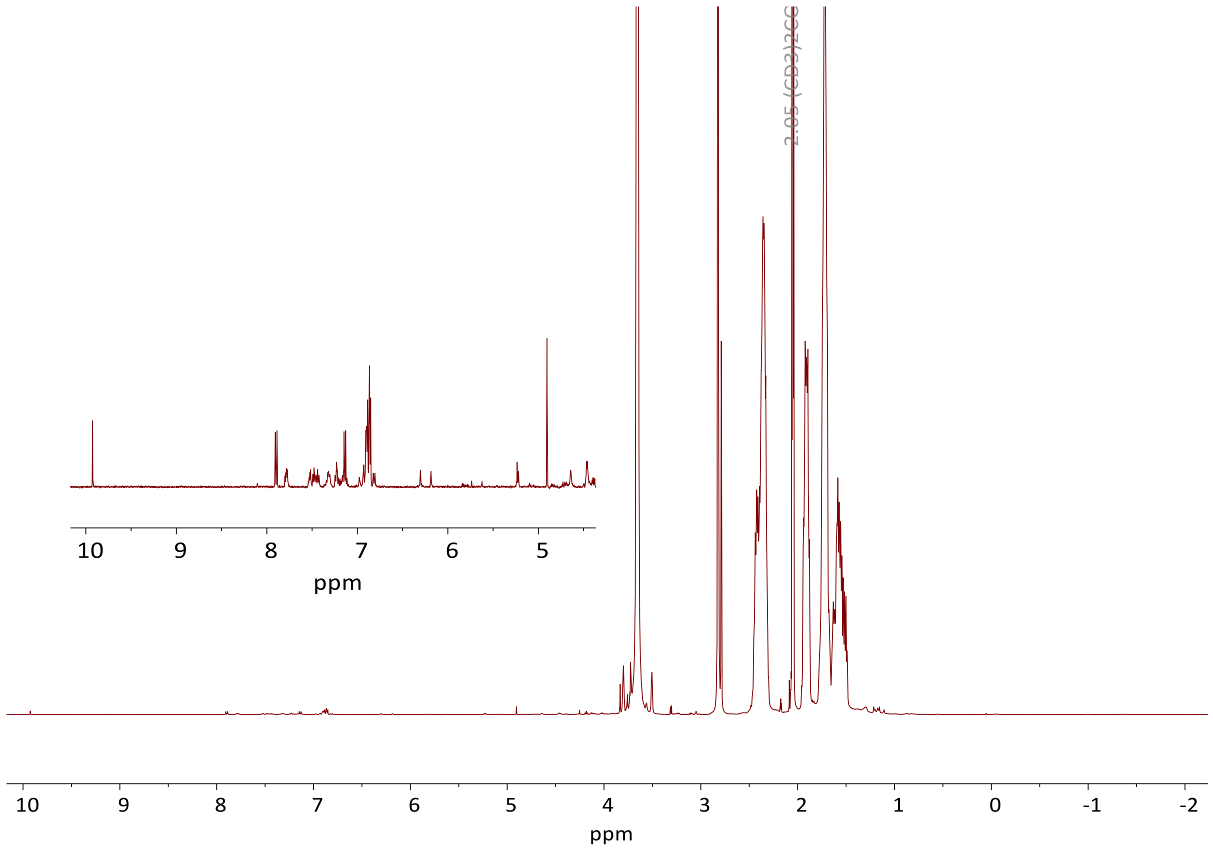

Spectrum S57. ^{1}H NMR (500 MHz, Acetone-d_6, 298 K) spectrum of post-sonication polymer **1*HYM*** after being washed with methanol.

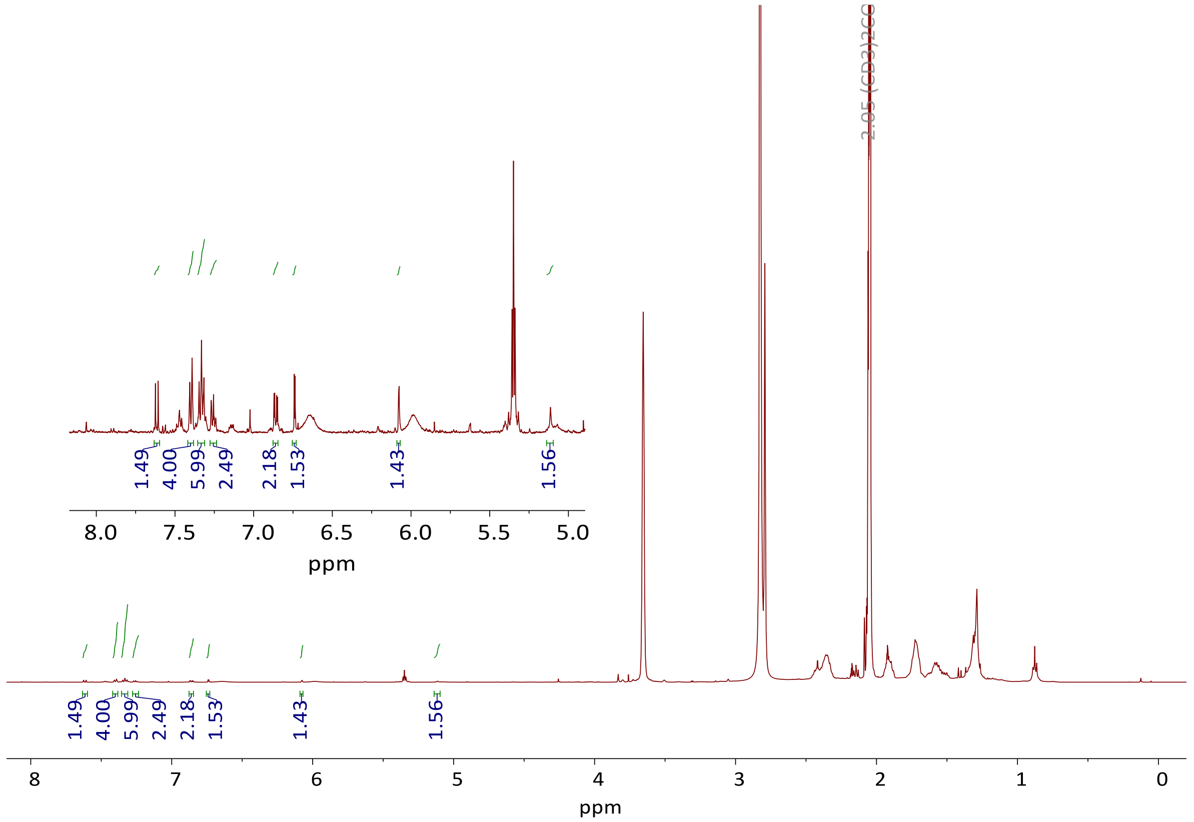

Spectrum S58. ^{1}H NMR (500 MHz, Acetone-d_6, 298 K) spectrum of the concentrated methanol washings from post-sonication polymer **1*HYM***.

9.3.7 Post-Sonication ^{1}H NMR Spectra of Polymer $\mathbf{1}_{GEM}$ (Run 1)

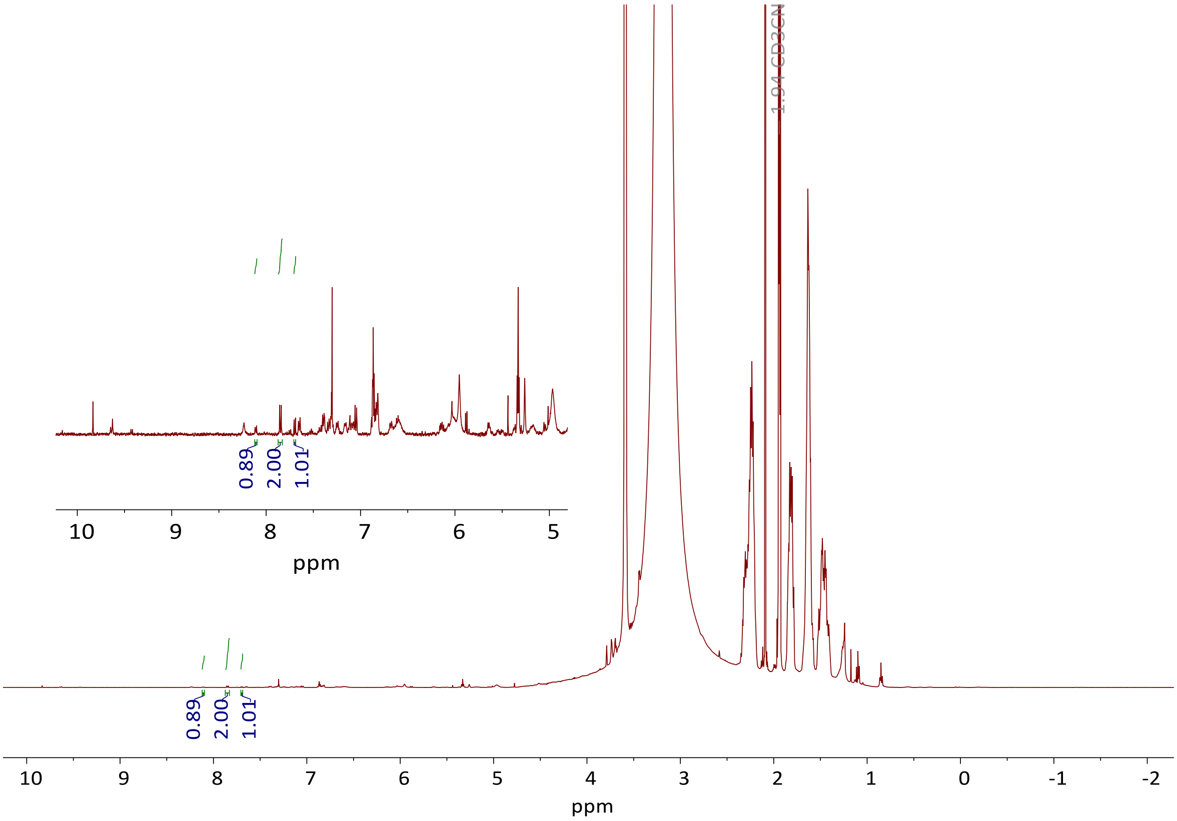

Spectrum S59. ^{1}H NMR (500 MHz, Acetonitrile-d_3/H_2O (9/1), 298 K) spectrum of post-sonication polymer $\mathbf{1}_{GEM}$ before being washed with methanol.

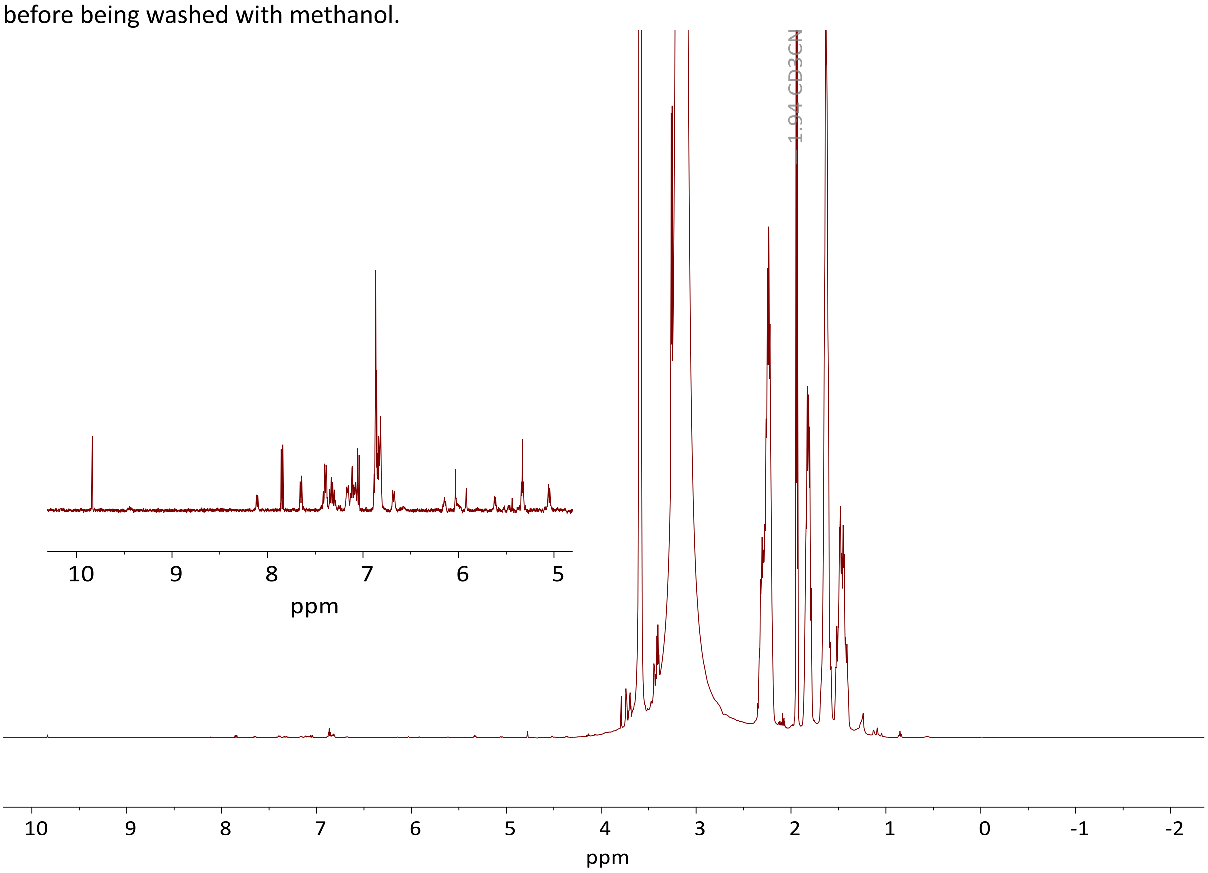

Spectrum S60. ^{1}H NMR (500 MHz, Acetonitrile-d_3/H_2O (9/1), 298 K) spectrum of post-sonication polymer $\mathbf{1}_{GEM}$ after being washed with methanol.

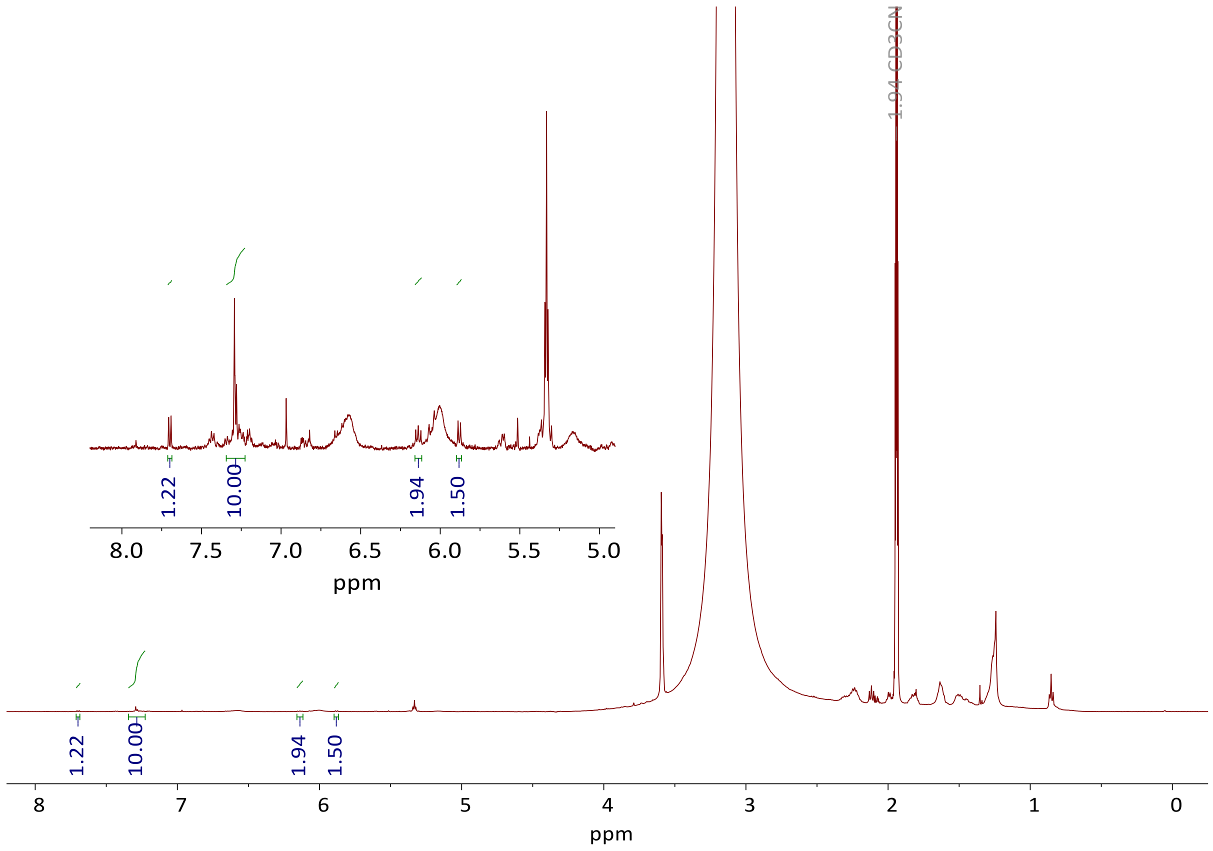

Spectrum S61. ^{1}H NMR (500 MHz, Acetonitrile-d_3/H_2O (9/1), 298 K) spectrum of the concentrated methanol washings from post-sonication polymer **1*GEM***.

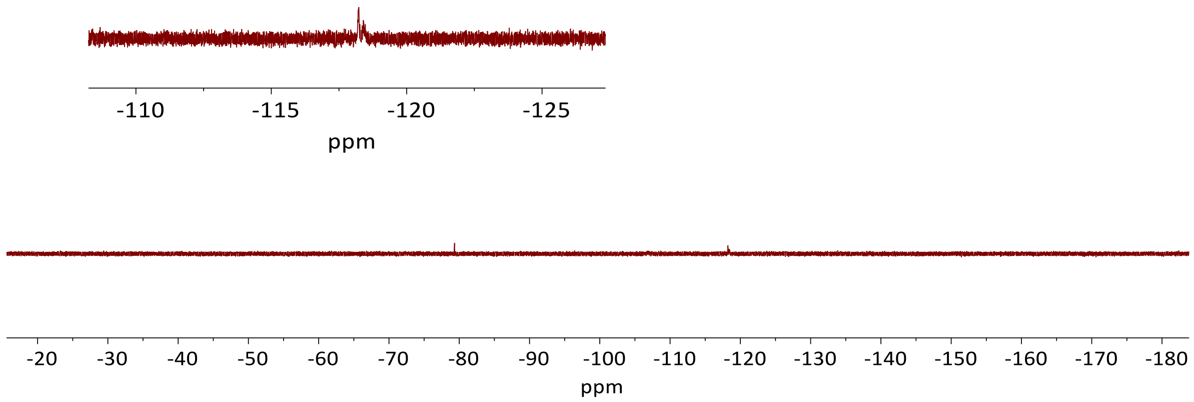

Spectrum S62. ^{19}F NMR (471 MHz, Acetonitrile-d_6/H_2O (9/1), 298 K) spectrum of the concentrated methanol washings from post-sonication polymer **1*GEM***.

9.3.8 Post-Sonication ^{1}H NMR Spectra of Polymer $\mathbf{1}_{GEM}$ (Run 2)

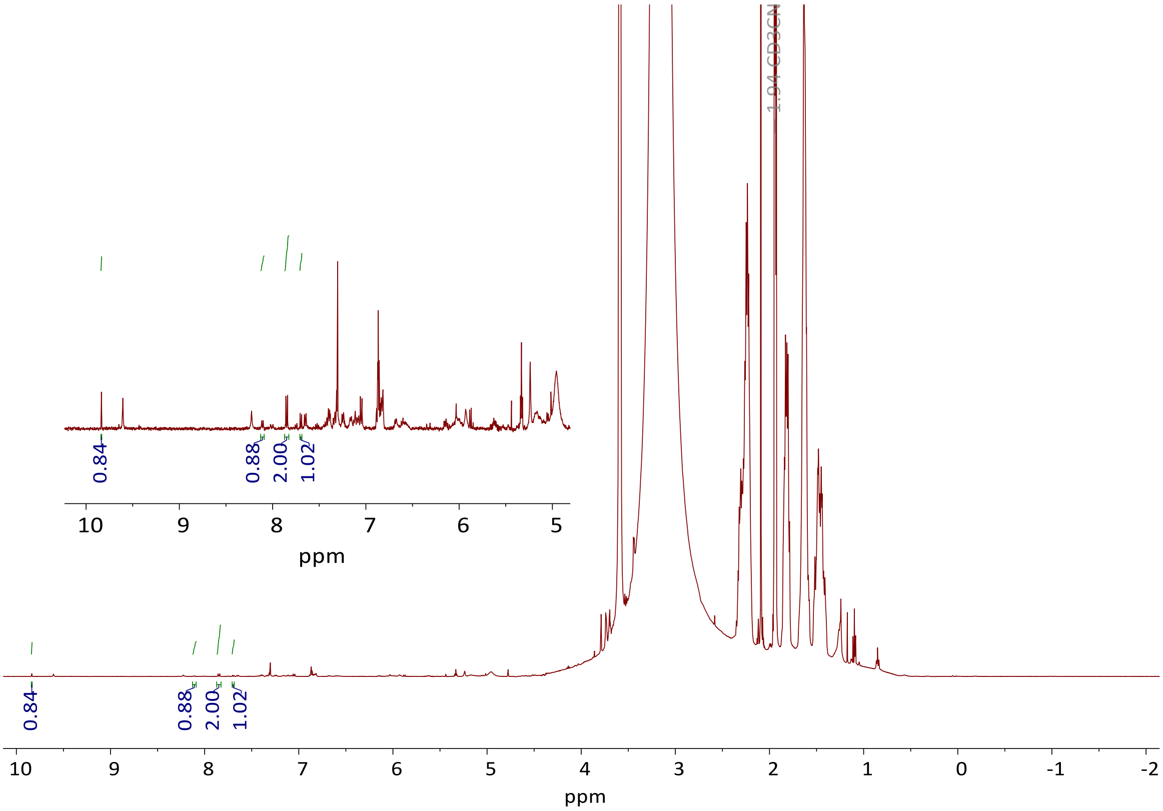

Spectrum S63. ^{1}H NMR (500 MHz, Acetonitrile-d_3/H_2O (9/1), 298 K) spectrum of post-sonication polymer $\mathbf{1}_{GEM}$ before being washed with methanol.

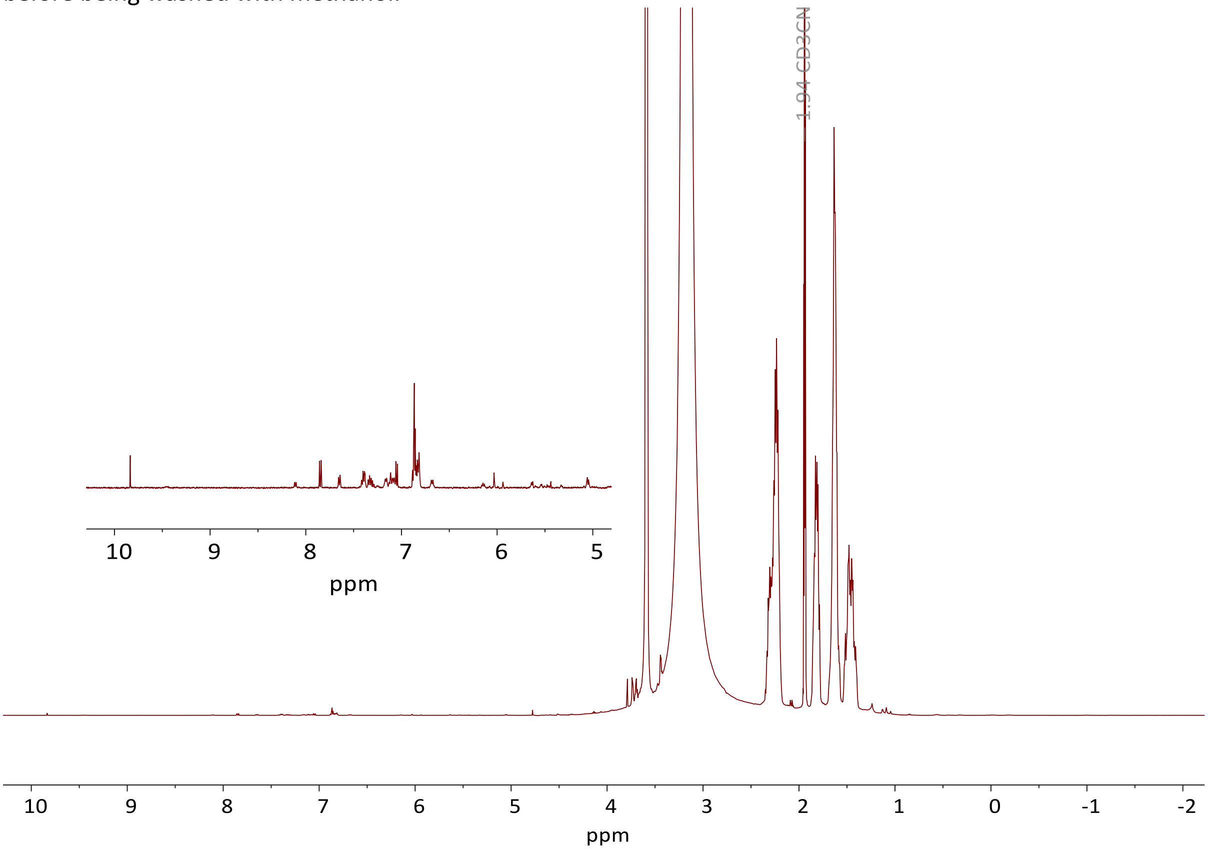

Spectrum S64. ^{1}H NMR (500 MHz, Acetonitrile-d_3/H_2O (9/1), 298 K) spectrum of post-sonication polymer $\mathbf{1}_{GEM}$ after being washed with methanol.

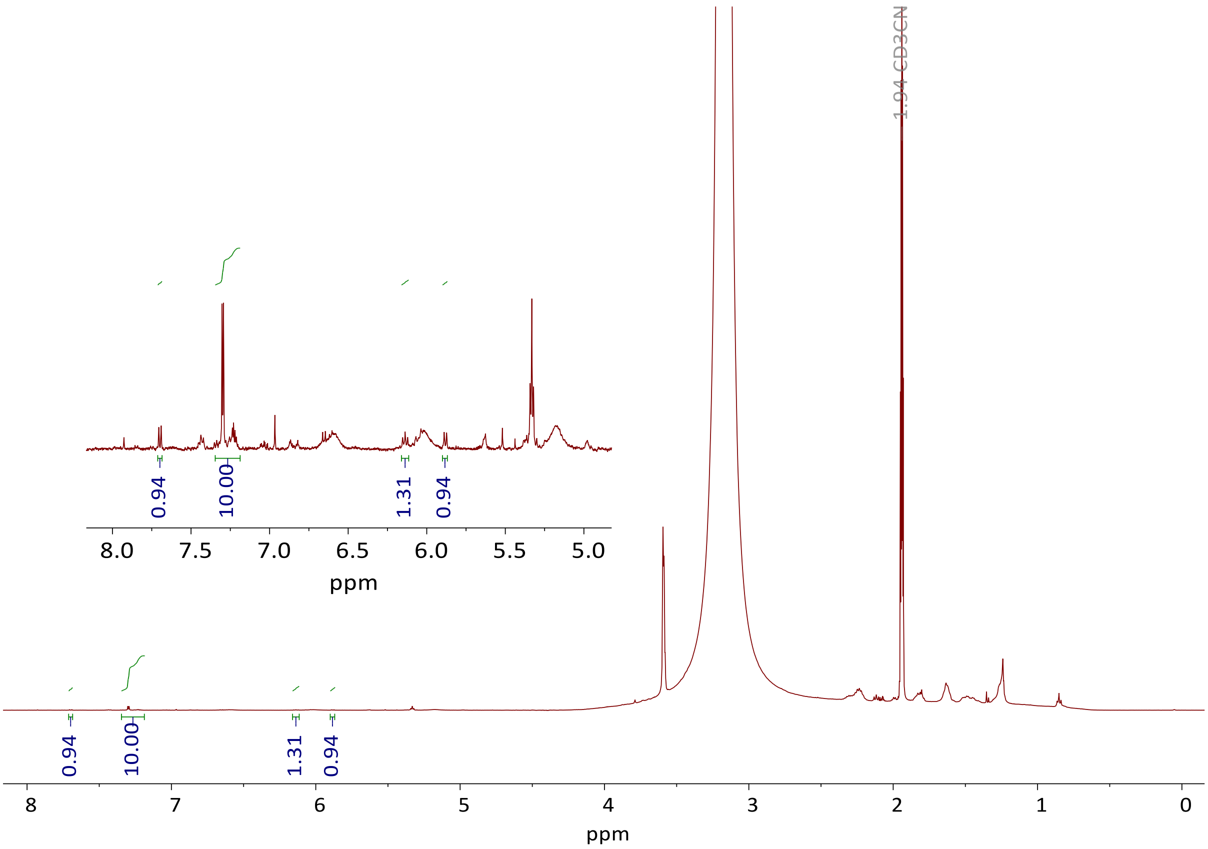

Spectrum S65. ^{1}H NMR (500 MHz, Acetonitrile-d_3/H_2O (9/1), 298 K) spectrum of the concentrated methanol washings from post-sonication polymer **1**$_{\boldsymbol{GEM}}$.

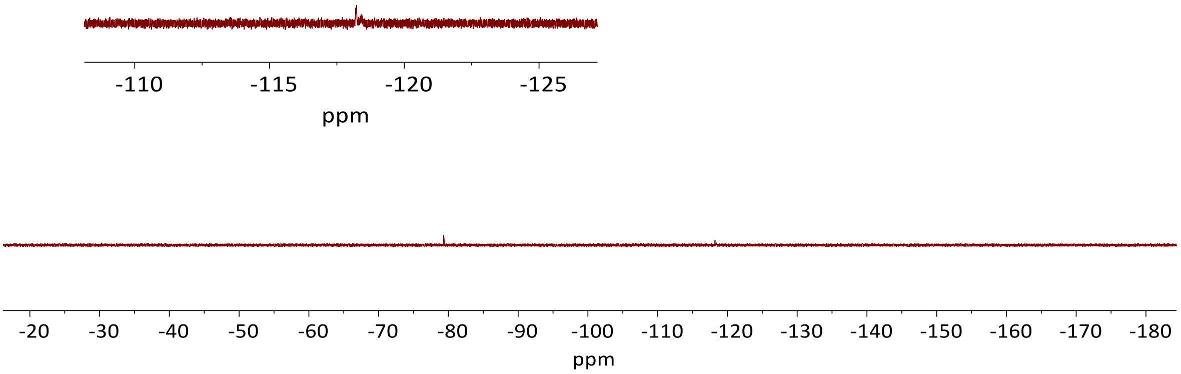

Spectrum S66. ^{19}F NMR (471 MHz, Acetonitrile-d_6/H_2O (9/1), 298 K) spectrum of the concentrated methanol washings from post-sonication polymer **1**$_{\boldsymbol{GEM}}$.

9.3.9 Post-Sonication ^1H NMR Spectra of Polymer S20

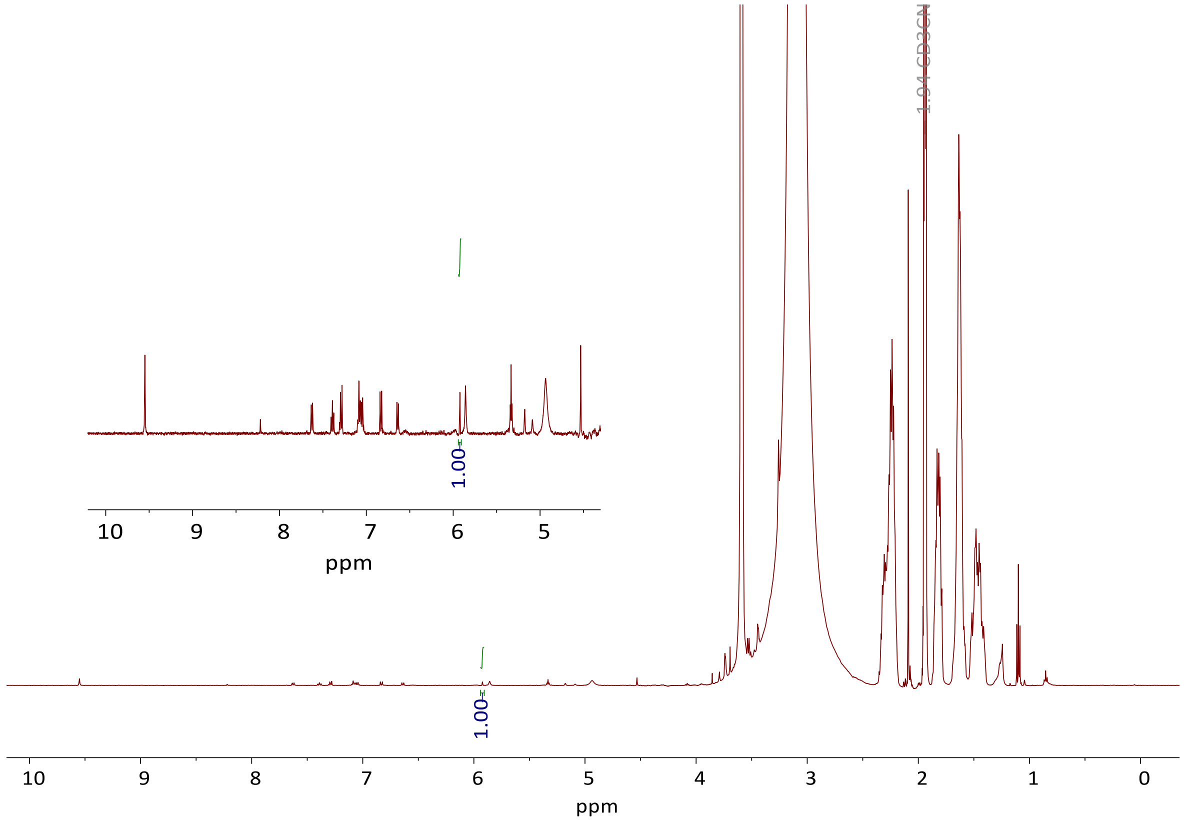

Spectrum S67. ^1H NMR (500 MHz, Acetonitrile-d_3/H_2O (9/1), 298 K) spectrum of post-sonication polymer **S20** before being washed with methanol.

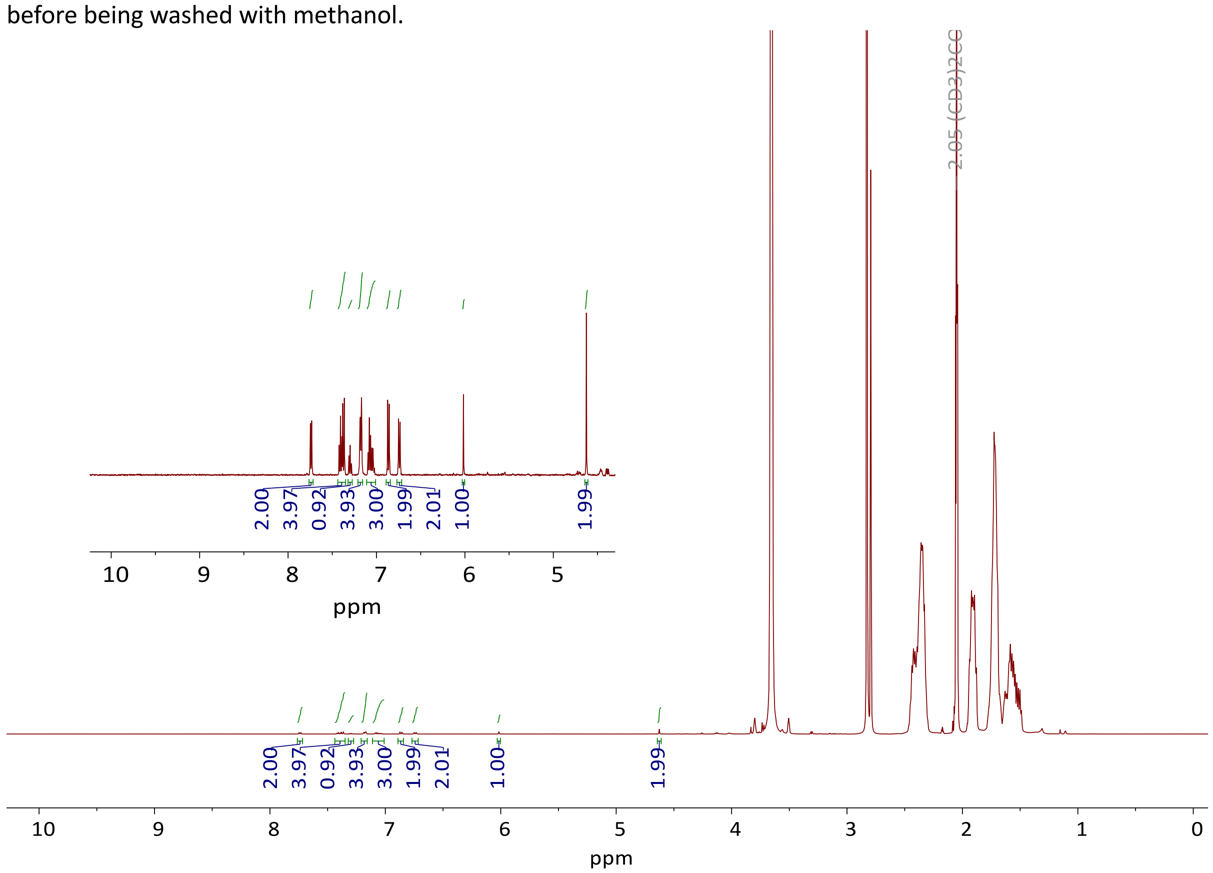

Spectrum S68. ^1H NMR (500 MHz, Acetone-d_6, 298 K) spectrum of post-sonication polymer **S20** after being washed with methanol.

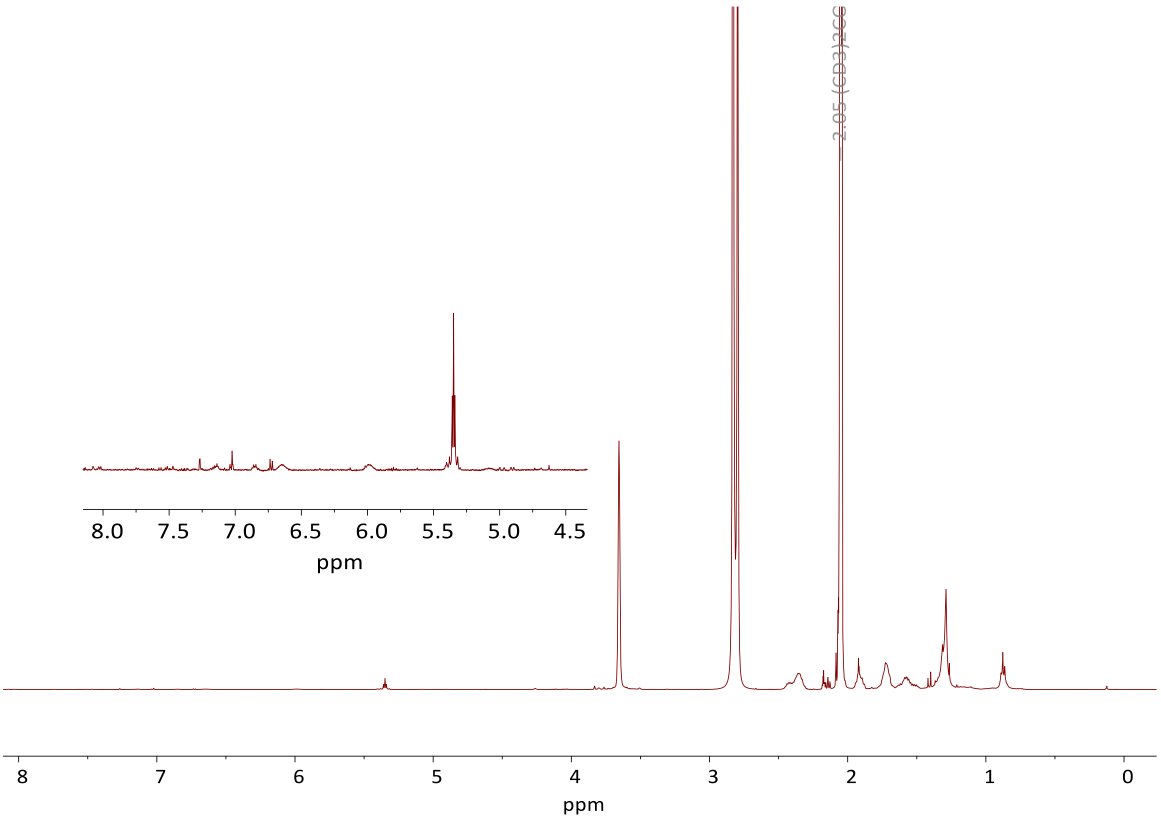

Spectrum S69. ^{1}H NMR (500 MHz, Acetone-d_6, 298 K) spectrum of the concentrated methanol washings from post-sonication polymer **S20**.

9.3.10 Post-Sonication ^{1}H NMR Spectra of Polymer S21

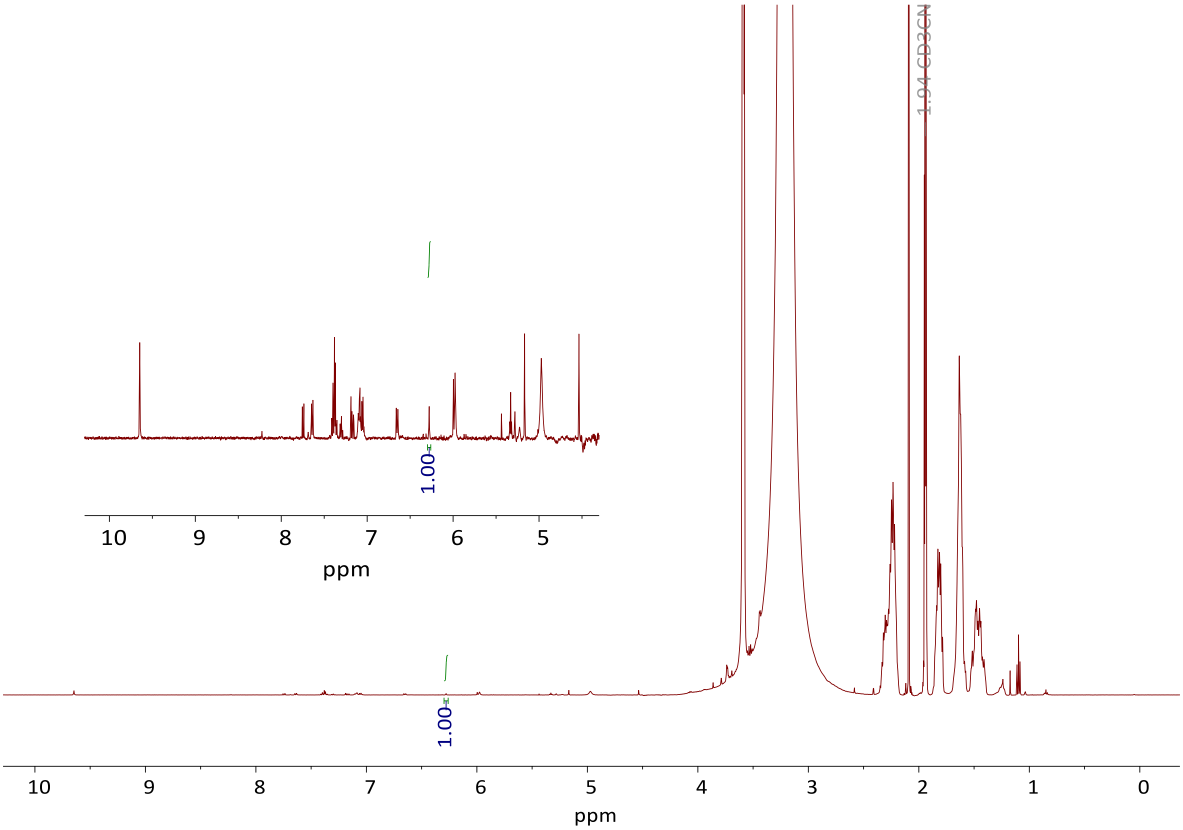

Spectrum S70. ^{1}H NMR (500 MHz, Acetonitrile-d_3/H_2O (9/1), 298 K) spectrum of post-sonication polymer **S21** before being washed with methanol.

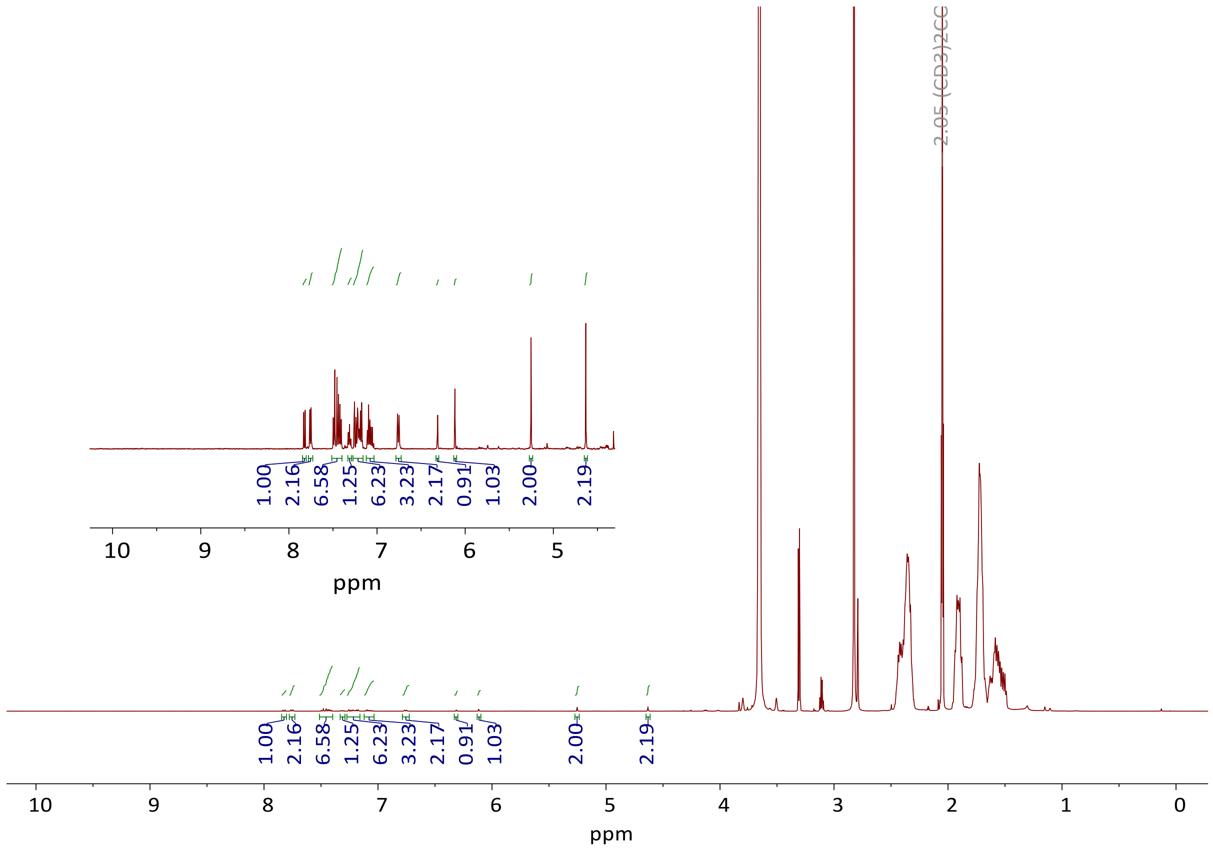

Spectrum S71. ^{1}H NMR (500 MHz, Acetone-d_6, 298 K) spectrum of post-sonication polymer **S21** after being washed with methanol.

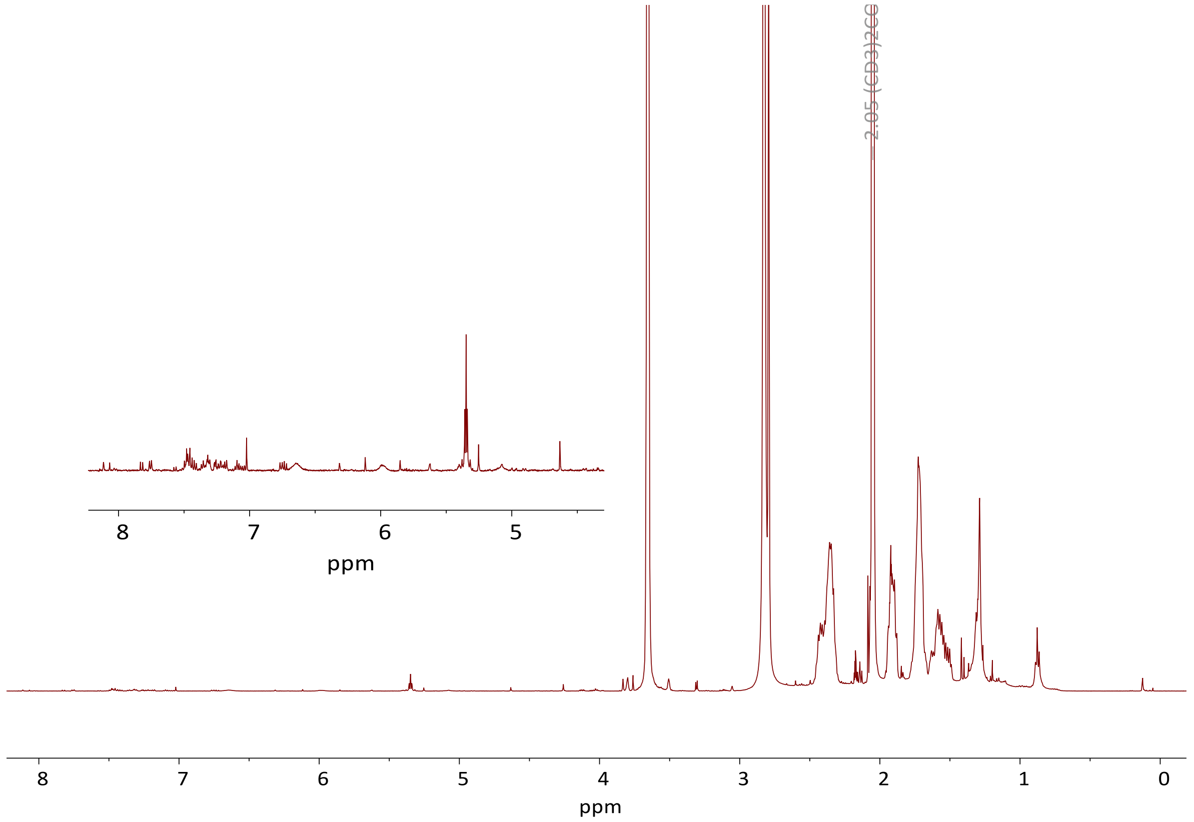

Spectrum S72. ^{1}H NMR (500 MHz, Acetone-d_6, 298 K) spectrum of the concentrated methanol washings from post-sonication polymer **S21**.

9.3.11 Post-Sonication ^{1}H NMR Spectra of Polymer $\mathbf{9_{-112}}$

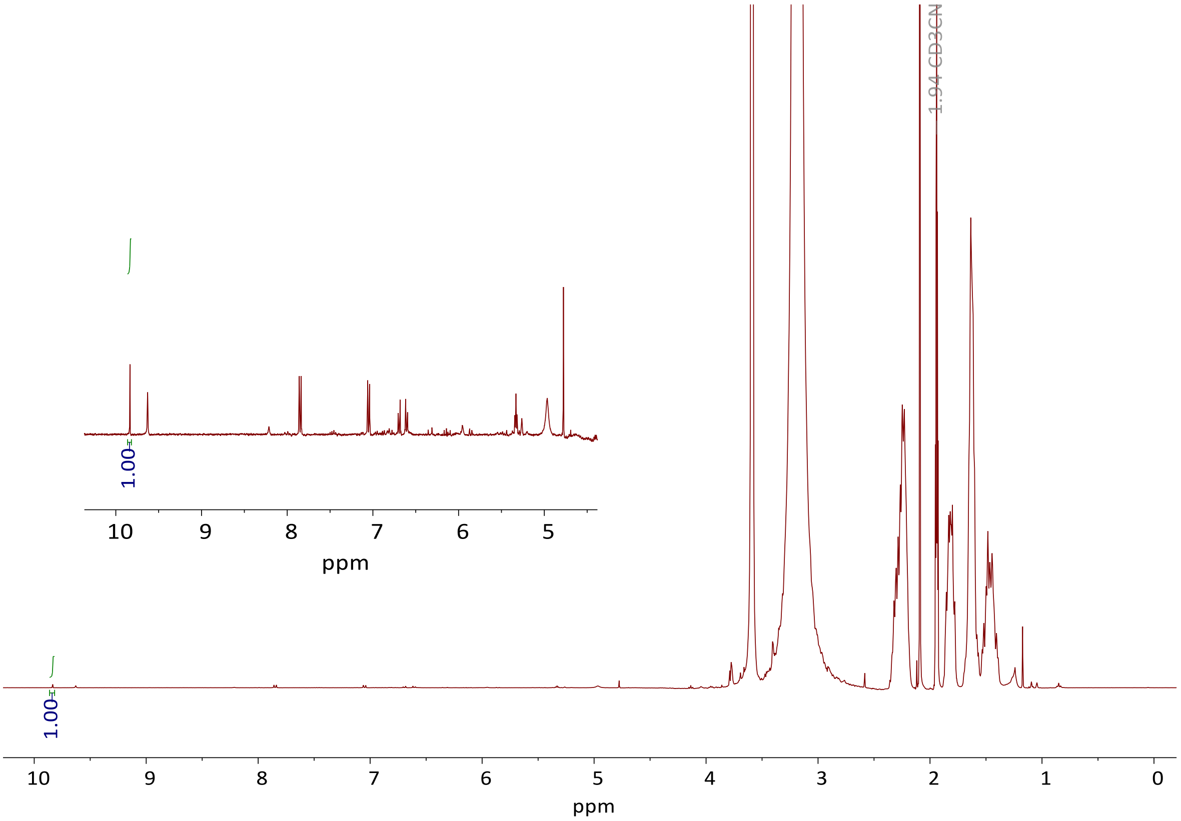

Spectrum S73. ^{1}H NMR (400 MHz, Acetonitrile-d_3/H_2O (9/1), 298 K) spectrum of post-sonication polymer $\mathbf{9_{-112}}$ before being washed with methanol.

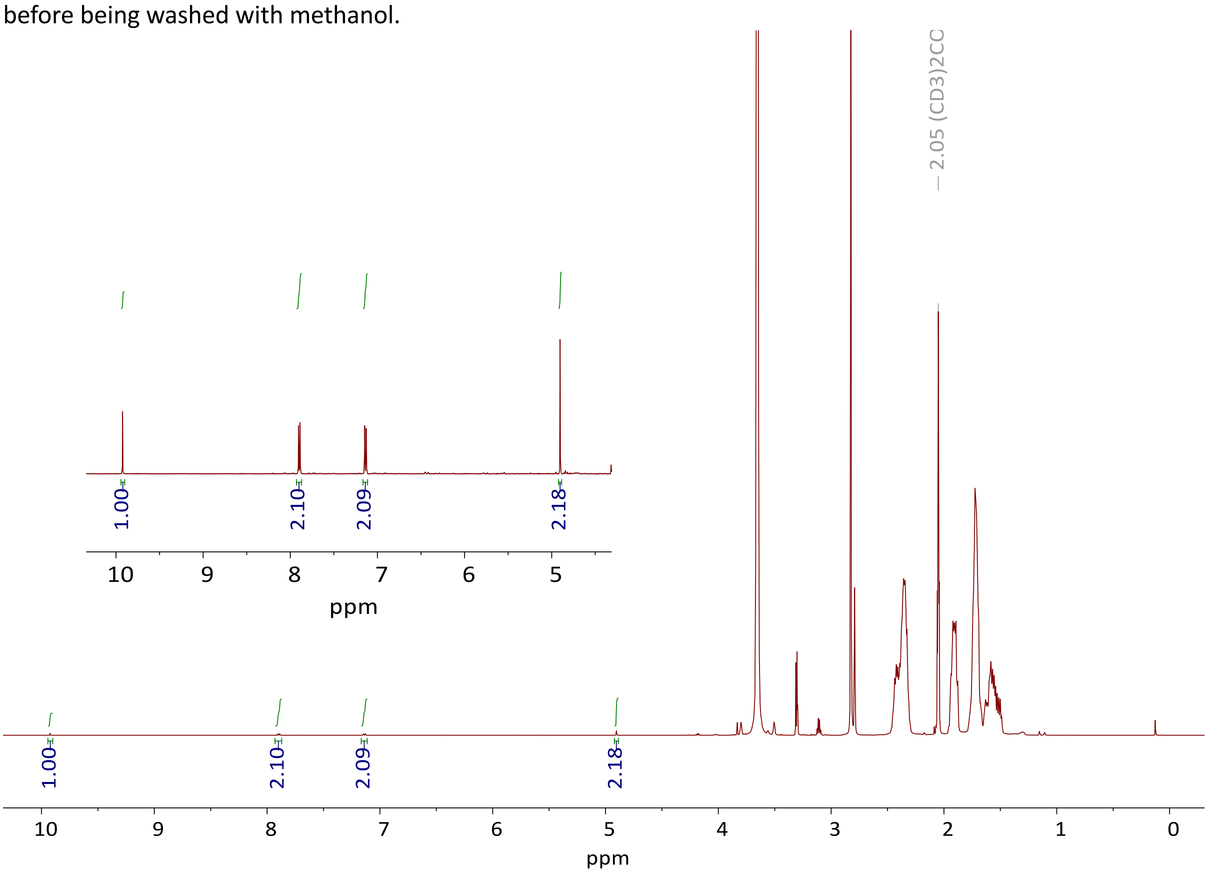

Spectrum S74. ^{1}H NMR (500 MHz, Acetone-d_6, 298 K) spectrum of post-sonication polymer $\mathbf{9_{-112}}$ after being washed with methanol.

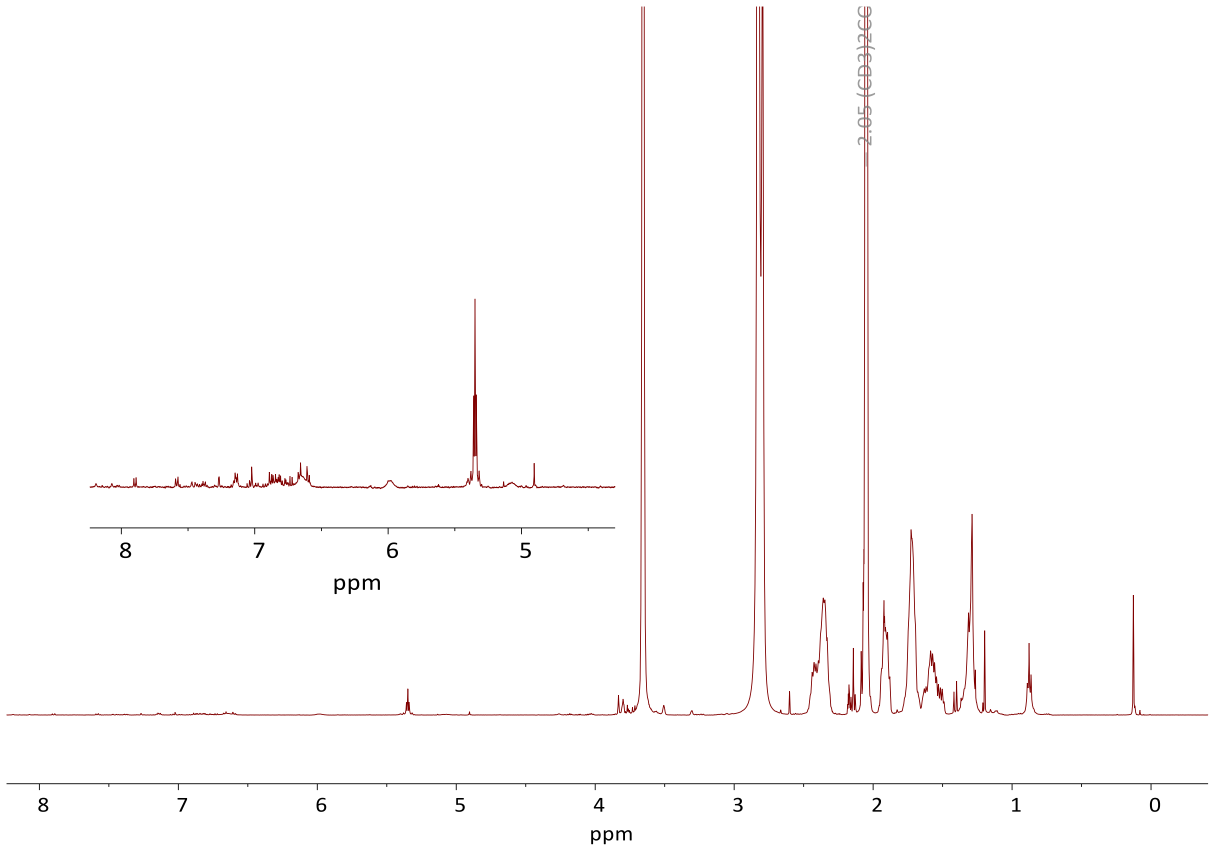

Spectrum S75. ^{1}H NMR (500 MHz, Acetone-d_6, 298 K) spectrum of the concentrated methanol washings from post-sonication polymer $\mathbf{9}_{\mathbf{-112}}$.

9.4 Post-Compression NMR Spectra

9.4.1 Post-Compression ^{1}H NMR Spectra of Polymer $\mathbf{5}_{\text{-138}}$ (Run 1)

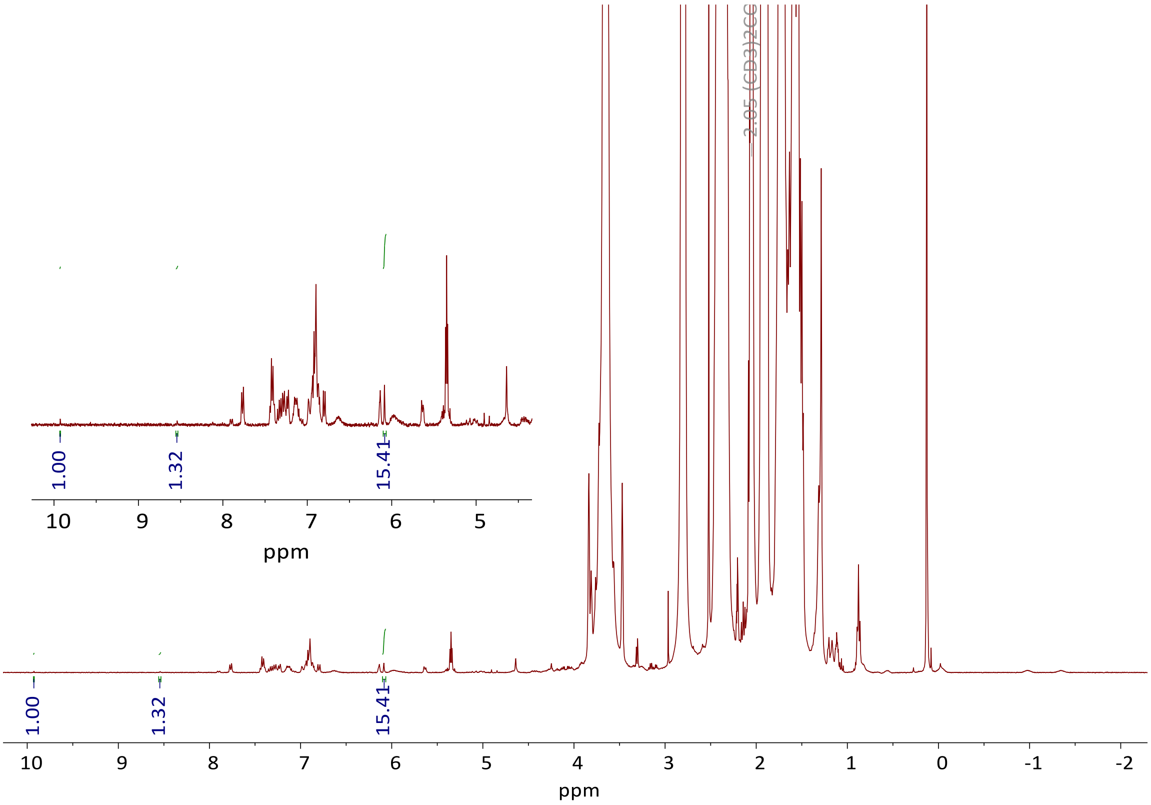

Spectrum S76. ^{1}H NMR (400 MHz, Acetone-d_6, 298 K) spectrum of post-compression polymer $\mathbf{5}_{\text{-138}}$ before being washed with methanol.

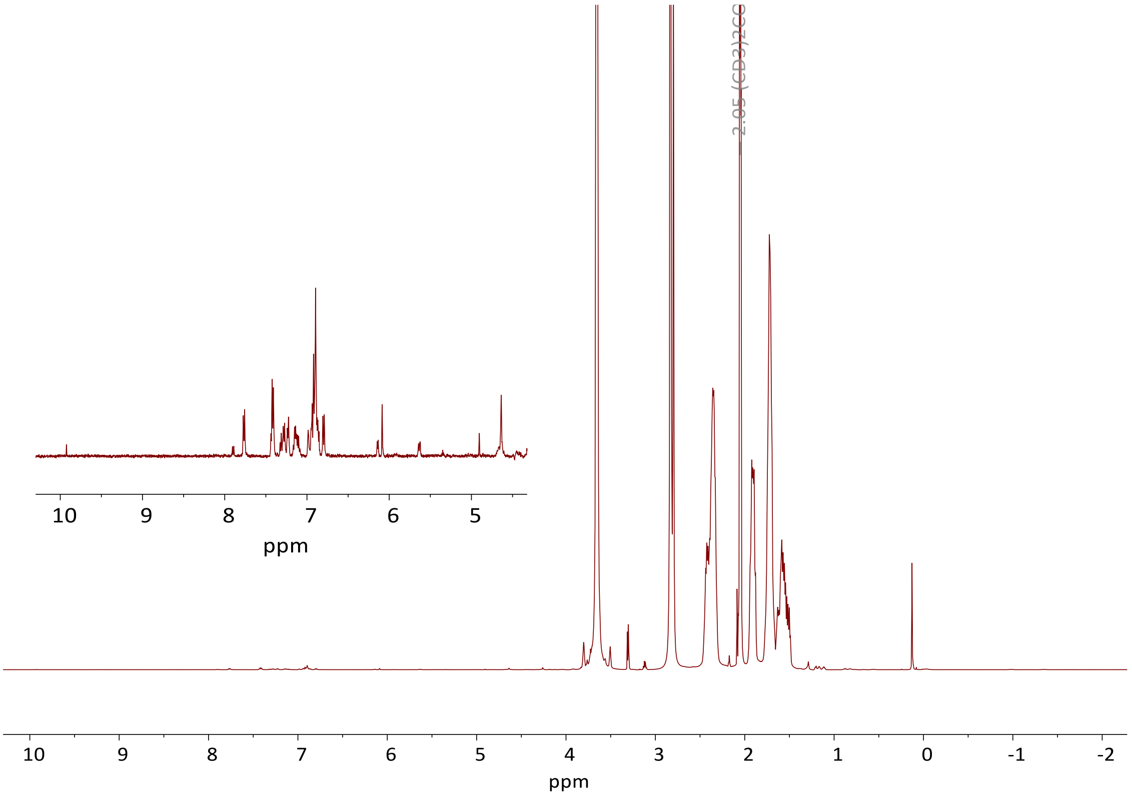

Spectrum S77. ^{1}H NMR (500 MHz, Acetone-d_6, 298 K) spectrum of post-compression polymer $\mathbf{5}_{\text{-138}}$ after being washed with methanol.

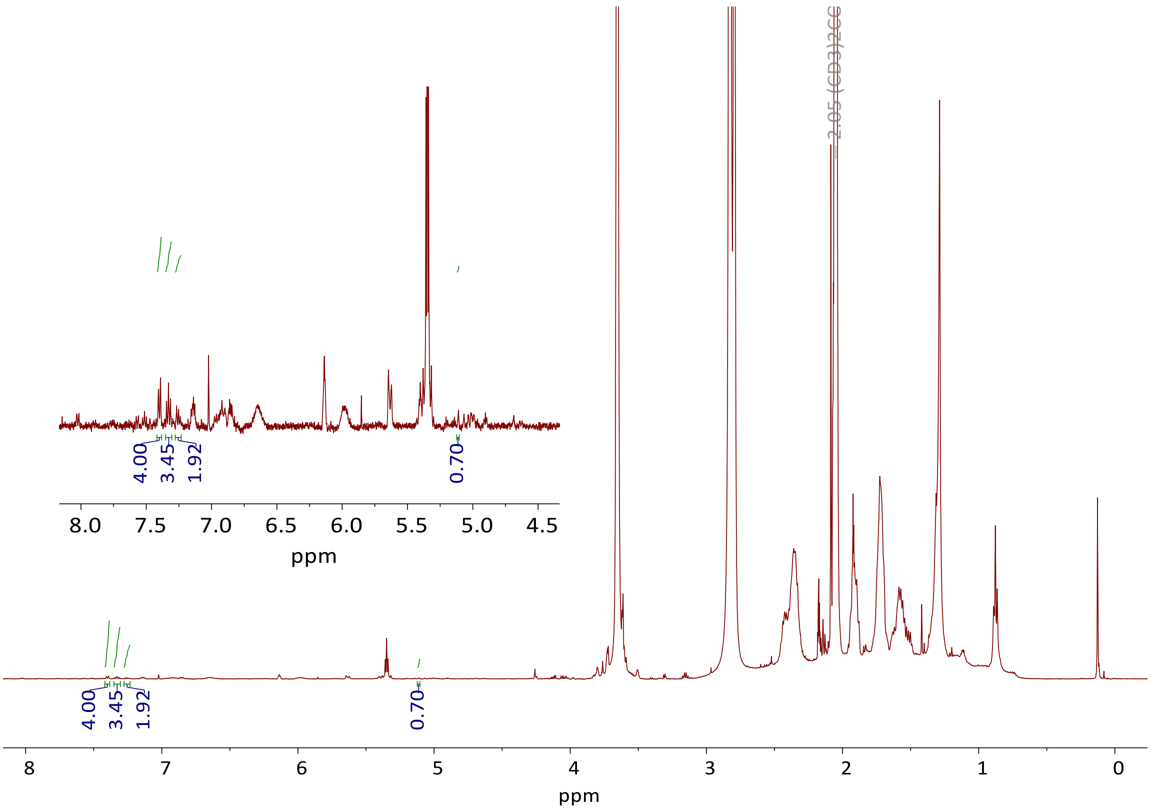

Spectrum S78. ^{1}H NMR (500 MHz, Acetone-d_6, 298 K) spectrum of the concentrated methanol washings from post-compression polymer **5**$_{\mathbf{-138}}$.

9.4.2 Post-Compression ^{1}H NMR Spectra of Polymer 5$_{-138}$ (Run 2)

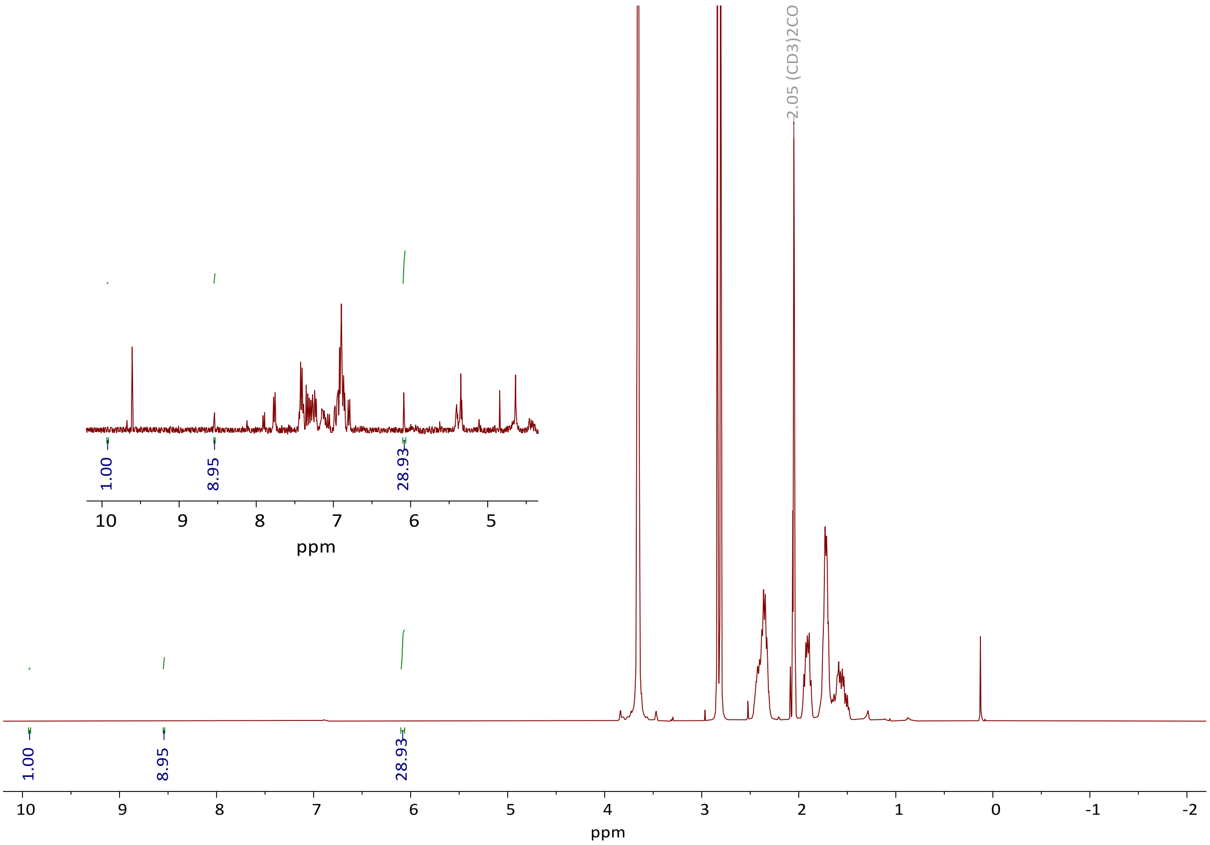

Spectrum S79. ^{1}H NMR (400 MHz, Acetone-d_6, 298 K) spectrum of post-compression polymer **5**$_{\mathbf{-138}}$ before being washed with methanol.

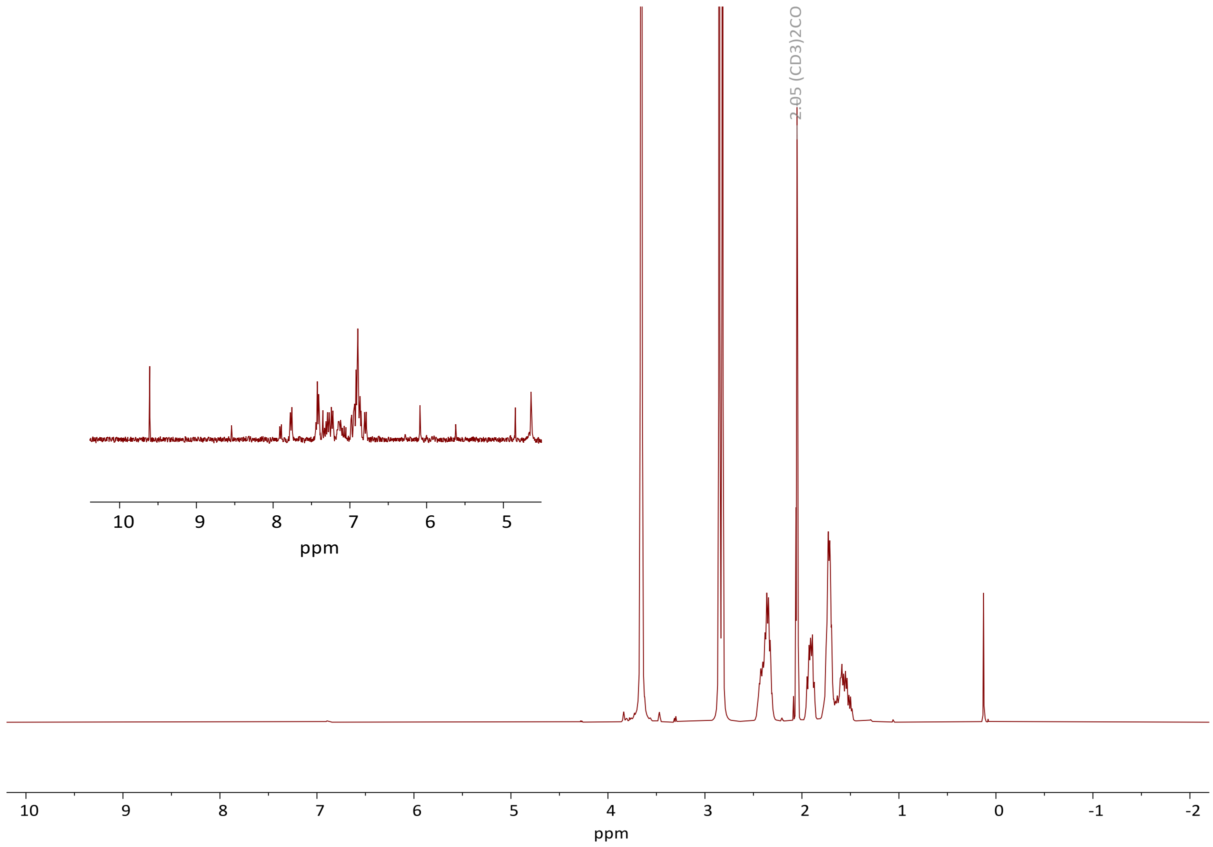

Spectrum S80. ^{1}H NMR (500 MHz, Acetone-d_6, 298 K) spectrum of post-compression polymer **5**$_{\mathbf{-138}}$ after being washed with methanol.

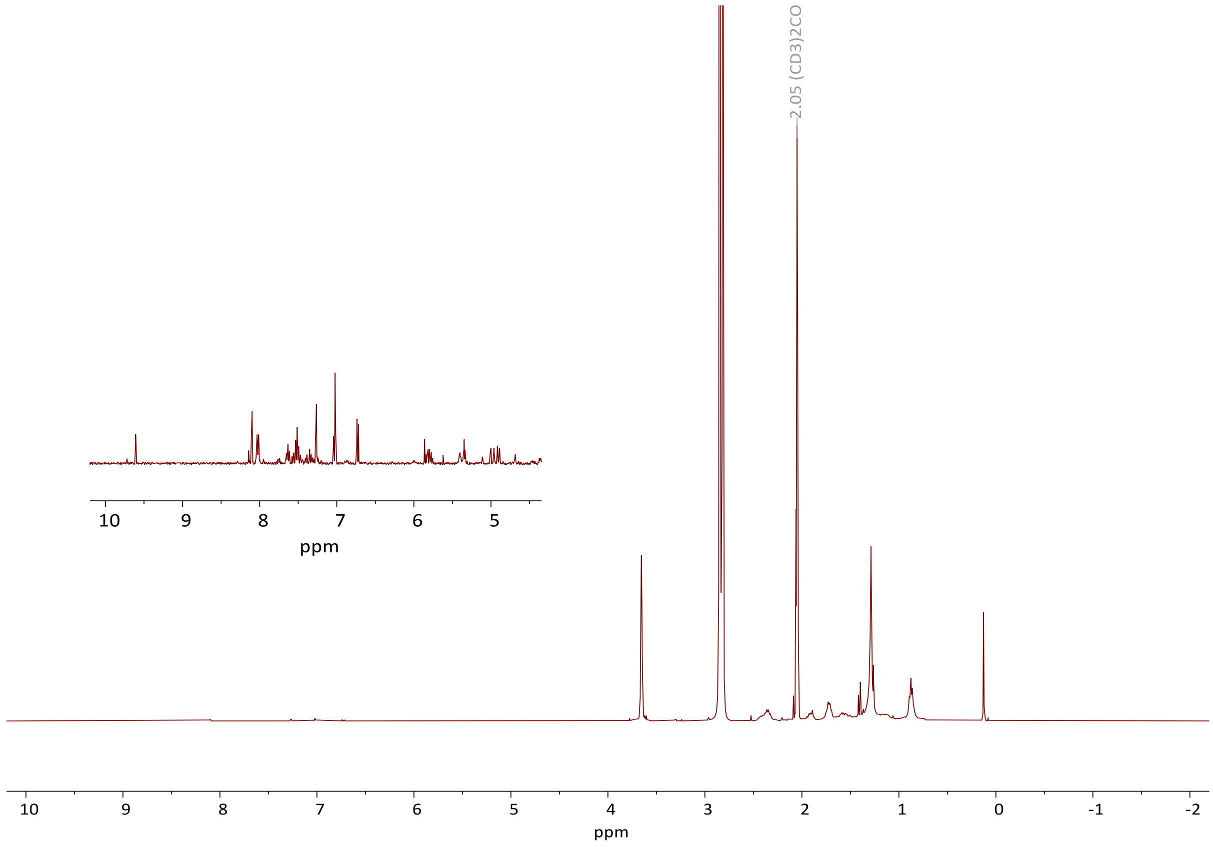

Spectrum S81. ^{1}H NMR (500 MHz, Acetone-d_6, 298 K) spectrum of the concentrated methanol washings from post-compression polymer **5**$_{\mathbf{-138}}$.

9.4.3 Post-Compression ^{1}H NMR Spectra of Polymer S20

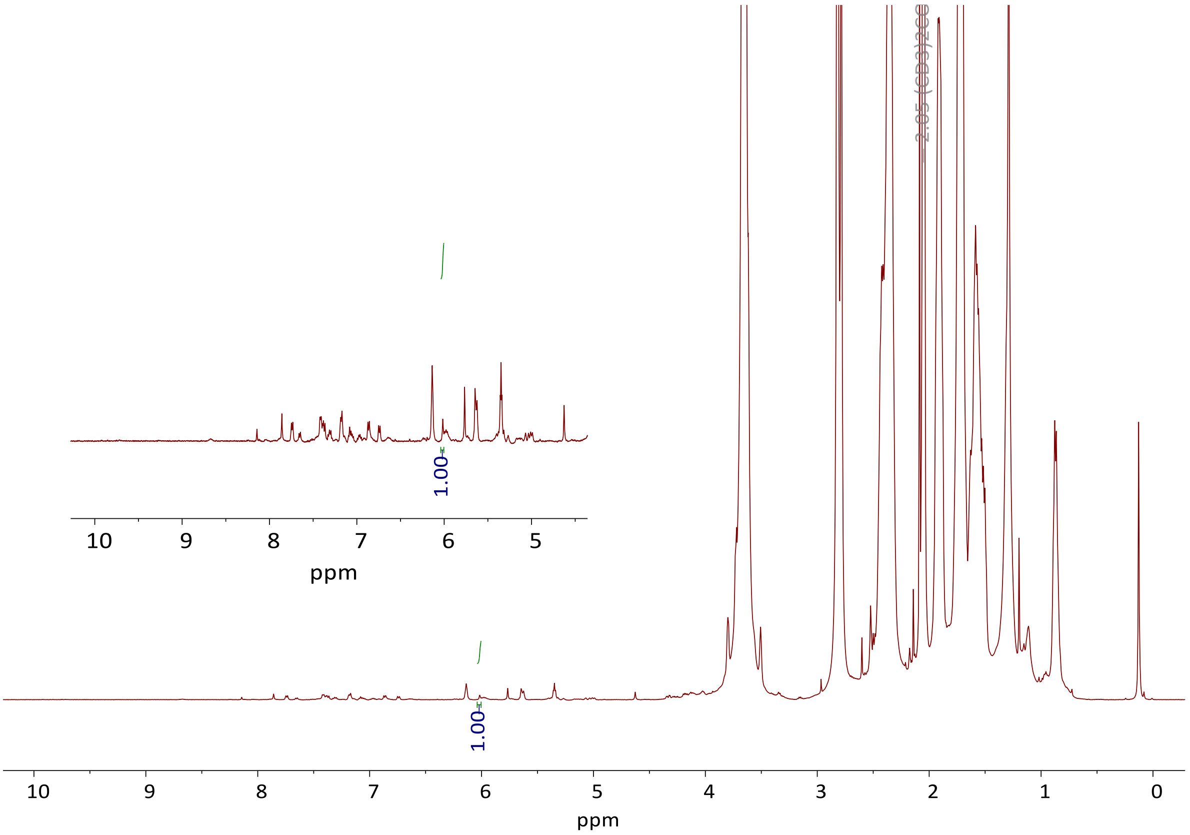

Spectrum S82. ^{1}H NMR (500 MHz, Acetone-d_6, 298 K) spectrum of post-compression polymer **S20** before being washed with methanol.

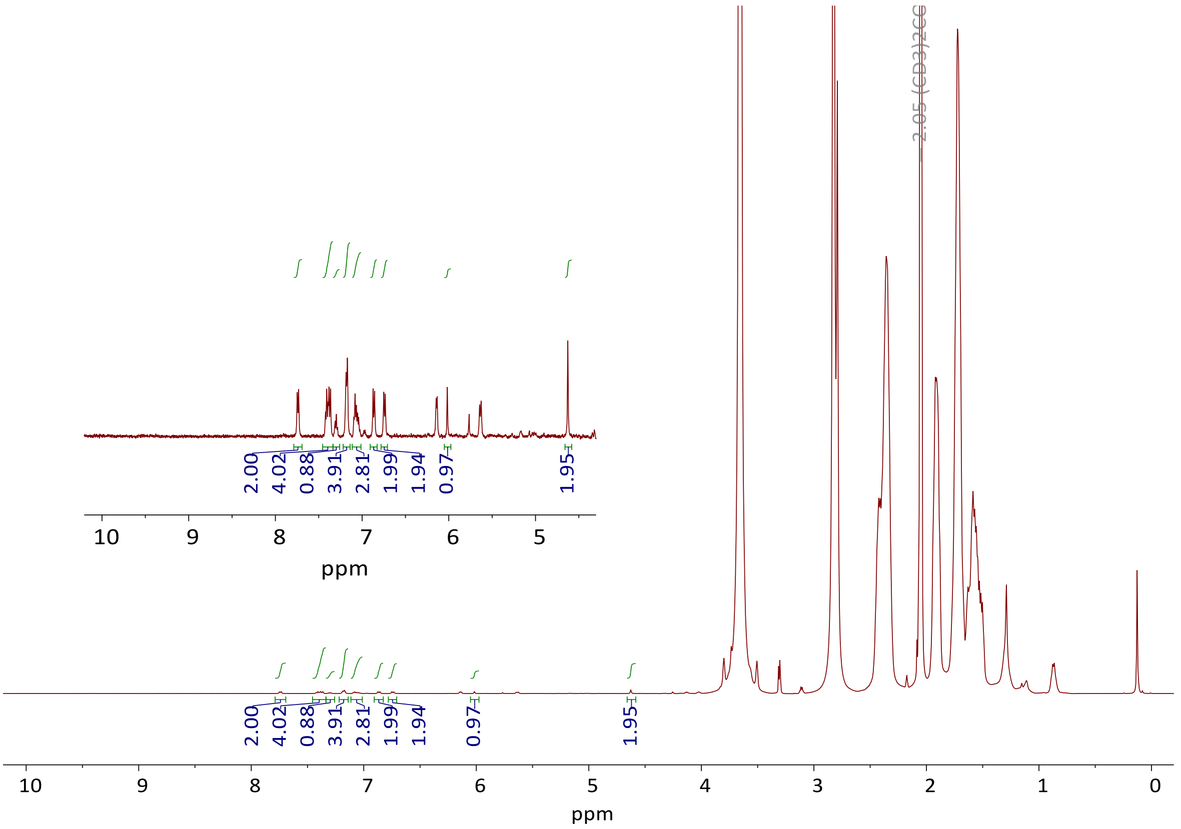

Spectrum S83. ^{1}H NMR (500 MHz, Acetone-d_6, 298 K) spectrum of post-compression polymer **S20** after being washed with methanol.

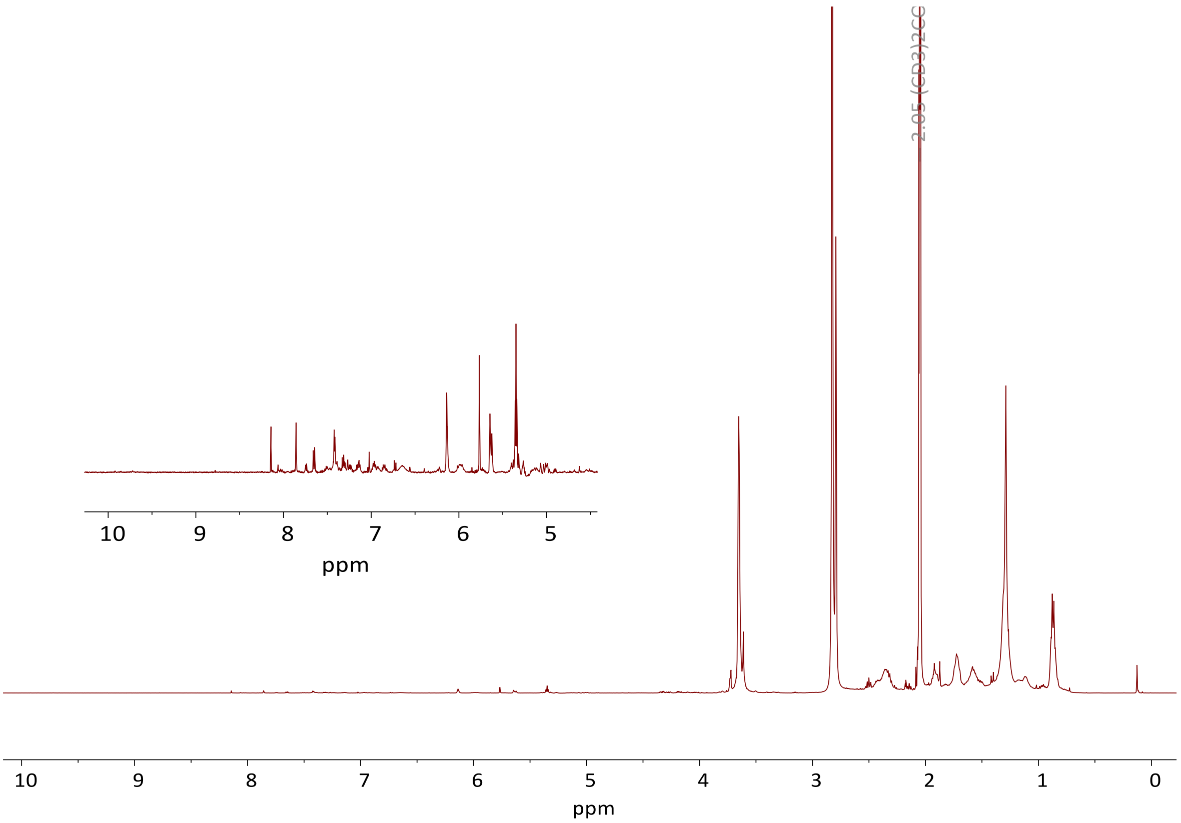

Spectrum S84. ^{1}H NMR (500 MHz, Acetone-d_6, 298 K) spectrum of the concentrated methanol washings from post-compression polymer **S20**.

9.4.4 Post-Compression ^{1}H NMR Spectra of Polymer 9$_{-112}$

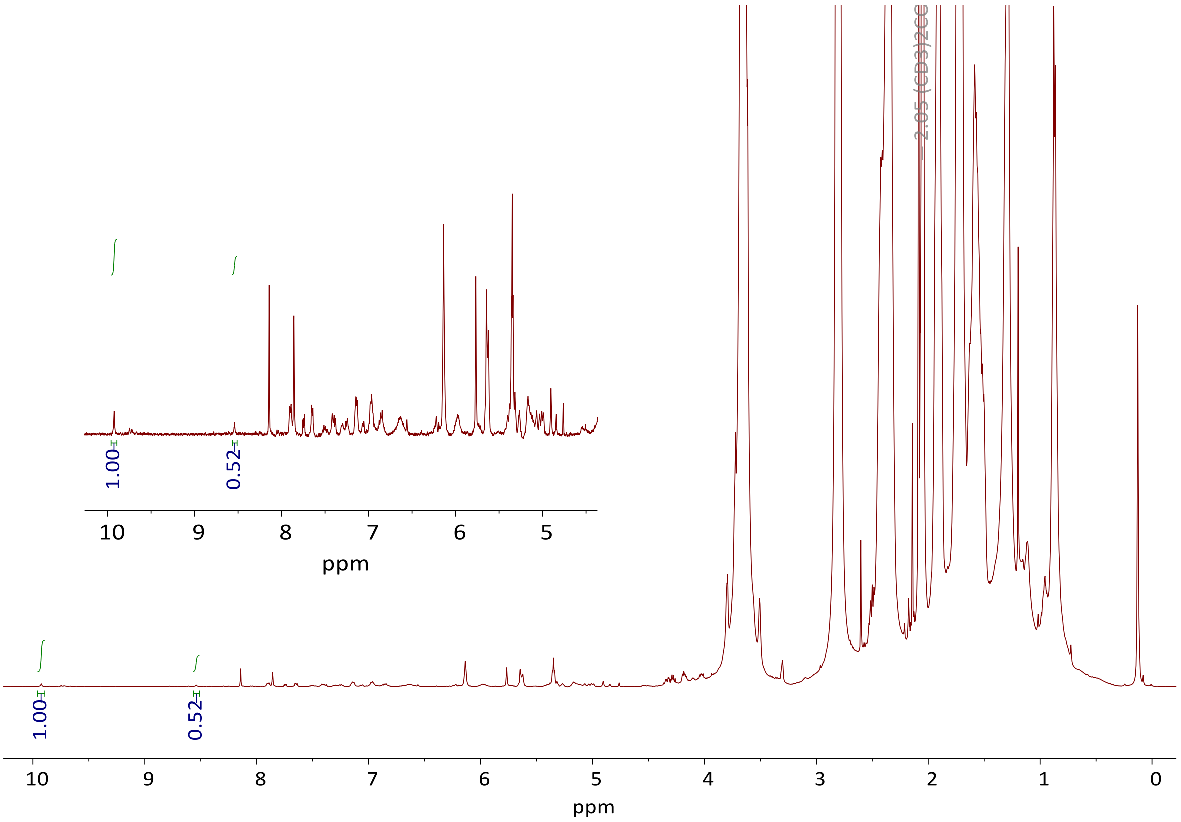

Spectrum S85. ^{1}H NMR (500 MHz, Acetone-d_6, 298 K) spectrum of post-compression polymer **9$_{-112}$** before being washed with methanol.

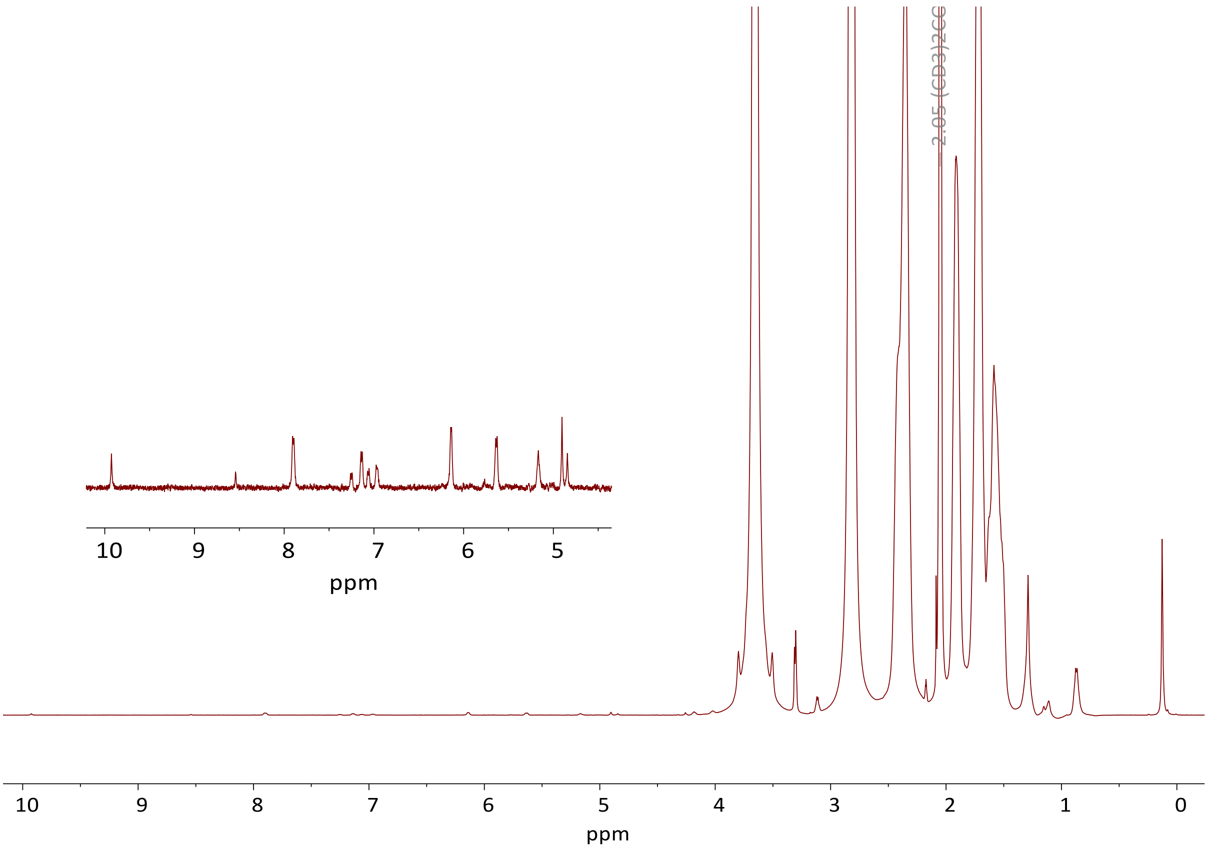

Spectrum S86. ^{1}H NMR (500 MHz, Acetone-d_6, 298 K) spectrum of post-compression polymer **9**$_{\mathbf{-112}}$ after being washed with methanol.

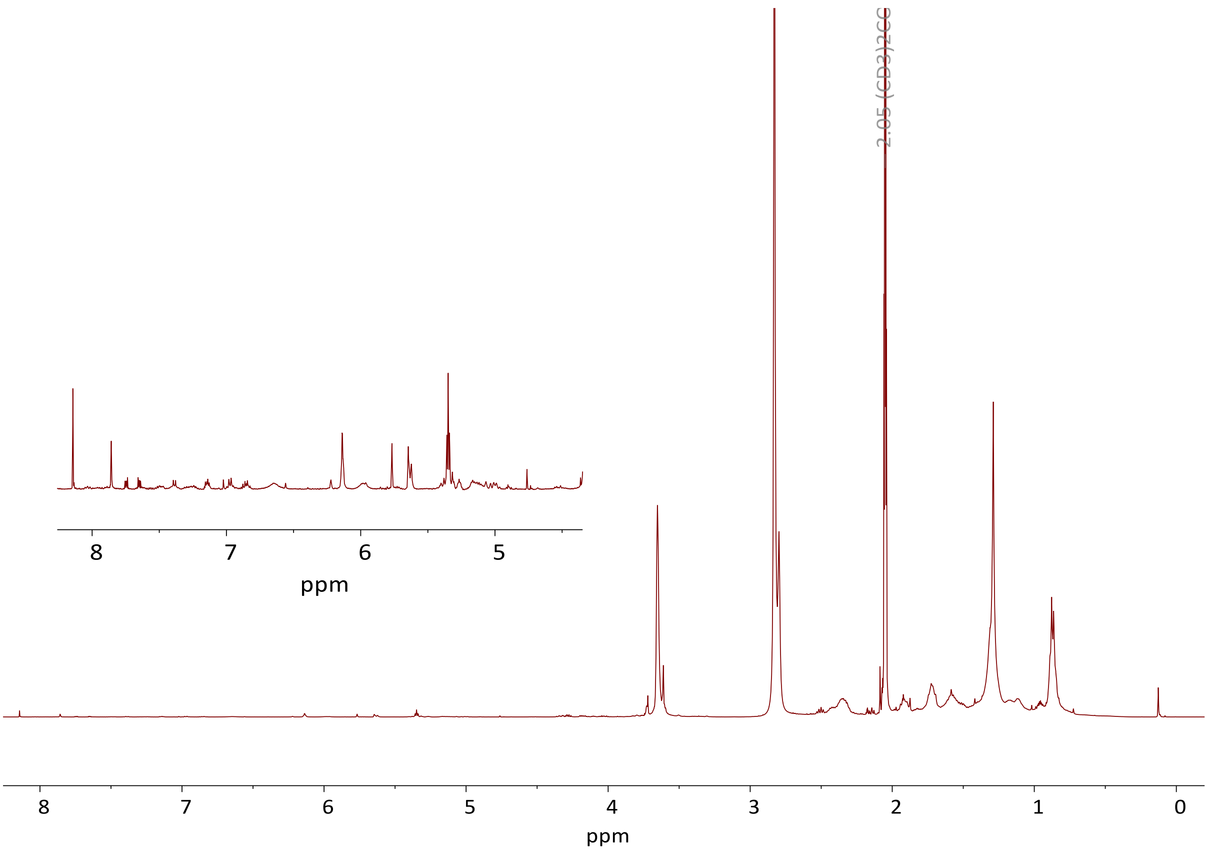

Spectrum S87. ^{1}H NMR (500 MHz, Acetone-d_6, 298 K) spectrum of the concentrated methanol washings from post-compression polymer **9**$_{\mathbf{-112}}$.

9.5 Post-BMG NMR Spectra

9.5.1 Post-BMG (Dry) ^1H NMR Spectra of Polymer 5_{-155} (Run 1)

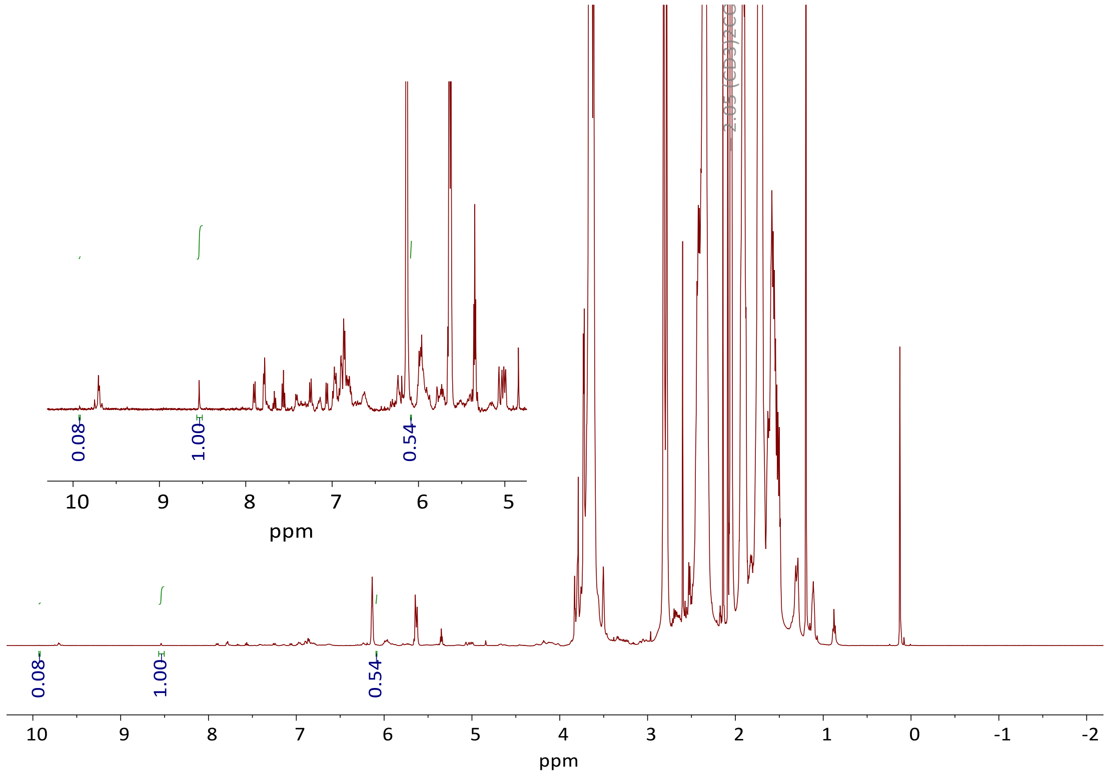

Spectrum S88. ^1H NMR (500 MHz, Acetone-d_6, 298 K) spectrum of post-BMG polymer **5**$_{-155}$ before being washed with methanol.

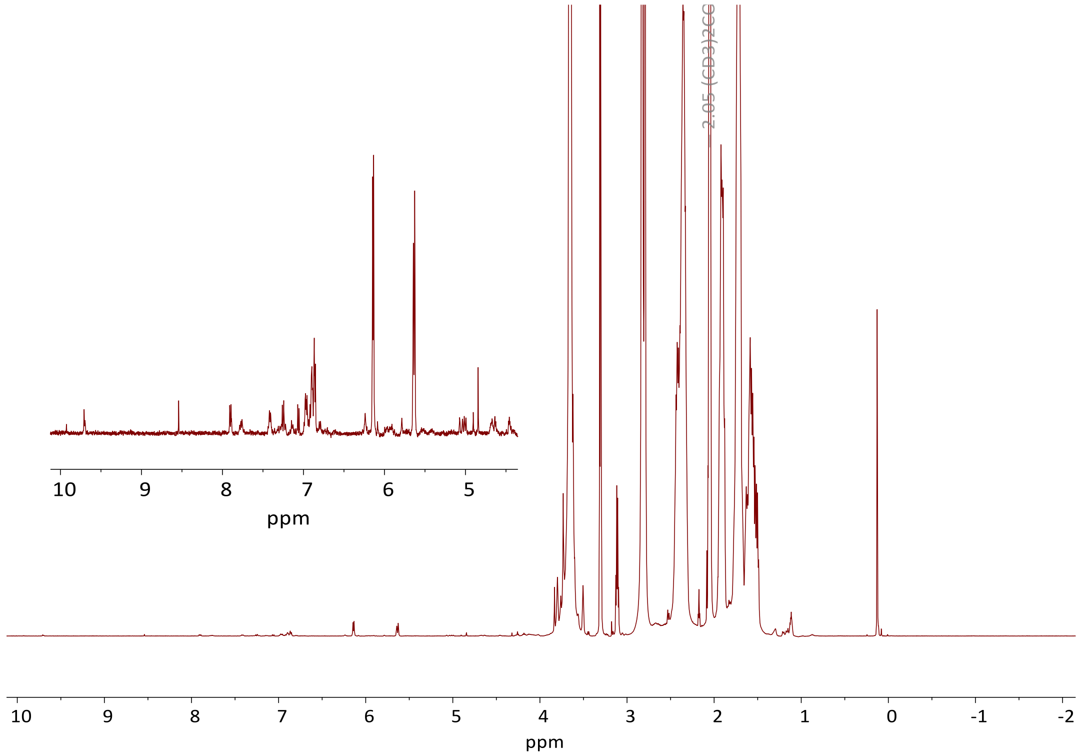

Spectrum S89. ^1H NMR (500 MHz, Acetone-d_6, 298 K) spectrum of post-BMG polymer **5**$_{-155}$ after being washed with methanol.

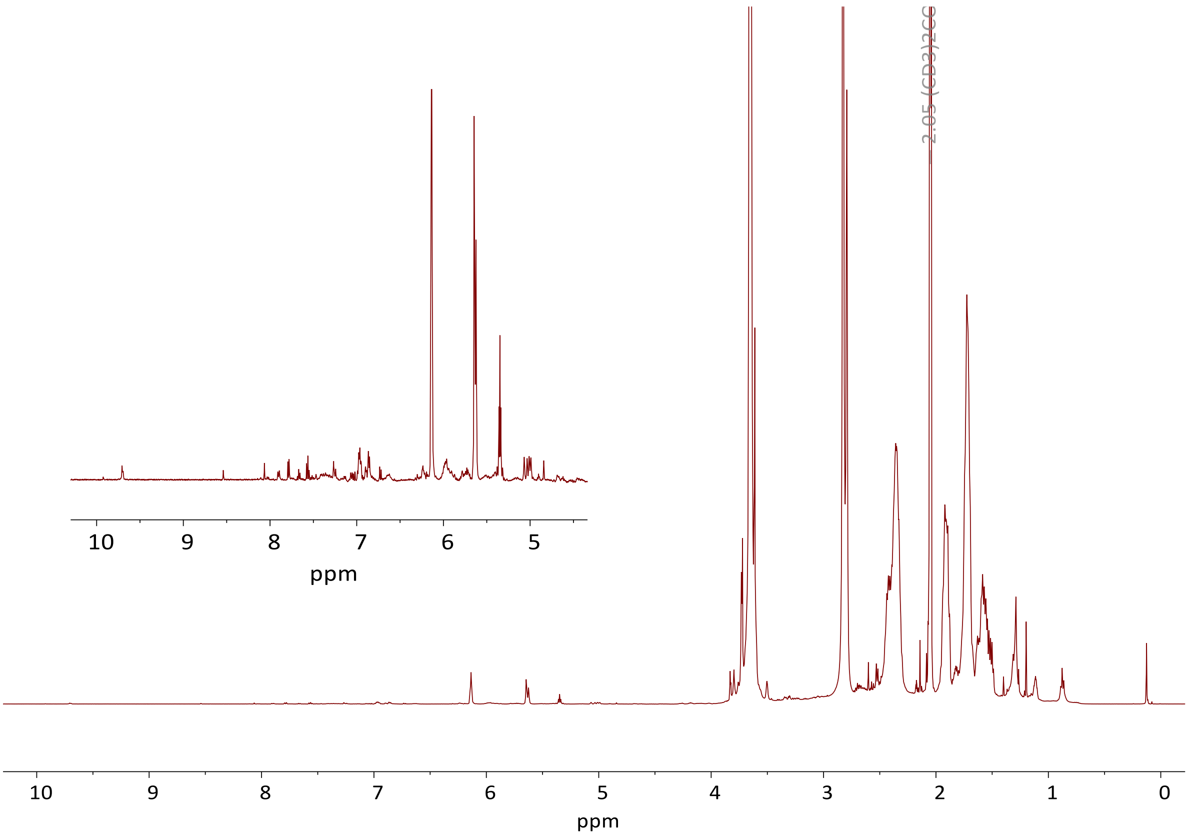

Spectrum S90. ^{1}H NMR (500 MHz, Acetone-d_6, 298 K) spectrum of the concentrated methanol washings from post-BMG polymer **5$_{-155}$**.

9.5.2 Post-BMG (Dry) ^{1}H NMR Spectra of Polymer 5$_{-155}$ (Run 2)

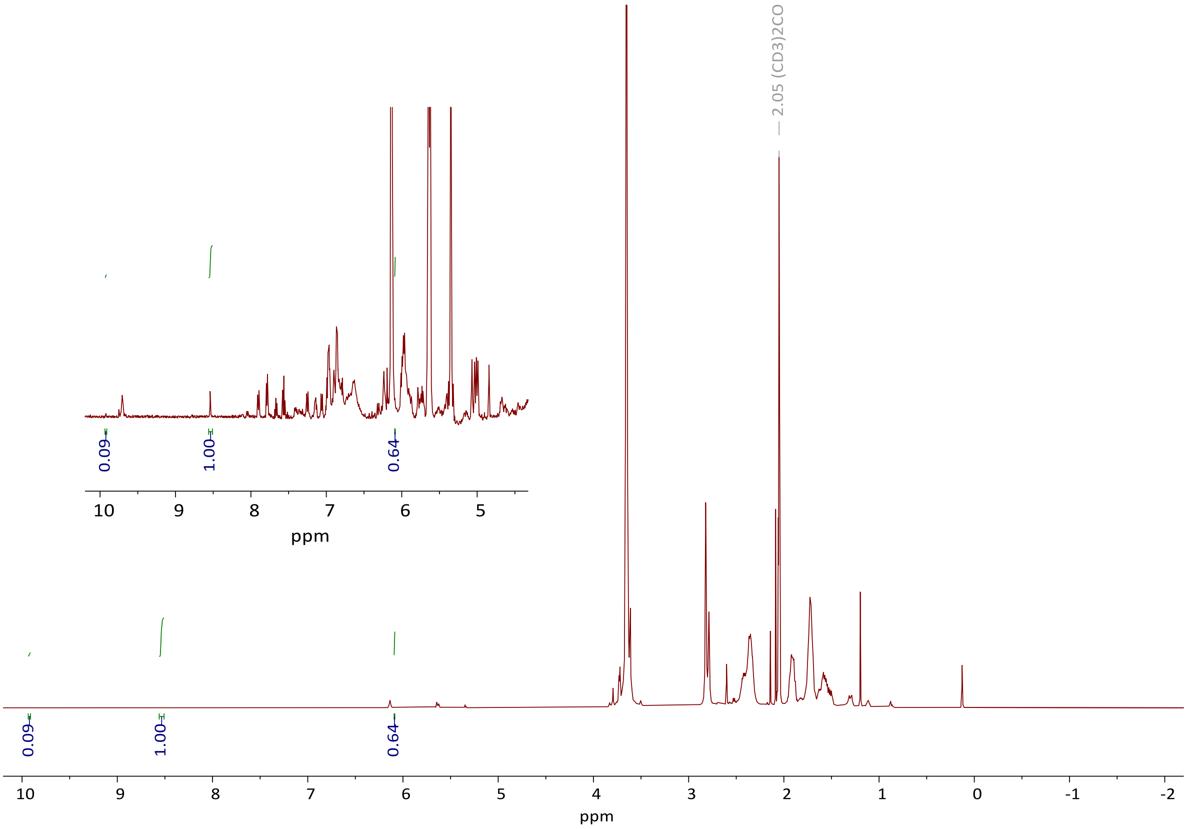

Spectrum S91. ^{1}H NMR (500 MHz, Acetone-d_6, 298 K) spectrum of post-BMG polymer **5$_{-155}$** before being washed

with methanol.

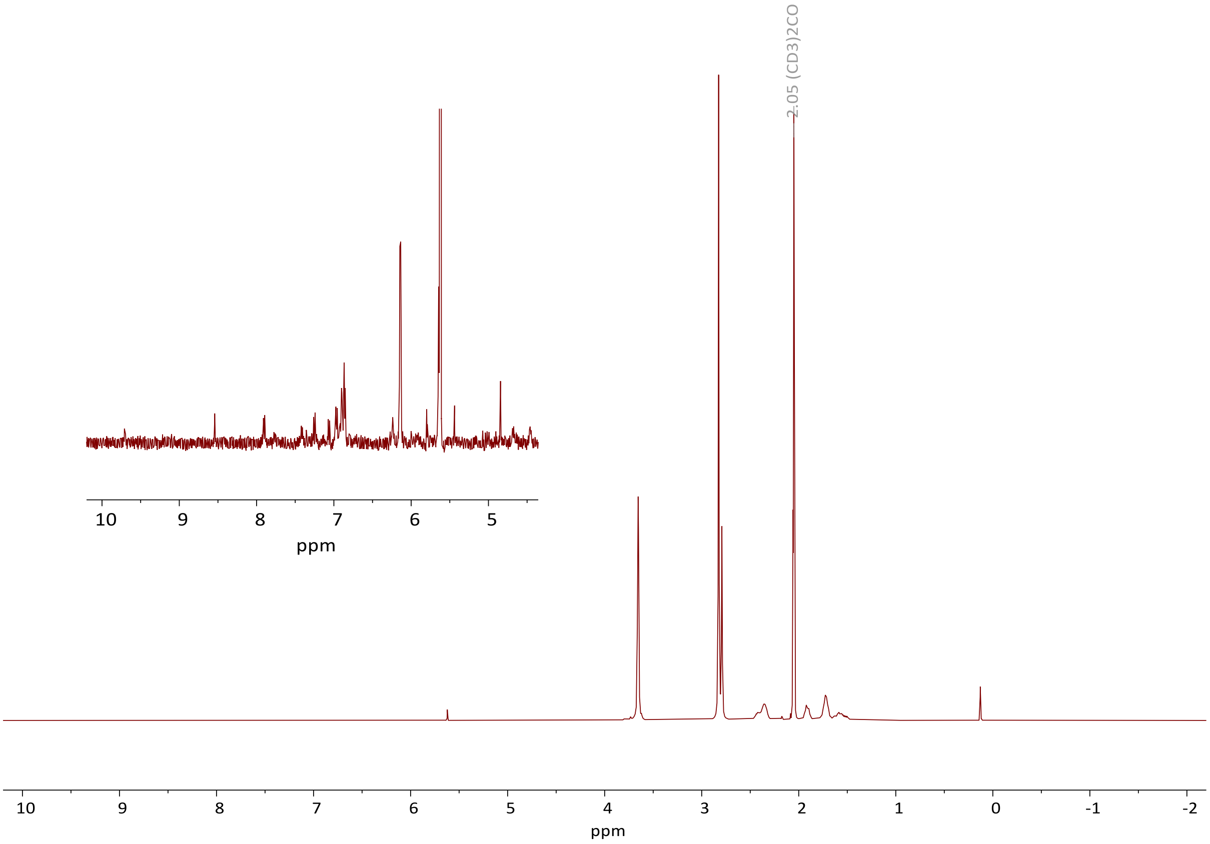

Spectrum S92. ^{1}H NMR (500 MHz, Acetone-d_6, 298 K) spectrum of post-BMG polymer **5**$_{\text{-155}}$ after being washed with methanol.

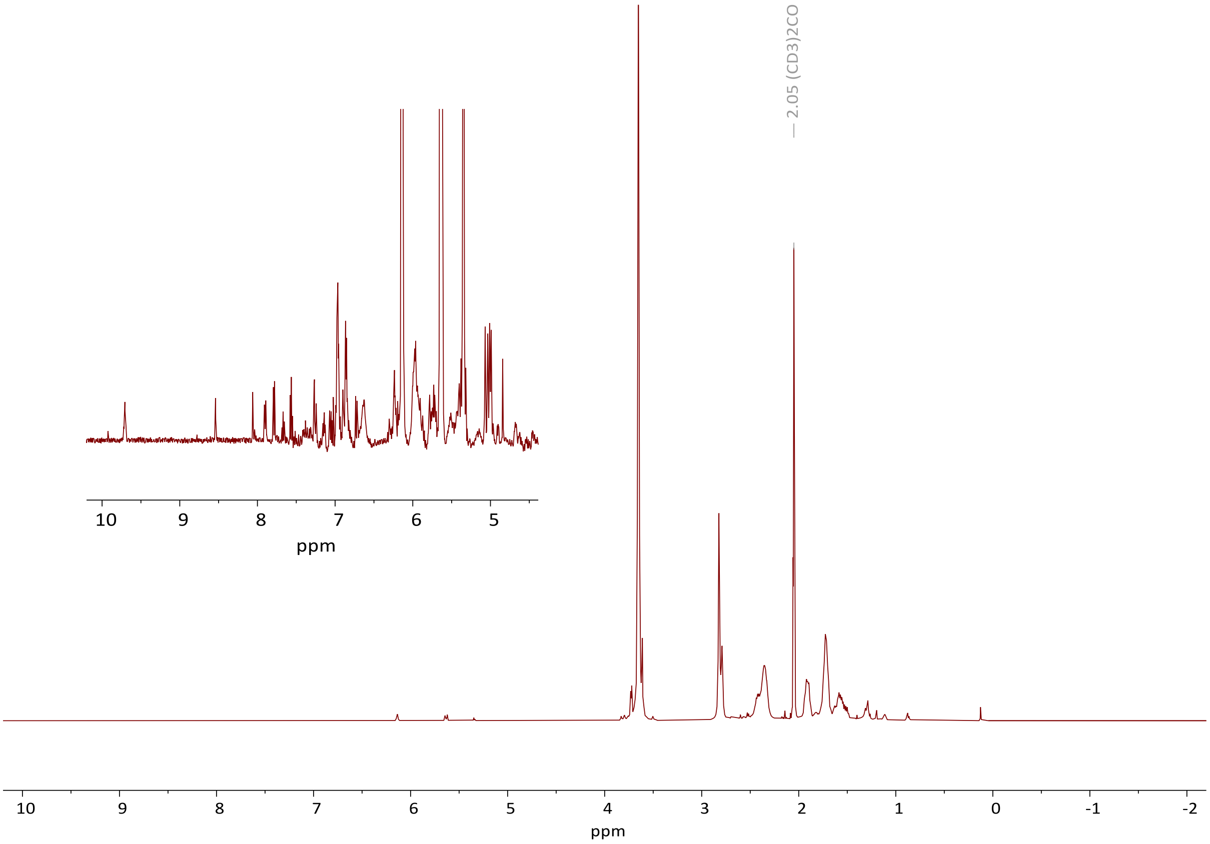

Spectrum S93. ^{1}H NMR (500 MHz, Acetone-d_6, 298 K) spectrum of the concentrated methanol washings from post-BMG polymer **5**$_{\text{-155}}$.

9.5.3 Post-BMG (Wet) ^{1}H NMR Spectra of Polymer $\mathbf{5_{-155}}$ (Run 1)

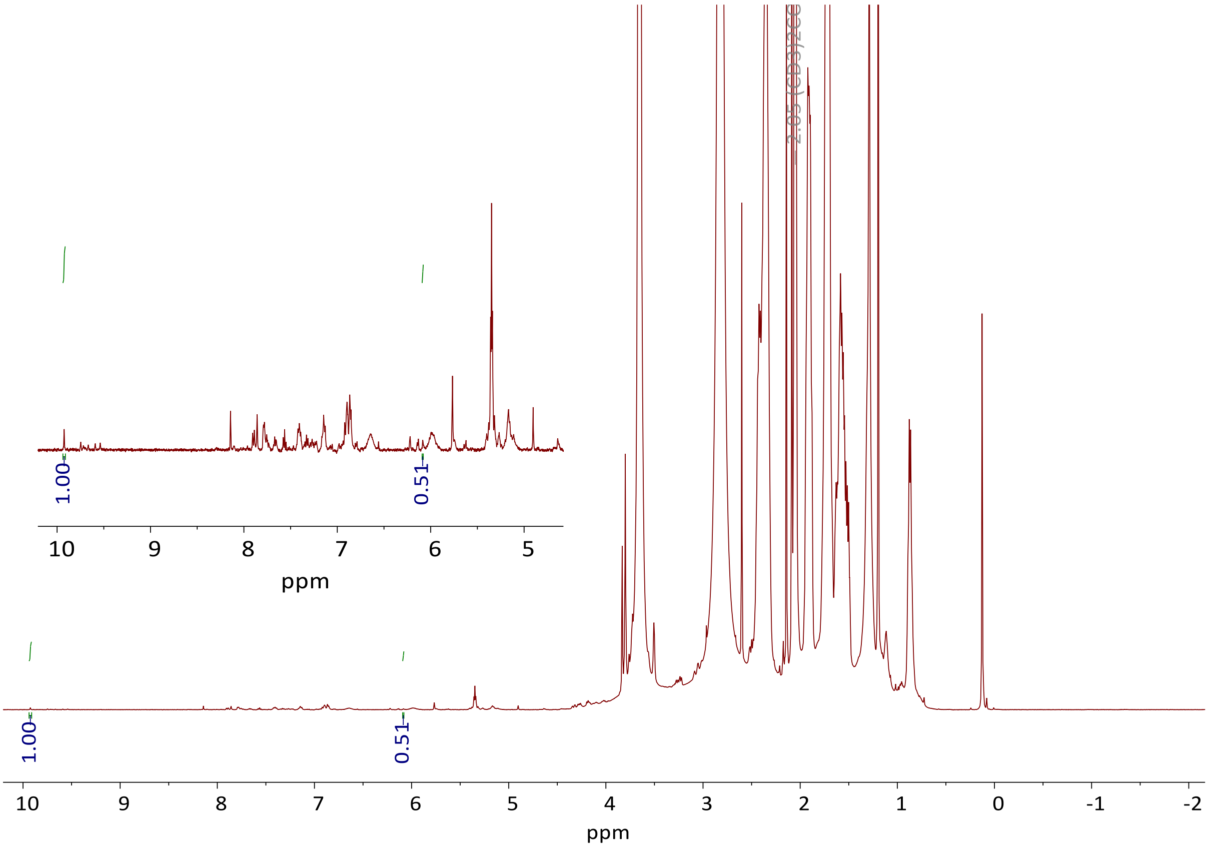

Spectrum S94. ^{1}H NMR (500 MHz, Acetone-d_6, 298 K) spectrum of post-BMG polymer $\mathbf{5_{-155}}$ before being washed with methanol.

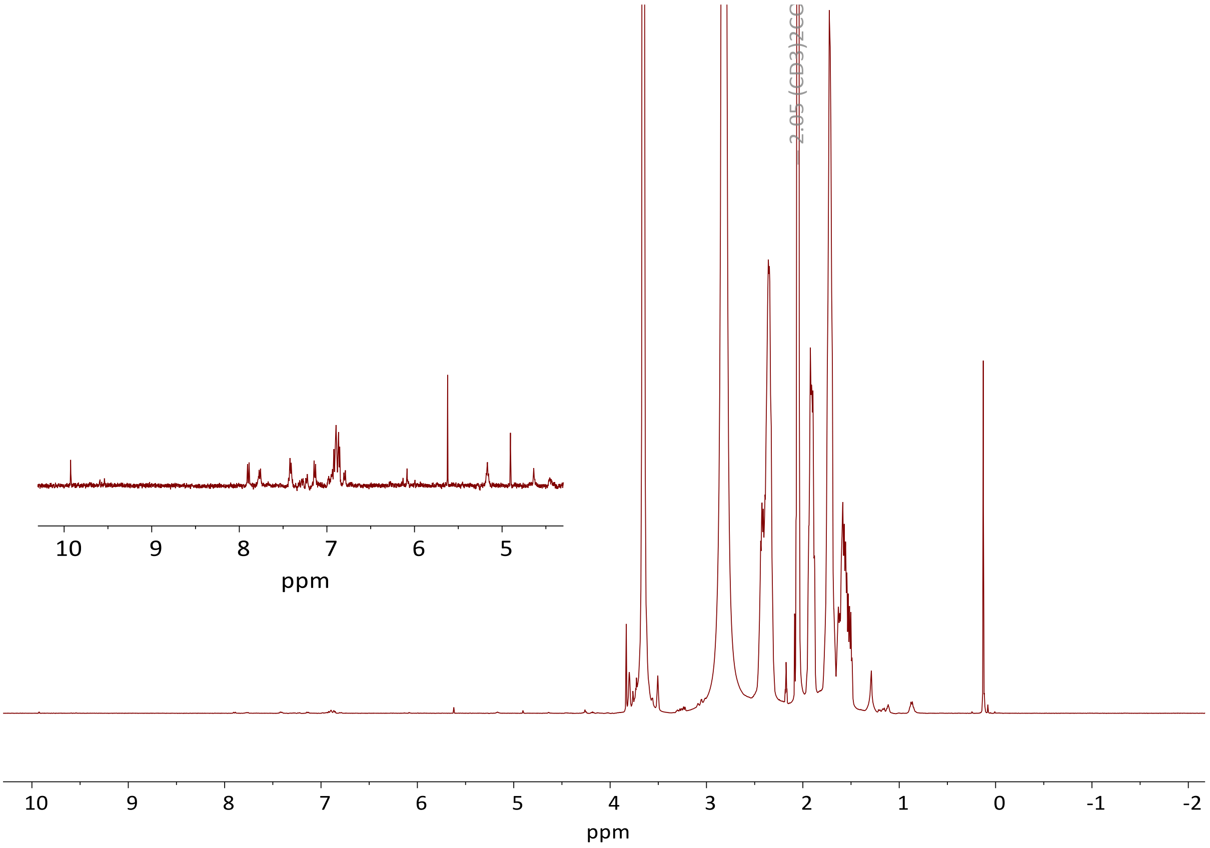

Spectrum S95. ^{1}H NMR (500 MHz, Acetone-d_6, 298 K) spectrum of post-BMG polymer **5$_{155}$** after being washed with methanol.

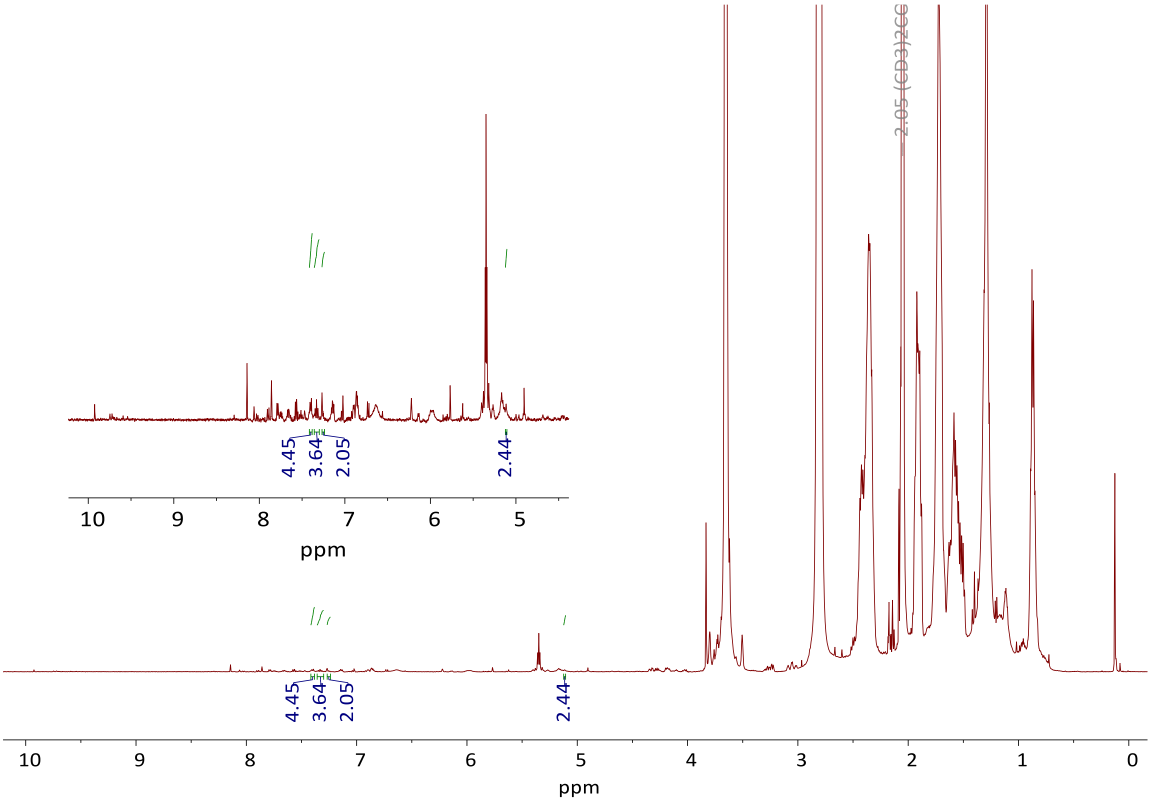

Spectrum S96. ^{1}H NMR (500 MHz, Acetone-d_6, 298 K) spectrum of the concentrated methanol washings from post-BMG polymer **5$_{155}$**.

9.5.4 Post-BMG (Wet) ^{1}H NMR Spectra of Polymer 5$_{155}$ (Run 2)

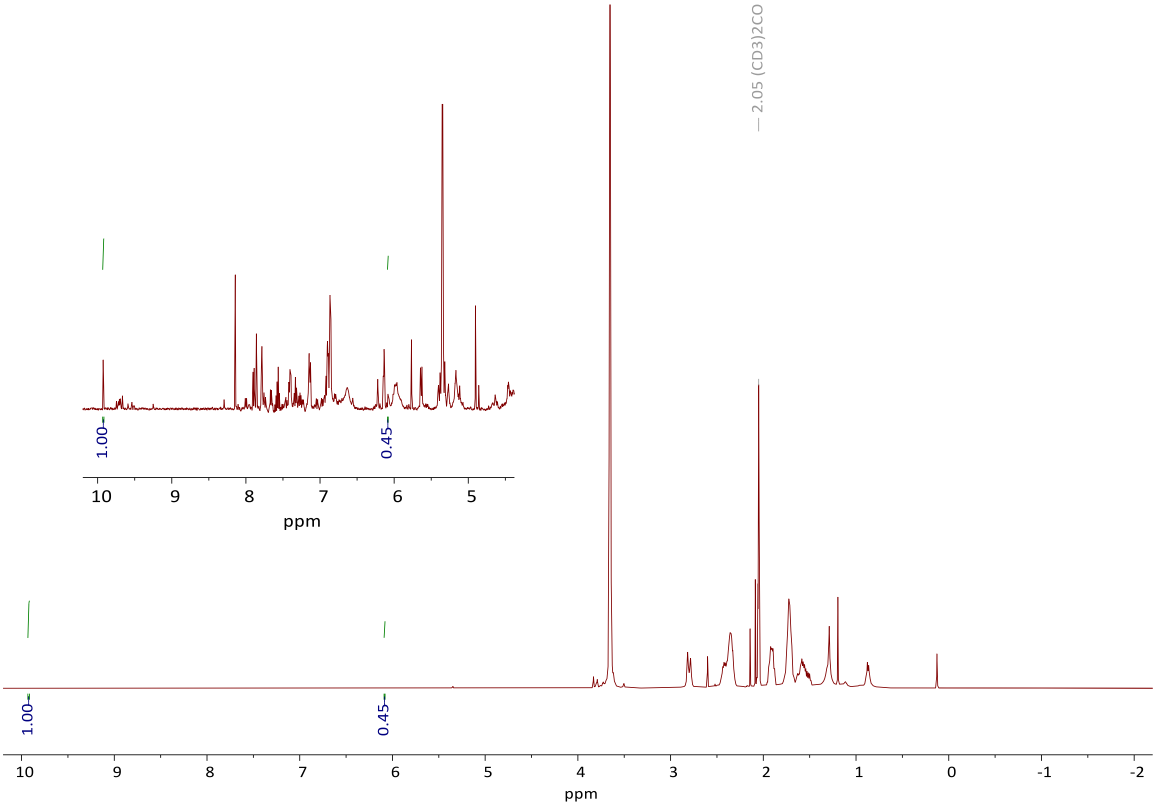

Spectrum S97. ^{1}H NMR (500 MHz, Acetone-d_6, 298 K) spectrum of post-BMG polymer **5**$_{\textbf{-155}}$ before being washed with methanol.

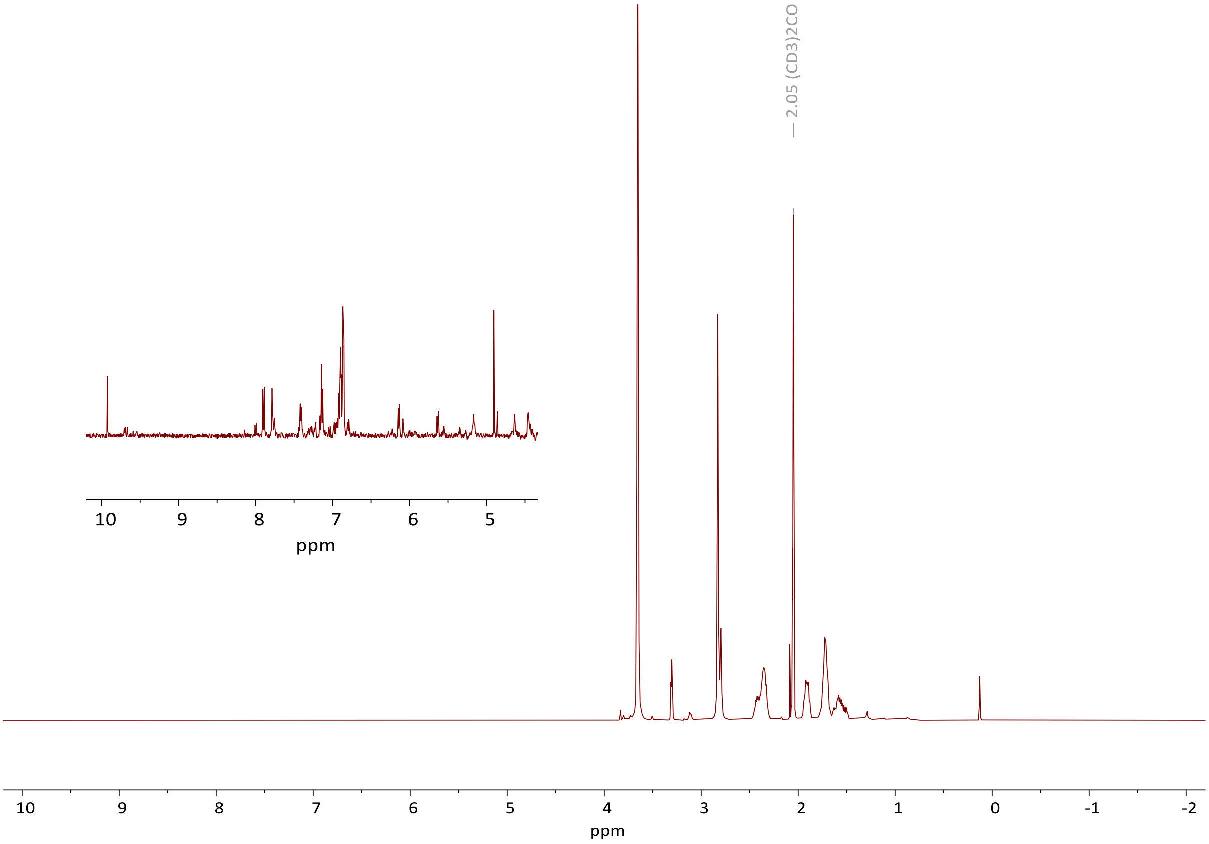

Spectrum S98. ^{1}H NMR (500 MHz, Acetone-d_6, 298 K) spectrum of post-BMG polymer **5**$_{\textbf{-155}}$ after being washed with methanol.

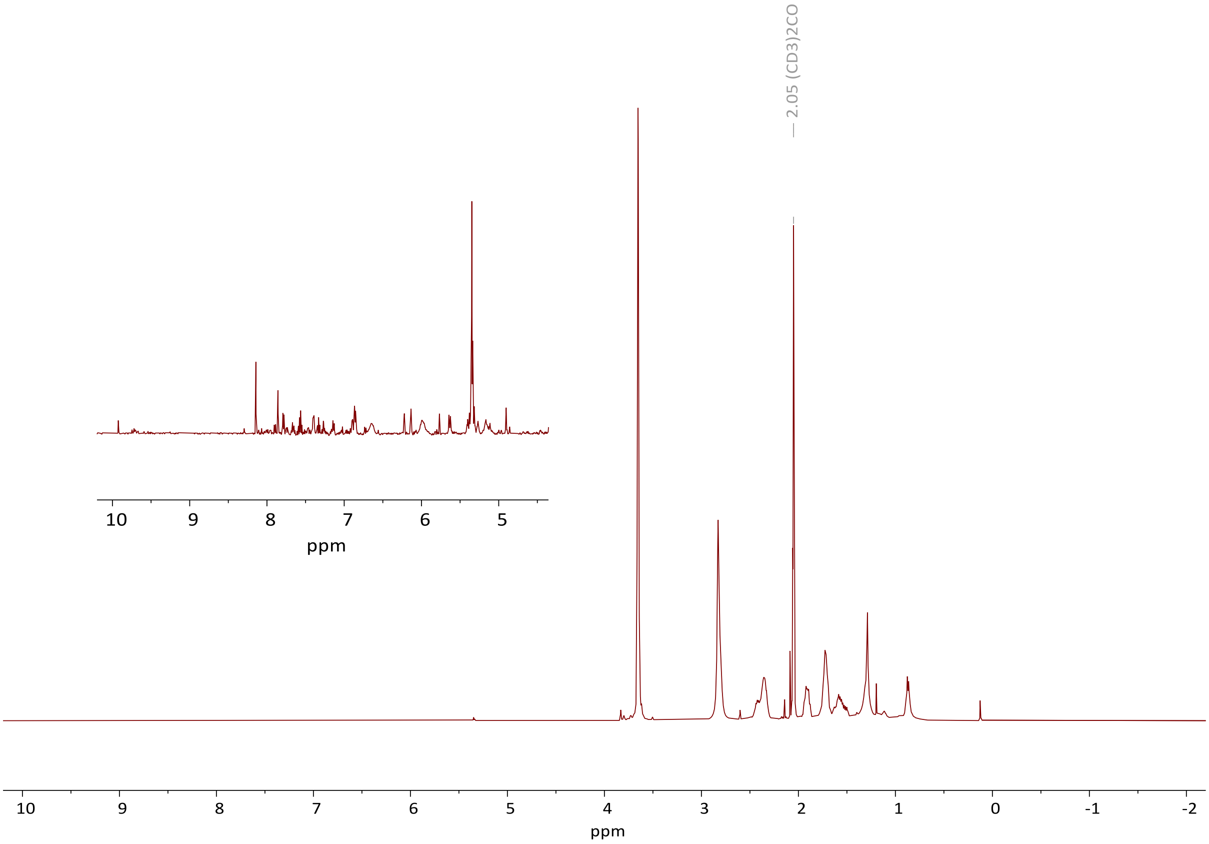

Spectrum S99. ^{1}H NMR (500 MHz, Acetone-d_6, 298 K) spectrum of the concentrated methanol washings from

post-BMG polymer **5**$_{\text{-155}}$.

9.5.5 Post-BMG (Wet) ^{1}H NMR Spectra of Polymer 1$_{HYM}$ (Run 1)

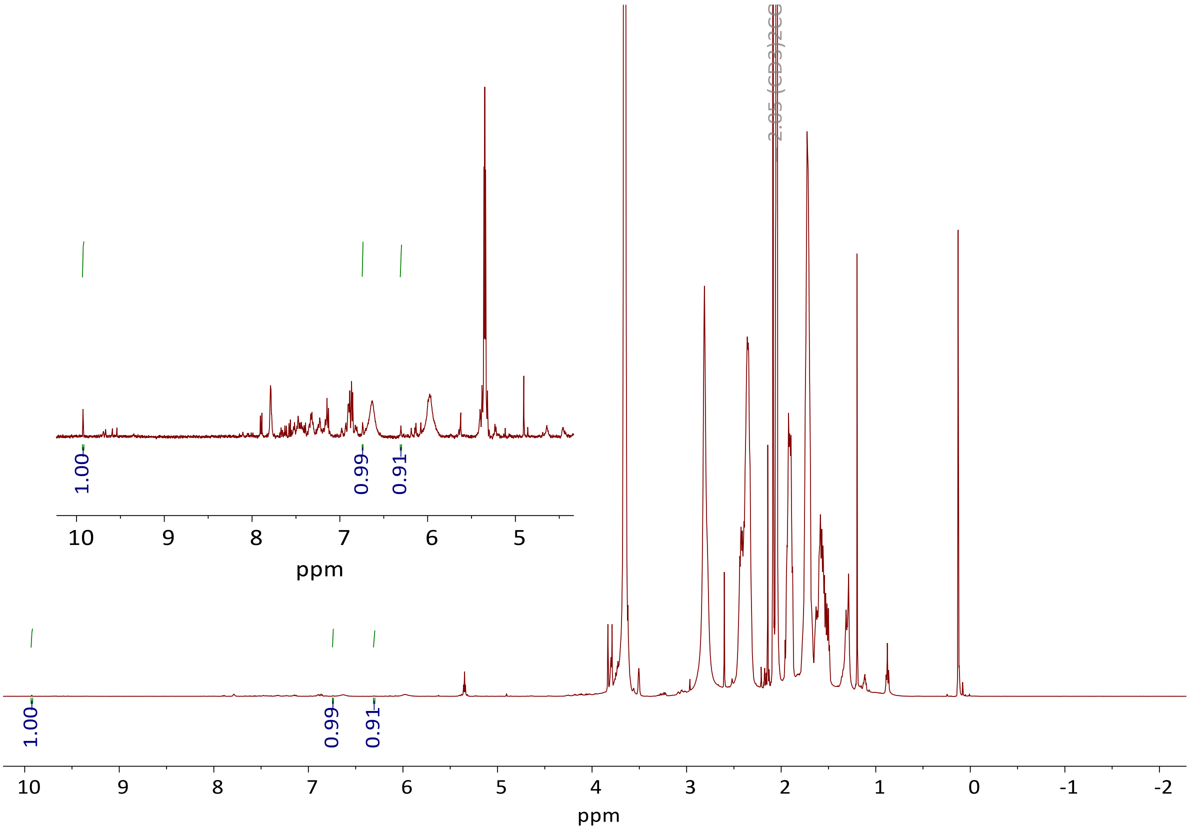

Spectrum S100. ^{1}H NMR (500 MHz, Acetone-d_6, 298 K) spectrum of post-BMG polymer **1**$_{HYM}$ before being washed with methanol.

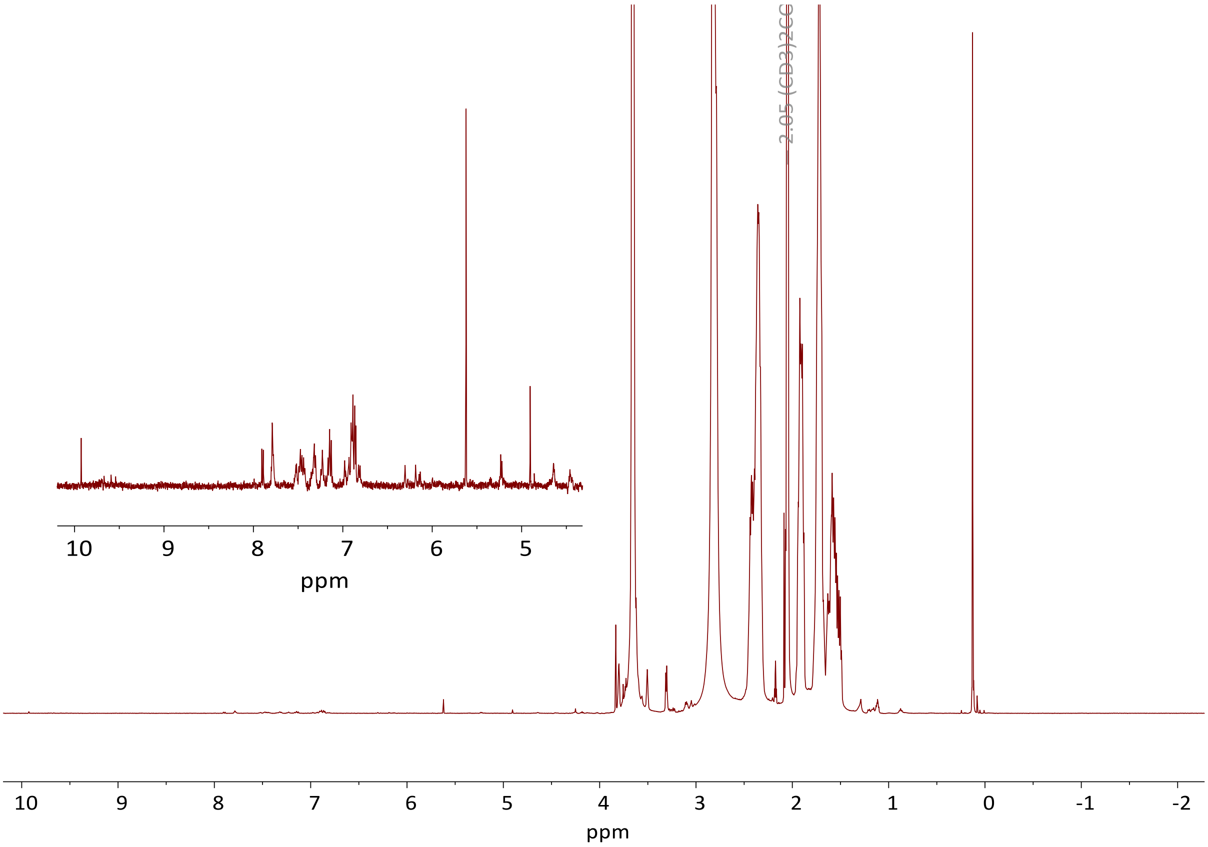

Spectrum S101. ^{1}H NMR (500 MHz, Acetone-d_6, 298 K) spectrum of post-BMG polymer $\mathbf{1}_{\boldsymbol{HYM}}$ after being washed with methanol.

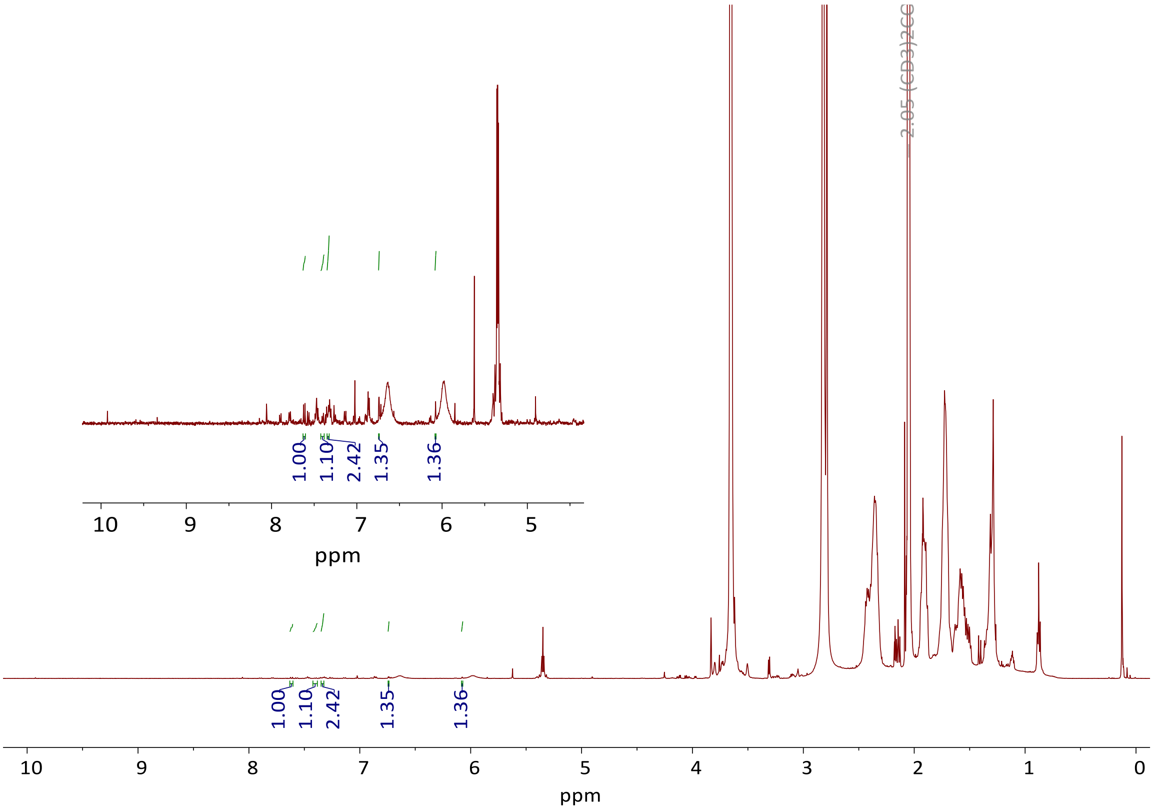

Spectrum S102. ^{1}H NMR (500 MHz, Acetone-d_6, 298 K) spectrum of the concentrated methanol washings from post-BMG polymer $\mathbf{1}_{\boldsymbol{HYM}}$.

9.5.6 Post-BMG (Wet) ^{1}H NMR Spectra of Polymer $\mathbf{1}_{\boldsymbol{HYM}}$ (Run 2)

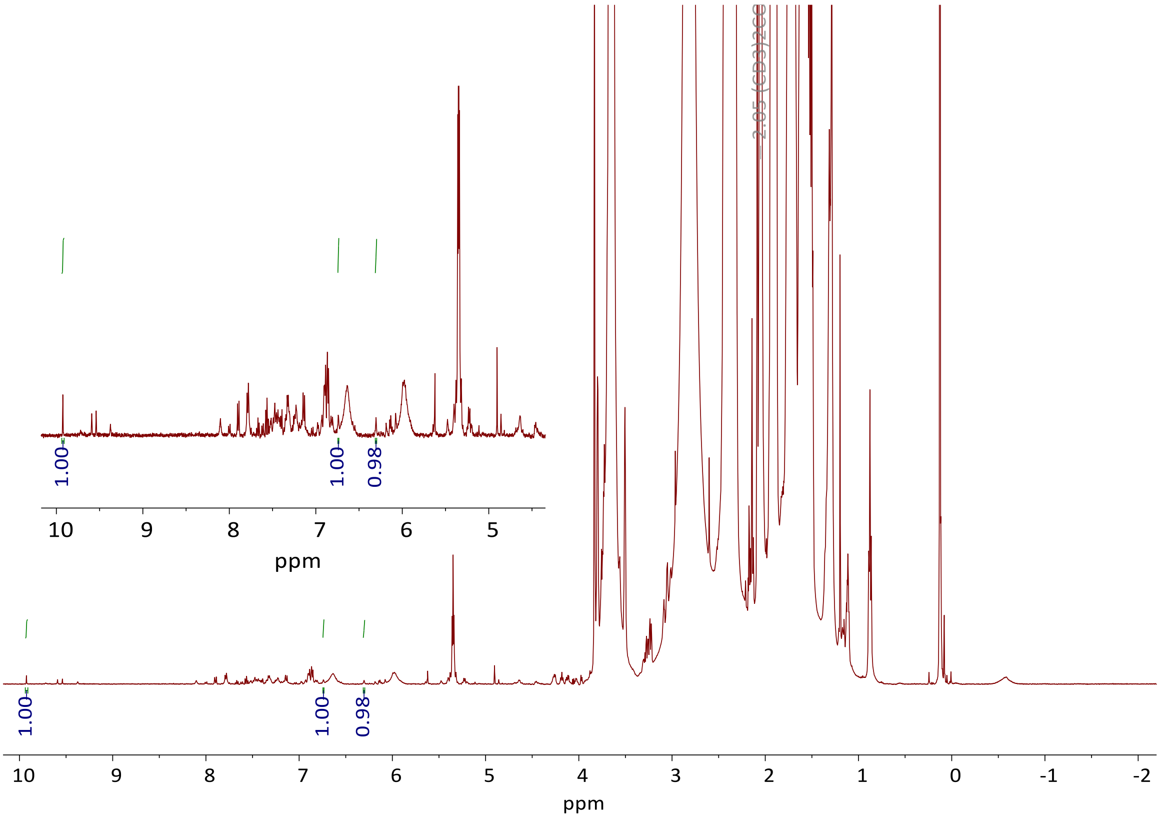

Spectrum S103. ^{1}H NMR (500 MHz, Acetone-d_6, 298 K) spectrum of post-BMG polymer $\mathbf{1}_{\boldsymbol{HYM}}$ before being washed

with methanol.

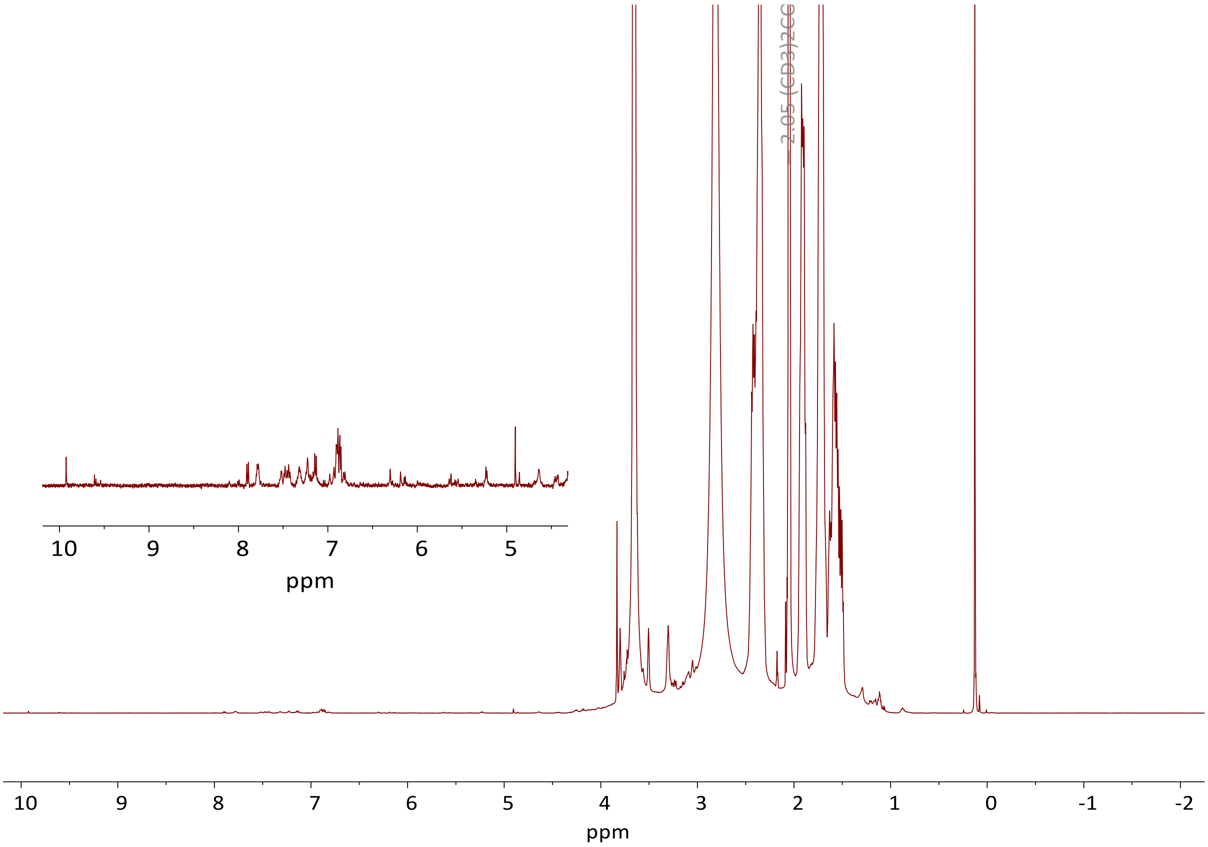

Spectrum S104. ^{1}H NMR (500 MHz, Acetone-d_6, 298 K) spectrum of post-BMG polymer **1*HYM*** after being washed with methanol.

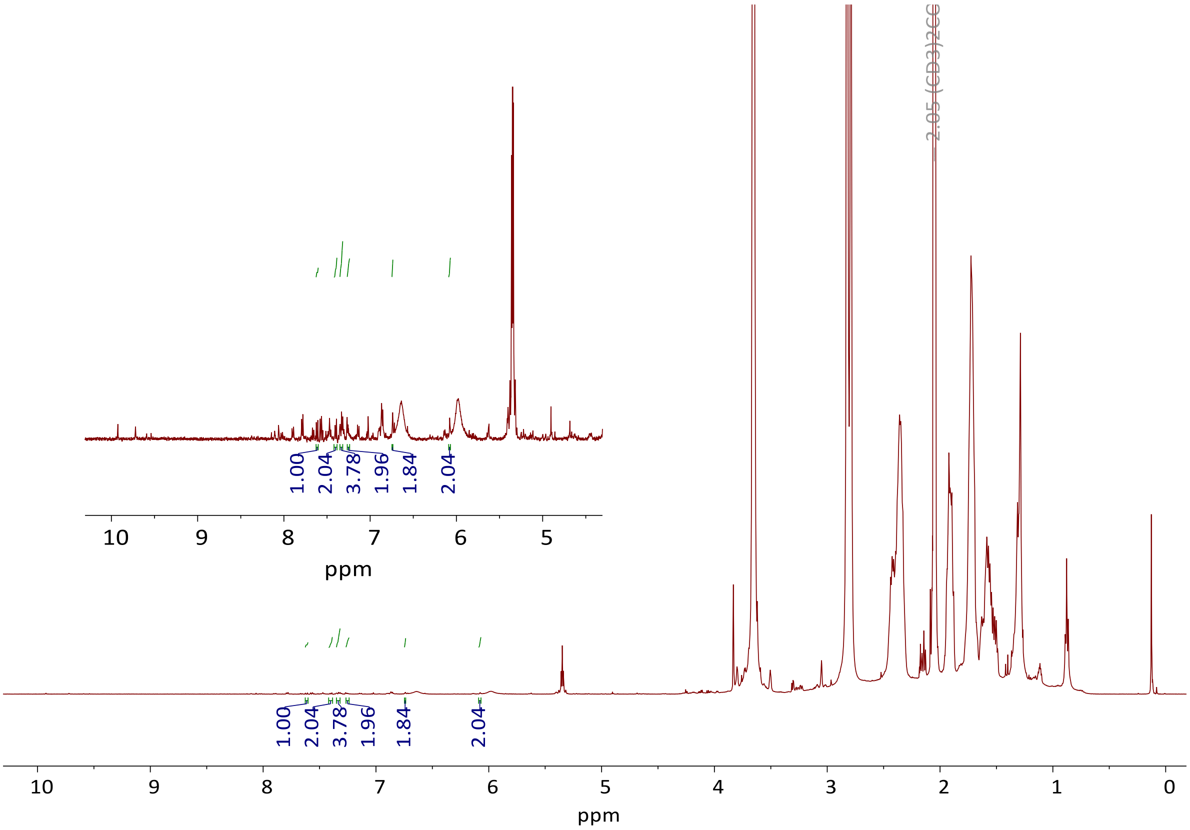

Spectrum S105. ^{1}H NMR (500 MHz, Acetone-d_6, 298 K) spectrum of the concentrated methanol washings from post-BMG polymer **1*HYM***.

9.5.7 Post-BMG (Wet) ^{1}H NMR Spectra of Polymer 1_{GEM} (Run 1)

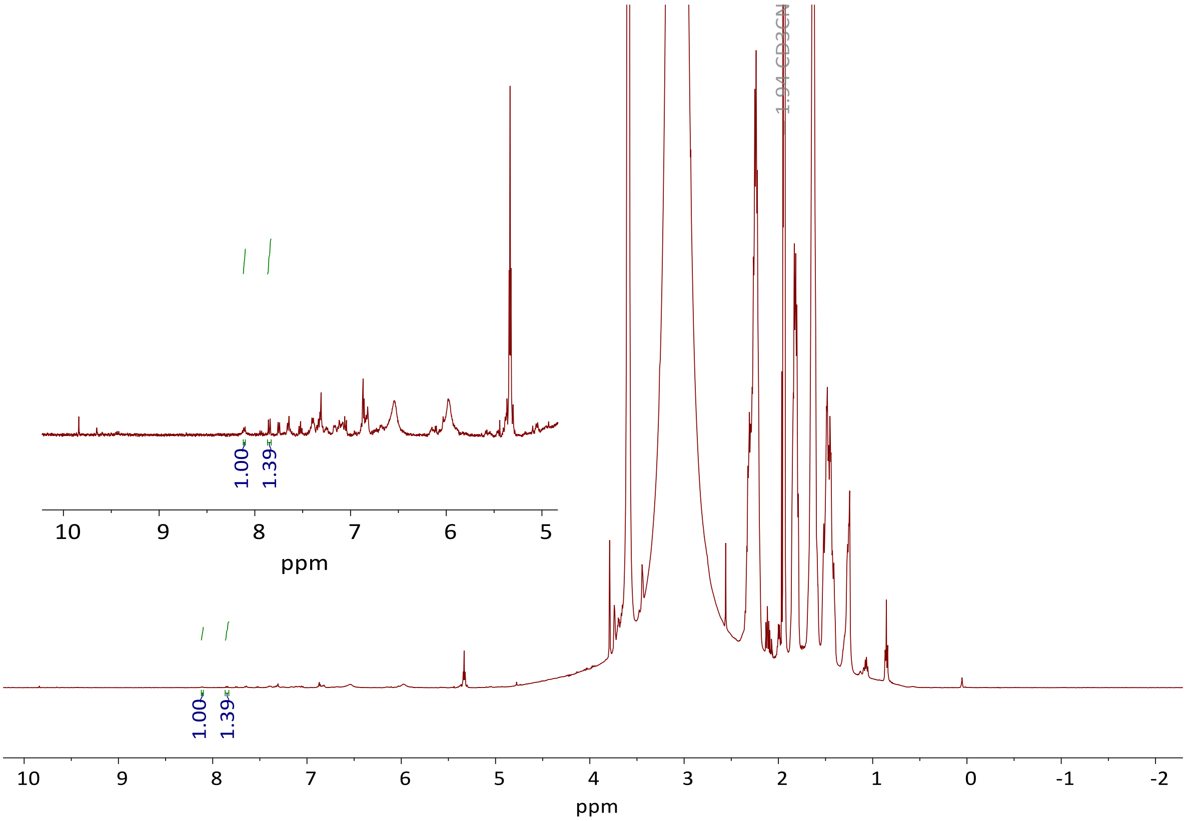

Spectrum S106. ^{1}H NMR (500 MHz, Acetonitrile-d_3/H_2O (9/1), 298 K) spectrum of post-BMG polymer $\mathbf{1}_{GEM}$ before being washed with methanol.

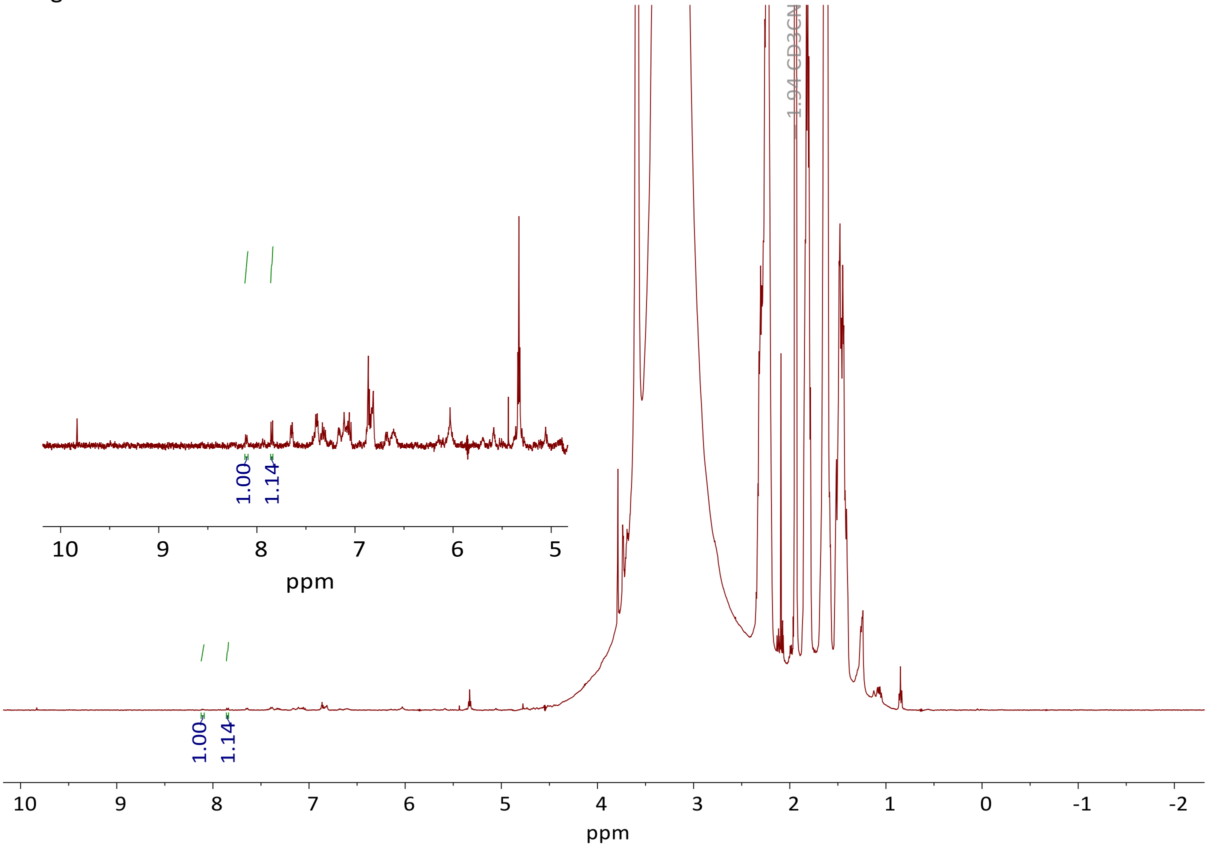

Spectrum S107. ^{1}H NMR (500 MHz, Acetonitrile-d_3/H_2O (9/1), 298 K) spectrum of post-BMG polymer $\mathbf{1}_{GEM}$ after

being washed with methanol.

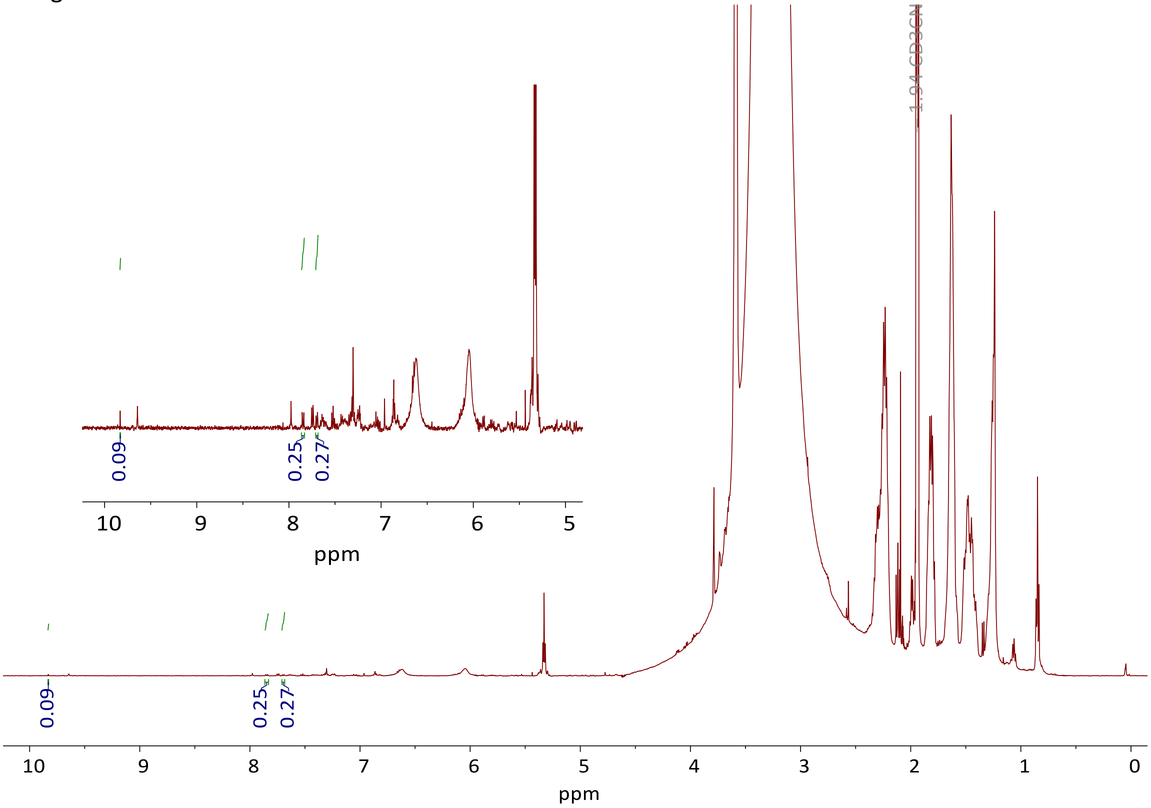

Spectrum S108. ^{1}H NMR (500 MHz, Acetonitrile-*d*$_3$/H_2O (9/1), 298 K) spectrum of the concentrated methanol washings from post-BMG polymer **1***$_{GEM}$*.

9.5.8 Post-BMG (Wet) ^{1}H NMR Spectra of Polymer 1$_{GEM}$ (Run 2)

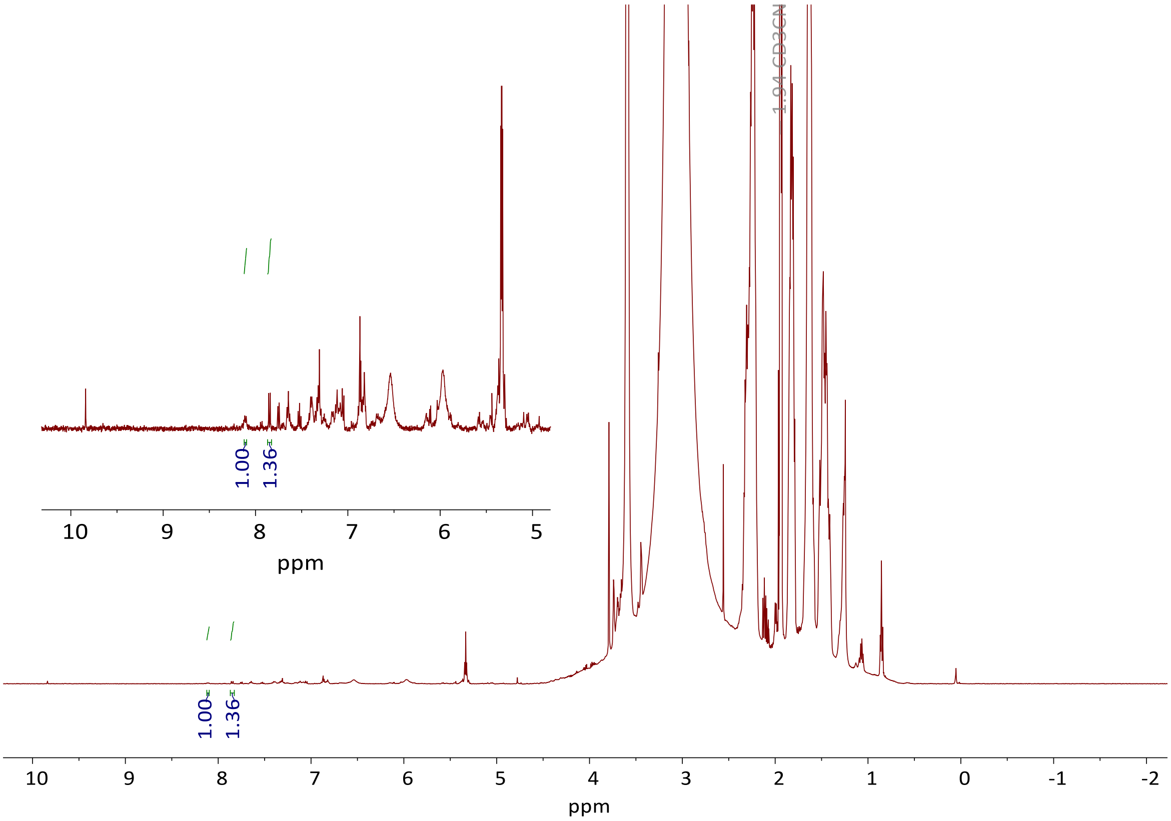

Spectrum S109. ^{1}H NMR (500 MHz, Acetonitrile-*d*$_3$/H_2O (9/1), 298 K) spectrum of post-BMG polymer **1***$_{GEM}$* before being washed with methanol.

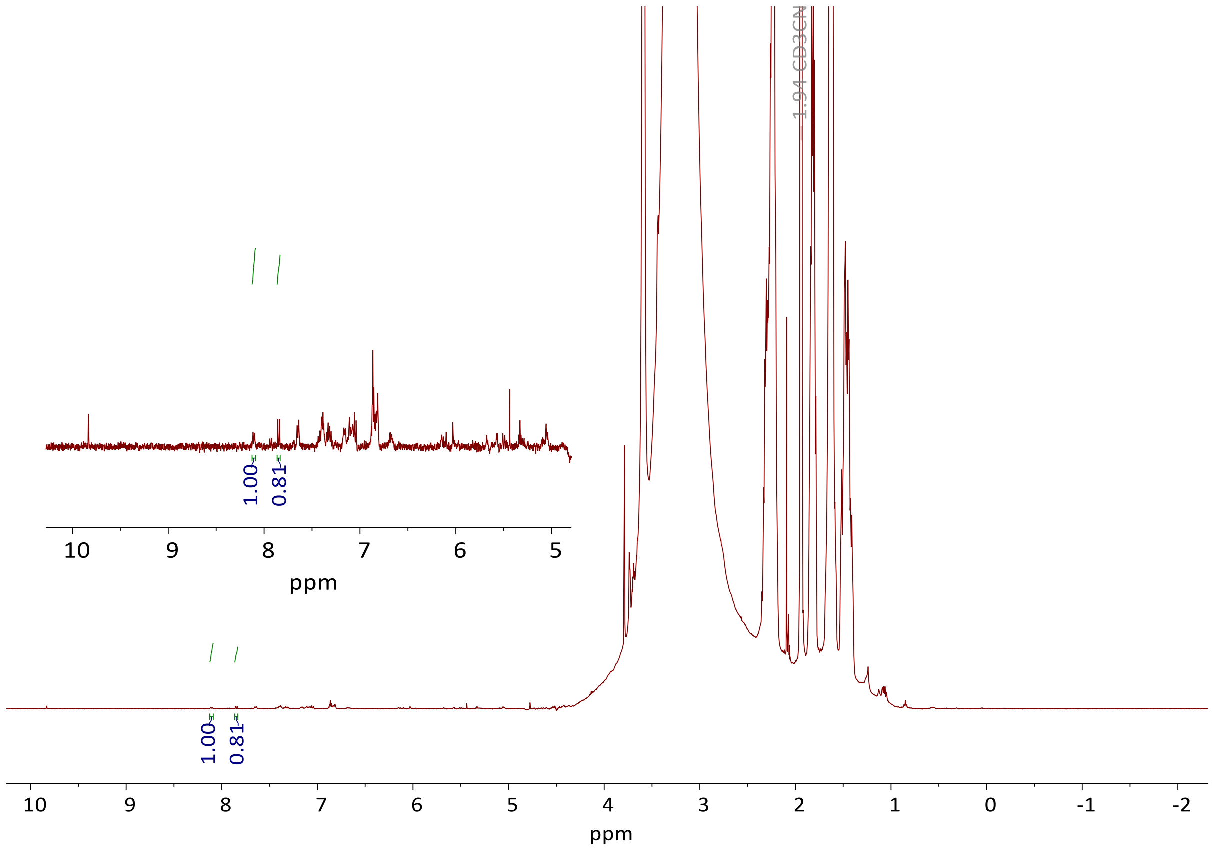

Spectrum S110. ^{1}H NMR (500 MHz, Acetonitrile-d_3/H_2O (9/1), 298 K) spectrum of post-BMG polymer **1*GEM*** after being washed with methanol.

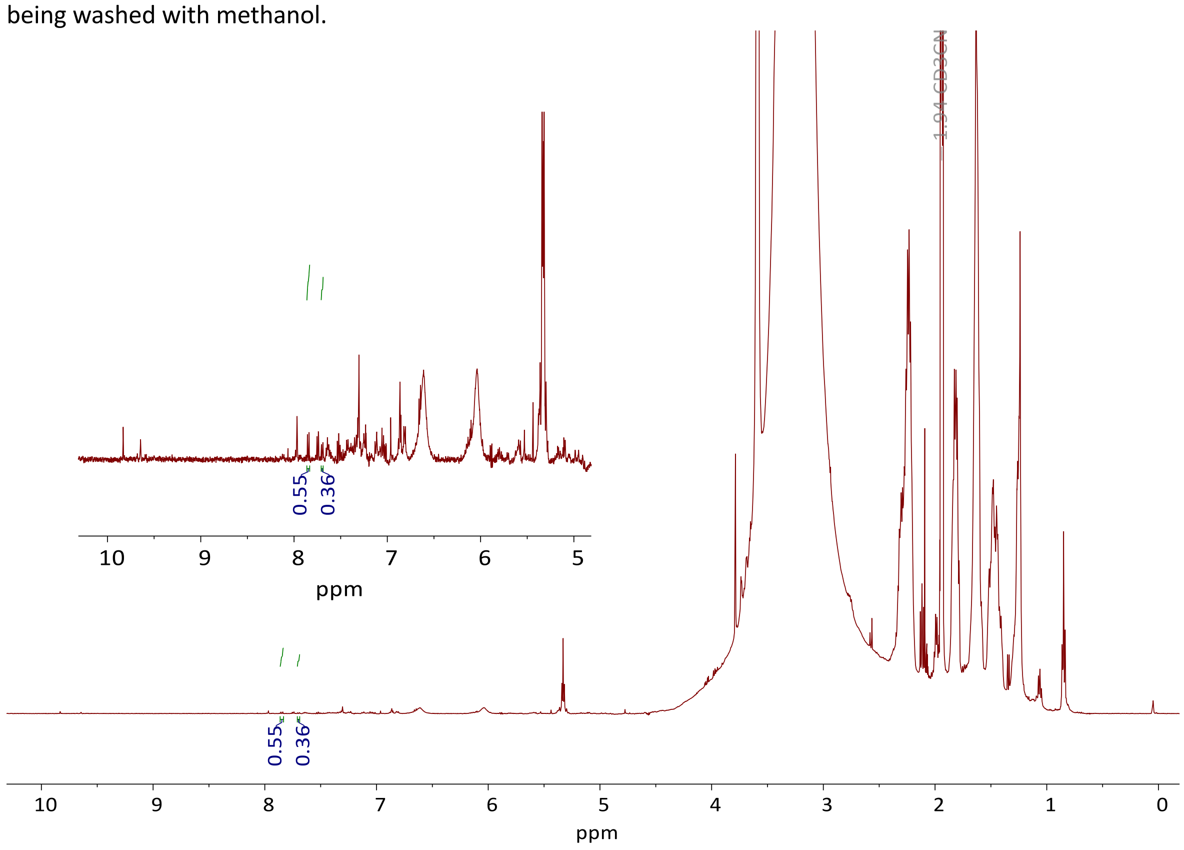

Spectrum S111. ^{1}H NMR (500 MHz, Acetonitrile-d_3/H_2O (9/1), 298 K) spectrum of the concentrated methanol washings from post-BMG polymer **1*GEM***.

9.5.9 Post-BMG (Dry) ^{1}H NMR Spectra of Polymer S20

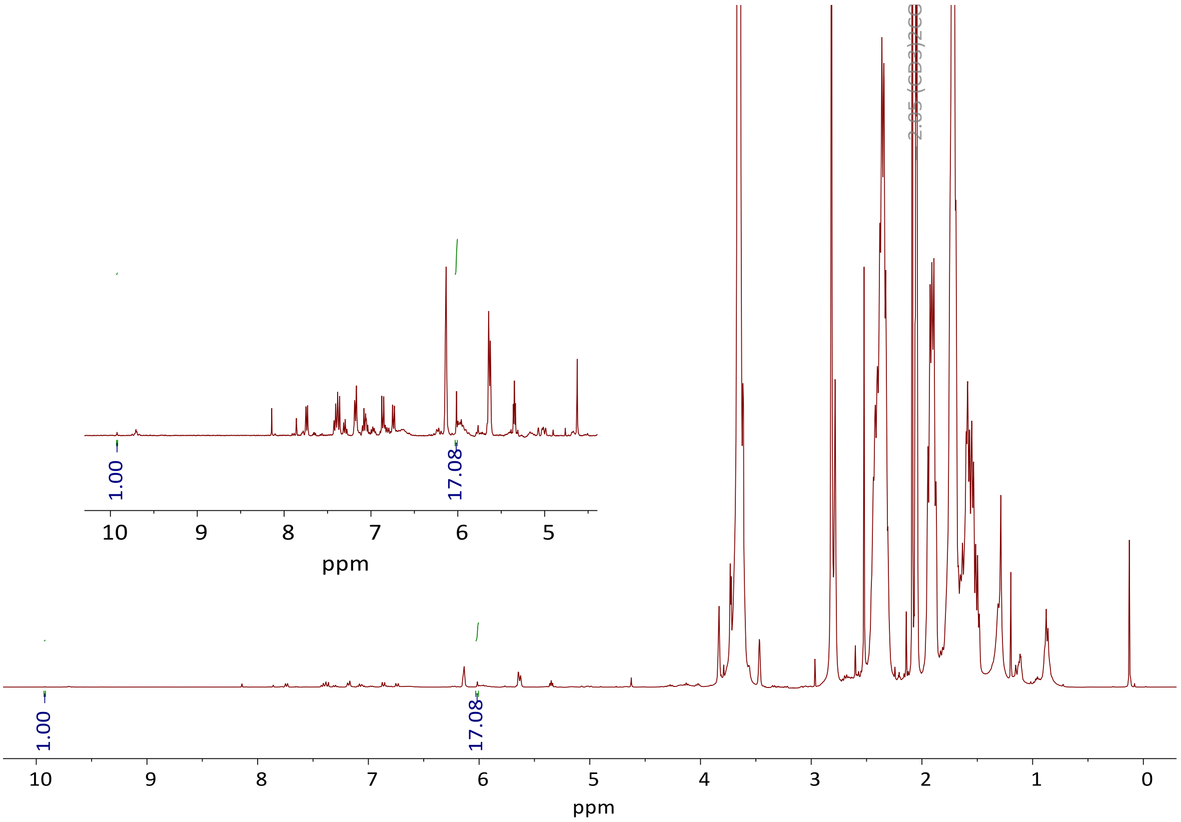

Spectrum S112. ^{1}H NMR (400 MHz, Acetone-d_6, 298 K) spectrum of post-BMG polymer **S20** before being washed with methanol.

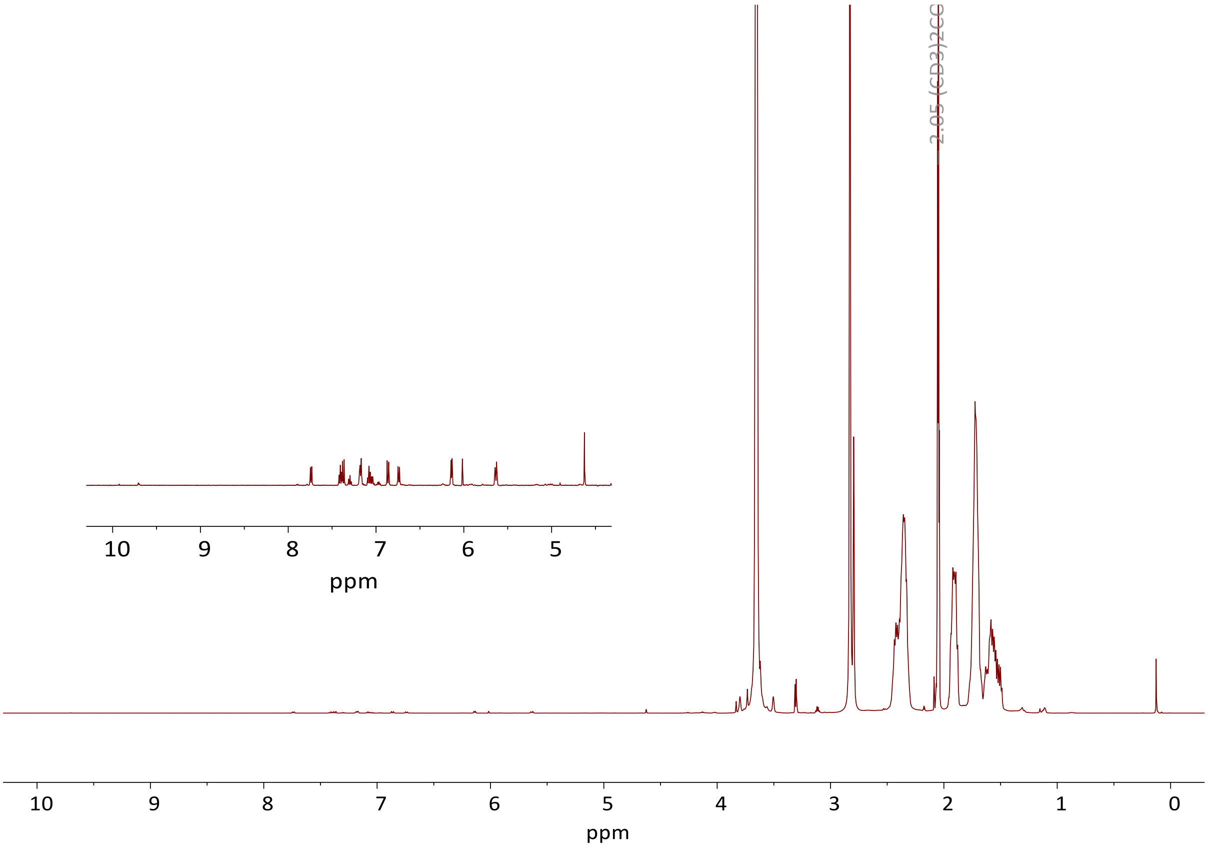

Spectrum S113. ^{1}H NMR (500 MHz, Acetone-d_6, 298 K) spectrum of post-BMG polymer **S20** after being washed with methanol.

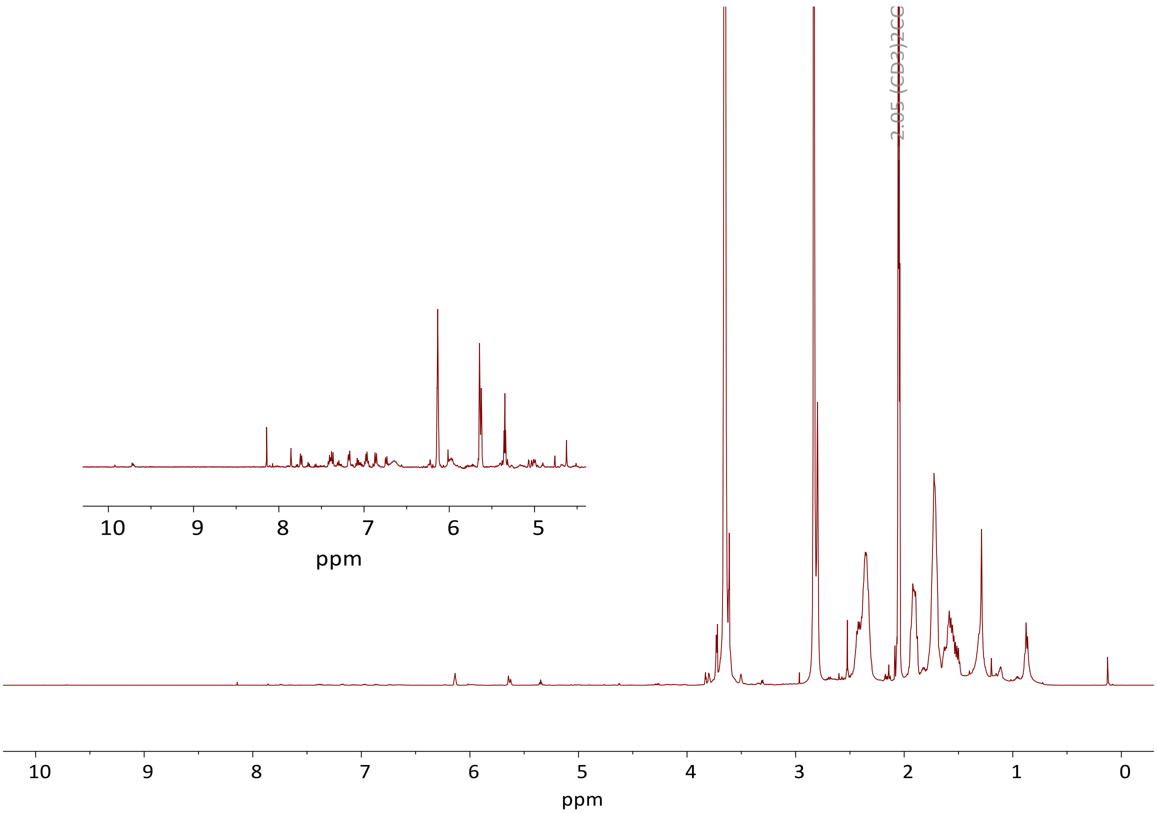

Spectrum S114. ^{1}H NMR (500 MHz, Acetone-d_6, 298 K) spectrum of the concentrated methanol washings from post-BMG polymer **S20**.

9.5.10 Post-BMG (Wet) ^{1}H NMR Spectra of Polymer S20

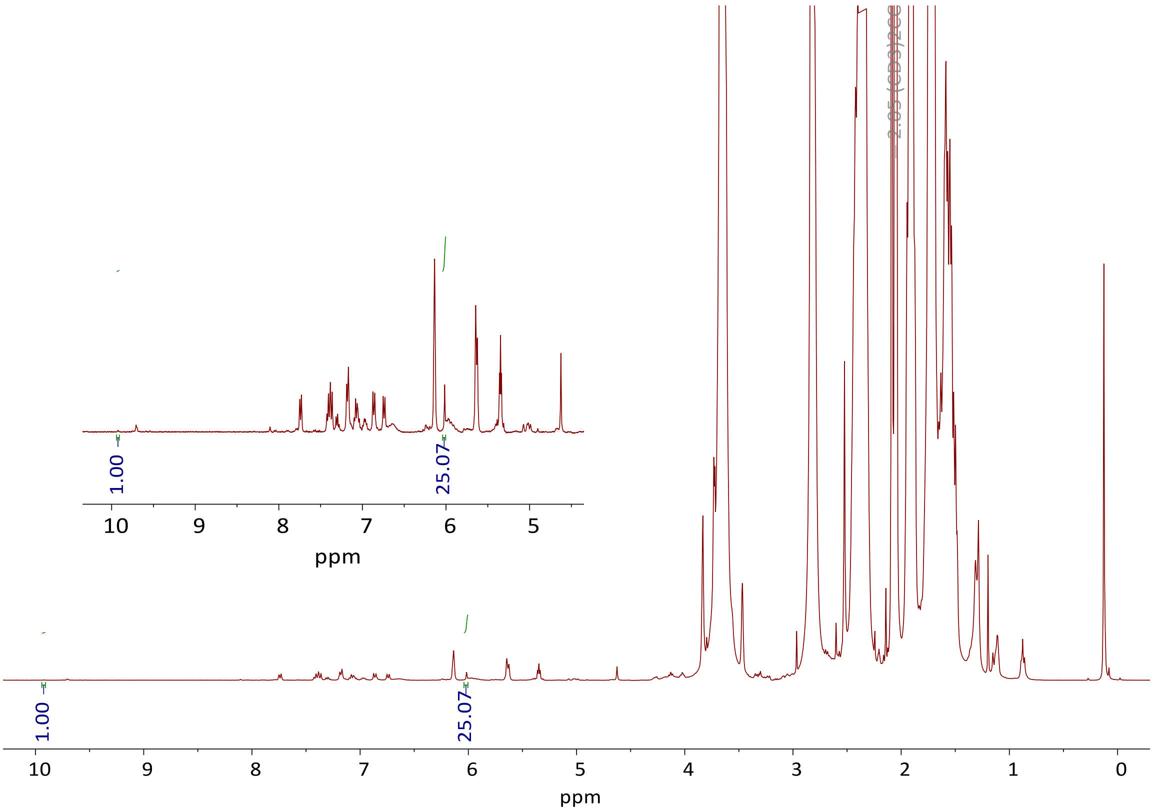

Spectrum S115. ^{1}H NMR (400 MHz, Acetone-d_6, 298 K) spectrum of post-BMG polymer **S20** before being washed with methanol.

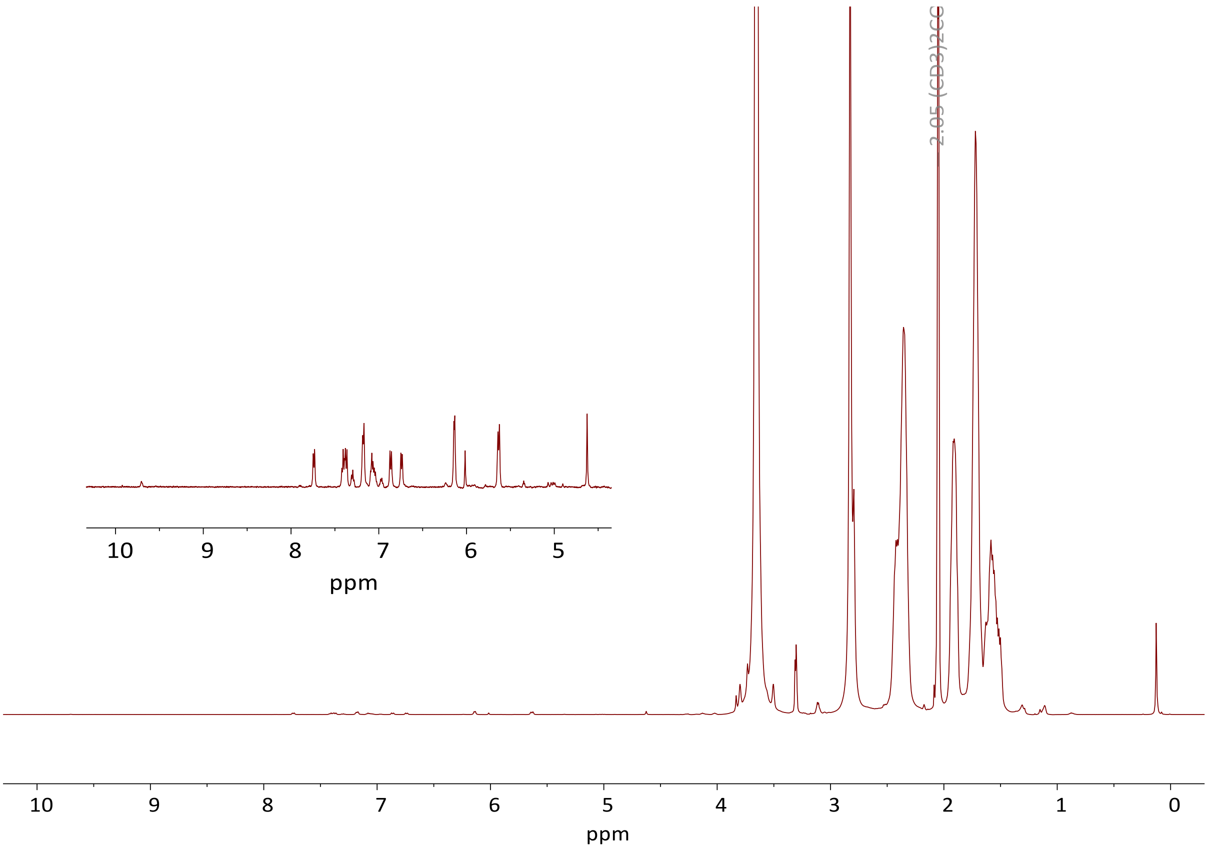

Spectrum S116. ^{1}H NMR (500 MHz, Acetone-d_6, 298 K) spectrum of post-BMG polymer **S20** after being washed with methanol.

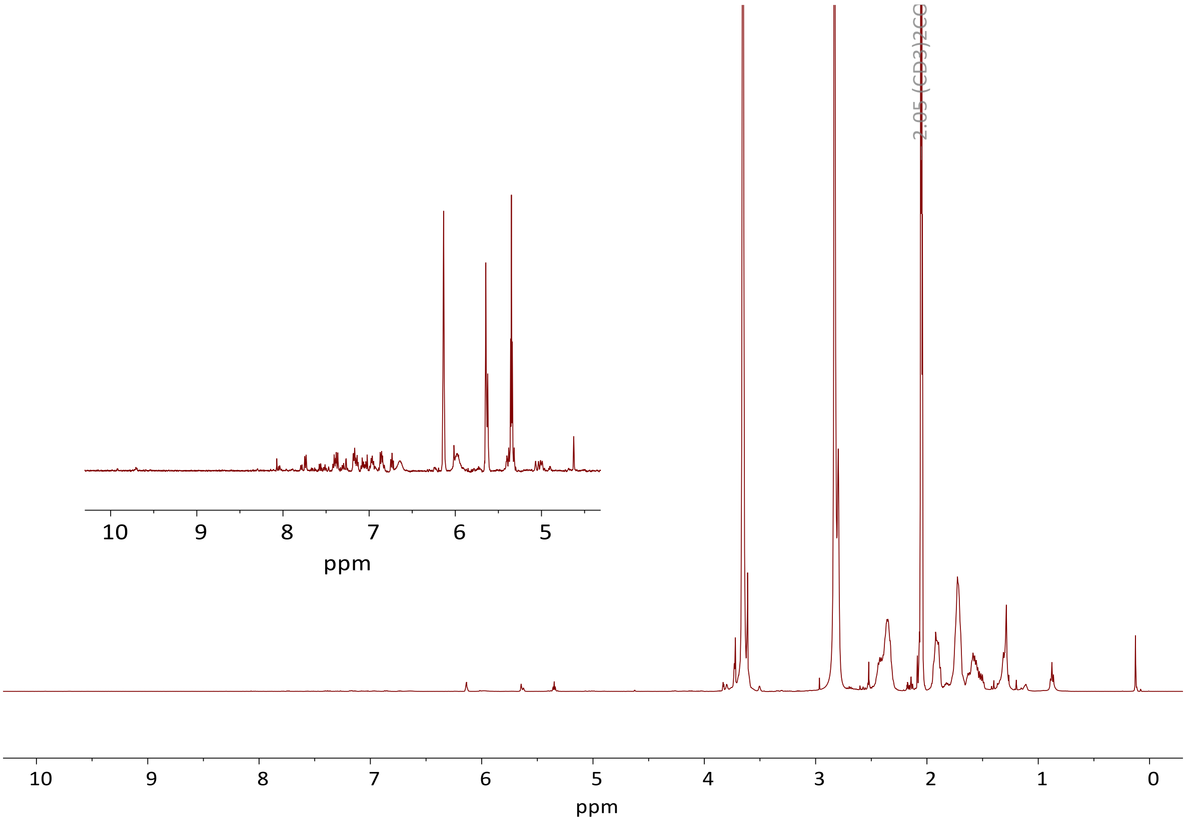

Spectrum S117. ^{1}H NMR (500 MHz, Acetone-d_6, 298 K) spectrum of the concentrated methanol washings from post-BMG polymer **S20**.

9.5.11 Post-BMG (Wet) ^{1}H NMR Spectra of Polymer S21

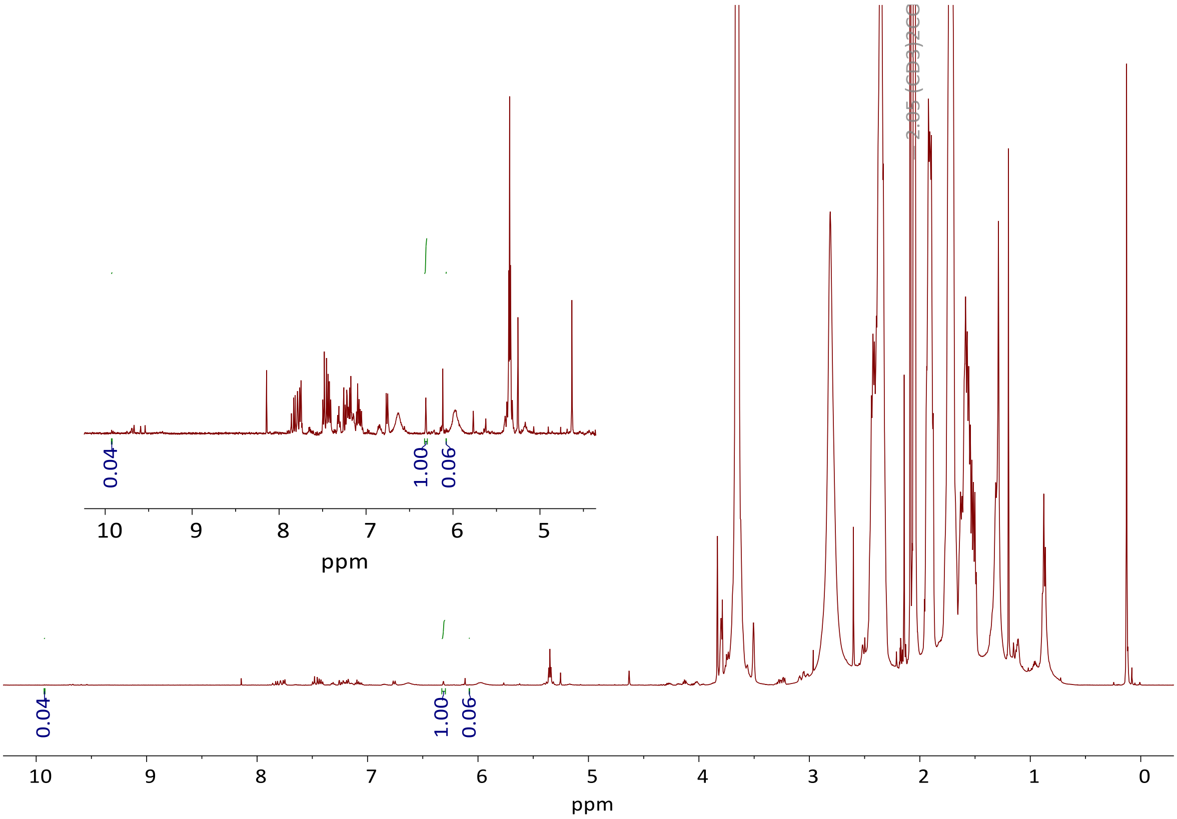

Spectrum S118. ^{1}H NMR (500 MHz, Acetone-d_6, 298 K) spectrum of post-BMG polymer **S21** before being washed with methanol.

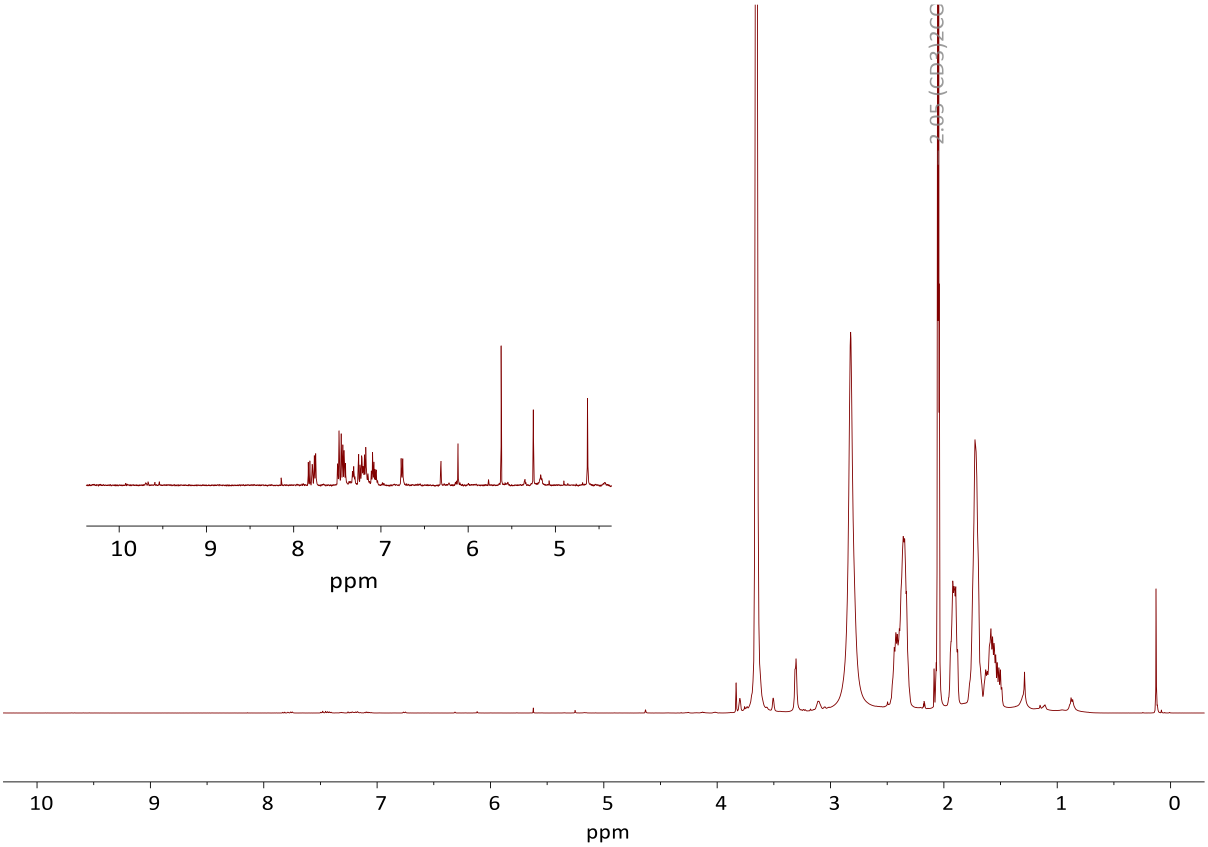

Spectrum S119. ^{1}H NMR (500 MHz, Acetone-d_6, 298 K) spectrum of post-BMG polymer **S21** after being washed with methanol.

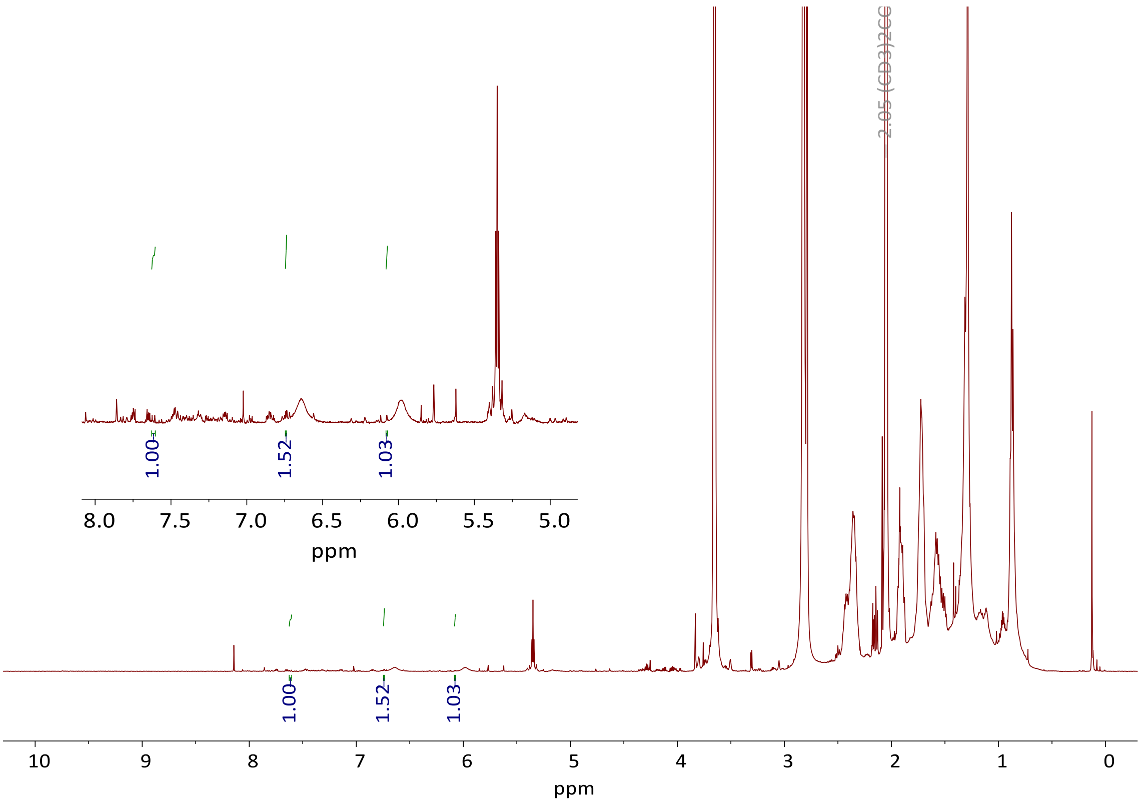

Spectrum S120. ^{1}H NMR (500 MHz, Acetone-d_6, 298 K) spectrum of the concentrated methanol washings from post-BMG polymer **S21**.

9.5.12 Post-BMG (Dry) ^{1}H NMR Spectra of Polymer $\mathbf{9}_{\text{-}114}$

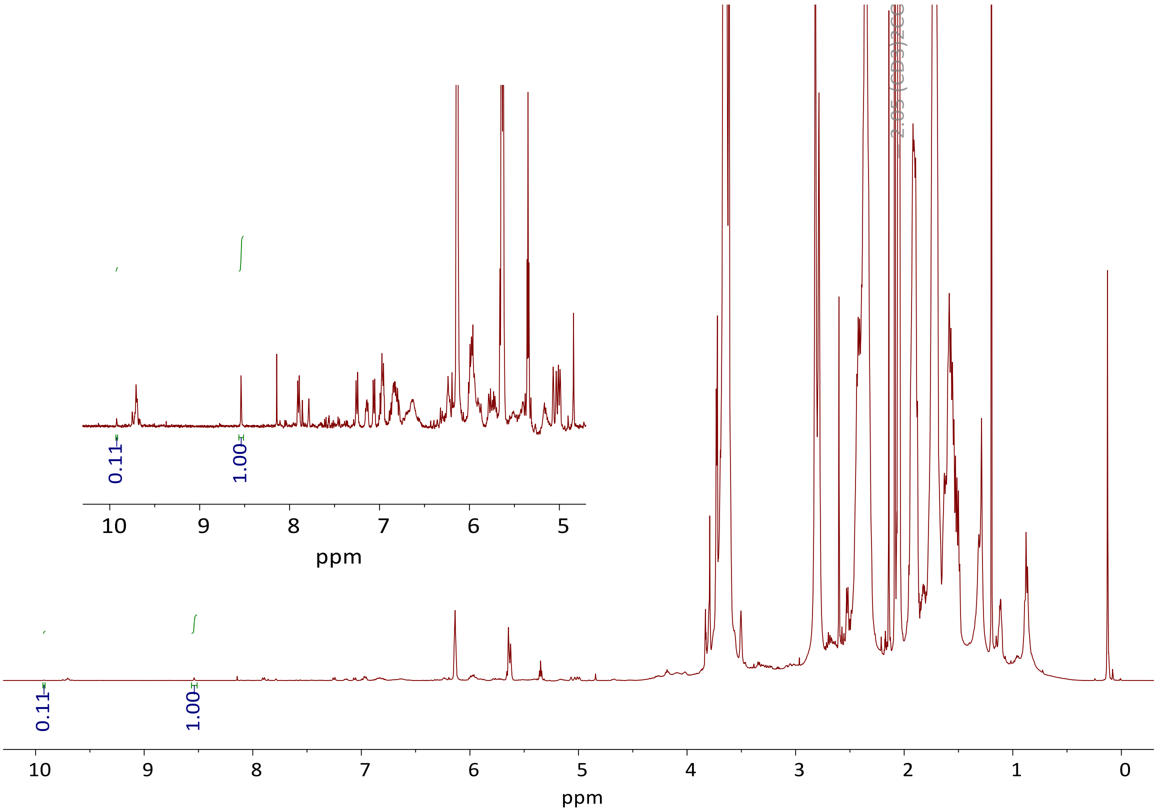

Spectrum S121. ^{1}H NMR (500 MHz, Acetone-d_6, 298 K) spectrum of post-BMG polymer $\mathbf{9}_{\text{-}114}$ before being washed with methanol.

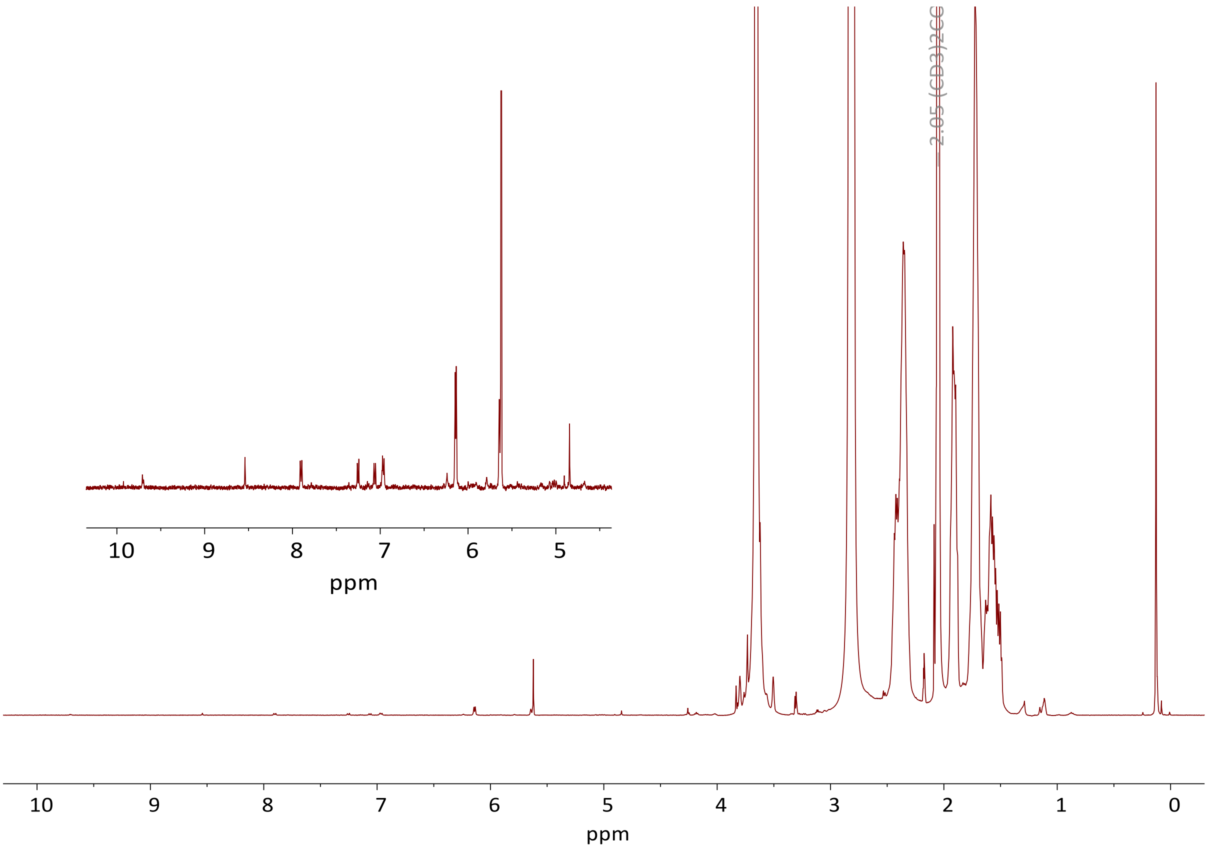

Spectrum S122. ^{1}H NMR (500 MHz, Acetone-d_6, 298 K) spectrum of post-BMG polymer $\mathbf{9}_{\mathbf{\text{-}114}}$ after being washed with methanol.

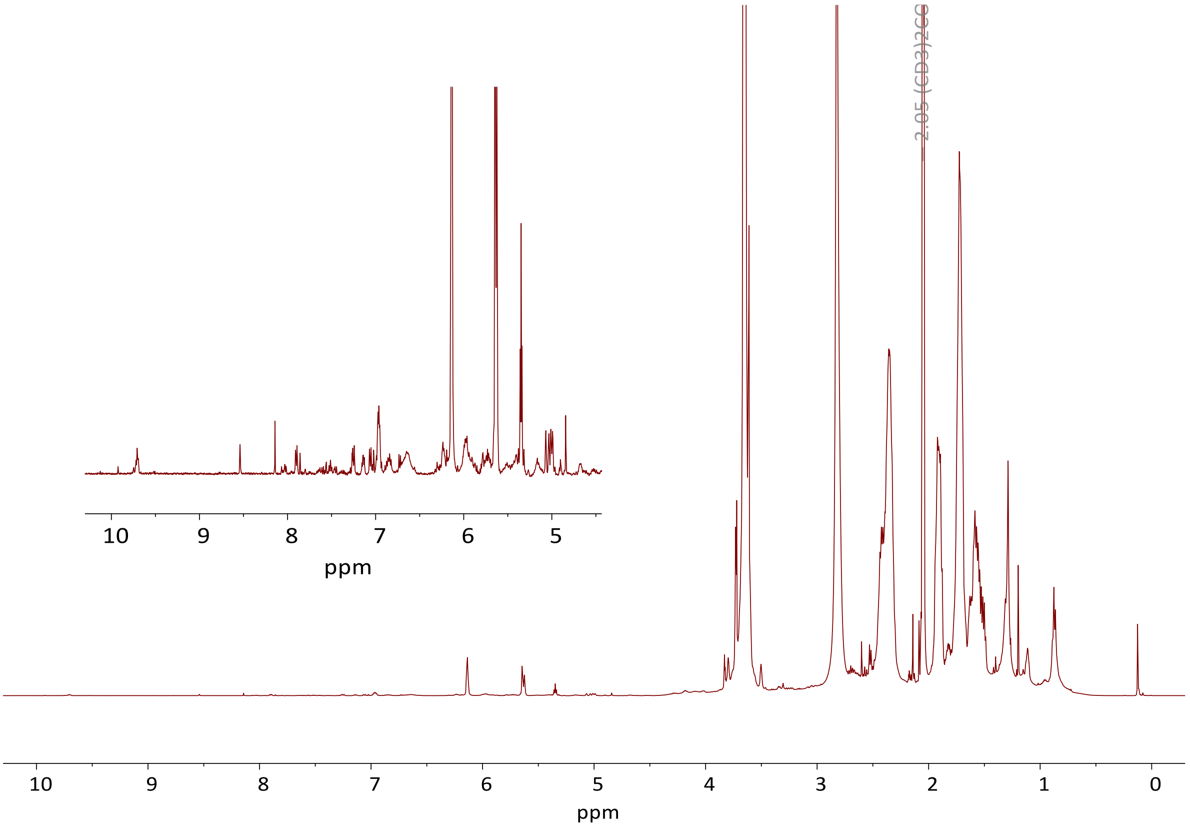

Spectrum S123. ^{1}H NMR (500 MHz, Acetone-d_6, 298 K) spectrum of the concentrated methanol washings from post-BMG polymer $\mathbf{9}_{\mathbf{\text{-}114}}$.

9.5.13 Post-BMG (Wet) ^{1}H NMR Spectra of Polymer $\mathbf{9}_{\text{-}114}$

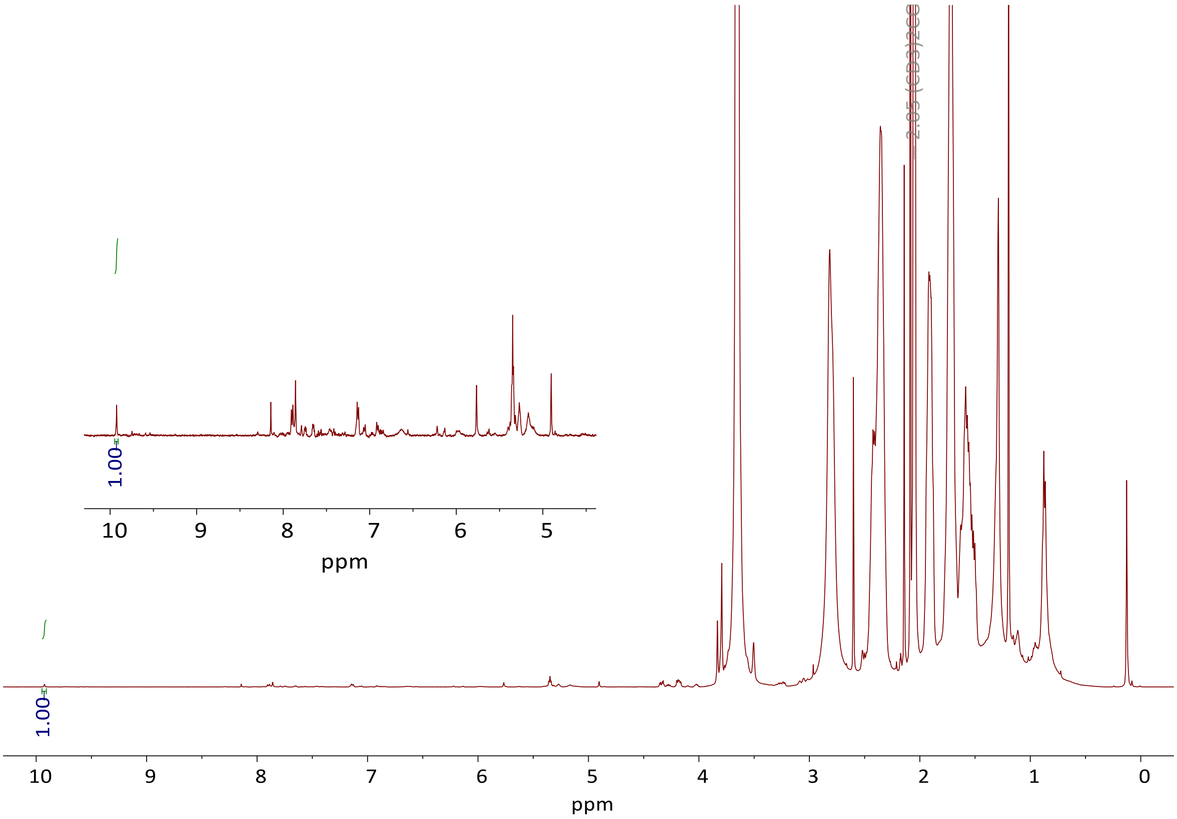

Spectrum S124. ^{1}H NMR (500 MHz, Acetone-d_6, 298 K) spectrum of post-BMG polymer $\mathbf{9}_{\text{-}114}$ before being washed with methanol.

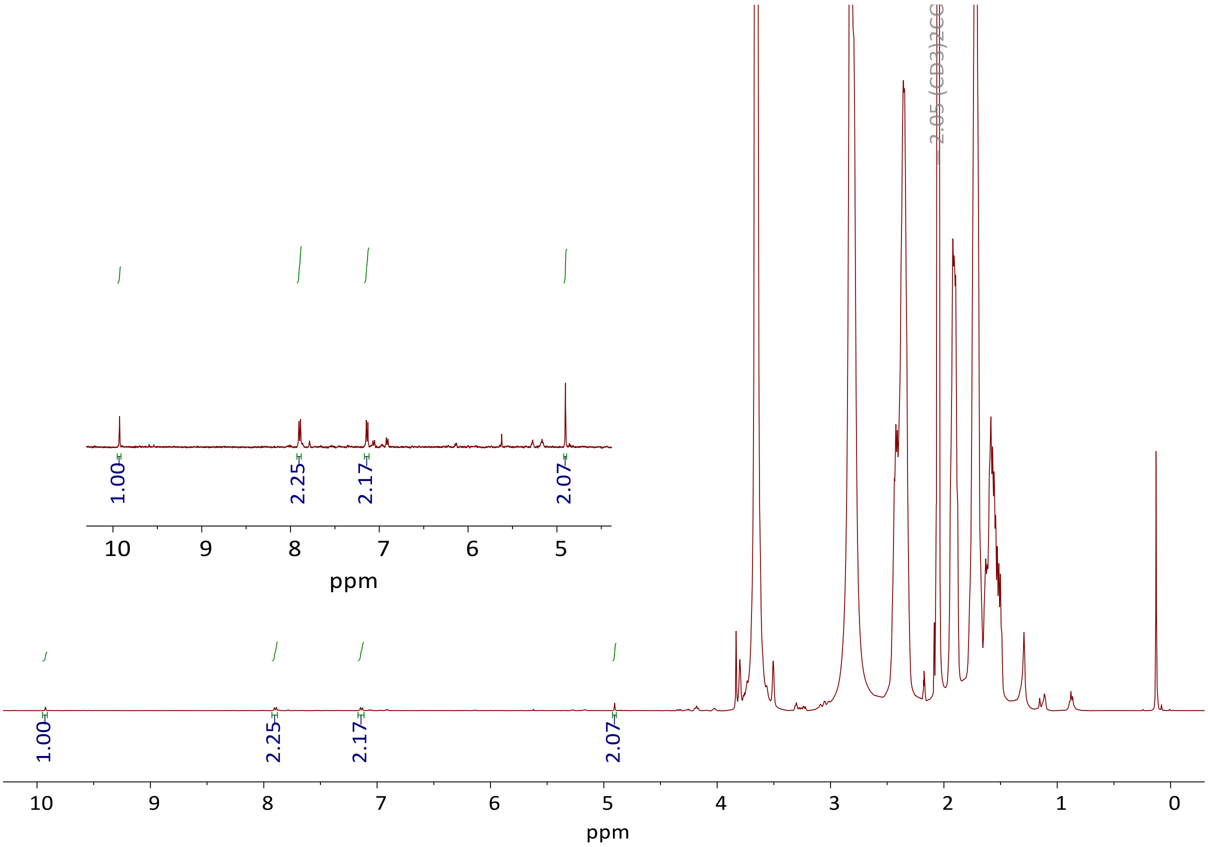

Spectrum S125. ^{1}H NMR (500 MHz, Acetone-d_6, 298 K) spectrum of post-BMG polymer $\mathbf{9}_{\text{-}114}$ after being washed with methanol.

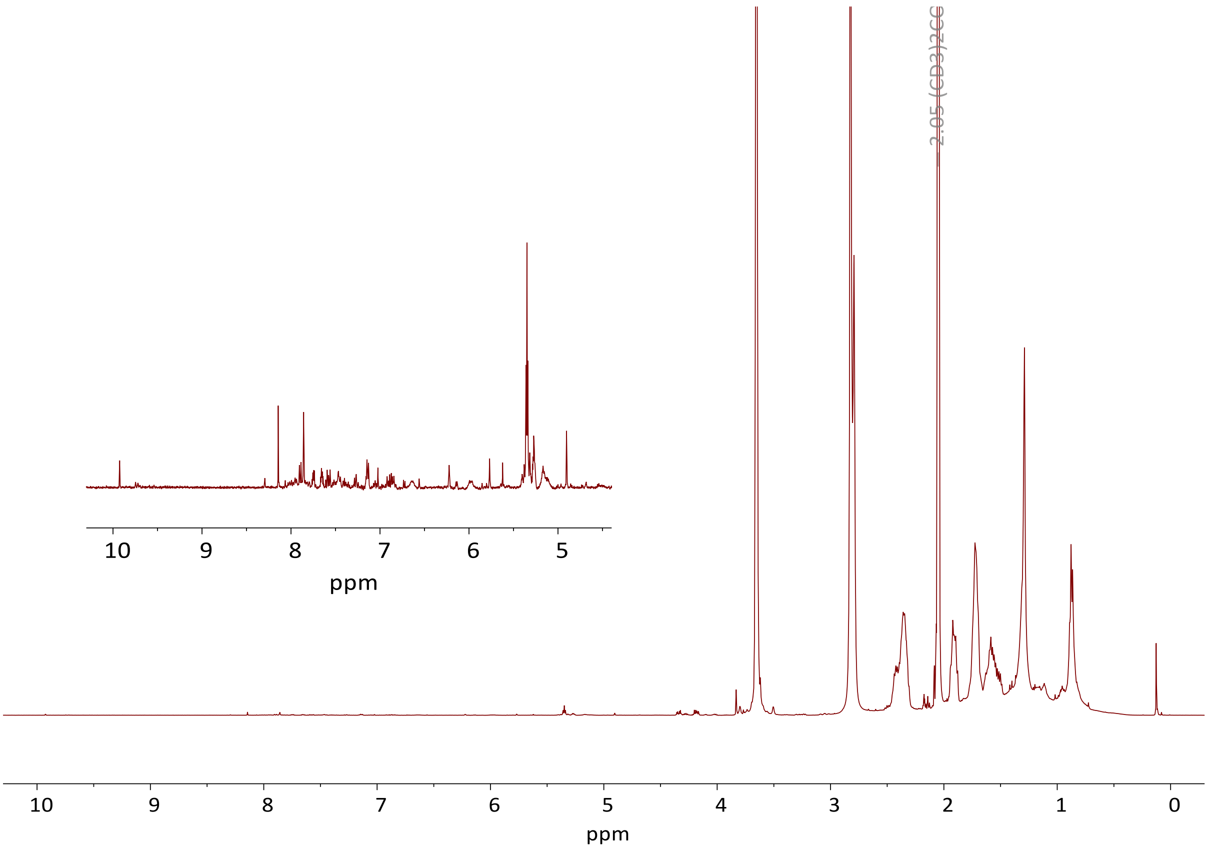

Spectrum S126. ^{1}H NMR (500 MHz, Acetone-d_6, 298 K) spectrum of the concentrated methanol washings from post-BMG polymer **9**$_{\mathbf{-114}}$.

10 Mass Spectrometry Isotopic Patterns

10.1 Isotopic distribution of S10

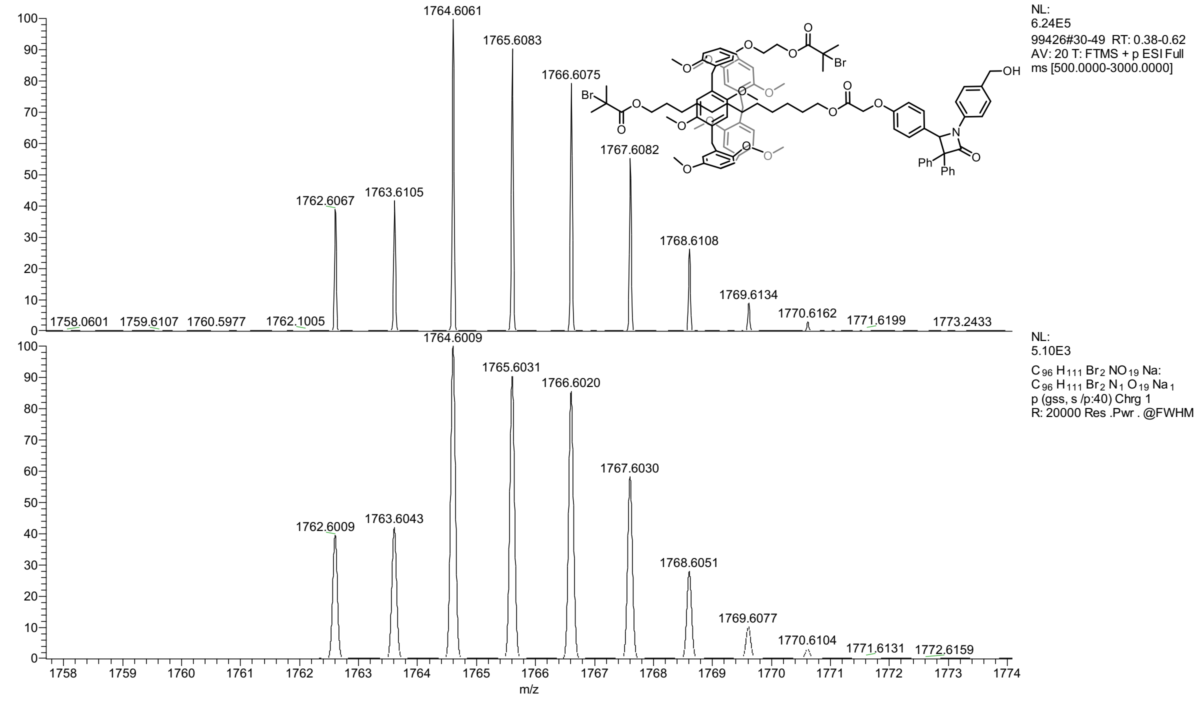

Spectrum S127. Isotopic distribution of **S10**. Top: Measured isotopic distribution for $C_{96}H_{111}Br_2N_2O_{19}Na$ ([M+Na]$^+$, +ESI). Bottom: Simulated isotopic distribution for $C_{96}H_{111}Br_2N_2O_{19}Na^+$.

10.2 Isotopic distribution of S11

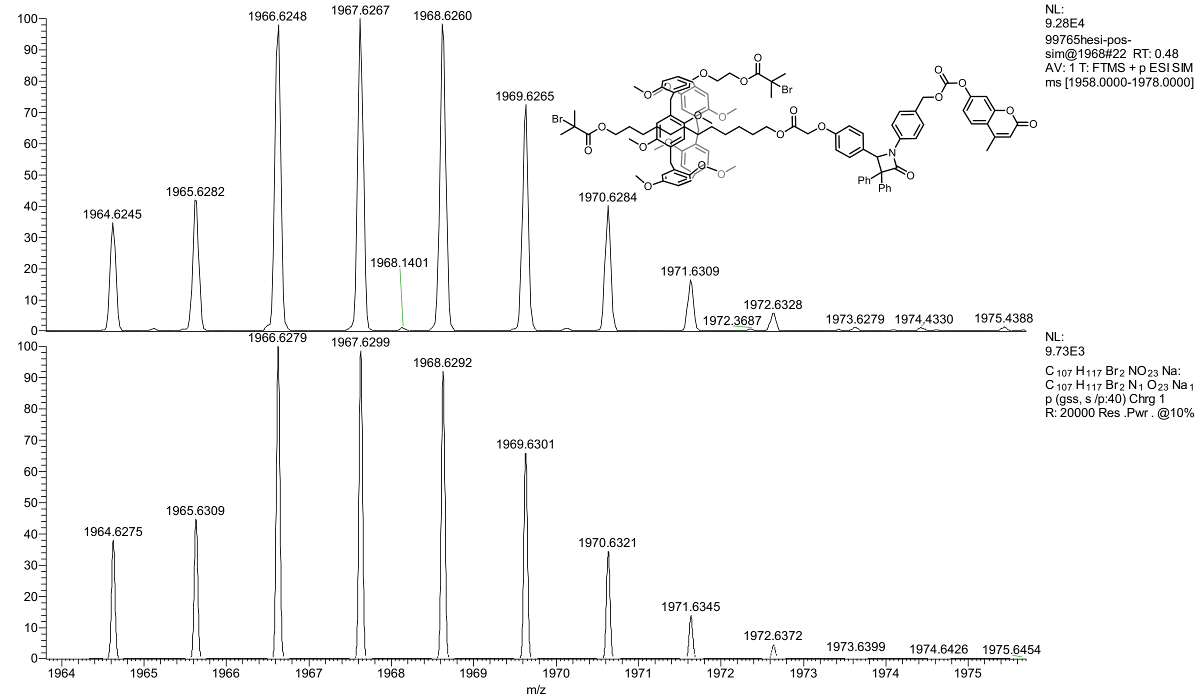

Spectrum S128. Isotopic distribution of **S11**. Top: Measured isotopic distribution for $C_{107}H_{117}Br_2NO_{23}Na$ ([M+Na]$^+$, +ESI). Bottom: Simulated isotopic distribution for $C_{107}H_{117}Br_2NO_{23}Na^+$.

10.3 Isotopic distribution of S12

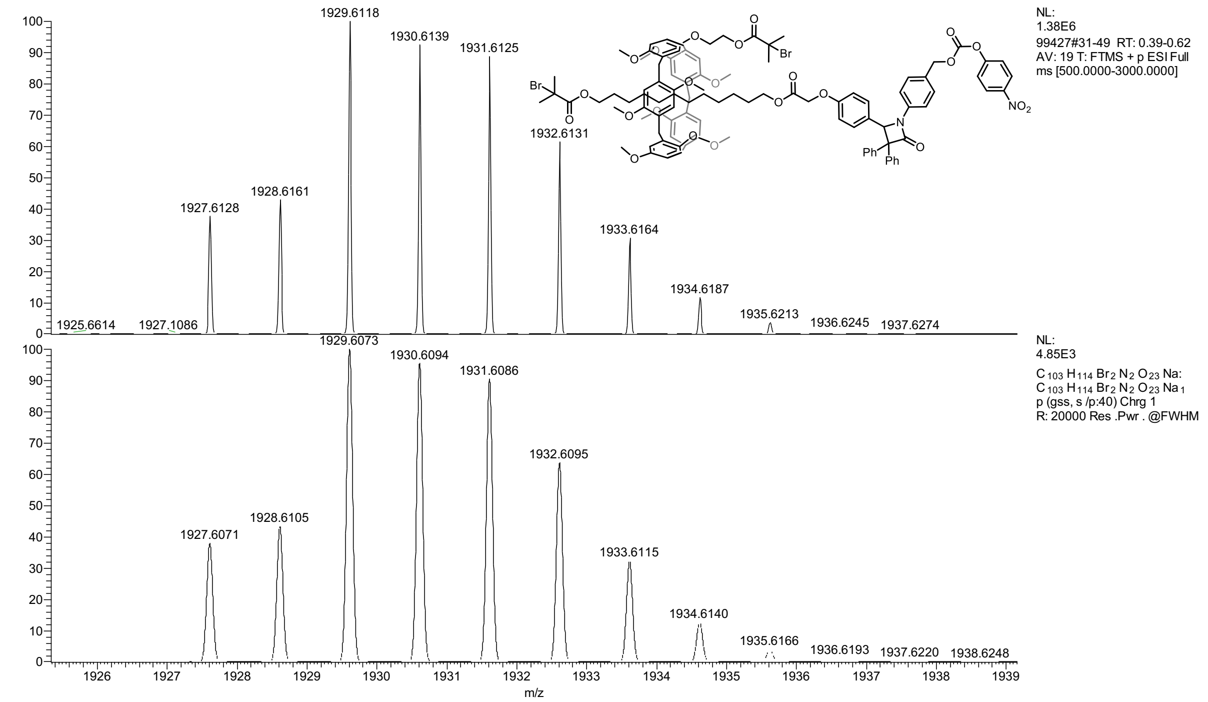

Spectrum S129. Isotopic distribution of **S12**. Top: Measured isotopic distribution for $C_{103}H_{114}Br_2NO_{23}Na$ ($[M+Na]^+$, +ESI). Bottom: Simulated isotopic distribution for $C_{103}H_{114}Br_2NO_{23}Na^+$.

10.4 Isotopic distribution of S14

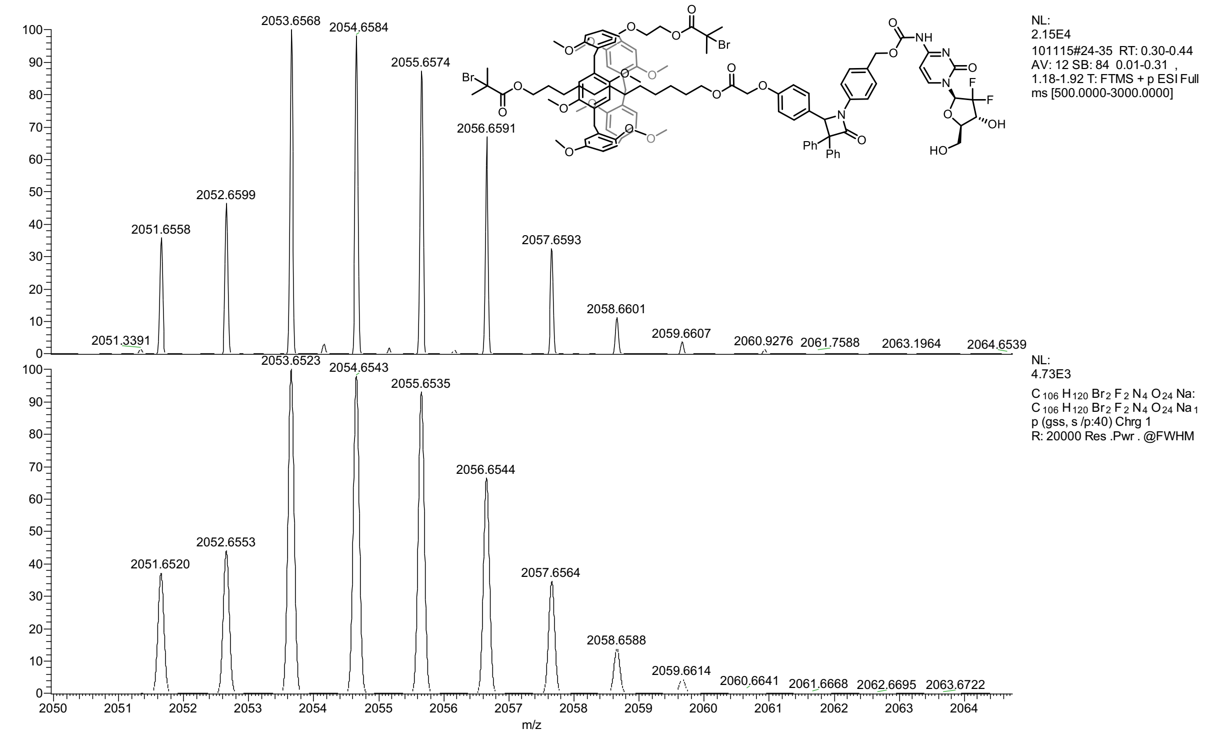

Spectrum S130. Isotopic distribution of **S14**. Top: Measured isotopic distribution for $C_{106}H_{120}Br_2F_2N_4O_{24}Na$ ($[M+Na]^+$, +ESI). Bottom: Simulated isotopic distribution for $C_{106}H_{120}Br_2F_2N_4O_{24}Na^+$.

10.5 Isotopic distribution of S16

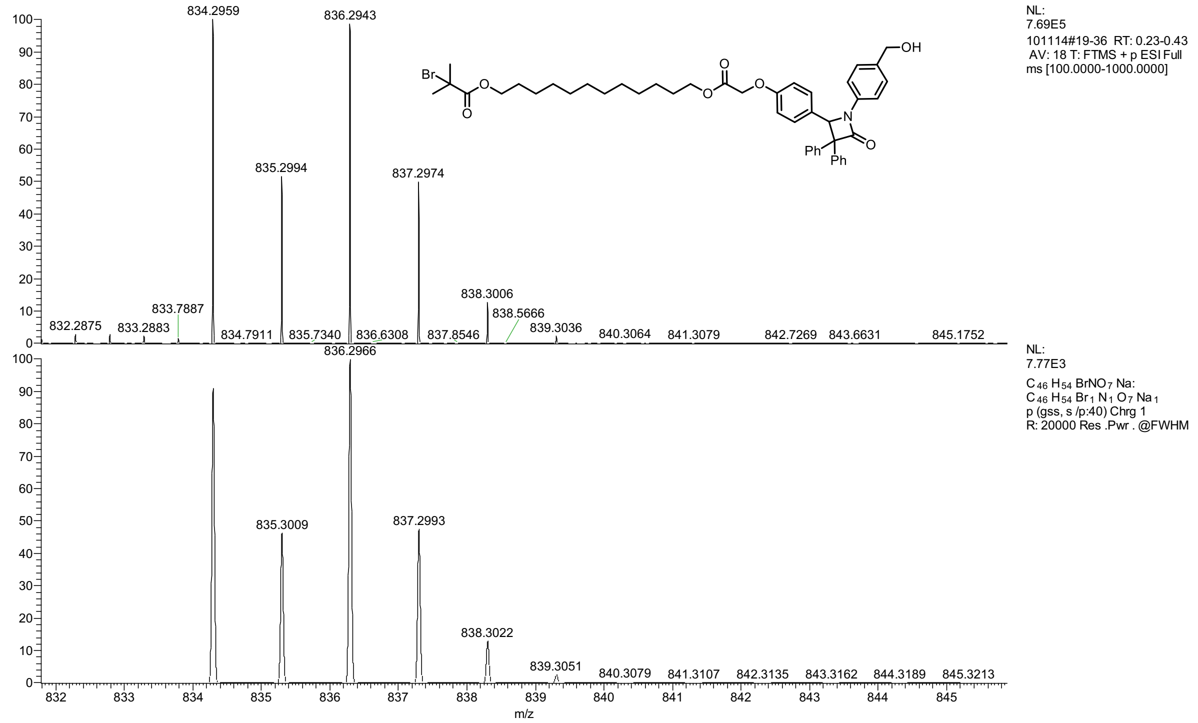

Spectrum S131. Isotopic distribution of **S16**. Top: Measured isotopic distribution for $C_{46}H_{54}BrNO_7Na$ ($[M+Na]^+$, +ESI). Bottom: Simulated isotopic distribution for $C_{46}H_{54}BrNO_7Na^+$.

10.6 Isotopic distribution of S17

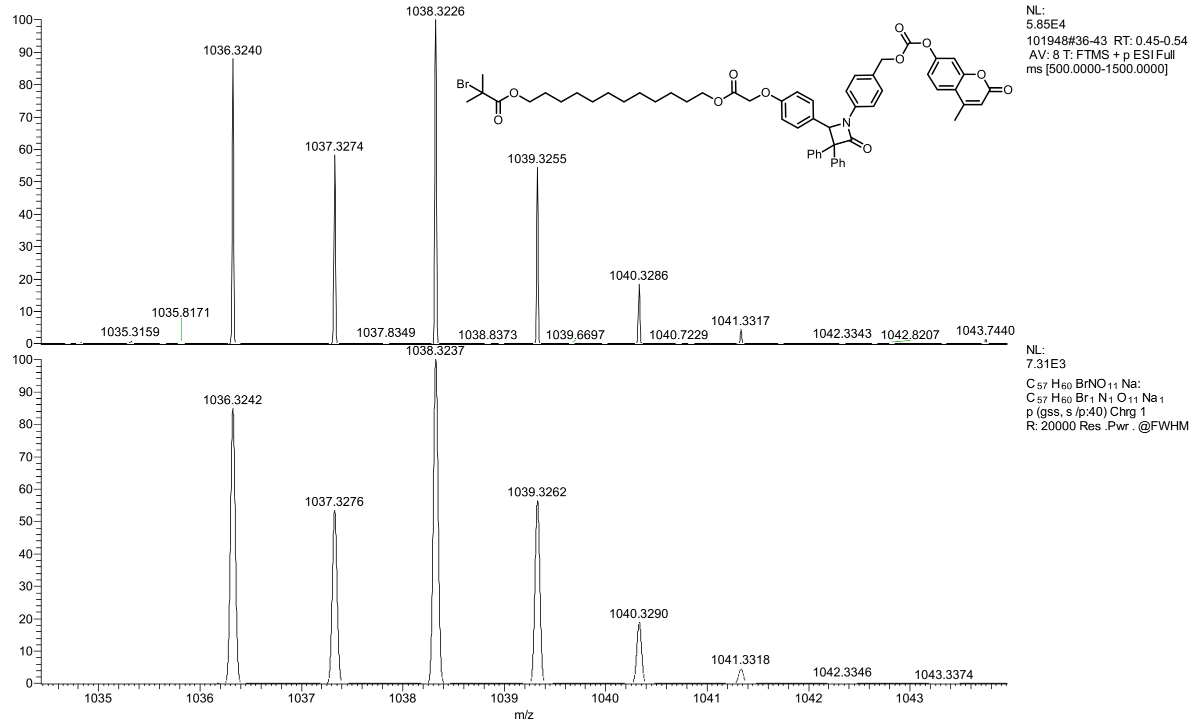

Spectrum S132. Isotopic distribution of **S17**. Top: Measured isotopic distribution for $C_{57}H_{60}BrNO_{11}Na$ ($[M+Na]^+$, +APCI). Bottom: Simulated isotopic distribution for $C_{57}H_{60}BrNO_{11}Na^+$.

11 References

(1) J. Bhaumik, Z. Yao, K. E. Borbas, M. Taniguchi, J. S. Lindsey, *J. Org. Chem.* **2006,** *71*, 8807.
(2) L. Chen, R. Nixon, G. De Bo, *Nature* **2024,** *628*, 320.
(3) A. P. Gorka, R. R. Nani, J. Zhu, S. Mackem, M. J. Schnermann, *J. Am. Chem. Soc.* **2014,** *136*, 14153.
(4) X. Li, Y. Hou, X. Meng, C. Ge, H. Ma, J. Li, J. Fang, *Angew. Chem. Int. Ed.* **2018,** *130*, 6249.
(5) L. Chen, G. De Bo, *Angew. Chem. Int. Ed.* **2025,** e202511039.
(6) C. R. Hickenboth, J. S. Moore, S. R. White, N. R. Sottos, J. Baudry, S. R. Wilson, *Nature* **2007,** *446*, 423.
